# Engineering β-sheets employing *N*-methylated heterochiral amino acids†
†Electronic supplementary information (ESI) available: Detailed experimental procedures, HPLC and MALDI traces, NMR spectra, DMSO-*d*_6_ titrations, coupling constants, chemical shifts, NMR structure calculations and overlay of structures. See DOI: 10.1039/c6sc00518g


**DOI:** 10.1039/c6sc00518g

**Published:** 2016-04-21

**Authors:** Dipan Ghosh, Priyanka Lahiri, Hitesh Verma, Somnath Mukherjee, Jayanta Chatterjee

**Affiliations:** a Molecular Biophysics Unit , Indian Institute of Science , Bangalore 560012 , India . Email: jayanta@mbu.iisc.ernet.in

## Abstract

There is a lack of functional group diversity in the reverse turn motifs nucleating a β-sheet conformation in designed peptides, proteins and foldamers. The majority of these sequences consist of d-Pro–l-Pro, d-Pro–Gly or Asn–Gly as the turn inducing motif restricting their biological application and physicochemical modulation. In this report, for the first time we elucidate that *N*-methylation of heterochiral amino acids in linear peptides nucleates β-sheet conformation without the necessity of having a ring or covalent constraint at the reverse turn. Our results show that d-Pro can be conveniently substituted by any other *N*-methylated d-amino acid followed by an *N*-methylated l-amino acid or sarcosine to adopt a βII′ turn inducing the β-sheet folding. Furthermore, we reveal that a single amino acid either at the *i* + 1 or *i* + 2 site of the reverse turn can modulate the right-handed twist, which eventually dictates the extent of the foldedness of the β-hairpin.

## Abstract 2

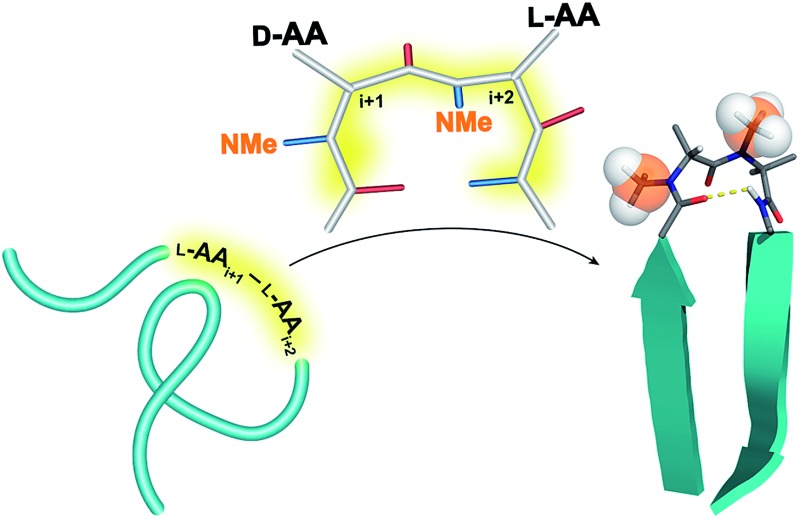
Engineerable β-turn motif is reported that modulates the extent of right-handed twist in β-sheets.

## Introduction

The design of β-hairpin forming peptides is of great importance in developing chemical tools that can perturb protein–protein/protein–peptide interactions in a chemical biology context^1^ and in developing metabolically stable, well-folded synthetic proteins from a protein engineering perspective.^2^ Extensive efforts to understand the design principles underlying the formation of reverse turns leading to the formation of β-hairpin peptides and β-sheets have established mainly two dipeptide motifs, d-Pro–Xaa (Xaa is any l-amino acid, although the preferred ones are l-Pro and Gly)^3^ and Asn–Gly, as the most favored β-hairpin nucleators in linear peptides.^4^ However, this severely limits the biological applications of β-hairpins, as the functionalization of these turn-inducing sequences either compromises their nucleating efficacy^5^ or involves tedious chemical synthesis,^6^ resulting in gross neglect towards the development of β-hairpins as chemical tools in contrast to α-helices.1*b* Thus, to expand the repertoire of reverse turn-inducing motifs we focused on employing *N*-methylated amino acids despite their inherent conformational flexibility.^7^

*N*-Methylation has been thoroughly investigated in cyclic peptides but its conformational impact on linear peptides is not well understood. We were thus keen to study the influence of *N*-methylation on the turn-inducing residues in linear β-hairpin peptides as there is no such report and the few known facts about the *N*-methylation of the turn residues are conflicting.^8^ In the present study, we have thoroughly investigated the influence of *N*-methylation on a varied selection of heterochiral residues in linear peptides using circular dichroism and NMR based structure calculations. We show that *N*-methylation indeed nucleates β-hairpin conformation irrespective of the amino acids present at the *i* + 1 and *i* + 2 positions of the β-turn. Furthermore, most of these hairpins are conformationally homogeneous^9^ in the NMR time scale in apolar and polar environments in spite of having consecutive *N*-methylated heterochiral amino acids. This indicates the wide applicability of the newly identified turn inducing motif towards peptide/protein engineering studies in lipophilic (*e.g.* membrane) and hydrophilic (*e.g.* cytosol) environments.^10^

## Results and discussion

**Fig. 1:**
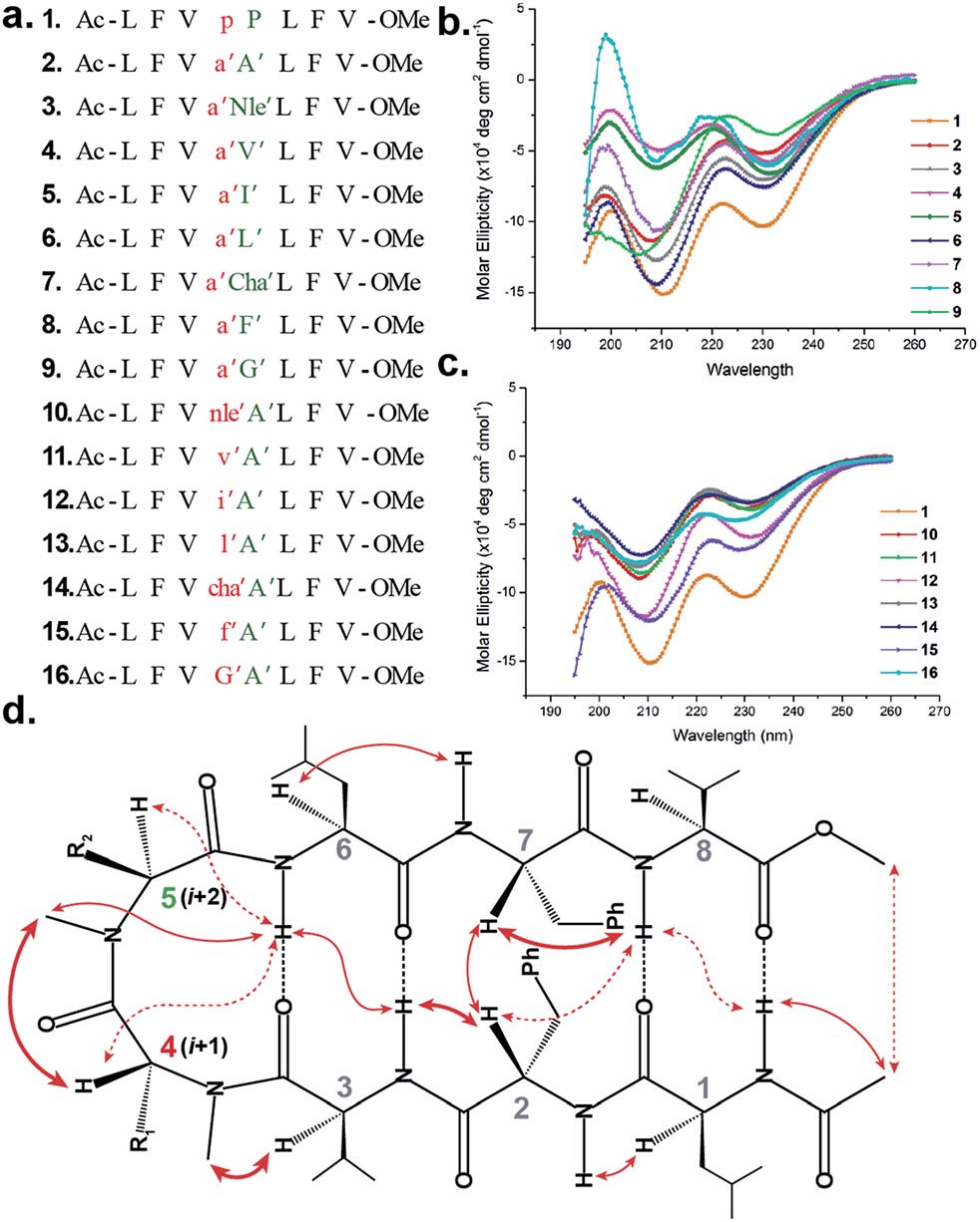
(a) Sequences of the model peptides synthesized in this study. The letters in lower case denote d-amino acid and the ′ denotes *N*-methylation. (b) CD spectra of the (*i* + 2) variants (**2–9**) and (c) (*i* + 1) variants (**10–16**) along with **1** in methanol. (d) The characteristic interresidue backbone NOEs observed in the synthesized peptides. Thick arrows denote distances between 1.8 and 2.2 Å, thin arrows denote 2.3–2.6 Å and dotted arrows denote 2.7–3.5 Å.

To initially study the influence of *N*-methylation on β-hairpin induction in apolar conditions, we selected the model octapeptide **1**,^11^ and substituted the d-Pro (*i* + 1) and l-Pro (*i* + 2) at the reverse turn with various *N*-methylated amino acids (Fig. 1a). We chose to *N*-methylate both the *i* + 1 and *i* + 2 residues to minimize the conformational entropy about the reverse turn.^12^

The hydrophobic amino acids: Ala, Val, Ile, Leu and Phe were chosen as substituents since they display the least propensity to occur at the β-turn region, according to Chou–Fasman's analysis of β-turns in protein structures.^13^ Besides, these residues also have a minimal stabilizing contribution from the side chain functionality on the turn conformation in the synthesized peptides. We also chose two unnatural amino acids: norleucine (Nle) which acts as a methionine isostere (methionine also has a low propensity to occur at the turn region) and a ring constrained leucine analog, cyclohexylalanine (Cha). All of the compounds were synthesized on a solid support and the *N*-methylation of the amino acids was performed on-resin using an optimized protocol.^14^ In order to determine whether any of our peptides undergo self-association under the conditions used for spectroscopic evaluation, we examined proton NMR chemical shift data for each peptide as a function of concentration. For the hydrophobic peptides, **1–16**, the chemical shifts measured at the concentration employed for 2D NMR studies (1–3 mM) were indistinguishable from the chemical shifts measured after 100-fold dilution. For the hydrophilic peptides, **17–21**, the chemical shifts measured at the concentration employed for 2D NMR studies were indistinguishable from the chemical shifts measured after ∼20-fold dilution.

The initial qualitative assessment of their potential to form β-hairpins was done using far UV (190–260 nm) CD measurements in methanol. The CD spectra of all of the *i* + 2 and *i* + 1 (Fig. 1) variants resembled the CD spectrum of **1**, showing the characteristic minima at 210 and 232 nm due to the anomalous behavior arising from the Phe2–Phe7 stacking interaction.4b,^15^ This clearly suggested that the replacement of the *i* + 1 and *i* + 2 residues in **1** with *N*-methylated amino acids was not detrimental to the overall topology of the molecule. However, as several compounds showed varying CD intensities with a single amino acid substitution, to understand the underlying cause we calculated their average solution conformation using restraints derived from ROESY. In the following discussion, the d-residues will be denoted in lower case and *N*-methylation with a prime symbol (′).

**Fig. 2:**
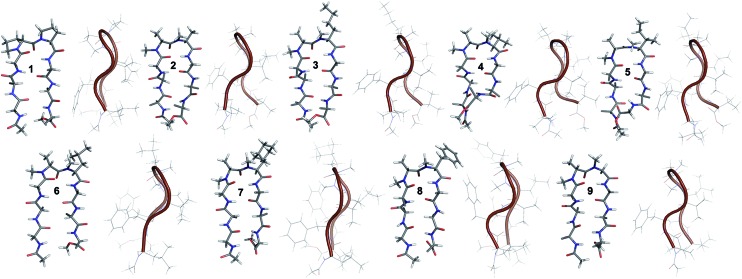
Solution structure of **1** and all of the *i* + 2 variants (**2–9**) in CDCl_3_. The front view of the peptide's backbone along with the side chains of the reverse turn motif are shown in a stick representation; all other side chains are restricted to C^β^ for the sake of clarity. The edge view is shown in a cartoon representation to depict the right-handed twist.

The end caps in **1–16** were carefully designed, so that if these were well folded, besides the characteristic NOEs (Fig. 1d) one would also expect the NOE between the end caps (NHAc and OMe) in the absence of strand fraying. The ^1^H NMR of **1** (in CDCl_3_) showed well dispersed signals in the H^α^ and H^N^ region with ^3^*J*_H^N^–H^α^_ > 8 Hz suggestive of a well-defined and extended conformation (Table S1†). The characteristic long-range NOEs Phe2H^α^–Phe7H^α^ and NHAc–OMe; strand NOEs Leu1H^α^–Phe2H^N^, Phe2H^α^–Val3H^N^, Leu6H^α^–Phe7H^N^ and Phe7H^α^–Val8H^N^ along with the turn region NOEs proH^α^–Pro5H^δ1/2^ and Pro5H^δ1^–Leu6H^N^ with the solvent shielded Leu1, Val3, Leu6 and Val8H^N^ (as determined by DMSO titration) (Table S2†) suggest the formation of a β-hairpin conformation. The solution structure of **1** (Fig. 2) revealed the formation of a β-hairpin with a centrally located βII′ turn (Table 1).

**Table 1:** Dihedral angles about the reverse turn in *i* + 2 variants obtained from the conformations generated by the restrained molecular dynamics simulation

	*i* + 1		*i* + 2	
	*Φ* (°)	*Ψ* (°)	*Φ* (°)	*Ψ* (°)
**1**	50 ± 14	–125 ± 13	–77 ± 8	43 ± 24
**2**	51 ± 11	–122 ± 15	–106 ± 14	25 ± 12
**3**	63 ± 7	–139 ± 6	–91 ± 8	23 ± 12
**4**	63 ± 8	–133 ± 8	–122 ± 9	48 ± 16
**5**	74 ± 9	–126 ± 15	–113 ± 12	37 ± 13
**6**	56 ± 11	–131 ± 9	–95 ± 8	14 ± 10
**7**	66 ± 11	–137 ± 10	–99 ± 10	35 ± 19
**8**	74 ± 8	–129 ± 7	–99 ± 9	20 ± 10
**9**	65 ± 7	–127 ± 6	–89 ± 10	15 ± 8

In **2**, the pP motif is substituted by the a′A′ motif and has a relatively less restricted *Φ*_(*i*+1 and *i*+2)_ at the reverse turn due to the lack of any ring constraint. Furthermore, the tertiary amide bonds have a low rotational barrier and thus **2** can co-exist in *cis*/*trans* form.^16^ However, we were surprised to note that **2** displayed a single conformation in CDCl_3_ with the absence of any (*i*^th^)H^α^–(*i* + 1^st^)H^α^ NOE confirming the presence of an all-trans conformer.^17^,^18^ It was interesting to note that most of the ^3^*J*_H^N^–H^α^_ values in **2** were higher than the corresponding values in **1**. The NOEs ala′H^α^–Ala′5NMe, ala′H^α^–Leu6H^N^ and Ala′5NMe–Leu6H^N^ along with solvent shielded Leu6H^N^ clearly define a βII′ turn.^18^ The long-range NOEs Leu1H^N^–Val8H^N^, Phe2H^α^–Phe7H^α^, Val3H^N^–Leu6H^N^ and NHAc–OMe additionally suggest the antiparallel strand registry in the molecule. The conformation of **2** (Fig. 2) revealed that the *Φ*_(*i*+1)_ and *Ψ*_(*i*+1)_ are quite close to the ideal βII′ turn (Table 1). Whereas, peptide **2a** with an A′A′ motif showed the presence of a *cis*-peptide bond between the 4^th^ and 5^th^ residue^18^ and the absence of any characteristic long-range NOEs defining the β-hairpin.

Compound **3**, with the long un-branched amino acid norleucine, displays all of the characteristic NOEs (Fig. 1d) with a comparable solvent accessibility of the HNs as observed in **2**. The conformation of **3** is quite close to **2** (backbone RMSD of 0.20 Å),^18^ with some variation in the dihedrals about the turn-motif. Compounds **4** and **5** both have an *N*-methylated β-branched amino acid at the *i* + 2 position and display a similar CD profile although with strikingly low intensity. However, we could assign the characteristic NOEs in both of the compounds, and the high ^3^*J*_H^N^–H^α^_ suggested the presence of a hairpin conformation. It was interesting to note that in these compounds the Leu1H^N^ and Leu6H^N^ are comparatively less solvent shielded than in **1**, **2** and **3** (Table S2†). In these β-branched analogs, the *i* + 1/*i* + 2 and *i* + 2/*i* + 3 amide planes are forced away from each other due to the steric repulsion between Val′5/Ile′5 C^γ^ and their respective *N*-methyl group resulting in higher values for *Φ*_(*i*+2)_ and *Ψ*_(*i*+2)_ (Table 1). The conformations of **4** and **5** are quite similar with a backbone RMSD of 0.45 Å and a stark right handed twist (Fig. 2).

Introduction of a γ-branched residue at the *i* + 2 position (**6**), results in less deviation from the optimal *Φ* and *Ψ* values (Table 1) in the turn region in comparison to the β-branched analogs. This is also evident from the CD spectrum of **6** showing a higher intensity than **4** and **5**. The ring constrained leucine isostere cyclohexylalanine substituted analog **7** and its aromatic congener **8**, with an *N*-methylated phenylalanine (although **8** shows an anomalous behavior in CD), display a similar solution conformation to **6**, suggesting the compatibility of the appended side chains at the *i* + 2 site.

Finally, we introduced an *N*-methylated glycine (sarcosine) (as it is achiral and lacks side chain) at the *i* + 2 position to assess its suitability in nucleating the β-hairpin. In spite of the absence of any side chain constraint, **9** exhibited conformational homogeneity in CDCl_3_ with all of the characteristic NOEs. To our surprise, the dihedral angles about the turn region of **9** (Table 1) in its solution conformation are quite similar to a designed β-hairpin peptide with a central pro–Gly turn motif in its crystal form.^19^ However, the solvent-shielded nature of the amide protons in **9** followed the trend that was observed for **5**, suggesting a flexible nature for both of the compounds. Compound **2** with the heterochiral *N*-methylated alanine in the turn region can be classified into both the *i* + 1 and *i* + 2 libraries. This gave us the clue that an *N*-methylated d-amino acid could potentially replace proline to form a stable β-hairpin structure. However, to assess the suitability of various branched and un-branched amino acids at the *i* + 1 position we chose to substitute this site with all of the aforementioned amino acids but with reversed chirality.

**Fig. 3:**
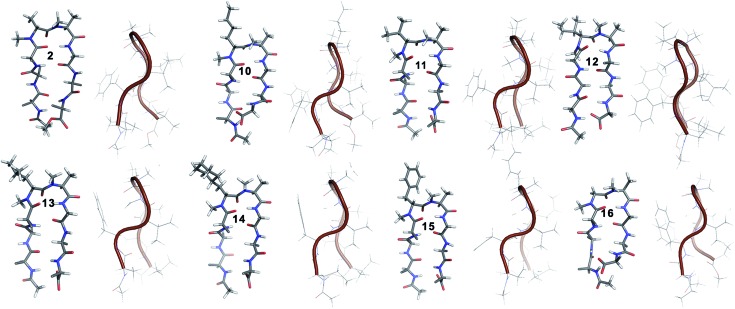
Solution structure of all of the *i* + 1 variants in CDCl_3_. The front view of the peptide's backbone along with the side chains of the reverse turn motif are shown in a stick representation; all other side chains are restricted to C^β^ for the sake of clarity. The edge view is shown in a cartoon representation to depict the right-handed twist.

**10** shows the entire signature NOEs of the reverse turn (nle′H^α^–Ala′5NMe, nle′H^α^–Leu6H^N^ and Ala′5NMe–Leu6H^N^) and displays the presence of a well-folded conformation (Leu1H^N^–Val8H^N^, Phe2H^α^–Phe7H^α^, Val3H^N^–Leu6H^N^ and NHAc–OMe). The solvent-shielded Leu1H^N^, Val3H^N^, Leu6H^N^ and Val8H^N^ along with the ^3^*J*_H^N^–H^α^_ > 8 Hz implies a putative β-hairpin structure. The structure of **10** (Fig. 3) is comparable to **2** and the norleucine swapped analog **3** with a backbone RMSD of 0.56 Å and 0.50 Å respectively.^18^

The β-branched *i* + 1 analog **11** showed a CD spectrum comparable to **10** (Fig. 1c) suggesting a structural similarity between these two analogs unlike in the corresponding *i* + 2 analogs. The characteristic NOEs and the high ^3^*J*_H^N^–H^α^_ revealed the structural integrity of the molecule. The *Φ*_(*i*+1)_ in **11** (Table 2) showed a significant increase that could be attributed to the steric interaction between the val′C^γ^ and val′NMe, which subsequently results in the reduced right handed twist of the hairpin unlike in **4**. A fairly good agreement between the solution structures of **11** and **10** (backbone RMSD of 0.39 Å) indicates the differential behavior of valine at the *i* + 1 and *i* + 2 sites.

**Table 2:** Dihedral angles about the reverse turn in *i* + 1 variants obtained from the conformations generated by the restrained molecular dynamics simulation

	*i* + 1		*i* + 2	
	*Φ* (°)	*Ψ* (°)	*Φ* (°)	*Ψ* (°)
**10**	73 ± 7	–121 ± 8	–105 ± 10	17 ± 10
**11**	86 ± 10	–118 ± 7	–106 ± 8	26 ± 9
**12**	93 ± 12	–112 ± 13	–107 ± 11	40 ± 17
**13**	67 ± 7	–131 ± 6	–90 ± 9	14 ± 7
**14**	69 ± 7	–135 ± 6	–88 ± 7	14 ± 7
**15**	65 ± 8	–126 ± 6	–93 ± 9	15 ± 8
**16**	75 ± 10	–104 ± 7	–128 ± 8	6 ± 5

Unlike **5**, compound **12** with the Ile′ shows a CD spectrum with a high intensity. The explanation was beautifully provided by the solution structure of **12**, where the *Φ*_(*i*+1)_ and *Ψ*_(*i*+2)_ deviate the most from the ideal geometry amongst all of the *i* + 1 analogs. The increased *Φ*_(*i*+1)_ is a result of the strong steric interaction between the ile′C^γ1^ and ile′NMe (Fig. 3) that eventually leads to a heavily restricted conformation about Ile′ as suggested by the multiple intra- and interresidue NOEs. This conformational restriction creates further steric clash between Ile′C^γ2^ and Ala′5NMe resulting in a higher *Ψ*_(*i*+2)_ value. This restriction is analogous to the ring constraint of Pro in **1**. A combination of these effects result in a flattened conformation in **12** that is comparable to **1** (backbone RMSD of 0.64 Å).

The CD spectrum of the γ-branched analog **13** is less intense than the CD spectrum of the corresponding *i* + 2 analog **6**. This observation is strikingly opposite to the trend observed for the β-branched analogs **5** and **12**. The solution structures of these compounds revealed the presence of a considerable right-handed twist in **13** as compared to **6**, validating the CD spectrum. The basis of this twist in **13** is the steric interaction between the isopropyl and the *N*-methyl group of leu′ resulting in a slightly higher value of *Φ*_(*i*+1)_ in **13** than in **6**. On the contrary, the steric repulsion between the isopropyl and the *N*-methyl group of leu′ in **6** is considerably less due to the greater torsion angle of C^(NMe)^–N*–*C^α^–C^β^_(*i*+2)_ than C^(NMe)^–N*–*C^α^–C^β^_(*i*+1)_ (due to the opposite stereochemistry at the *i* + 1 and *i* + 2 site) resulting in the flattened hairpin in **6**.

Surprisingly, the CD spectrum of **14** showed a lower intensity in comparison to all other *i* + 1 analogs. Nevertheless, we could identify the characteristic NOEs, and the solution conformation of **14** is almost identical to that of **13** (backbone RMSD of 0.24 Å) displaying the right handed twist. On the other hand the aromatic *i* + 1 γ-branched analog **15** shows the most intense CD spectrum amongst all of the *i* + 1 congeners indicating the occurrence of a flatter hairpin. However, the conformation of **15** and the solvent exposure of amide protons show a striking similarity with the corresponding *i* + 2 analog **8** (backbone RMSD of 0.26 Å), which shows a less intense CD spectrum. This re-emphasizes the anomalous behavior of the electronic CD for peptides with aromatic residues.^20^ Furthermore, we did not observe any aggregation at the concentrations used for NMR and CD measurements,^18^ therefore the extent of twist in these peptides arises mainly due to the substitution pattern at the reverse turn.

**Fig. 4:**
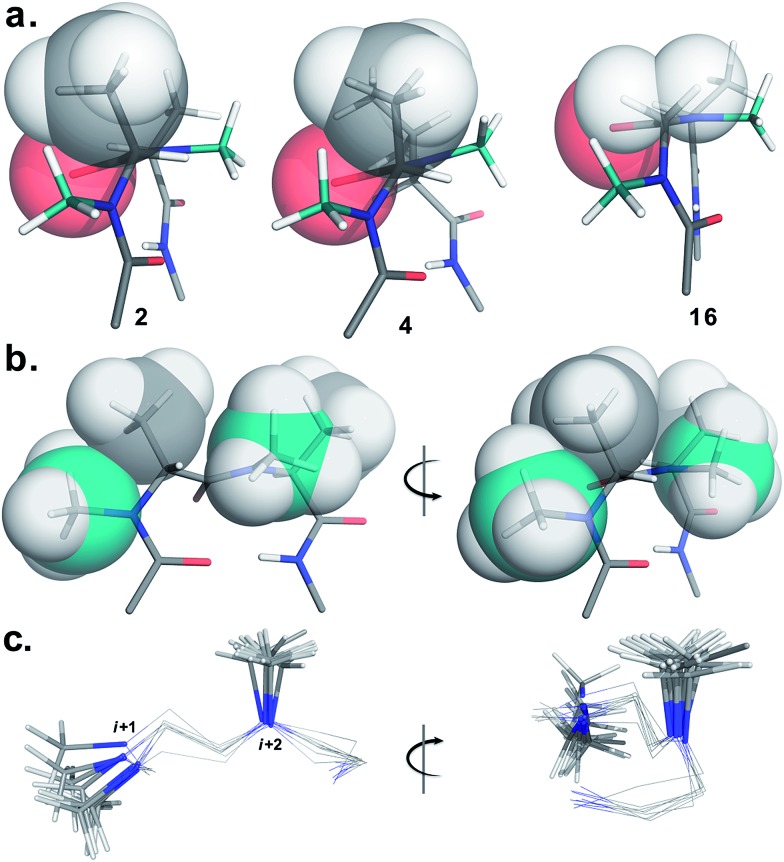
Critical factors determining the reverse turn stability. (a) The relative orientation of the *i* + 1 C^β^ and *i* + 1 CO is depicted in three different substitution patterns. In spite of the strong steric repulsion at the *i* + 2 site in **4** the relative orientation of the *i* + 1 C^β^ and *i* + 1 CO does not alter. However, it effectively alters the orientation of the *i* + 2 C^β^ resulting in a twist in the structure (note the parallel orientation of the strands in **2** and **16**). However, the absence of *i* + 1 C^β^ in **16** relaxes the steric repulsion between *i* + 1 H^α^ and *i* + 1 CO resulting in enhanced conformational flexibility. (b) The relative orientation of the *i* + 1 *N*-methyl, *i* + 1 C^β^, *i* + 2 *N*-methyl and *i* + 2 C^β^ groups that are responsible for the induction and the stability of the β-hairpin conformation (*e.g.* front and side view of the reverse turn in **2**). (c) Overlay of the βII′ turn of **1–9** depicting the spatial distribution of the *i* + 1 and *i* + 2 *N*-methyl groups (front and side view). The conformational space spanned by the *i* + 1 *N*-methyl group is relatively larger than the *i* + 2 *N*-methyl group. Only the *N*-methyl groups in the turn motif are represented as sticks for the sake of clarity.

To determine the importance of the *i* + 1 side chain on the induction of the βII′ turn and the subsequent folding of the hairpin, we synthesized compound **16** with sarcosine at the *i* + 1 position. Although the CD spectrum of **16** is comparable to other *i* + 1 analogs, it has considerably less intensity than the CD spectrum of **9**. This observation directly correlates with the flexibility of **16** in CDCl_3_,^18^ although the major conformer displays all of the characteristic signatures (NOEs, ^3^*J*_H^N^–H^α^_ and solvent shielded H^N^) of a β-hairpin. It is interesting to note that in spite of the absence of any side chains at the *i* + 1 site, there is a clear indication of a βII′ turn as suggested by the following NOEs: Gly′H^α1^–Ala′5NMe, Gly′H^α1^–Leu6H^N^, Ala′5NMe–Leu6H^N^ and a shorter distance for Ala′5NMe–Ala′5H^β^ than Ala′5NMe–Ala′5H^α^. However, there is a substantial increase in the *Φ*_(*i*+1)_ and *Φ*_(*i*+2)_ values. A close assessment of the structure revealed the separation of *i* + 1/*i* + 2 and *i* + 2/*i* + 3 amide planes to a greater extent in **16** than all of the other analogs including the most sterically demanding *i* + 2 β-branched analogs, **4** and **5**. This is mainly due to the absence of *i* + 1 C^β^ in **16**, which restricts the inward rotation of the *i* + 1/*i* + 2 amide plane in the other analogs due to the van der Waals repulsion with the *i* + 1 CO group (Fig. 4a) (note the greater torsion angle C^β^–C^α^–C–O_(*i*+1)_ in **2** and **4** in comparison to H^α(Pro–R)^–C^α^–C–O_(*i*+1)_ in **16**).

The tolerance of various *N*-methylated chiral or achiral amino acids at the turn region clearly indicates that the steric interactions in the reverse turn are crucial in nucleating the β-hairpin that is subsequently stabilized by the intramolecular hydrogen bonding.^21^ The relative orientations of the *i* + 1 *N*-methyl, *i* + 1 C^β^, *i* + 2 *N*-methyl and *i* + 2 C^β^ along with the *i* + 1 CO are critical in dictating the stability of the β-turn (Fig. 4b). Any relaxation in the van der Waals repulsion about these residues destabilizes the global conformation of the hairpin. This is clearly evident from **16**, where the absence of *i* + 1 C^β^ destabilizes the global conformation. The torsion angles C^(NMe)^–*N–*C^α^–C^β^_(*i*+1)_ and C^(NMe)^–*N–*C^α^–C^β^_(*i*+2)_ also play a critical role in stabilizing the reverse turn as observed in the γ-branched analogs **6** and **13**. Finally, the conformational flexibility of the *N*-methyl group at the *i* + 1 position (Fig. 4c) allows for a better tolerance of bulkier substituents (*e.g.* β-branched amino acids) at the *i* + 1 site than at *i* + 2. This leads to the differential behavior of certain substituents at these two sites (*e.g.***4** and **11**).

Next, to assess the suitability of the designed reverse turn motif in the context of protein engineering, we studied several hydrophilic peptides with a common strand sequence^22^ but with varied turn inducing motifs. We were keen to understand the behavior of our turn inducing motif in aqueous conditions, as only a few reverse turn motifs have found utility in protein engineering.^23^ We initially synthesized **17**, with d-Ala–l-Ala as the turn inducing motif, since in small cyclic peptides heterochirality is enough to induce the formation of a βII′ turn.1*a*

**Fig. 5:**
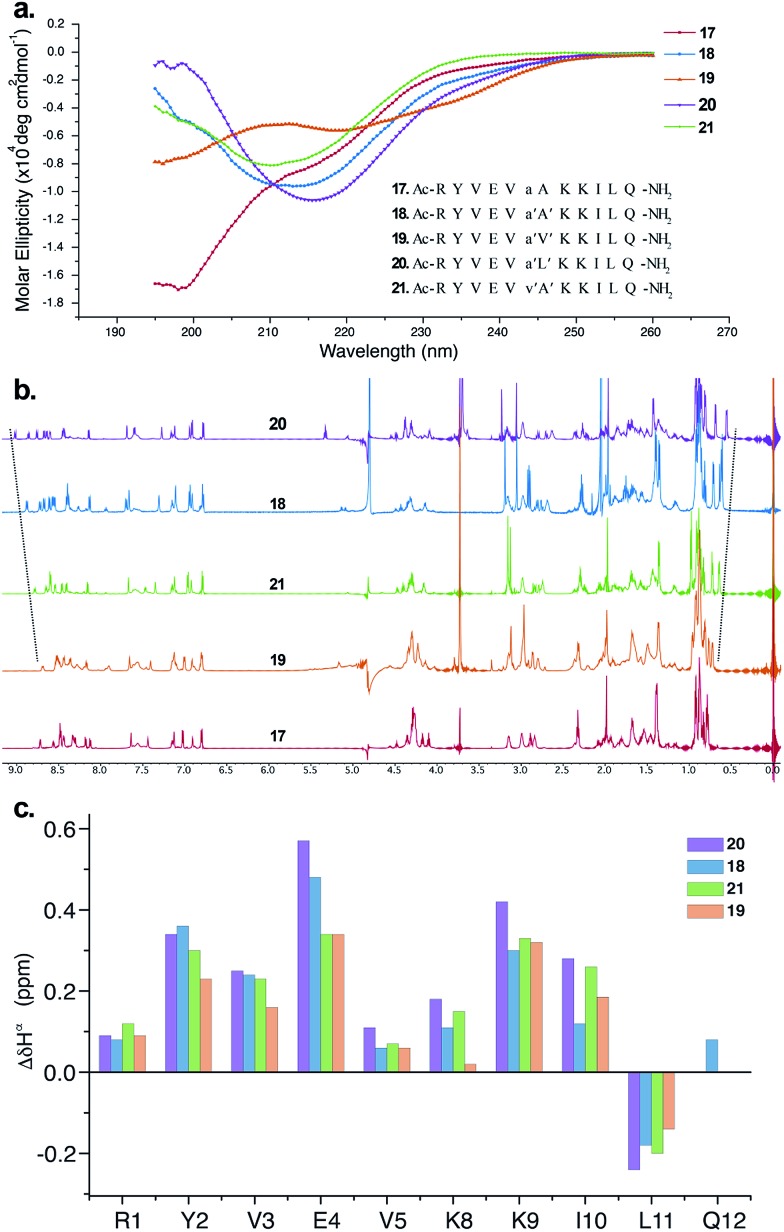
(a) The CD spectra of the hydrophilic peptides in acetate buffer (pH 3.8) and the synthesized sequences **17–21** (inset), which are color-coded. (b) ^1^H NMR spectra of **17–21** showing the gradual upfield shift of Leu11CH_3_^γ^ as well as the gradual downfield shift of the Ile10H^N^. (c) Comparison of Δ*δ*H^α^ chemical shifts of the strand residues in **18–21** (obtained from the respective unfolded control peptides), showing the modulation of the folded conformation by a single amino acid substitution (assignment of Gln12H^α^ in **19**, **20** and **21** was not possible due to resonance overlap).

The CD spectrum of **17** (Fig. 5a) showed the signature of a random coil, suggesting that heterochirality in a linear peptide is not sufficient to induce a hairpin formation. To emphasize the role of *N*-methylation in inducing the β-hairpin formation, we synthesized **18**, with *N*-methylation at d-Ala and l-Ala. The CD spectrum of **18** showed a broad minima centred around 215 nm that is characteristic of a β-sheet structure (Fig. 5a). Further, to show the compatibility of different amino acid side chains at the reverse turn of the β-hairpin, we synthesized **19**, **20** and **21**. It was gratifying to note that the CD spectrum for these analogs followed a similar pattern as observed in the hydrophobic peptides, with the *i* + 2 γ-branched amino acid (**20**) showing the maximum and the β-branched analog (**19**) showing the minimum intensity. It was also encouraging to observe the differential behavior of the *i* + 2 and *i* + 1 β-branched analogs **19** and **21**, respectively, that followed the trend as noted for the hydrophobic peptides.

To obtain additional insights into the folding behavior of these compounds, we performed NMR spectroscopy in acetate buffer (pH 3.8). The ^1^H spectrum of **17** showed clear indications of an unfolded structure, with the absence of any upfield shifted methyl groups^24^ and overlapping resonance for the β-methyl groups (∼1.4 ppm) of alanine (Fig. 5b). On the contrary, all of the *N*-methylated analogs show the presence of two upfield shifted methyl groups that progressively increase in the order **19** < **21** < **18** < **20**. This order also correlated with the enhanced dispersion of chemical shifts in the amide region of these compounds.

The secondary chemical shifts of these compounds (obtained from the respective unfolded controls, where the *N*-methylated d-residue was substituted with an *N*-methylated l-residue)^18^ also follows the order **19** < **21** ≈ **18** < **20**, suggesting the increased foldedness of the β-sheet from left to right.25*a* The results from the NMR analyses corroborated the different intensities observed in the CD spectrum. Together these results strongly indicate the subtle modulation of the β-sheet folded conformation *via* a single substitution at the *i* + 1 or *i* + 2 site, which would find enormous utility in protein engineering.

**Fig. 6:**
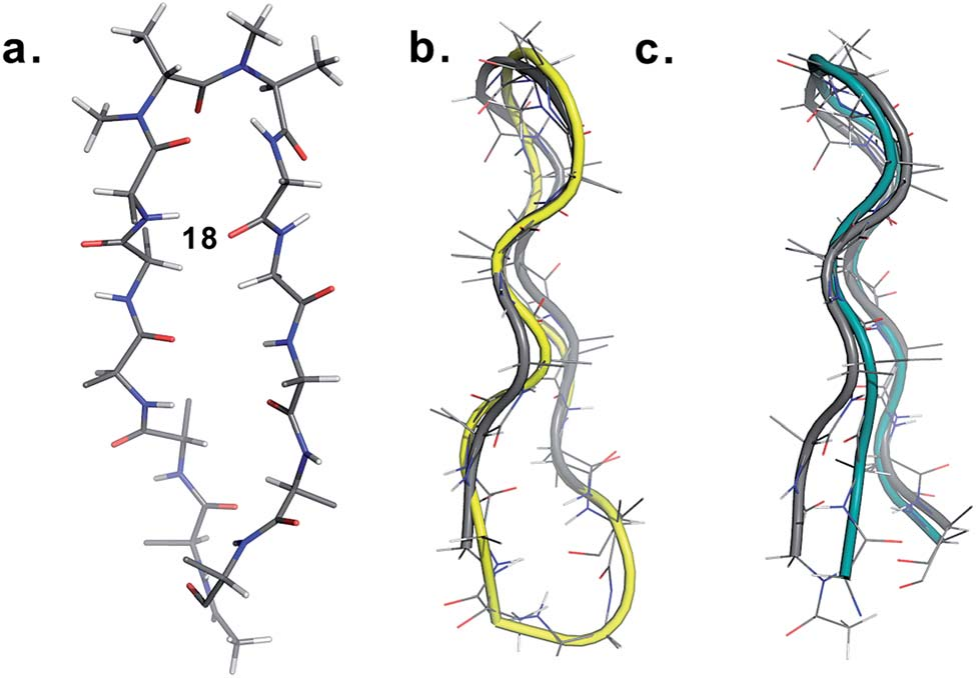
(a) Structure of **18** in aqueous solution and backbone overlay (in grey) with (b) a cyclic β-sheet peptide TS+ (yellow) (RMSD 1.0 Å) and (c) an engineered GB1 peptide (teal) (RMSD 0.75 Å).

Finally, to validate the broad scope of our design strategy in foldamer design, we calculated the solution structure of **18** at 25 °C. The conformation of **18** (Fig. 6a) was well-defined by several inter- and intraresidue NOEs. The reverse turn was specified by ala′H^β^–ala′NMe, ala′H^α^–Ala′7NMe, ala′H^α^–Lys8H^N^, and Ala′7NMe–Lys8H^N^ NOEs, while Tyr2H^α^–Leu11H^α^, Tyr2H^α^–Gln12H^N^, and Val5H^N^–Lys8H^N^ NOEs confirmed the strand registry.^18^ The structure of **18** was calculated utilizing the distances derived from the NOEs, which showed the presence of a β-hairpin conformation with a central βII′ turn. It was gratifying to see that **18** displayed significant structural similarity with two different β-sheet peptides^25^ in aqueous conditions (Fig. 6b and c).

## Conclusions

In conclusion, we show that the *N*-methyl groups in conjunction with the C^β^ at the *i* + 1 and *i* + 2 amino acid side chains provide sufficient steric locking to fold a linear peptide into a β-hairpin. The introduction of heterochirality in linear peptides alone is not enough to induce the formation of β-sheets, unlike in cyclic peptides. Our engineering strategy is quite modular in terms of decorating the reverse turn with different functional groups to alter the physicochemical properties of the designed β-sheets and attach various probes for biophysical studies. Furthermore, branched amino acids at the reverse turn add another dimension to its modularity by introducing a varying extent of twist that could be utilized to probe the structure activity relationship of designed β-sheets and the modulate folding of proteins. *N*-methylation has found tremendous utility in cyclic peptides, however, its use in linear peptides was limited. With this report we strongly believe that this simple strategy will not only find enormous utility in foldamer design^26^ and protein engineering but also in the development of novel materials^27^ and peptide based catalysts.^28^

## Supplementary Material

Supplementary information
