# Supplementary material for: Engineering β-sheets employing N-methylated heterochiral amino acids
Source: Chem Sci. 2016 Apr 21;7(8):5212–8. doi: 10.1039/c6sc00518g (PMC5784515; doi:10.1039/c6sc00518g)
Supplement: Supplementary file 1 [file SC-007-C6SC00518G-s001.pdf]

Electronic Supplementary Material (ESI) for Chemical Science.
This journal is © The Royal Society of Chemistry 2016

Supplementary Information

Engineering β-Sheets Employing N-Methylated Heterochiral Amino Acids

Dipan Ghosh, Priyanka Lahiri, Hitesh Verma, Somnath Mukherjee and Jayanta Chatterjee*

Molecular Biophysics Unit, Indian Institute of Science, Bangalore 560012, India

Table of Contents:

1. Materials and Methods……………………………………………….S2
2. Characterization Data of **1-16**……………………………………..S6
3. Characterization Data of **2a**………………………………………S19
4. Table S1 : Coupling Constant of **1** to **16**………………………...S109
5. Table S2 : DMSO Titration Slope……………………………….S109
6. Characterization Data of **17-21, 18a-21a**………………………..S110
7. Table S3 : Chemical shifts table of compounds **17** to **21**………..S124
8. Table S3a : Chemical shifts table of compounds **18a** to **21a**…...S126
9. Table S4 :Coupling Constants of **18** and **20**…………………….S126
10. Structure Calculation for compound **18**………………………...S127
11. Backbone Overlayed Structures………………………………...S131
12. References……………………………………………………….S135

Materials and Methods:

General Information

All the Fmoc and orthogonally protected amino acids, (1[Bis(dimethylamino)methylene]-1*H*-1,2,3-triazolo[4,5-*b*] pyridinium 3-oxid hexafluorophosphate) (HATU), 1-hydroxy-7-azabenzotriazole (HOAt), 1-hydroxybenzotriazole (HOBt) were purchased from GL Biochem, Shanghai, China. 2-Chlorotrityl chloride polystyrene (2Cl-TCP) and Rink Amide AM resin were also purchased from GL Biochem, Shanghai, China. *N*,*N'*-Diisopropylcarbodiimide (DIC), *N*,*N*-diisopropylethylamine (DIPEA), Trifluoroacetic acid (TFA), Trifluoroethanol (TFE), Triisopropylsilane (TIPS), Triphenylphosphine, anhyd. tetrahydrofuran (THF), anhyd. methanol, Diisopropyl azodicarboxylate (DIAD), 1,8-Diazabicyclo[5.4.0]undec-7-ene (DBU), 2-Mercaptoethanol, Glacial acetic acid, *N*,*N'*-Dicyclohexylcarbodiimide (DCC), N-methyl-2-pyrrolidone (NMP) , Thionyl Chloride, Calcium hydride and piperidine were purchased from Sigma-Aldrich. All the above reagents were used as commercially supplied. Solvents for RP-HPLC were purchased as HPLC grade and used without further purification. Dichloromethane was dried with Calcium hydride. All the other solvents were used as commercially supplied.

All the reactions were performed in oven-dried glass apparatus. Reactions on solid support were carried out in plastic syringes (10 ml) fitted with a frit column plate.

High-resolution mass spectra were recorded on a Bruker Daltonics ESI Q TOF- (Maxix Impact) with Nano LC (Proxeon easy nLC) mass spectrometer. ESI mass spectra were recorded in positive ion mode on a HCTultra ETD II ion trap spectrometer (PTM Discovery System, Bruker Daltonics, Germany). MALDI mass spectra were recorded on UltrafleXtreme TOF/TOF (Bruker Daltonics, Germany) and the data were processed and analysed using the Flex Analysis 3.1 software.

Nuclear magnetic resonance (NMR) spectra were recorded either on a 700 MHz Bruker Avance spectrometer (Bruker, Karlsruhe, Germany), or a 600 MHz Agilent NMR spectrometer at 298K.

Analytical RP-HPLC was performed on a Shimadzu UFLC system equipped with Prominence Diode Array (PDA) UV Detector at 210 and 254 nm using an analytical column (Phenomenex C18, 250 mm x 4.6 mm I.D., 5 μm) at a flow rate of 1 mL min^{-1}. Purifications were performed using a semi-preparative column (Phenomenex C18, 250 mm x 10 mm I.D., 5 μm) at a flow rate of 4 mL min^{-1}.

Circular Dichroism (CD) spectra were acquired on a JASCO-715 spectropolarimeter using 0.1cm path length cuvette. The CD spectra were averaged over 3 scans and the baseline correction was done by subtraction of the spectrum with the appropriate blank solution.

Peptide Synthesis

Peptides **1** to **16** were synthesized on TCP resin (1.3 $mmolg^{-1}$), using standard Fmoc-based chemistry[1]. The penultimate C-terminal amino acid residues, Fmoc-Phe-OH (1.25 equiv , **1-16**) were loaded on to the resin with 2.5 equiv DIPEA in anhydrous DCM (4 mL) at room temperature. After loading the first amino acid, the remaining unreacted

trityl chloride groups bound to the solid support were capped using methanol (200 µl/100 mg resin) for 15 min. Next, the resin was thoroughly washed with DCM (3 times), 1:1 DCM-methanol (3 times) and methanol (3 times) and finally dried under vaccum. The loading capacity was estimated from the dry weight of the resin, which ranged from 0.6-0.8 $mmolg^{-1}$. The elongation of the rest of the peptide was performed on 150 mg (0.09-0.12 mmol) scale with DIC/HOBt as the coupling agents (2.5 equiv). Fmoc deprotections were carried out with 20% piperidine (5 min x 1, 15 min x 1) in DMF.

Peptides **17** to **21,** including peptides **18a** to **21a** were synthesized on Rink Amide AM resin (0.8 $mmolg^{-1}$) on 200 mg scale (0.16 mmol) using standard Fmoc-based strategy. The resin was swollen in DMF and deprotected with 20% piperidine in DMF (5 min x 1, 15 min x 1) followed by thorough washing with DMF (3 times). The C-teminal amino acid, Fmoc-Gln(Trt)-OH (3.5 eq.) was loaded onto the resin by using standard coupling reagents (3.5 equiv HOBt, 3.5 equiv DIC) in DMF for 2 hours at room temperature. The entire peptide was assembled with this same protocol as well.

N-Methylation

A modified protocol for Mitsunobu reaction on the solid support was utilized for selective N-methylation of amino acid residue.[2]

Coupling of the amino acid residue following the N-methylated amino acid

Coupling of Fmoc-Xaa-OH to the free N^{α}-methylamine terminal of the peptides on the resin was carried out using 3 equiv each of HOAt, HATU and Fmoc-Xaa-OH and 6 equiv of DIPEA in DMF at room temperature.[3]

N-terminal Acetylation of the Peptide

After the final Fmoc deprotection of the peptides **1** to **21**, the N-term was acetylated with pyridine and acetic anhydride (9:1) for 15 mins (3 times) at room temperature. The resin was then washed thoroughly with DMF (5 times) followed by DCM (2 times).

Solution-phase Synthesis of Valine-OMe

Following a literature procedure[4] valine methyl ester was prepared by reacting valine (5 gm) with 50 mL methanol in presence of 9.1 mL of $SOCl_2$. To a cooled (0 °C) suspension of L-valine in methanol, $SOCl_2$ was added drop wise and the mixture was stirred at room temperature for 16 hours. The completion of reaction was monitored by ESI-MS. The reaction mixture after removal of the solvent was dried *in vacuo* to give a pale yellow solid. Thus, the solid obtained was washed with Et_2O to obtain the ester hydrochloride as a colorless solid.

Global Deprotection and Cleavage from the Resin

Peptides **1** to **16** were cleaved-off from the resin under mild condition with the cleavage cocktail- AcOH:TFE:DCM (3:1:6) for 3 hrs. The cleaved peptide product, having a free C-terminal, was then subjected to removal of excess DCM, acetic acid etc. and dried under high vacuum to obtain a white powder. This was then coupled to valine methyl ester in solution phase using 2.5 equiv each of HOBt, DCC and valine methyl ester in

DCM at room temperature for 10-12 hrs. The completion of reaction was monitored by ESI-MS. After the reaction was completed, the product mixture was concentrated in a rotary evaporator and the oily mass was dissolved in minimum volume of methanol (1-2 mL). Finally, the peptide solution was precipitated in chilled water, centrifuged twice and the white solid obtained was dissolved in 4-5 mL of methanol for purification by RP-HPLC.

Peptide **17** was cleaved off from the resin and globally deprotected by using the cleavage cocktail TFA:TIPS:Water (95:2.5:2.5) for 3 hours at room temperature. The cleaved peptide solution was then precipitated in chilled diethyl ether, centrifuged twice and dissolved in 30% acetonitrile for purification by RP-HPLC.

Peptide **18** was cleaved off from the resin and globally deprotected by using the cleavage cocktail TFA:DCM:TIPS:Water (47.5:47.5:2.5:2.5) for 5 hours at room temperature. The cleaved peptide solution was then precipitated in chilled diethyl ether, centrifuged twice and dissolved in water (1-2 mL) for purification by RP-HPLC.

While peptides **19-21** and **18a-21a** were cleaved off from the resin and globally deprotected by using the cleavage cocktail TFA:TIPS:Water (95:2.5:2.5) for 20 minutes at room temperature. The cleaved peptide solution was then precipitated in chilled diethyl ether, centrifuged twice and dissolved in 5% DMSO for purification by RP-HPLC.

Purification By RP-HPLC

A suitably adjusted gradient of 70% B to 100% B was used for purification of compounds **1** to **16**, where solvent A was 0.1% TFA in H_2O and B was 0.1% TFA in methanol. Compounds **17-21** were purified using a gradient of 10% B to 50% B, where solvent A was 0.1% TFA in H_2O and B was 0.1% TFA in acetonitrile. Compounds **18a-21a** were purified using a gradient of 15% B to 50% B, where solvent A was 0.1% TFA in H_2O and B was 0.1% TFA in acetonitrile.

Circular Dichroism Spectroscopy

CD spectra for compounds **1** to **21** were recorded at 125 μM concentration over a wavelength range of 190-260 nm with a scan rate of 100 nm per minute and data pitch of 0.5 nm.

NMR Acquisition

For compounds **1** to **16,** the samples were dissolved in $CDCl_3$ and TMS (δ = 0 ppm) was used as an internal standard in $CDCl_3$.For compounds **17** to **21** (including peptides **18a** to **21a**) were dissolved in deuterated acetate buffer (pH 3.8):D_2O **(**9:1). Here, DSS was used as an internal standard (δ = 0 ppm). Standard Varian pulse sequences including presat, wgtocsy and wgroesy were used to acquire the NMR data. Water suppression was performed using water-gate solvent suppression as implemented in the Varian Biopack Suit.

The NMR of all compounds **1** to **21** (including peptides **18a** to **21a**) were obtained using concentration of 1-3 mM. ^{1}H NMR spectra at three different dilutions for compounds **1** to **21** were performed to check for aggregation at 25°C in their respective NMR buffer.

The highest concentration of each compound was used to obtain two-dimensional NMR spectra.

Two-dimensional data were obtained using 2048 data points in the direct dimension and 512 data points in the indirect dimension. TOCSY and ROESY spectra were acquired with a mixing time of 60 milliseconds and 200 milliseconds respectively.

All NMR data were processed using iNMR (www.inmr.net), and the 2D NMR data were analyzed with SPARKY[5] The chemical shift tables were generated from TOCSY, COSY and ^{1}H spectra. The sequential assignments and inter- and intra-residue NOEs were determined through ROESY. The NOEs were then integrated and the integration values were converted to distances using the formula $V=Kd^{-6}$, where V is the integrated peak volume, K is a constant (determined using resolved diastereotopic CH_2 groups from Phe2 or some cases Gly), and d is the distance between the protons.

DMSO-d_6 titration was performed by addition of 200µl of DMSO-d_6 in 500µl of $CDCl_3$ in 25µl increment. The resulting concentration ranges from ~5% to 29% v/v. Proton spectrum was acquired at each step, and shifts were determined to generate the DMSO-d_6 titration graphs.

Structure Calculation

To calculate the structure of the molecule we have used charmM force field,[6] via the interface of Discovery Studio, for the entire process.

The distance restraints were converted into a charm restraint file using a custom Perl script. The resulting file was then used to define NOE restraints inside the charmM syntax. To the distance, 10% were added or subtracted to define the upper and lower limits respectively. If there were any methyl protons involved in the restraints, an additional 0.4Å per methyl group (pseudoatom correction) were added to the upper limit to compensate for the errors involved.[7]

The initial structure was obtained by following a simulated annealing protocol. It was then refined by dihedral angle constraints derived from ^{1}H NMR spectra employing Bystrov equation[8] followed by a 10 ns restrained molecular dynamics run. The average over the dynamics run was considered to be the final structure and 10 structures were sampled at equal time intervals to generate the ensemble.

Compound 1:

(A)

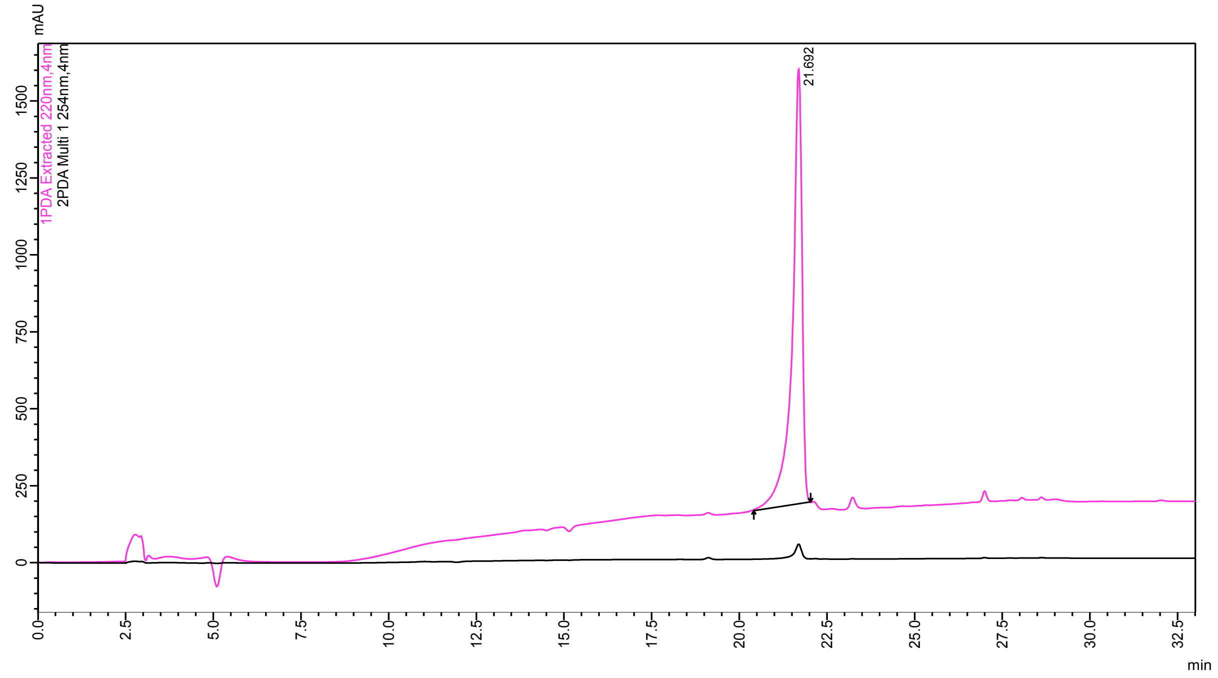

(B)

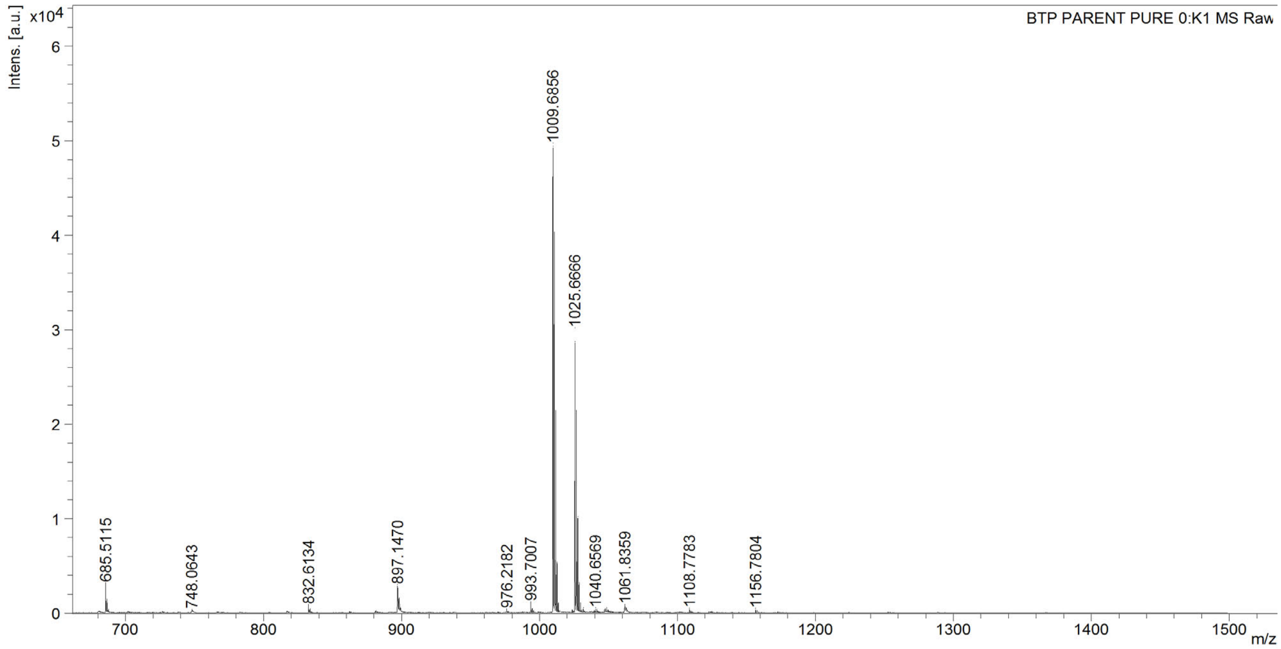

Figure S1.1: (A) Analytical HPLC chromatogram of purified compound **1** at 70-100% MeOH/H_2O gradient and (B) the respective MALDI profile of the pure compound. Calculated MW: 1009.5841 [M+Na]; Observed MW: 1009.6856.

(A)

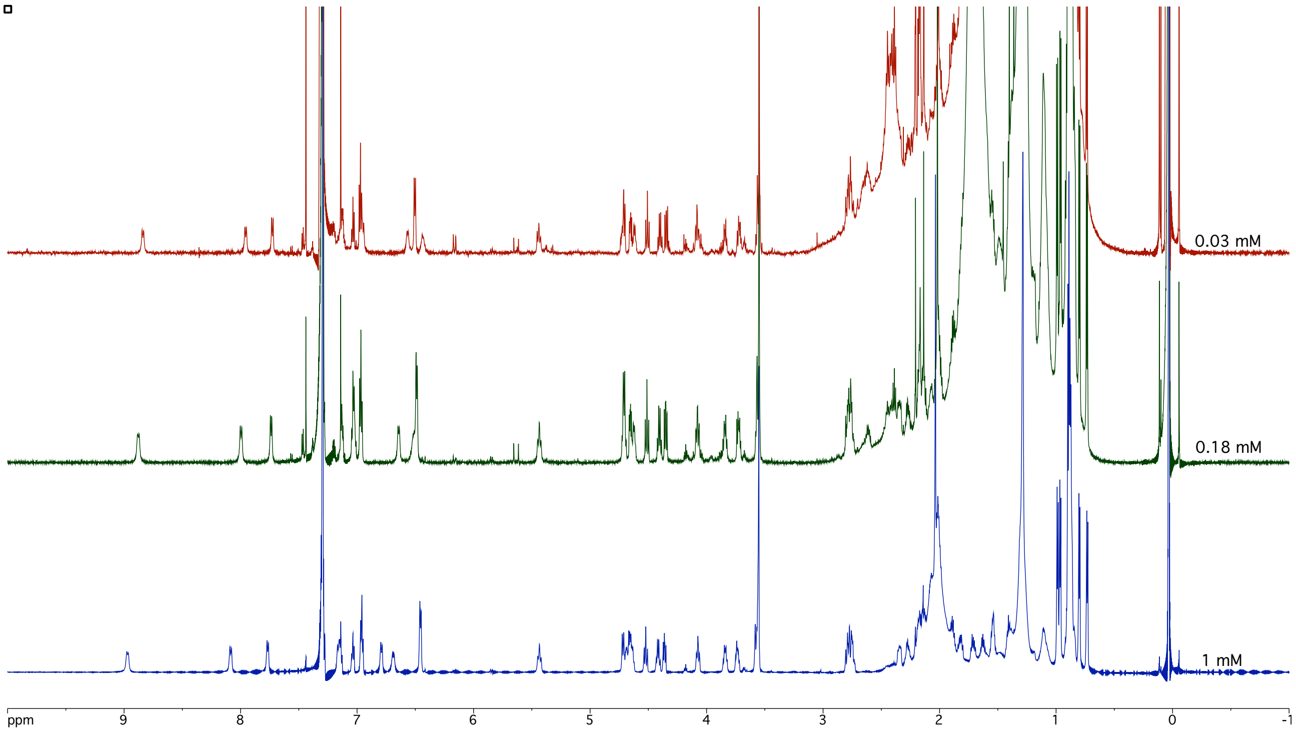

Figure S1.2: ^{1}H NMR spectra of Compound **1** at three different dilutions in $CDCl_3$ at 25°C.

Table 1.1: Chemical shifts table.

Residue	Atoms									
	HN	HA	HB		HG		HD		OMe	NAc
			1	2	1	2	1	2		
**LEU1**	7.07	4.60	1.52	1.39			0.87			1.99
**PHE2**	6.61	5.4	3.53	2.77						
**VAL3**	8.89	4.49	2.11		0.94	0.87				
**D-PRO4**		4.63	2.15	2	2.25		3.81	3.71		
**PRO5**		4.68	2.12	2.31	1.98		4.05	3.54		
**LEU6**	7.73	4.39	1.78	1.7	1.61		0.96	0.87		
**PHE7**	6.75	4.67	2.74							
**VAL8**	8.01	4.32	1.86		0.77	0.71			3.52	

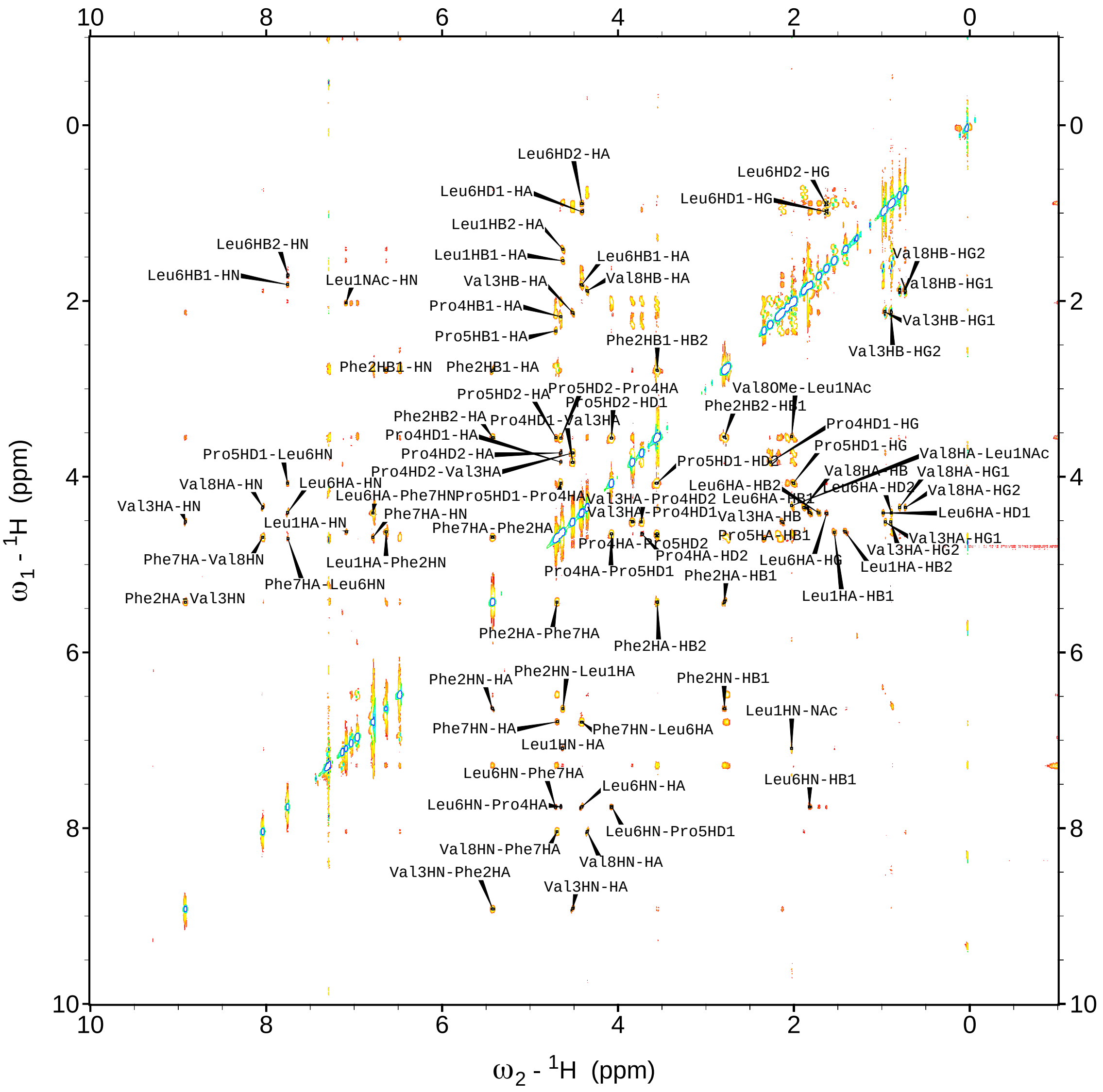

Figure S1.3: ROESY spectrum with assigned peaks.

Table1.2: List of ROEs with respective NMR distances and violations.

Interactions	NMR Distance	Lower Limit	Upper Limit	Observed Distance	Violations
Leu1HA-HN	3.46	3.11	3.81	2.88	-0.2
Leu1HA-Phe2HN	2.66	2.39	2.93	2.33	-0.1
Leu1HB1-HA	2.80	2.52	3.08	2.65	0
Leu1HB2-HA	2.86	2.57	3.15	2.95	0
Leu1NAc-HN	3.09	2.78	3.80	2.78	0
Phe2HA-Val3HN	2.68	2.41	2.95	2.12	-0.3
Phe2HB1-HA	2.98	2.68	3.28	3.07	0
Phe2HB1-HB2	1.87	1.68	2.06	1.74	0
Phe2HB1-HN	3.16	2.84	3.48	2.90	0
Phe2HB2-HA	2.59	2.33	2.85	2.47	0

**Phe2HN-HA**	3.47	3.12	3.82	2.96	-0.2
**Val3HA-HG1**	2.92	2.63	3.21	3.00	0
**Val3HA-HG2**	2.93	2.64	3.22	3.32	0.1
**Val3HA-HN**	3.45	3.11	3.80	2.96	-0.1
**Val3HB-HA**	3.05	2.75	3.36	2.92	0
**Val3HB-HG1**	2.74	2.47	3.01	2.48	0
**Val3HB-HG2**	2.97	2.67	3.27	2.48	-0.2
**pro4HB1-HA**	2.50	2.25	2.75	2.38	0
**pro4HD1-Val3HA**	2.34	2.11	2.57	2.27	0
**pro4HD2-Val3HA**	2.28	2.05	2.51	2.52	0
**Pro5HB1-HA**	2.92	2.63	3.21	2.78	0
**Pro5HD1-pro4HA**	2.13	1.92	2.34	2.31	0
**Pro5HD1-Leu6HN**	3.24	2.92	3.56	3.92	0.4
**Pro5HD2-pro4HA**	2.31	2.08	2.54	2.30	0
**Pro5HD2-HA**	3.30	2.97	3.63	3.75	0.1
**Pro5HD2-HD1**	1.81	1.63	1.99	1.79	0
**Leu6HA-HB2**	2.92	2.63	3.21	2.97	0
**Leu6HA-HG**	3.31	2.98	3.64	3.59	0
**Leu6HA-HN**	3.24	2.92	3.56	2.90	0
**Leu6HA-Phe7HN**	2.27	2.04	2.50	2.22	0
**Leu6HB1-HA**	2.80	2.52	3.08	2.64	0
**Leu6HD1-HA**	3.24	2.92	3.96	4.06	0.1
**Leu6HD1-HG**	2.73	2.46	3.40	2.46	0
**Leu6HD2-HA**	3.03	2.73	3.73	2.97	0
**Leu6HD2-HG**	2.47	2.22	3.12	2.47	0
**Phe7HA-Phe2HA**	2.42	2.18	2.66	2.82	0.2
**Pro5HA-Leu6HN**	3.43	3.09	3.77	2.92	-0.2
**Phe7HA-HN**	3.02	2.72	3.32	2.88	0
**Phe7HA-Val8HN**	2.48	2.23	2.73	2.59	0
**Val8HA-HB**	2.93	2.64	3.22	2.70	0
**Val8HA-HG1**	3.15	2.84	3.47	2.99	0
**Val8HA-HG2**	3.36	3.02	3.70	3.68	0
**Val8HA-HN**	3.17	2.85	3.49	2.84	0
**Val8OMe-Leu1NAc**	3.14	2.83	4.25	3.94	0

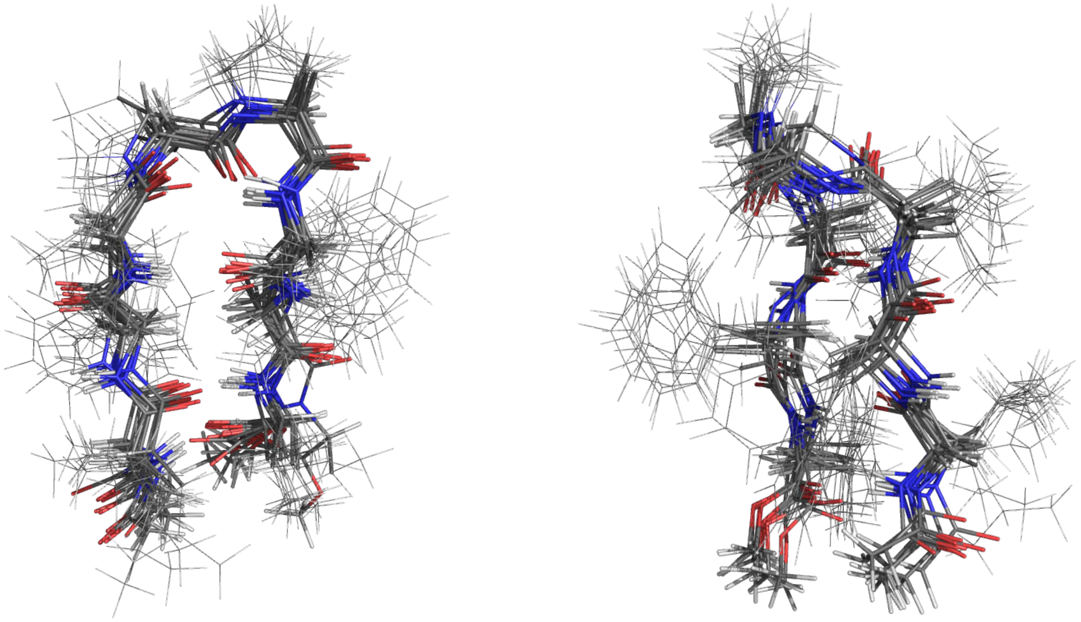

Figure S1.4: Overlay of 10 representative conformations generated using Molecular Dynamics simulation, showing both front view (left panel) and side view (right panel).

(A)

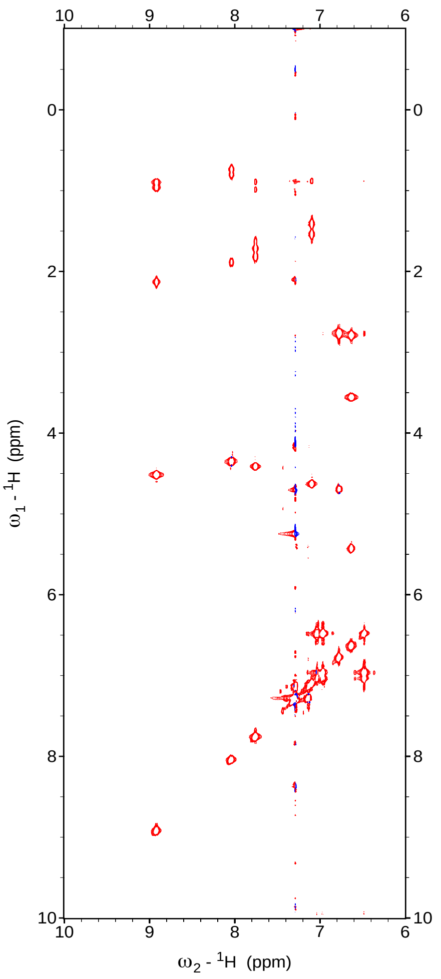

CDCl₃

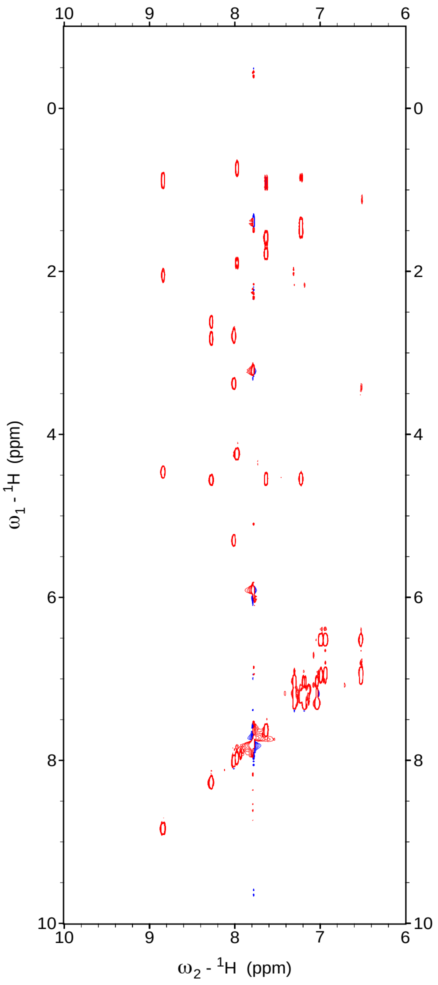

DMSO-d_6 – $CDCl_3$
(1:2)

(B)

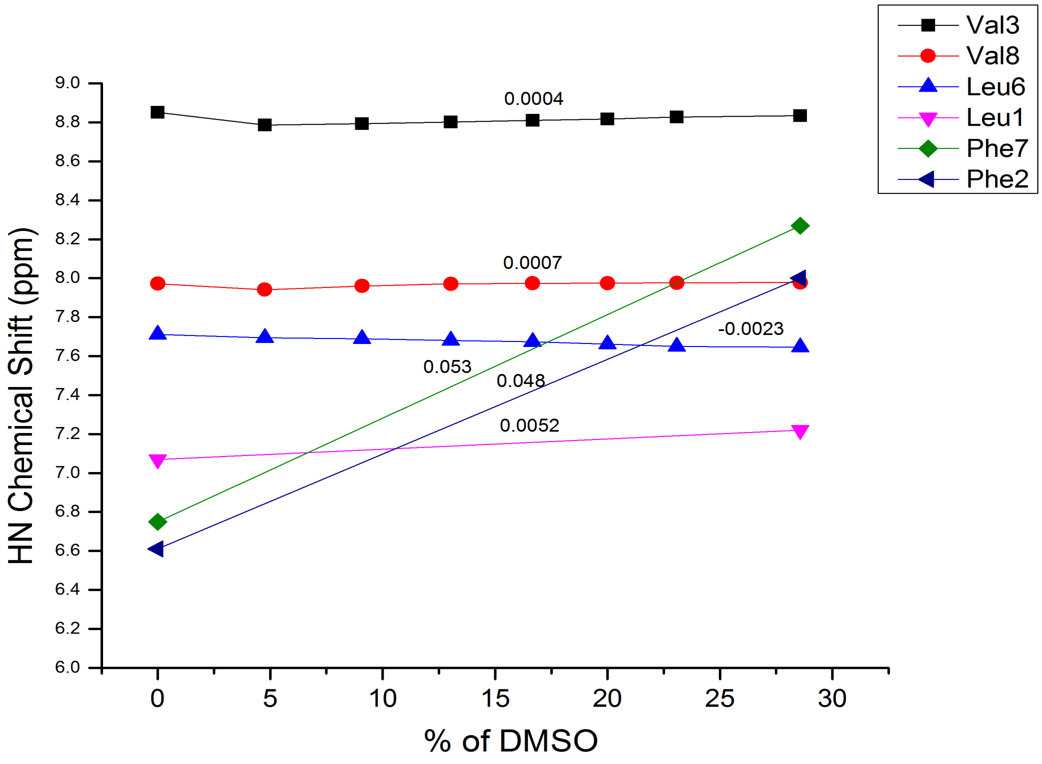

Figure S1.5: (A) TOCSY spectra in $CDCl_3$ and DMSO-d_6 – $CDCl_3$ (1:2) and (B) DMSO-d_6 titration curve indicating the solvent exposed (F2, F7) and solvent shielded (L1, V3, L6, V8) amide protons. The value indicates the slope generated by the linear fit of the data points.

Compound 2:

(A)

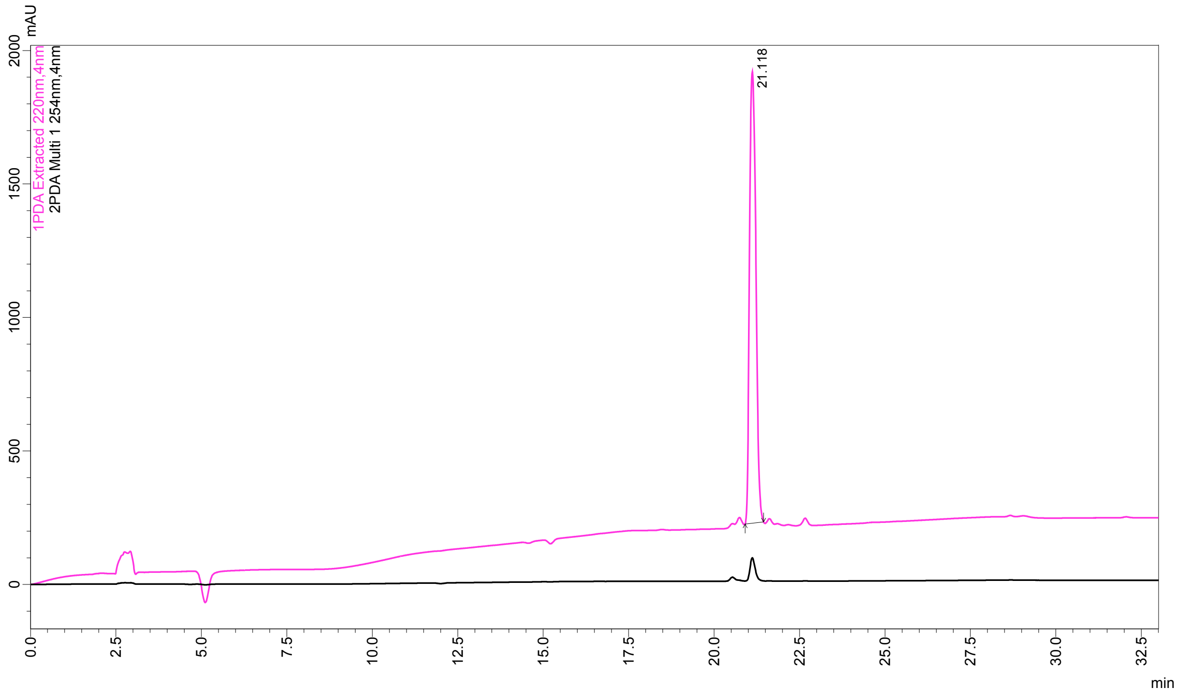

(B)

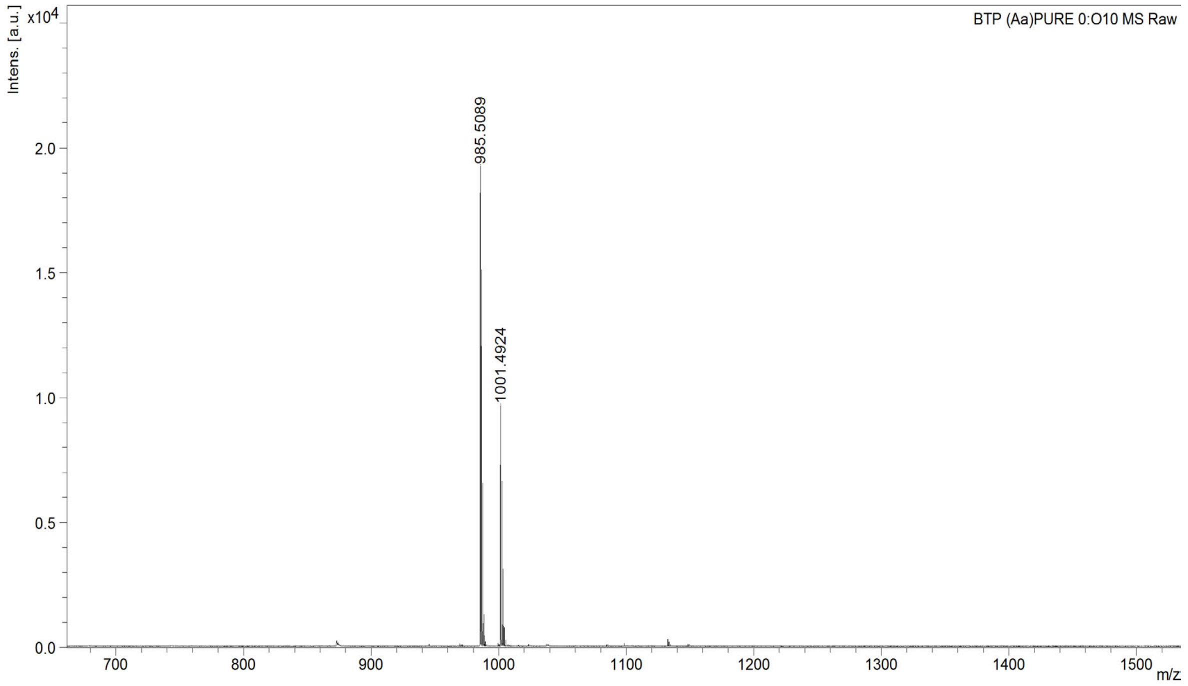

Figure S2.1: A) Analytical HPLC chromatogram of purified compound **2** at 70-100% MeOH/H_2O gradient and (B) the respective MALDI profile of the pure compound. Calculated MW: 985.5841 [M+Na]; Observed MW: 985.5089.

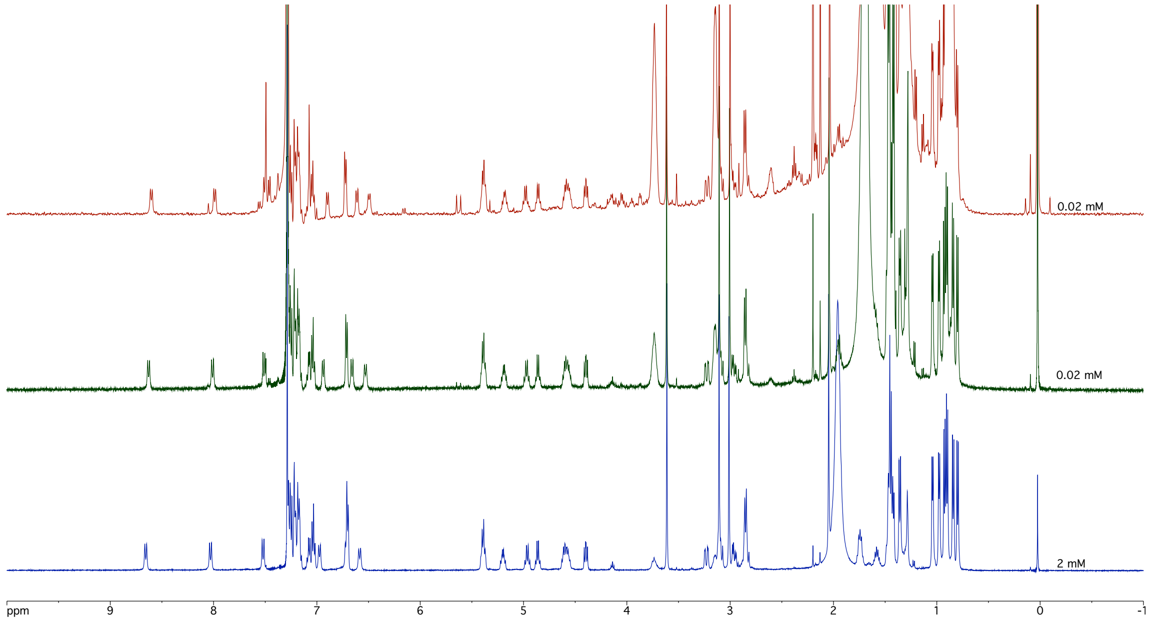

Figure S2.2: ^{1}H NMR spectra of Compound **2** at three different dilutions in $CDCl_3$ at 25°C.

Table 2.1: Chemical shifts table.

Residue	Atoms									
	HN	NMe	HA	HB		HG		HD	OMe	NAc
				1	2	1	2			
**LEU1**	7.12		4.6	1.5	1.37			0.85		2.00
**PHE2**	6.67		5.38	2.8	3.28					
**VAL3**	8.69		4.69	2.11		0.88				
**D-ALA4**		3.27	4.99	1.45						
**ALA5**		3.1	5.38	1.43						
**LEU6**	7.49		4.52	1.78	1.66			0.93		
**PHE7**	6.76		4.75	2.74						
**VAL8**	8.06		4.36	1.88		0.77	0.72		3.54	

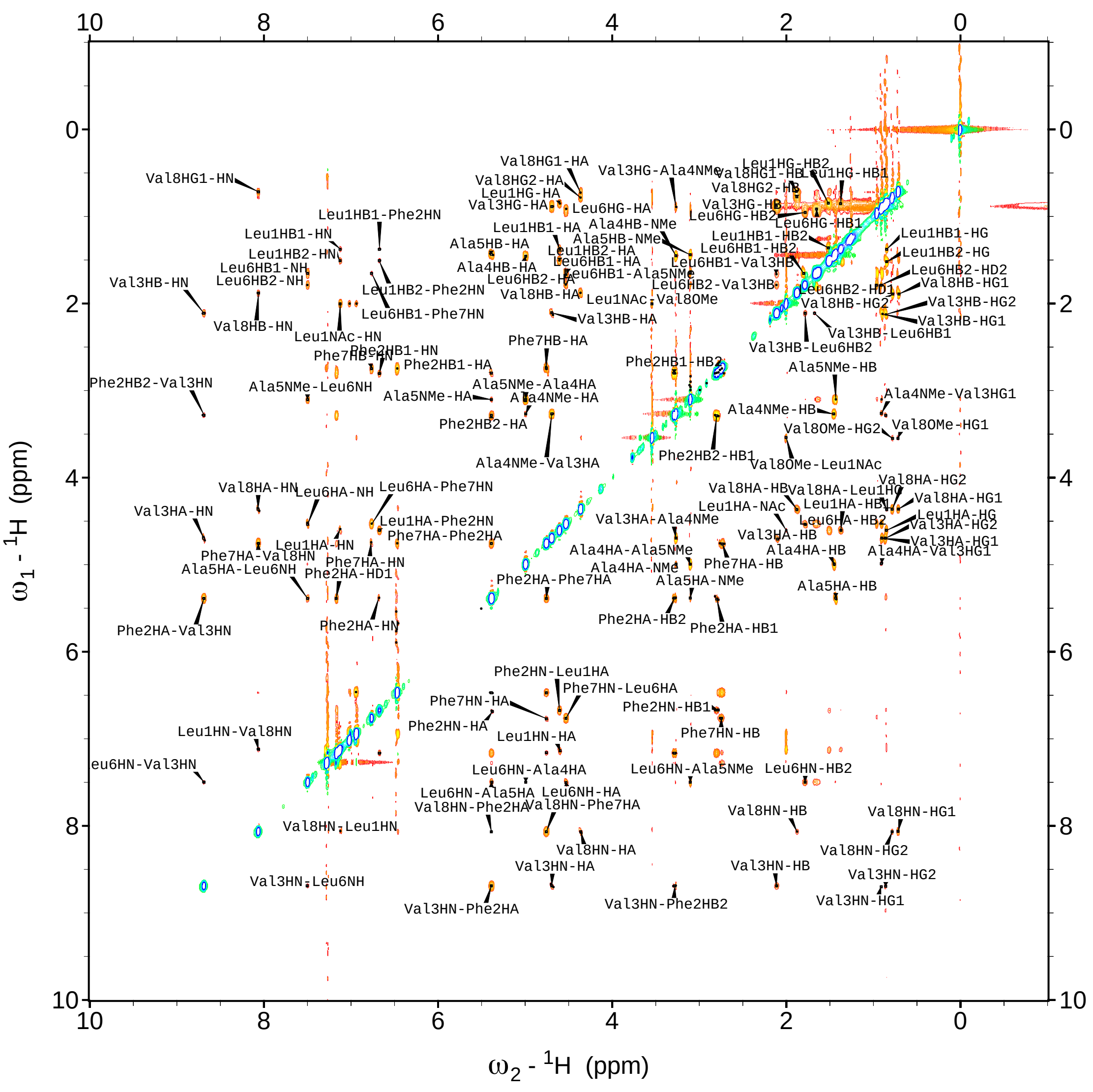

Figure S2.3: ROESY spectra with assigned peaks.

Table2.2: List of ROEs with respective NMR distances and violations.

Interactions	NMR Distance	Upper Limit	Lower Limit	Observed Distance	Violations
**Leu1HA-HN**	2.77	3.05	2.49	2.86	0
**Leu1HA-NAc**	3.63	3.99	3.27	4.41	0
**Leu1HA-Phe2HN**	2.52	2.77	2.27	2.47	0
**Leu1HB1-HA**	2.64	2.90	2.37	2.73	0
**Leu1HB1-HN**	2.98	3.27	2.68	3.08	0
**Leu1HB1-Phe2HN**	3.14	3.45	2.82	3.75	0.3
**Leu1HB2-HA**	2.47	2.72	2.22	2.91	0.2
**Leu1HB2-HN**	2.91	3.20	2.62	3.24	0
**Leu1HG-HA**	2.53	2.78	2.27	2.93	0.1
**Leu1HG-HB1**	2.34	2.58	2.11	2.81	0.2

**Leu1HN-Val8HN**	2.38	2.62	2.14	2.60	0
**Phe2HA-HN**	3.19	3.51	2.87	2.96	0
**Phe2HA-Val3HN**	2.24	2.46	2.01	2.15	0
**Phe2HB1-HA**	2.76	3.03	2.48	2.78	0
**Phe2HB1-HB2**	1.81	1.99	1.63	1.73	0
**Phe2HB1-HN**	2.81	3.09	2.53	3.34	0.2
**Phe2HB2-HA**	2.44	2.69	2.20	2.42	0
**Phe2HB2-Val3HN**	3.13	3.44	2.82	3.58	0.1
**Val3HA-HN**	2.93	3.23	2.64	2.94	0
**Val3HB-HA**	2.59	2.85	2.33	3.05	0.2
**Val3HB-HG1**	2.17	2.39	1.96	2.49	0
**Val3HB-HG2**	2.24	2.46	2.02	2.49	0
**Val3HB-HN**	2.80	3.08	2.52	2.69	0
**Val3HN-HG1**	3.58	3.94	3.22	4.56	0.2
**Val3HN-HG2**	3.13	3.44	2.81	3.83	0
**Val3HN-Leu6HN**	2.58	2.84	2.32	3.10	0.3
***ala4HA-Val3HG1**	2.81	3.09	2.53	4.01	0.5
**ala4HB-HA**	2.16	2.37	1.94	2.49	0
**ala4HB-NMe**	2.13	2.34	1.92	3.29	0.1
**Val3HA-ala4NMe**	2.08	2.29	1.87	2.59	0
**ala4NMe-Val3HG1**	2.61	2.88	2.35	4.01	0.3
**ala4NMe-HA**	2.99	3.29	2.69	3.43	0
**Ala5HA-Leu6HN**	2.72	2.99	2.44	2.94	0
**Ala5HB-HA**	2.16	2.38	1.95	2.48	0
**Ala5HB-NMe**	2.13	2.34	1.91	3.43	0.3
**Ala5NMe-ala4HA**	2.06	2.26	1.85	2.57	0
**Ala5NMe-HA**	3.14	3.46	2.83	3.79	0
**Ala5NMe-Leu6HN**	2.48	2.72	2.23	3.24	0.1
**Leu6HA-HN**	2.67	2.94	2.40	2.92	0
**Leu6HA-Phe7HN**	2.28	2.51	2.05	2.38	0
**Val3HB-Leu6HB1**	2.74	3.01	2.46	3.23	0.2
**Leu6HB1-Ala5NMe**	2.82	3.10	2.53	3.48	0
**Leu6HB1-Phe7HN**	2.98	3.28	2.68	3.76	0.5
**Val3HB-Leu6HB2**	2.77	3.05	2.49	2.98	0
**Leu6HA-HB2**	2.63	2.89	2.37	2.92	0
**Leu6HG-HA**	2.33	2.56	2.09	2.60	0
**Leu6HG-HB1**	1.82	2.01	1.64	2.41	0.4
***Leu6HG-HB2**	2.12	2.33	1.90	2.97	0.6
**Leu6HN-ala4HA**	3.25	3.57	2.92	3.68	0.1
**Leu6HN-HB2**	2.68	2.95	2.42	3.28	0.3
**Phe7HA-Phe2HA**	2.41	2.65	2.17	2.29	0
**Phe7HN-HA**	2.90	3.19	2.61	2.92	0
**Val8HN-Phe7HA**	2.21	2.43	1.99	2.32	0

**Phe7HB-HN**	2.51	2.77	2.26	3.17	0.4
**Val8HA-HN**	2.74	3.01	2.47	2.90	0
**Val8HB-HA**	2.44	2.68	2.20	2.42	0
**Val8HB-HN**	3.02	3.32	2.72	3.27	0
***Val8HA-HG1**	2.64	2.91	2.38	3.83	0.5
**Val8HB-HG1**	2.30	2.53	2.07	2.48	0
**Val8HG1-HN**	2.80	3.08	2.52	3.05	0
**Val8HA-HG2**	2.51	2.76	2.26	3.06	0
**Val8HB-HG2**	2.29	2.52	2.06	2.47	0
**Val8HN-Phe2HA**	3.49	3.83	3.14	3.72	0
***Val8HN-HG2**	3.18	3.50	2.86	4.49	0.6
**Val8OMe-HG1**	2.48	2.72	2.23	3.92	0.4
**Val8OMe-HG2**	2.95	3.24	2.65	4.28	0.2
**Val8OMe-Leu1NAc**	2.70	2.97	2.43	3.78	0

* violations ≥0.5. The observed high violations can be explained by the local flexibility about the γ and δ methyl groups (Val and Leu respectively) and the terminal ester bond, peak overlap, additional J-mediated transfer and inaccuracies in the force fields.[9]

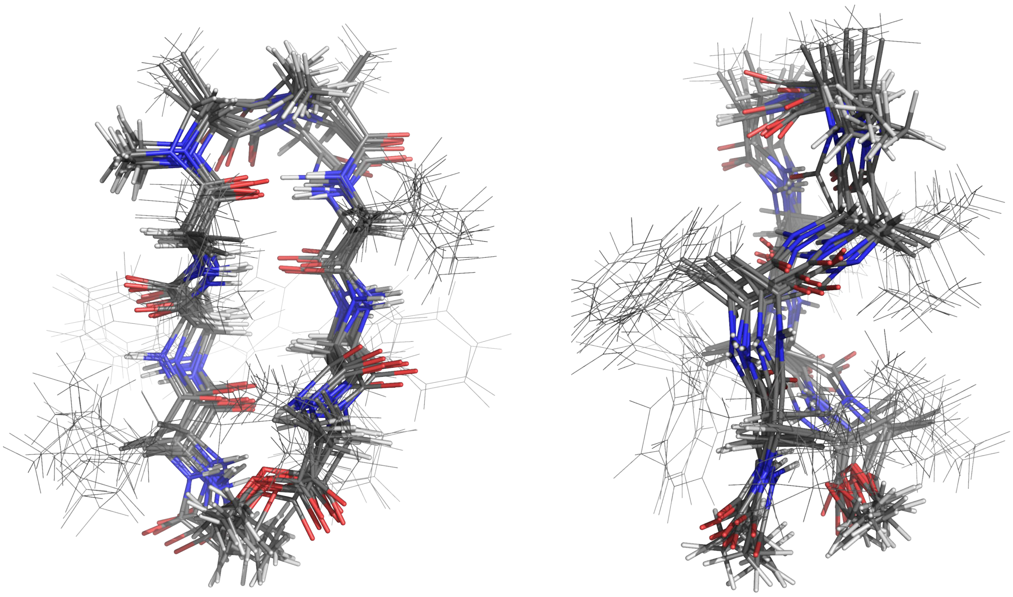

Figure S2.4: Overlay of 10 representative conformations generated using Molecular Dynamics simulation, showing both front view (left panel) and side view (right panel).

(A)

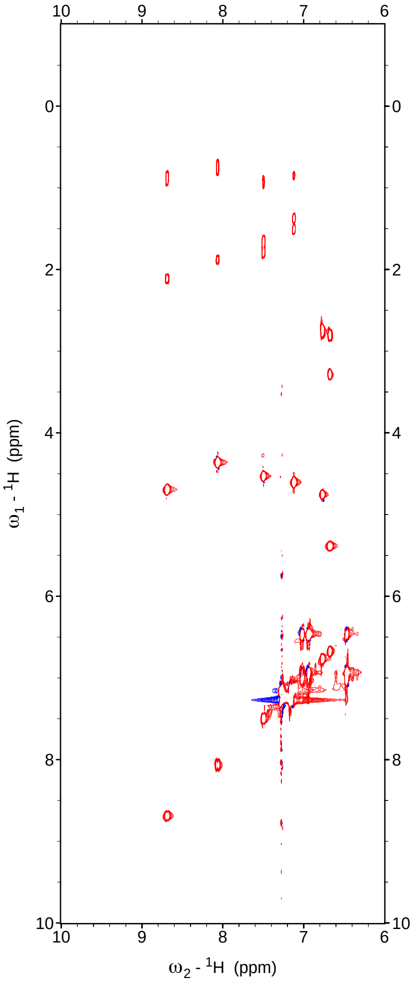

CDCl3

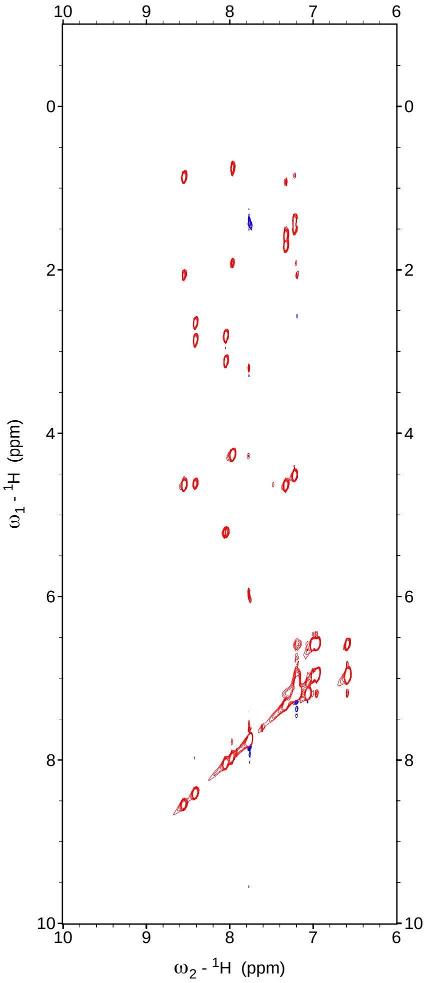

DMSO-d_6 – $CDCl_3$
(1:2)

(B)

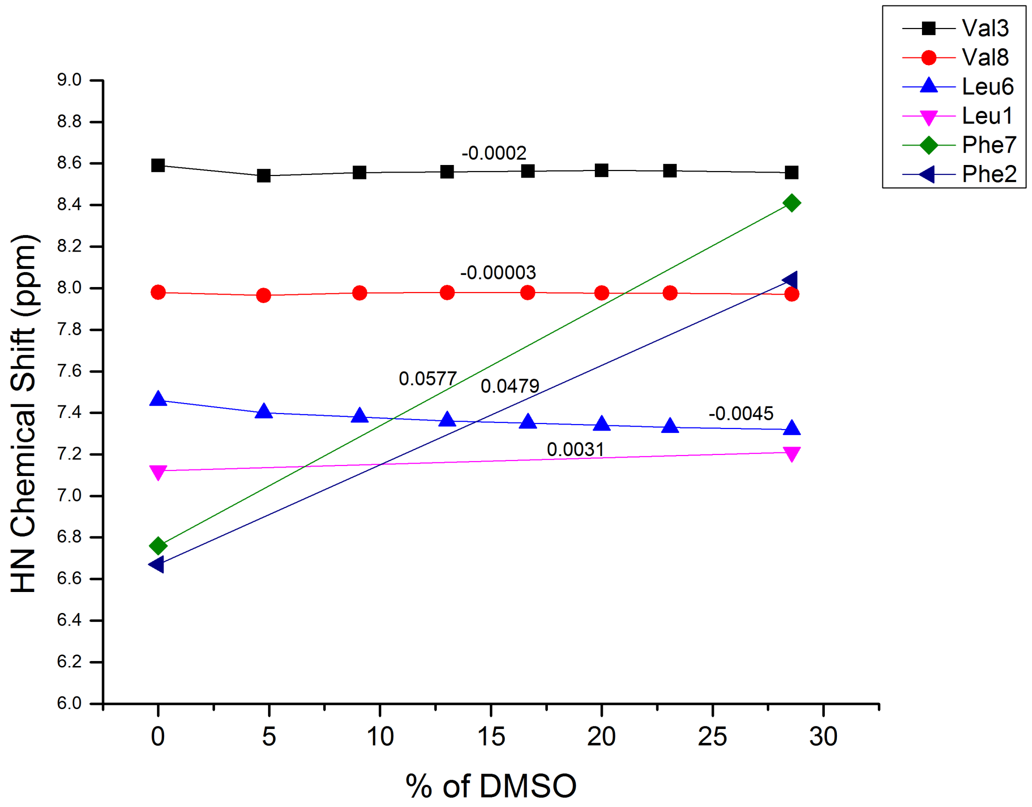

Figure S2.5: (A) TOCSY spectra in $CDCl_3$ and DMSO-d_6 – $CDCl_3$ (1:2) and (B) DMSO-d_6 titration curve indicating the solvent exposed (F2, F7) and solvent shielded (L1, V3, L6, V8) amide protons. The value indicates the slope generated by the linear fit of the data points.

Compound 2a:

Sequence: Ac-L-F-V-A’-A’-L-F-V-OMe

(A)

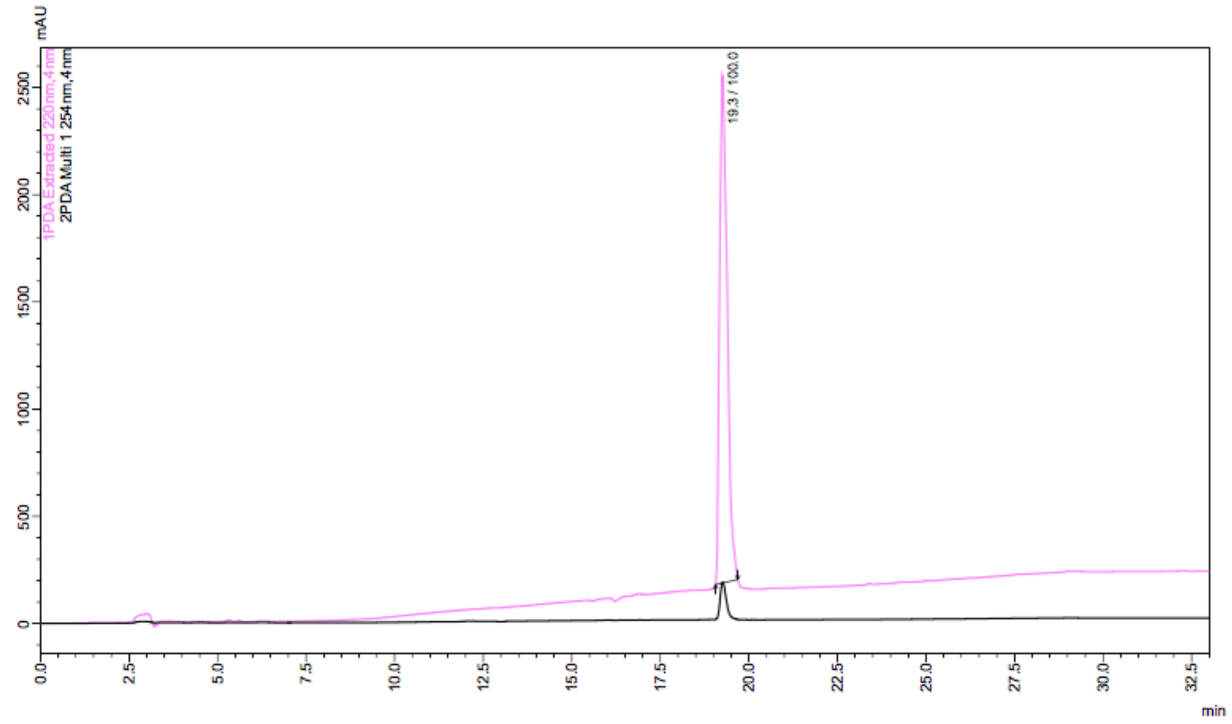

(B)

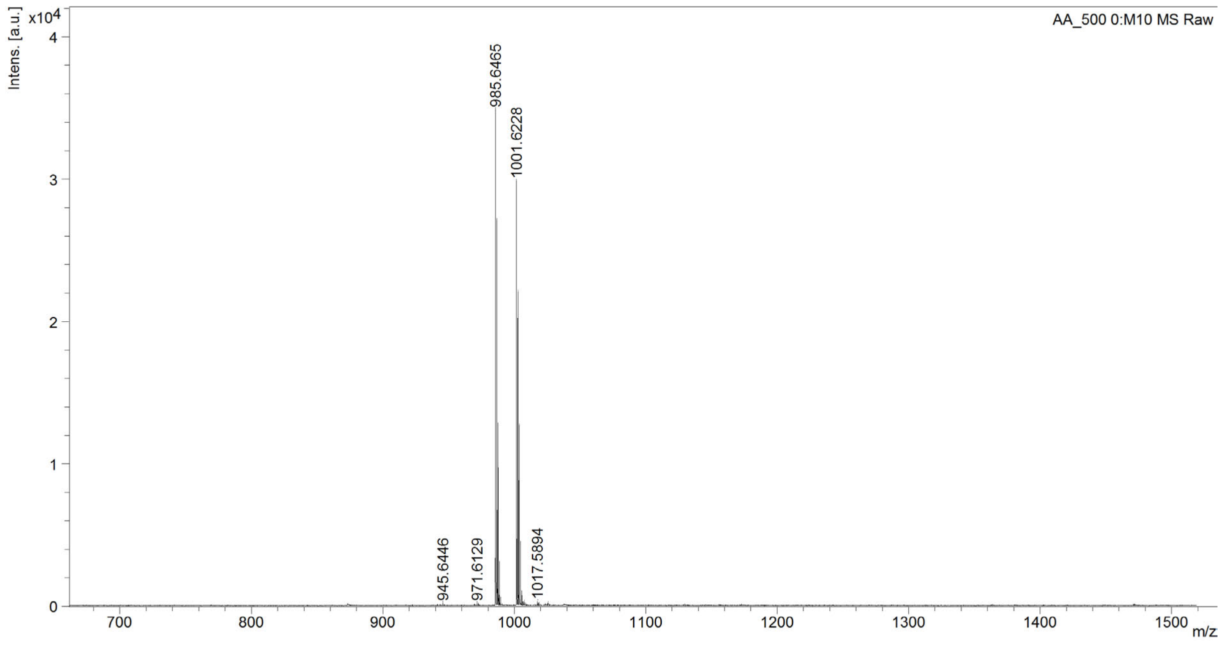

Figure S2a.1: A) Analytical HPLC chromatogram of purified compound **2a** at 70-100% MeOH/H_2O gradient and (B) the respective MALDI profile of the pure compound. Calculated MW: 985.5841 [M+Na]; Observed MW: 985.6465.

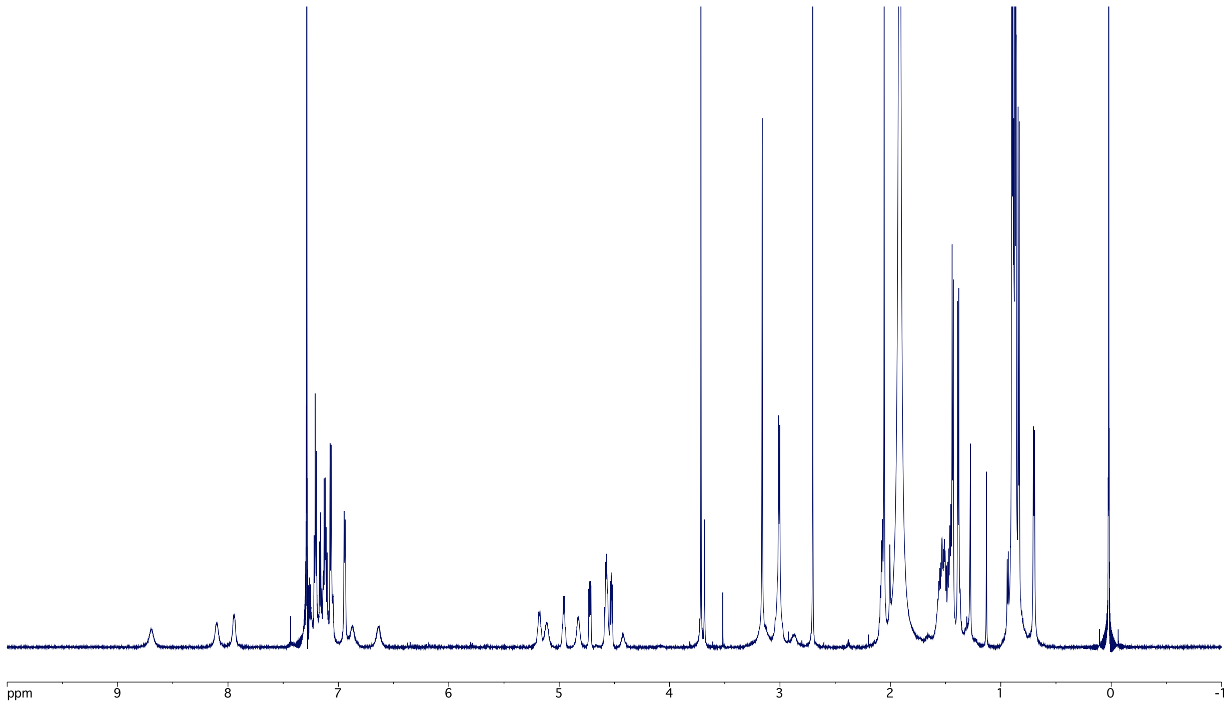

Figure S2a.2: ^{1}H NMR spectra of Compound **2a** in $CDCl_3$ at 25°C.

Table 2a.1: Chemical shifts table.

Residue	Atoms									
	**HN**	**NMe**	**HA**	**HB**		**HG**		**HD**	**OMe**	**NAc**
				1	2	1	2			
**LEU1**	7.05		4.58	1.48				0.88		2.06
**PHE2**	6.86		5.11	3.01						
**VAL3**	7.93		4.74	2.09		0.90	0.70			
**ALA4**		3.16	4.82	1.43						
**ALA5**		2.70	4.95	1.39						
**LEU6**	8.68		4.57	1.49				0.88		
**PHE7**	6.63		5.18	3.01						
**VAL8**	8.09		4.52	2.08		0.86			3.70	

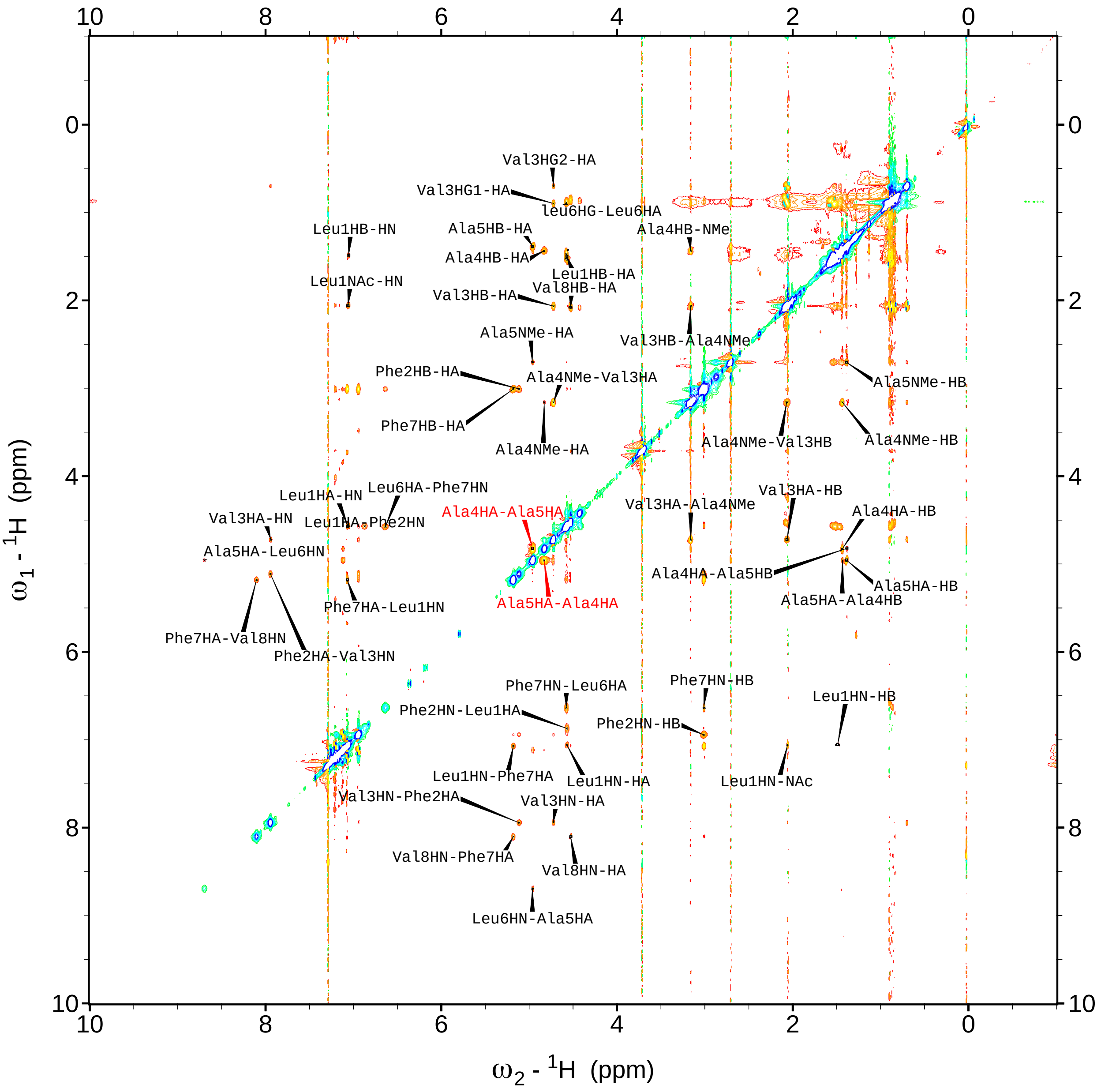

Figure S2a.3: ROESY spectra with assigned peaks.(Note: The assigned peaks marked in red indicates cis peptide bond)

Compound 3:

(A)

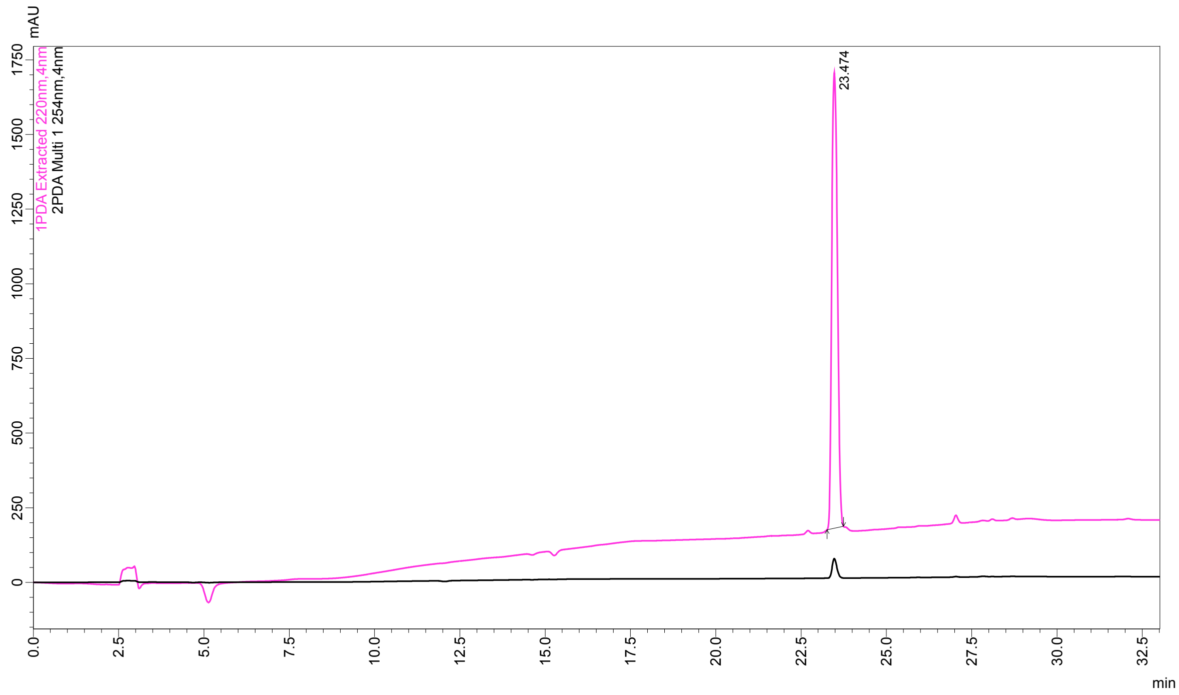

(B)

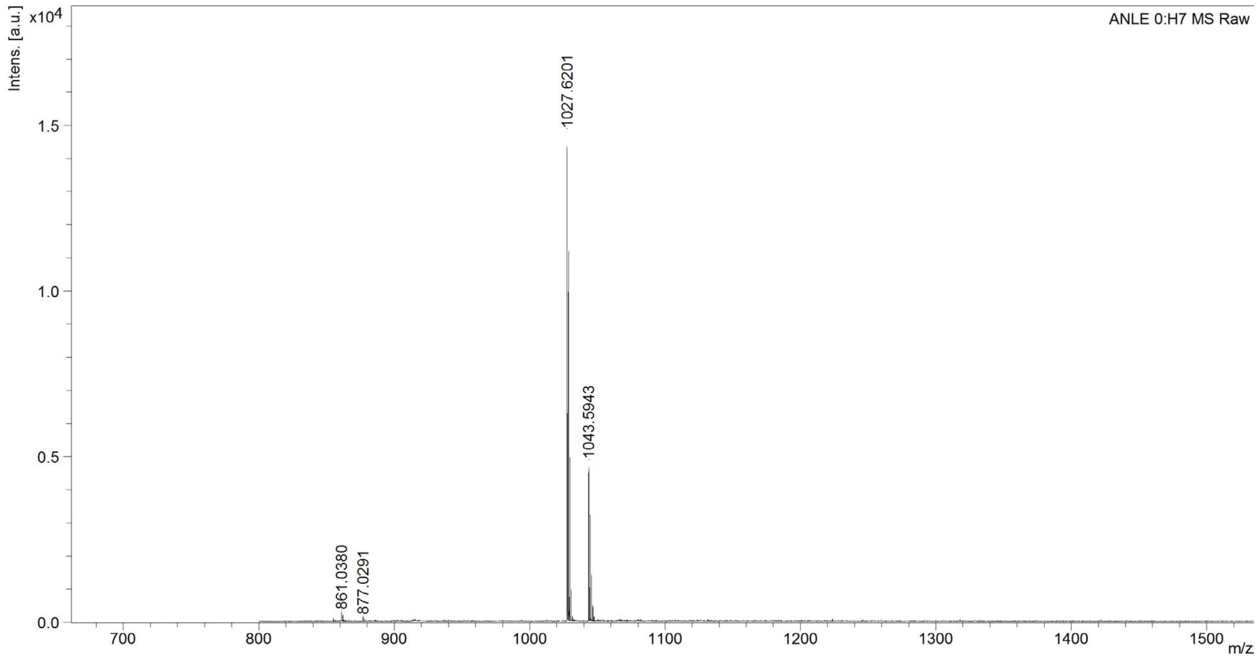

Figure S3.1: A) Analytical HPLC chromatogram of purified compound **3** at 70-100% MeOH/H_2O gradient and (B) the respective MALDI profile of the pure compound. Calculated MW: 1027.6310 [M+Na]; Observed MW: 1027.6201.

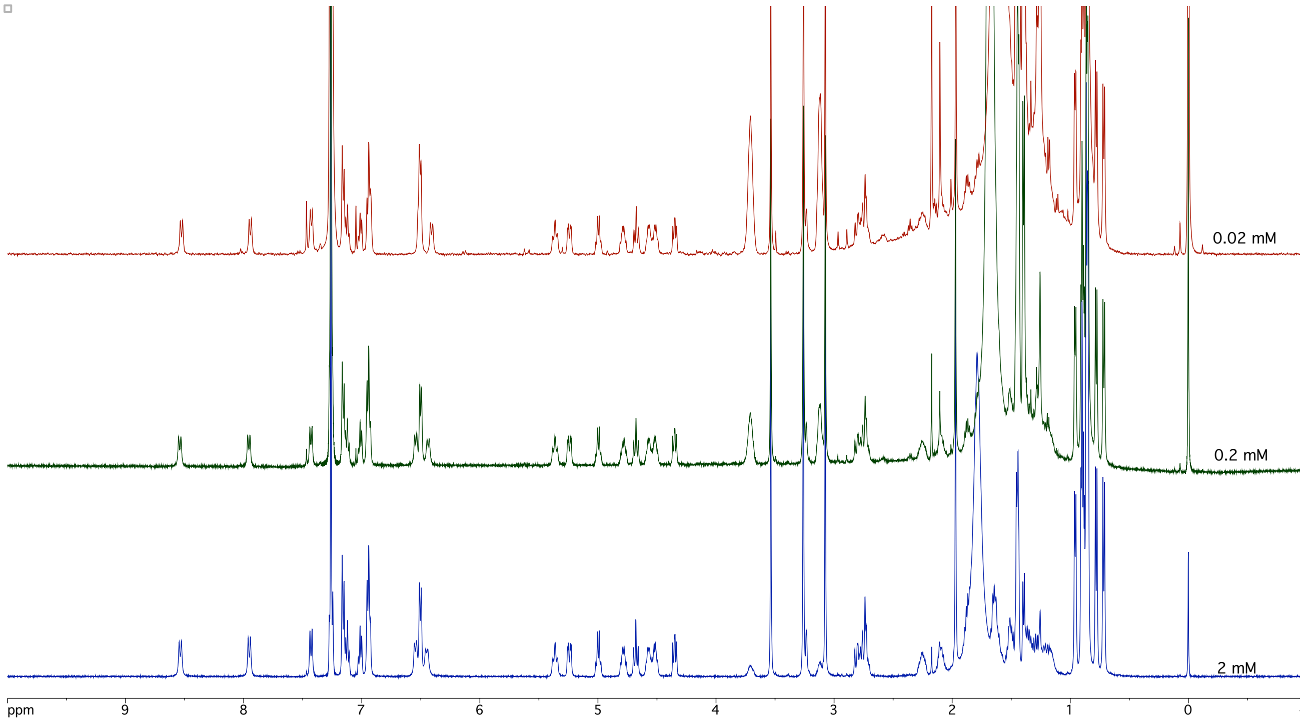

Figure S3.2: ^{1}H NMR spectra of Compound **3** at three different dilutions in $CDCl_3$ at 25°C.

Table 3.1: Chemical shifts table.

Residue	Atoms										
	HN	NMe	HA	HB		HG		HD		OMe	NAc
				1	2	1	2	1	2		
**LEU1**	7.05		4.58	1.51	1.38			0.86			1.98
**PHE2**	6.58		5.37	2.8	3.24						
**VAL3**	8.61		4.69	2.11		0.9	0.86				
**D-ALA4**		3.24	5	1.46							
**NLE5**		3.08	5.24	2.26	1.62						
**LEU6**	7.46		4.53	1.78	1.65			0.96	0.91		
**PHE7**	6.65		4.76	2.72							
**VAL8**	8.02		4.36	1.86		0.79	0.72			3.53	

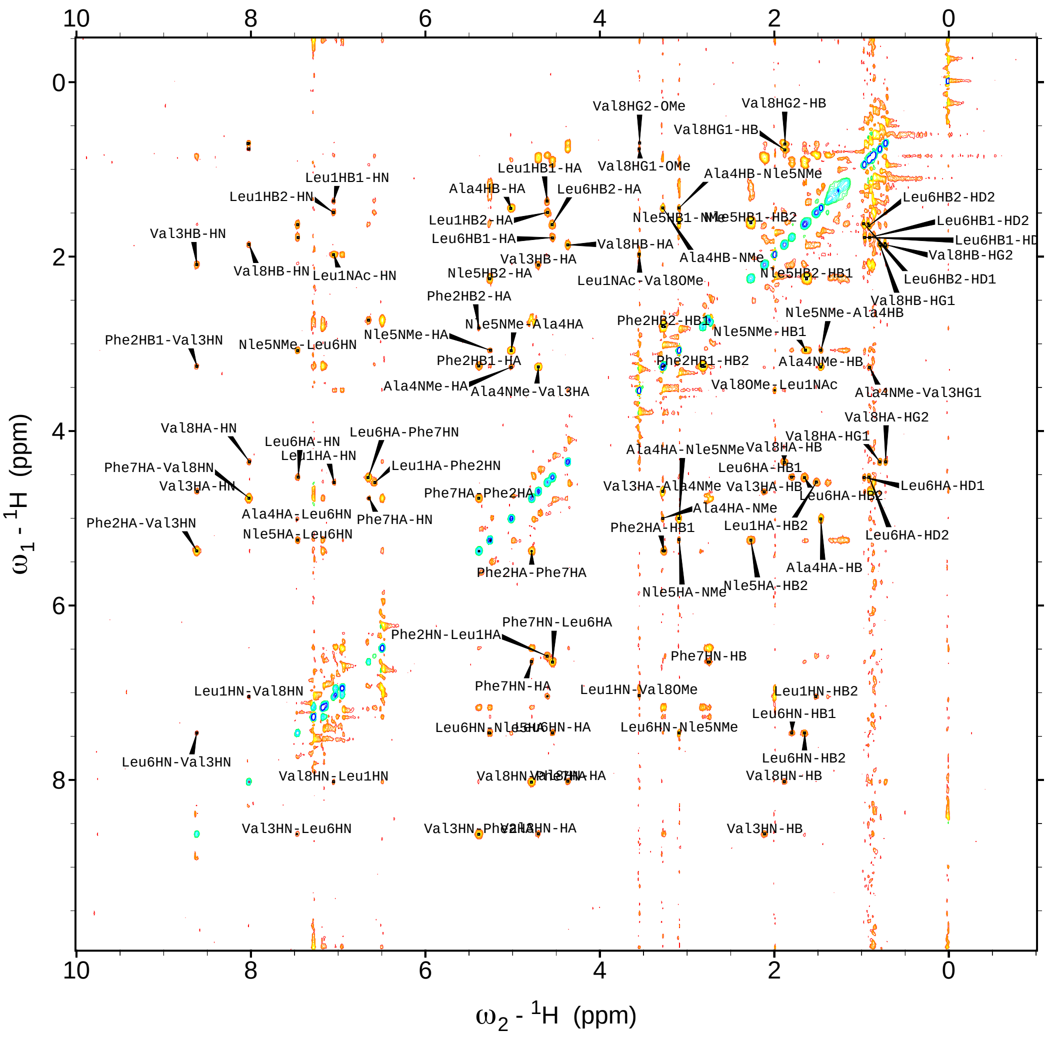

Figure S3.3: ROESY spectra with assigned peaks.

Table3.2: List of ROEs with respective NMR distances and violations.

Interactions	NMR Distance	Lower Limit	Upper Limit	Observed Distance	Violations
**Leu1HA-HN**	3.06	2.76	3.37	2.82	0
**Leu1HA-Phe2HN**	2.53	2.28	2.78	2.44	0
**Leu1HB1-HA**	2.66	2.39	2.92	2.58	0
**Leu1HB1-HN**	3.12	2.8	3.43	3.38	0
**Leu1HB2-HA**	2.5	2.25	2.75	3.05	0.3
**Leu1HB2-HN**	3.02	2.71	3.32	2.98	0
**Leu1HN-Val8HN**	3.26	2.93	3.58	2.95	0
**Leu1NAc-HN**	2.34	2.11	2.98	2.74	0
**Leu1NAc-Val8OMe**	2.72	2.45	3.79	3.84	0
**Phe2HA-Val3HN**	2.2	1.98	2.42	2.13	0
**Phe2HB1-HA**	2.42	2.18	2.67	2.59	0
**Phe2HB1-HB2**	1.8	1.62	1.98	1.73	0

**Phe2HB1-Val3HN**	2.87	2.58	3.16	3.16	0
**Val3HA-HN**	2.91	2.62	3.2	2.96	0
**Val3HB-HA**	2.7	2.43	2.97	3.04	0.1
**Val3HB-HN**	2.73	2.45	3	2.8	0
**ala4HB-HA**	2.17	1.95	2.79	2.46	0
**ala4HB-NMe**	2.14	1.92	3.15	3.29	0.1
**ala4HB-Nle5NMe**	2.73	2.45	3.8	4.03	0.2
**ala4NMe-Val3HA**	1.97	1.78	2.57	2.68	0.1
**ala4NMe-HA**	2.95	2.65	3.64	3.62	0
**Nle5HA-HB2**	2.32	2.09	2.55	2.41	0
**Nle5HA-Leu6HN**	2.58	2.32	2.84	2.88	0
**Nle5HB2-HA**	2.22	1.99	2.44	2.41	0
**Nle5HB1-HB2**	1.77	1.59	1.94	1.73	0
**Nle5HB1-NMe**	2.15	1.93	2.76	2.93	0.2
**Nle5NMe-ala4HA**	1.95	1.75	2.54	2.55	0
**Nle5NMe-HA**	2.89	2.6	3.58	3.76	0.2
**Nle5NMe-Leu6HN**	2.5	2.25	3.15	3.47	0.3
**Leu6HA-HD1**	2.81	2.53	3.5	3.23	0
**Leu6HA-HD2**	2.54	2.29	3.2	3.21	0
**Leu6HA-HN**	2.79	2.51	3.07	2.97	0
**Leu6HA-Phe7HN**	2.21	1.99	2.43	2.3	0
**Leu6HB1-HA**	2.66	2.39	2.92	2.96	0
***Leu6HB1-HD1**	2.54	2.29	3.19	3.7	0.5
**Leu6HB1-HD2**	2.71	2.44	3.38	3.17	0
**Leu6HN-HB1**	2.87	2.58	3.16	2.67	0
**Leu6HB2-HA**	2.12	1.9	2.33	2.75	0.4
**Leu6HN-Val3HN**	2.67	2.4	2.93	3.15	0.2
**Phe7HA-Phe2HA**	2.18	1.96	2.39	2.67	0.3
**Phe7HA-HN**	2.99	2.69	3.29	2.92	0
**Phe7HA-Val8HN**	2.15	1.93	2.36	2.28	0
**Phe7HN-HB**	2.63	2.37	3.29	3.45	0.2
**Val8HA-HG1**	2.6	2.34	3.26	3.01	0
**Val8HA-HG2**	2.72	2.45	3.39	3.26	0
**Val8HA-HN**	2.84	2.56	3.13	2.96	0
**Val8HB-HA**	2.41	2.17	2.66	3.05	0.4
**Val8HB-HN**	2.87	2.58	3.15	2.86	0
**Val8HB-HG1**	2.33	2.1	2.96	2.49	0
**Val8HG1-OMe**	3.2	2.88	4.32	4.28	0
**Val8HB-HG2**	2.35	2.11	2.98	2.43	0
***Val8HG2-OMe**	3.53	3.18	4.69	5.47	0.8

* violations ≥0.5. The observed high violations can be explained by the local flexibility about the γ and δ methyl groups (Val and Leu respectively) and the terminal ester bond, peak overlap, additional J-mediated transfer and inaccuracies in the force fields.[9]

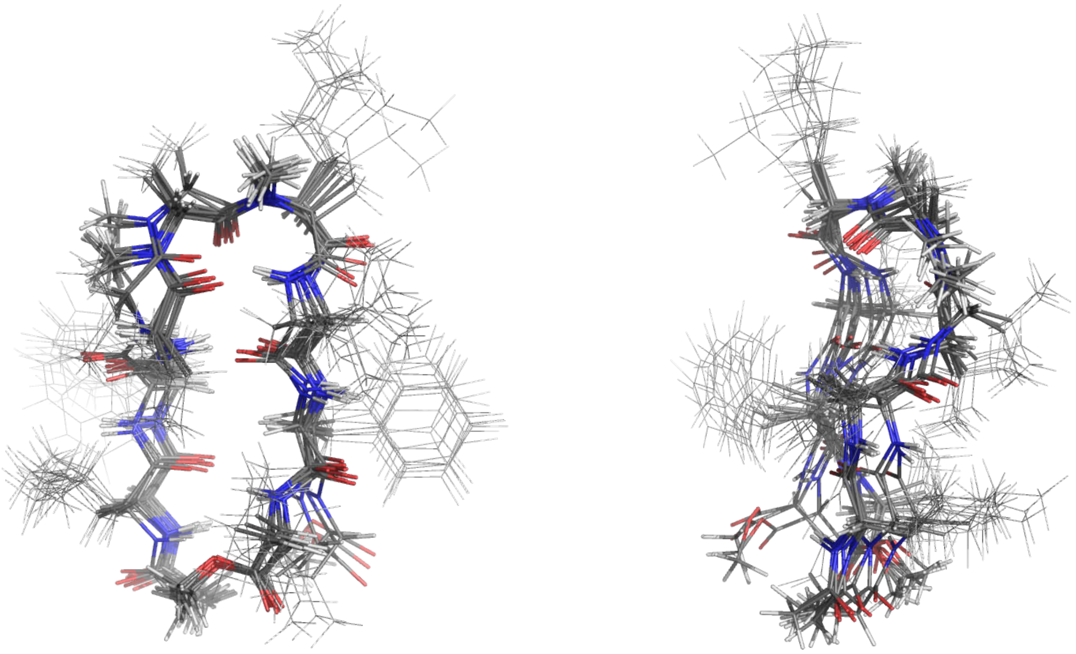

Figure S3.4: Overlay of 10 representative conformations generated using Molecular Dynamics simulation, showing both front view (left panel) and side view (right panel).

(A)

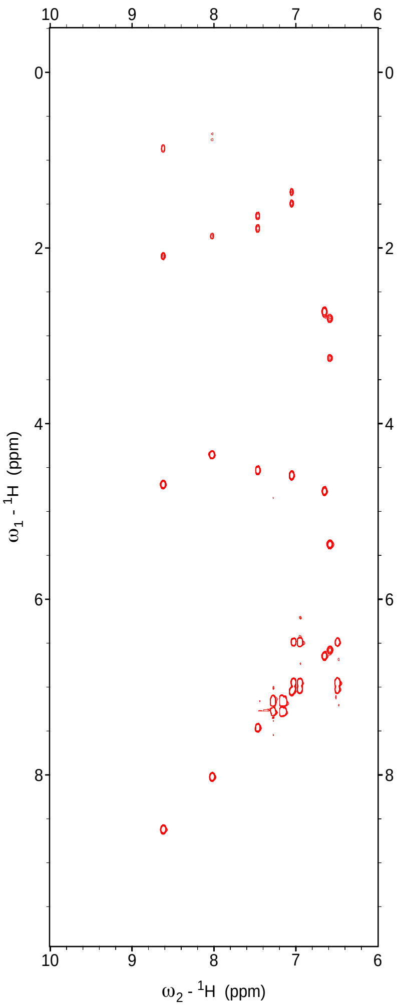

CDCl3

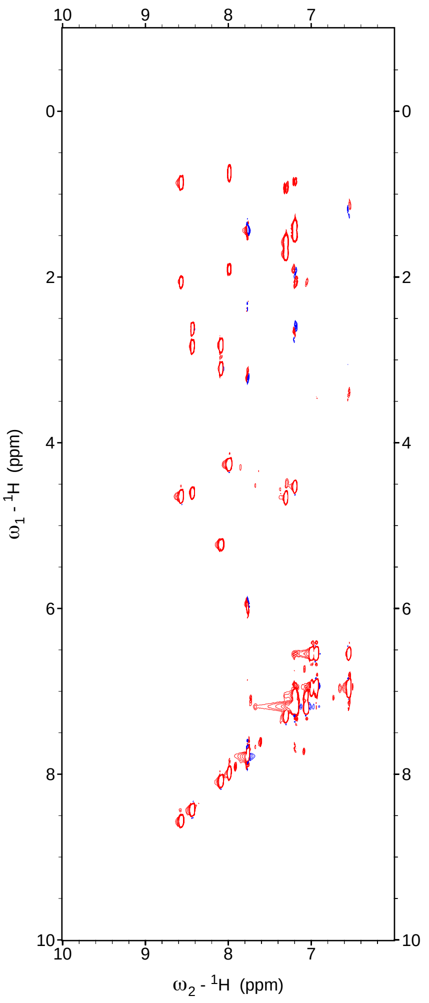

DMSO-d_6 – $CDCl_3$ (1:2)

(B)

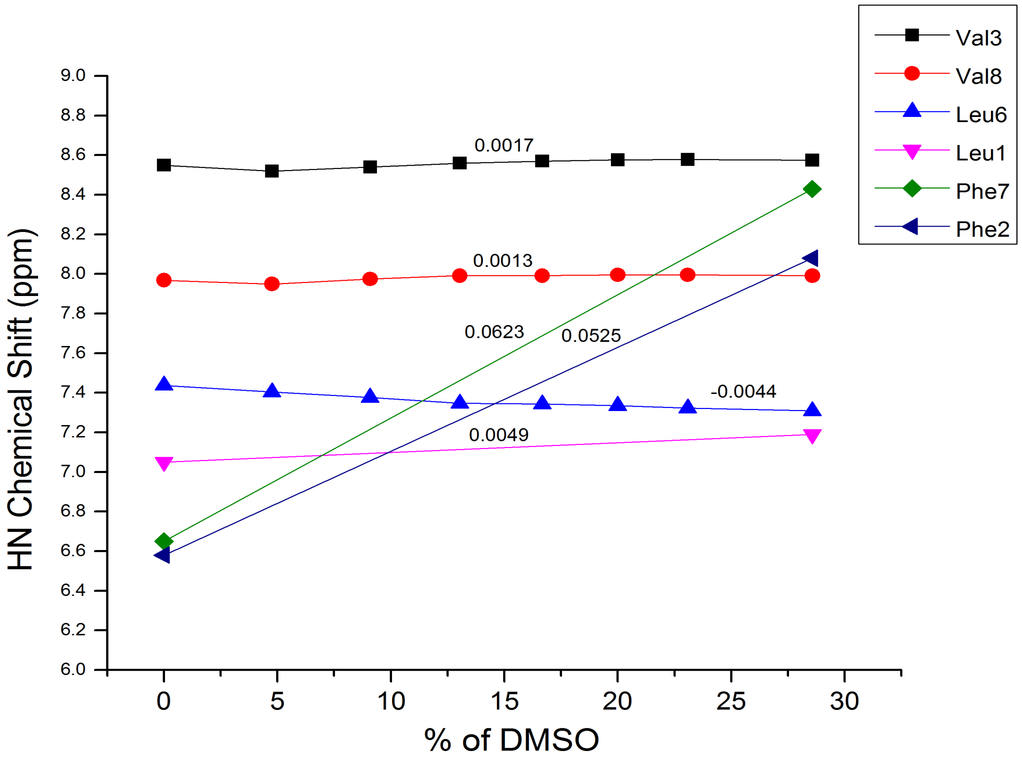

Figure S3.5: (A) TOCSY spectra in $CDCl_3$ and DMSO-d_6 – $CDCl_3$ (1:2) and (B) DMSO-d_6 titration curve indicating the solvent exposed (F2, F7) and solvent shielded (L1, V3, L6, V8) amide protons. The value indicates the slope generated by the linear fit of the data points.

Compound 4:

(A)

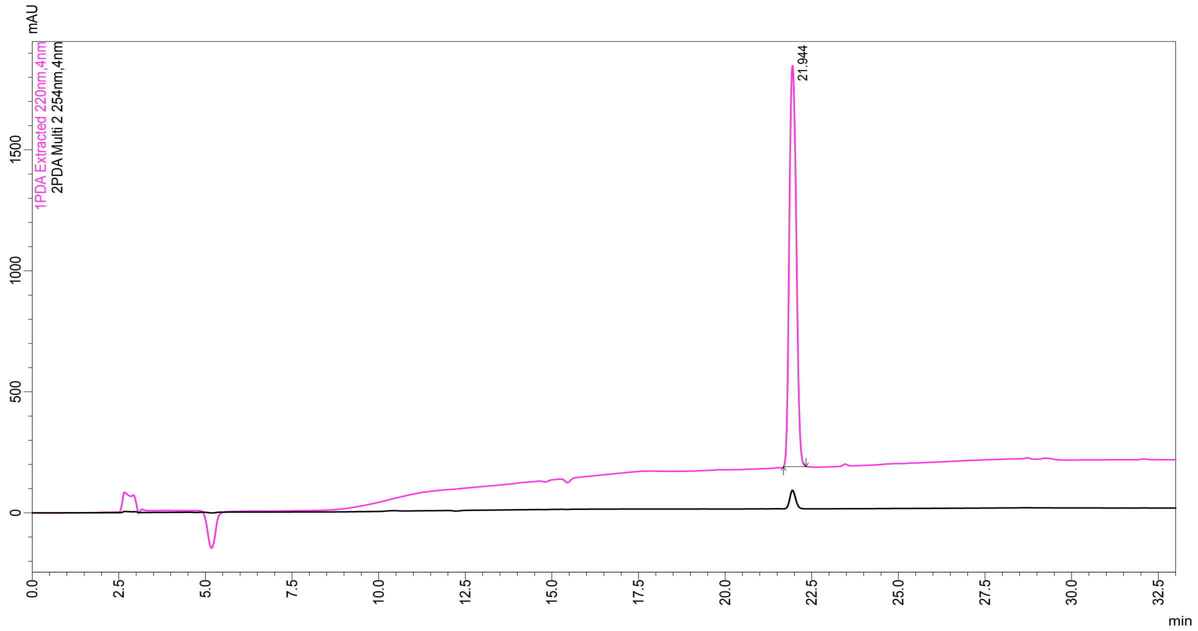

(B)

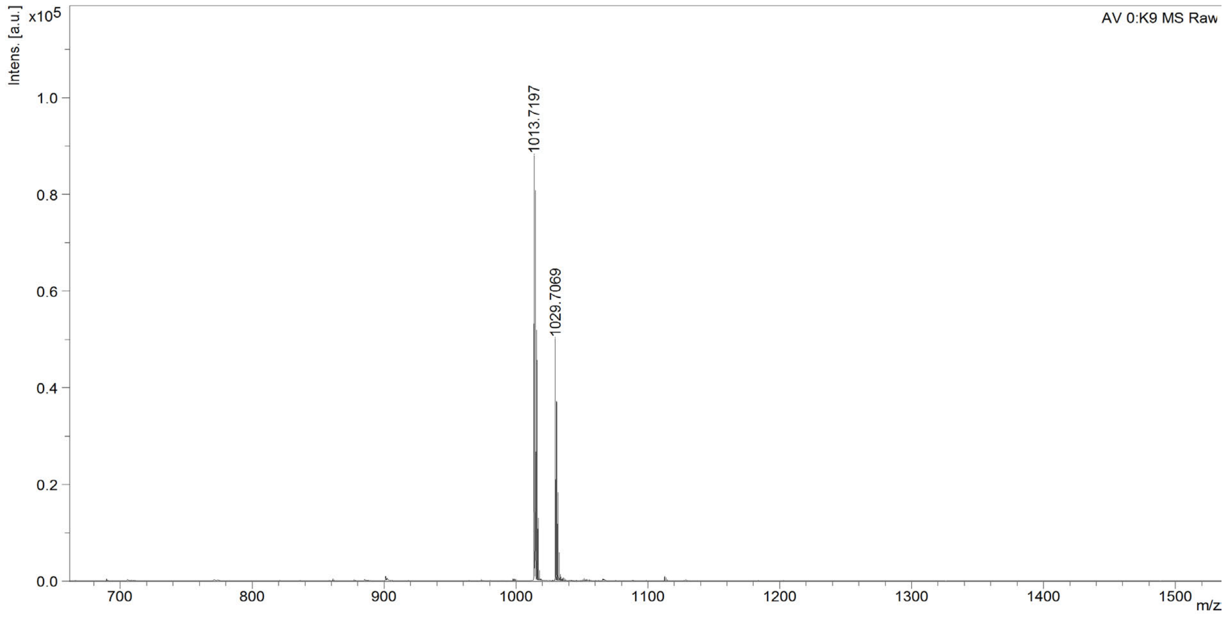

Figure S4.1: A) Analytical HPLC chromatogram of purified compound **4** at 70-100% MeOH/H_2O gradient and (B) the respective MALDI profile of the pure compound. Calculated MW: 1013.6154 [M+Na]; Observed MW: 1013.7197.

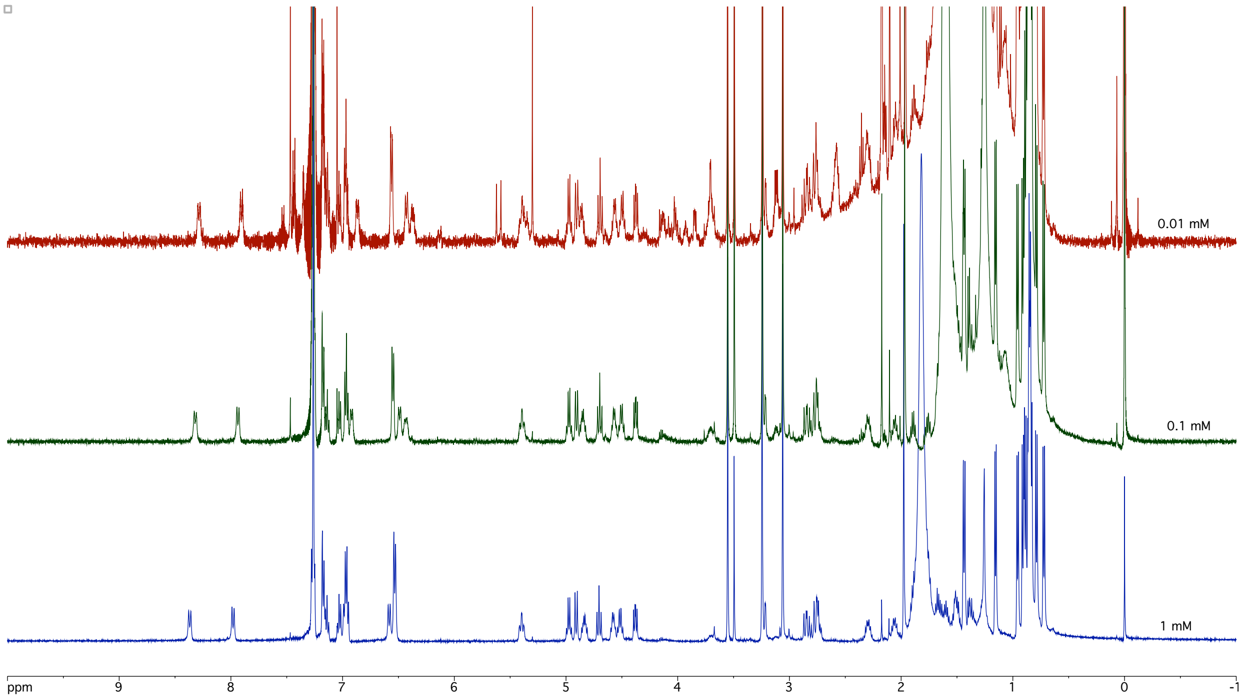

Figure S4.2: ^{1}H NMR spectra of Compound **4** at three different dilutions in $CDCl_3$ at 25°C.

Table 4.1: Chemical shifts table.

**Residue**	**Atoms**									
	**HN**	**NMe**	**HA**	**HB**		**HG**		**HD**	**OMe**	**NAc**
				1	2	1	2			
**LEU1**	6.96		4.58	1.37	1.5			0.85		1.97
**PHE2**	6.52		5.39	3.22	2.85					
**VAL3**	8.35		4.7	2.06		0.87				
**D-ALA4**		3.23	4.97	1.43						
**VAL5**		3.04	4.9	2.29		1.15	0.83			
**LEU6**	7.27		4.51	1.75	1.65			0.93		
**PHE7**	6.56		4.83	2.76						
**VAL8**	7.96		4.38	1.9		0.75			3.55	

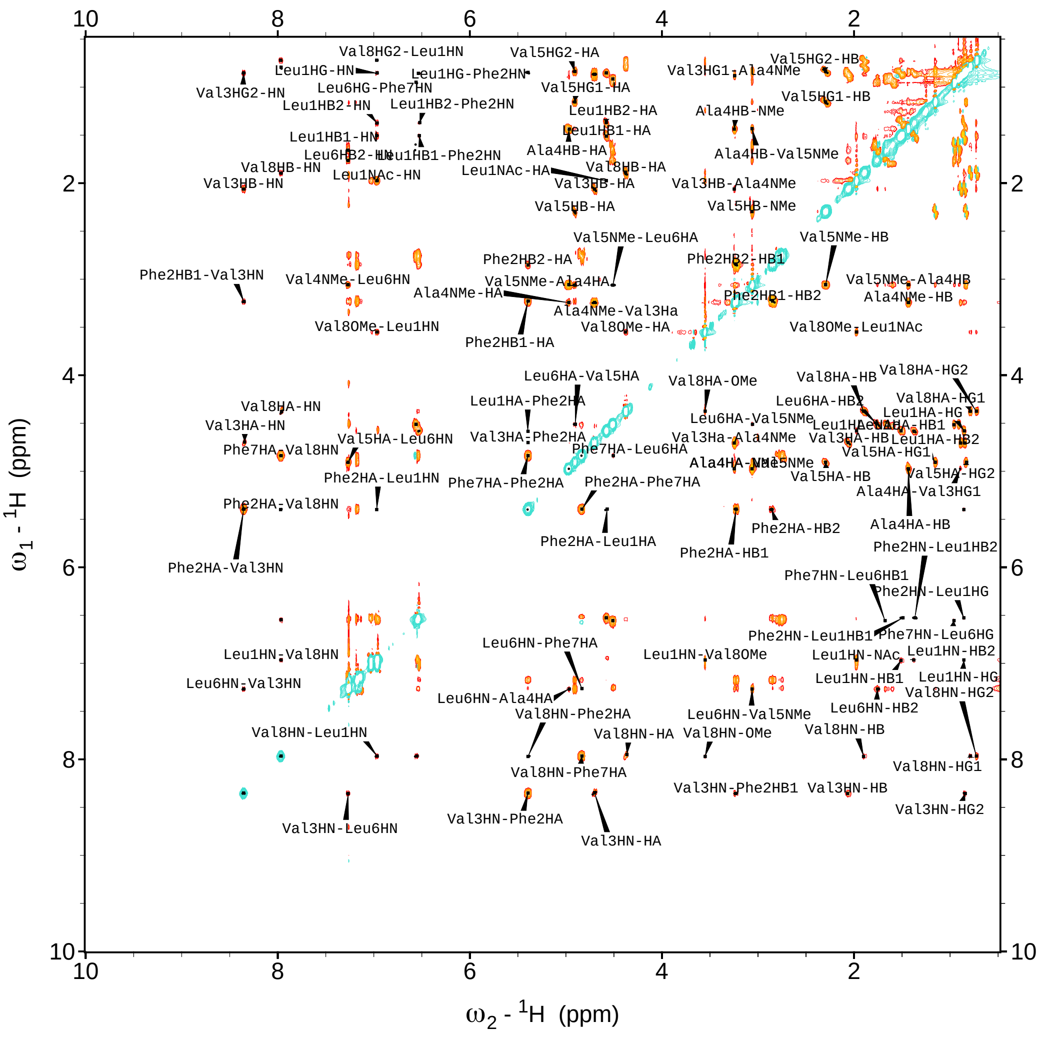

Figure S4.3: ROESY spectra with assigned peaks.

Table4.2: List of ROEs with respective NMR distances and violations.

Interactions	NMR Distance	Lower Limit	Upper Limit	Observed Distance	Violations
Leu1HB1-HA	2.62	2.35	2.88	2.63	0
Leu1HB1-HN	3.23	2.91	3.55	3.23	0
Leu1HA-HB2	2.81	2.53	3.09	3	0
Leu1HB2-HN	3.41	3.07	3.76	3.06	0
Leu1HG-HA	2.67	2.4	2.94	3.06	0.1
Leu1HG-HN	3.85	3.47	4.24	4.31	0.1
Leu1HN-Val8HN	3.62	3.26	3.98	3.05	-0.2
Leu1NAc-HA	3.63	3.26	4.39	4.4	0
Leu1NAc-HN	2.50	2.25	3.15	2.77	0
Phe2HA-Val3HN	2.42	2.18	2.66	2.13	0
Phe2HB1-HA	2.52	2.27	2.77	2.63	0
Phe2HB1-Val3HN	3.48	3.13	3.83	3.61	0
Phe2HB2-HA	3.06	2.76	3.37	2.39	-0.4

**Phe2HB2-HB1**	1.80	1.62	1.98	1.73	0
**Val3HA-HG1**	2.63	2.37	3.29	3.14	0
**Val3HA-HG2**	2.56	2.3	3.22	3.13	0
**Val3HA-HN**	3.25	2.93	3.58	2.95	0
**Val3HB-HA**	2.72	2.44	2.99	3.04	0.1
**Val3HB-HN**	3.09	2.78	3.4	2.77	0
***Val3HB-ala4NMe**	3.42	3.08	4.16	4.62	0.5
**Val3HG1-ala4NMe**	3.09	2.78	4.2	3.54	0
**Val3HG2-HN**	3.37	3.03	4.11	3.75	0
**ala4HA-Val3HG1**	3.55	3.2	4.31	4.49	0.2
**ala4HA-HB**	2.32	2.09	2.95	2.48	0
**ala4HB-NMe**	2.27	2.04	3.29	3.29	0
**ala4HB-Val5NMe**	2.87	2.58	3.96	3.98	0
**ala4NMe-Val3HA**	2.05	1.84	2.65	2.66	0
**ala4NMe-HA**	3.01	2.71	3.72	3.61	0
**Val5HB-HA**	2.76	2.49	3.44	3.02	0
**Val5HG1-HA**	2.53	2.28	2.79	2.95	0.2
**Val5HG1-HB**	2.28	2.05	2.91	2.5	0
**Val5HG2-HB**	2.32	2.09	2.96	2.48	0
**Val5NMe-ala4HA**	2.03	1.83	2.63	2.59	0
**Val5NMe-HA**	3.08	2.77	3.78	3.79	0
**Val5NMe-HB**	2.41	2.17	3.45	3.13	0
**Leu6HA-Val5NMe**	3.92	3.52	4.71	4.91	0.2
**Leu6HA-HB2**	2.72	2.45	2.99	2.99	0
**Leu6HN-HB2**	3.04	2.74	3.35	3.04	0
**Leu6HG-HA**	2.44	2.19	2.68	2.88	0.2
**Leu6HN-Val3HN**	3.53	3.18	3.89	3.2	0
**Leu6HN-ala4HA**	3.41	3.07	3.75	3.6	0
**Leu6HN-Val5NMe**	2.81	2.53	3.49	3.48	0
**Phe7HA-Phe2HA**	2.41	2.17	2.65	2.79	0.1
**Phe7HA-Val8HN**	2.31	2.08	2.54	2.09	0
**Val8HA-HG1**	2.76	2.48	3.43	3.02	0
**Val8HA-HG2**	2.75	2.47	3.42	3.24	0
**Val8HA-HN**	3.05	2.74	3.35	2.98	0
**Val8HB-HA**	2.48	2.24	2.73	2.98	0.3
**Val8HB-HN**	3.15	2.83	3.46	2.9	0
**Val8HN-HG2**	3.12	2.81	3.84	3.55	0
**Leu1HN-Val8OMe**	3.38	3.04	4.12	4.19	0.1
**Val8OMe-Leu1NAc**	3.10	2.79	4.21	3.94	0
**Val8OMe-HA**	3.25	2.93	3.98	4.23	0.3

* violations ≥0.5. The observed high violations can be explained by the local flexibility about the γ and δ methyl groups (Val and Leu respectively) and the terminal ester bond, peak overlap, additional J-mediated transfer and inaccuracies in the force fields.[9]

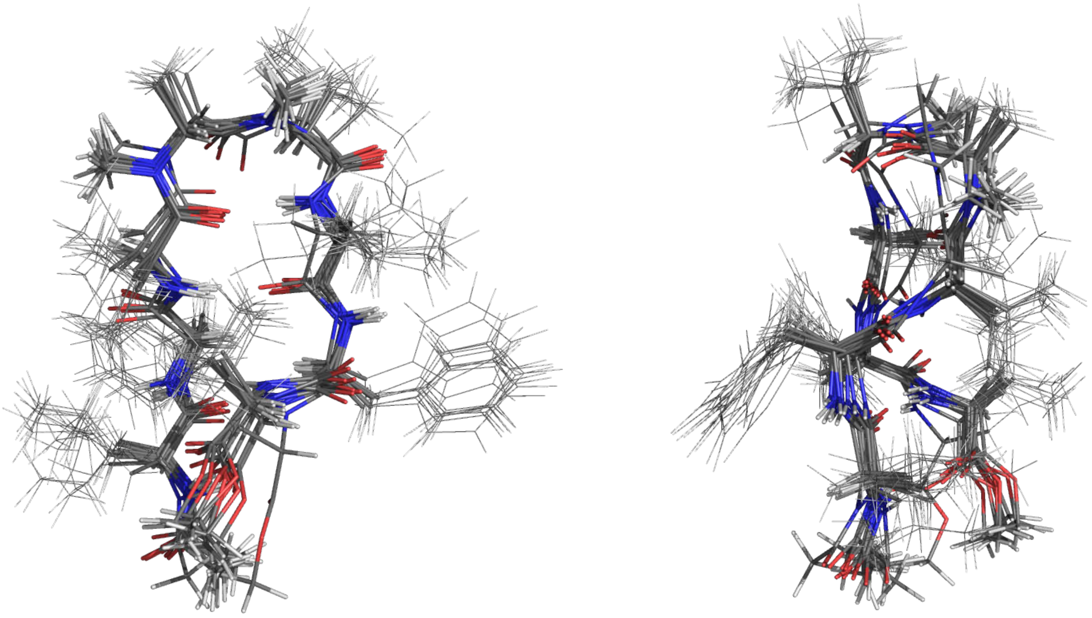

Figure S4.4: Overlay of 10 representative conformations generated using Molecular Dynamics simulation, showing both front view (left panel) and side view (right panel).

(A)

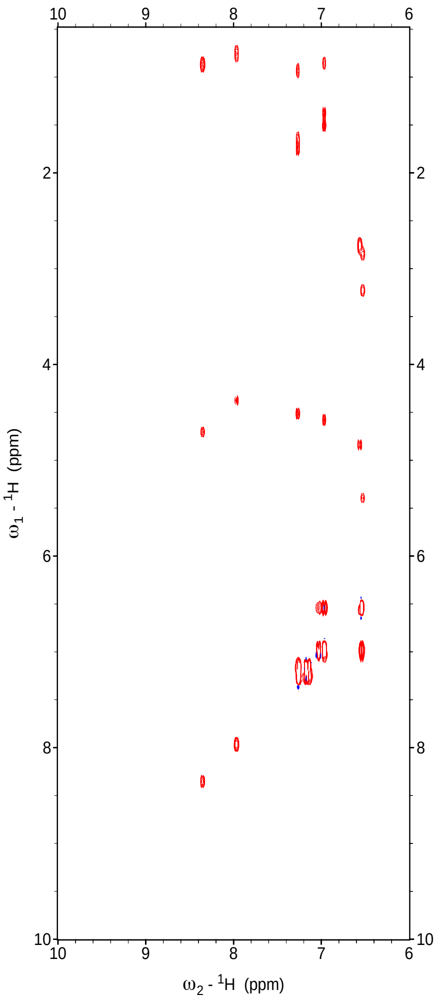

$CDCl_3$

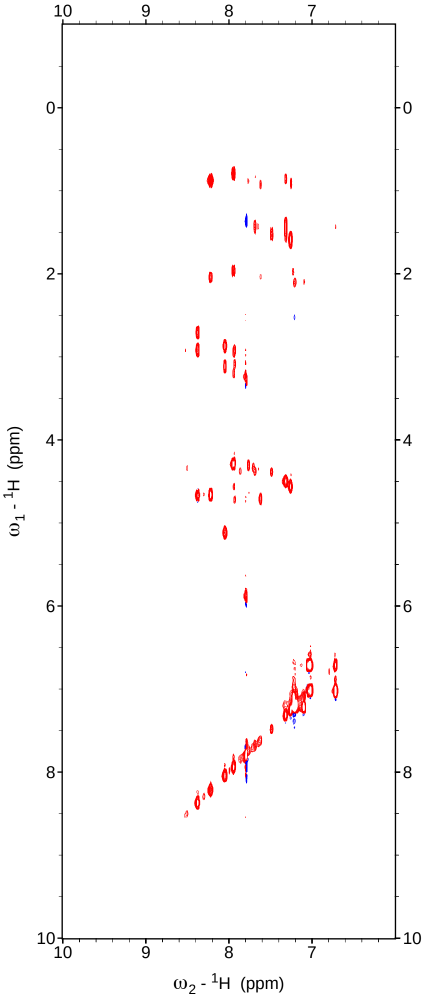

DMSO-d_6 – $CDCl_3$
(1:2)

(B)

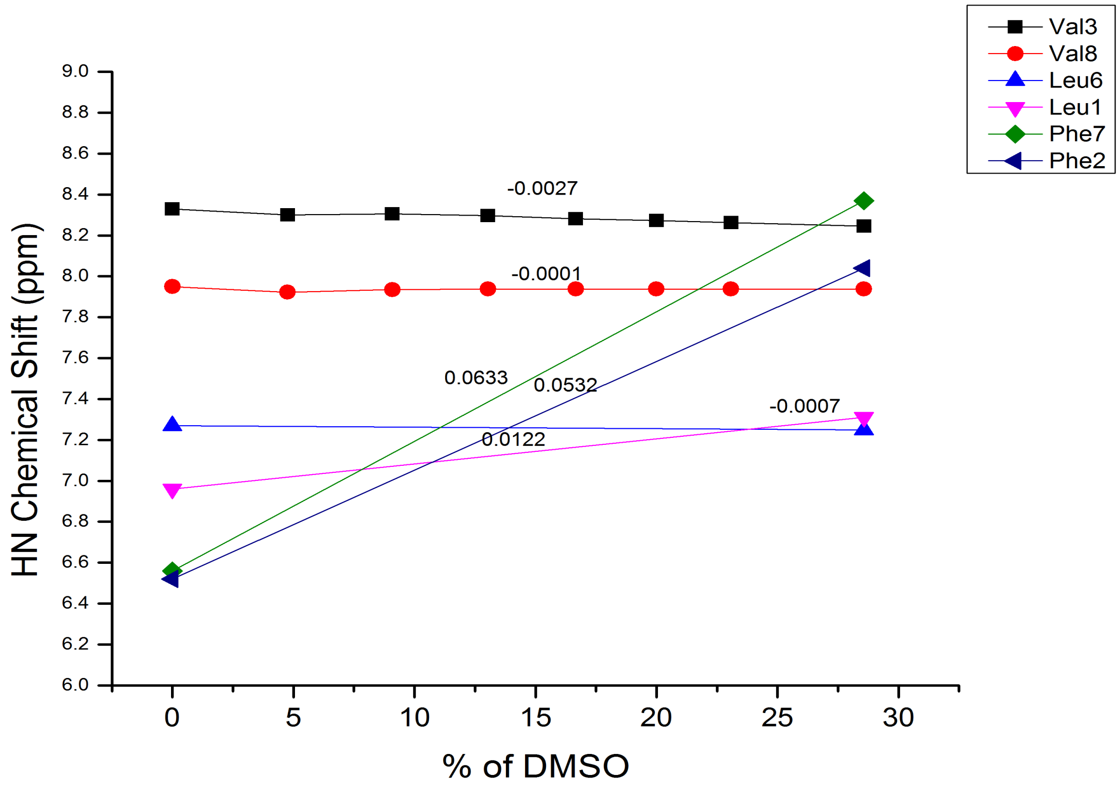

Figure S4.5: (A) TOCSY spectra in $CDCl_3$ and DMSO-d_6 – $CDCl_3$ (1:2) and (B) DMSO-d_6 titration curve indicating the solvent exposed (F2, F7) and solvent shielded (V3, L6, V8) amide protons. The value indicates the slope generated by the linear fit of the data points.

Compound 5:

(A)

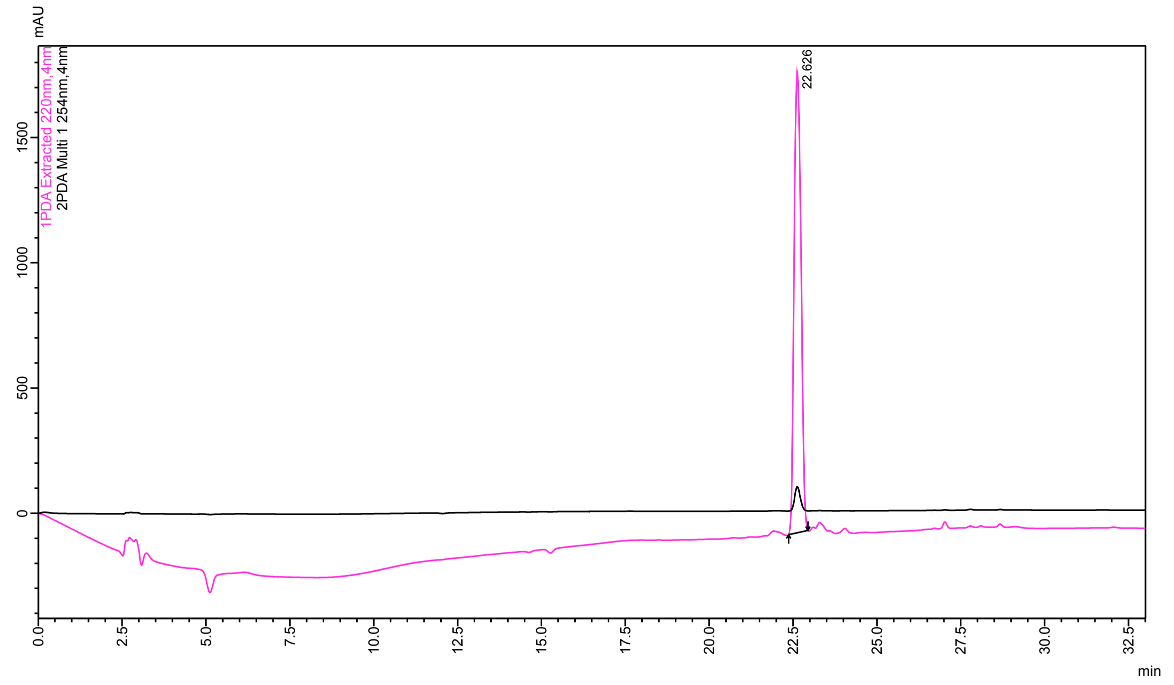

(B)

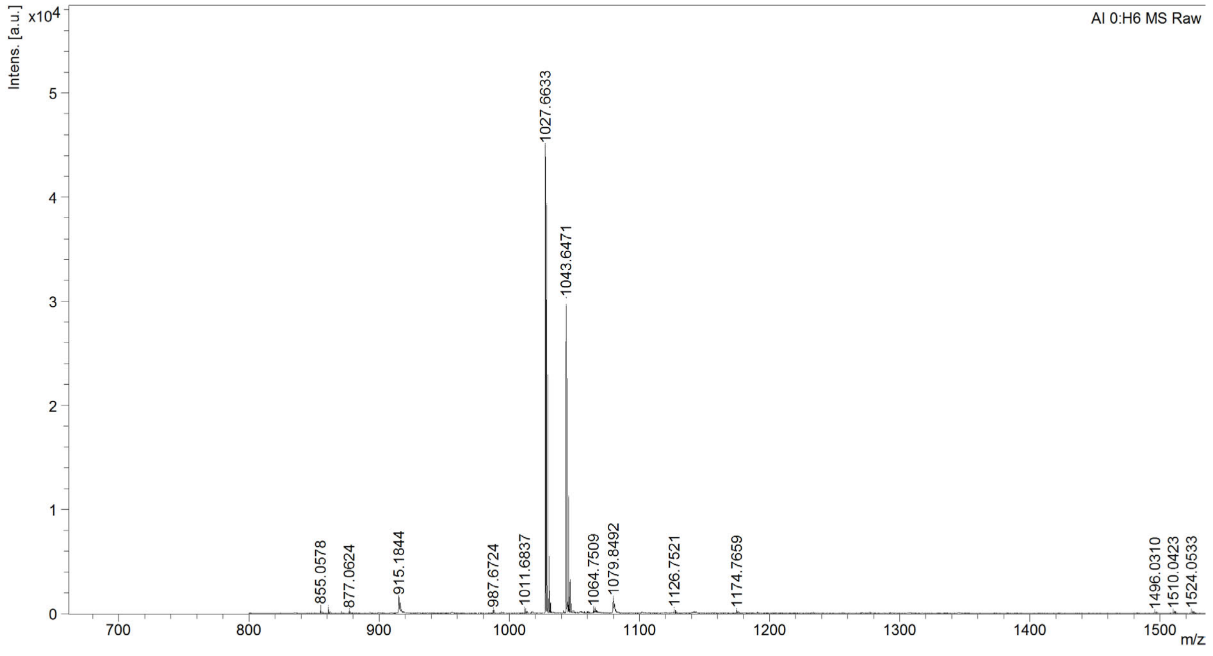

Figure S5.1: A) Analytical HPLC chromatogram of purified compound **5** at 70-100% MeOH/H_2O gradient and (B) the respective MALDI profile of the pure compound. Calculated MW: 1027.6310 [M+Na]; Observed MW: 1027.6633.

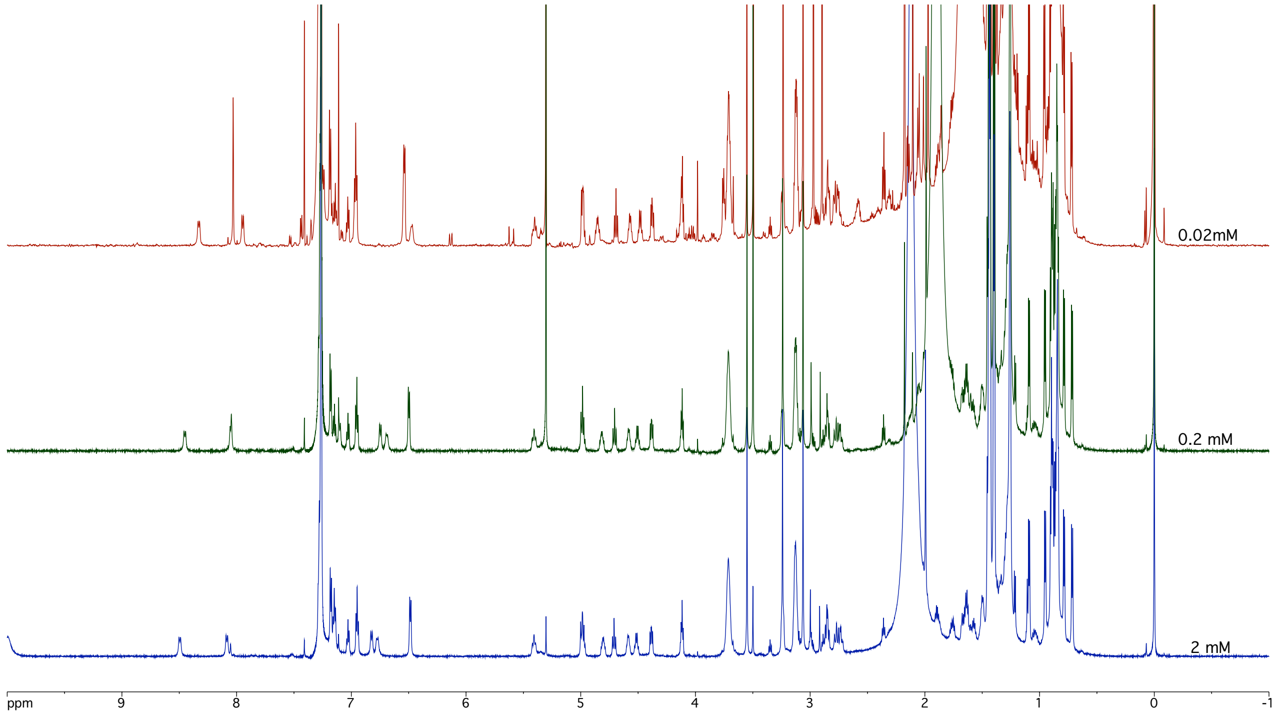

Figure S5.2: ^{1}H NMR spectra of Compound **5** at three different dilutions in $CDCl_3$ at 25°C.

Table 5.1: Chemical shifts table.

Residue	Atoms										
	HN	NMe	HA	HB		HG		HD		OMe	NAc
				1	2	1	2	1	2		
LEU1	7.10		4.59	1.38	1.52			0.86			1.98
PHE2	6.68		5.41	2.86	3.24						
VAL3	8.47		4.71	2.06		0.9	0.86				
D-ALA4		3.23	4.99	1.43							
ILE5		3.05	4.99	2.13		1.35	1.08	0.85			
LEU6	7.35		4.49	1.77	1.67	1.61		0.96	0.91		
PHE7	6.70		4.85	2.76							
VAL8	8.07		4.38	1.91		0.8	0.74			3.55	

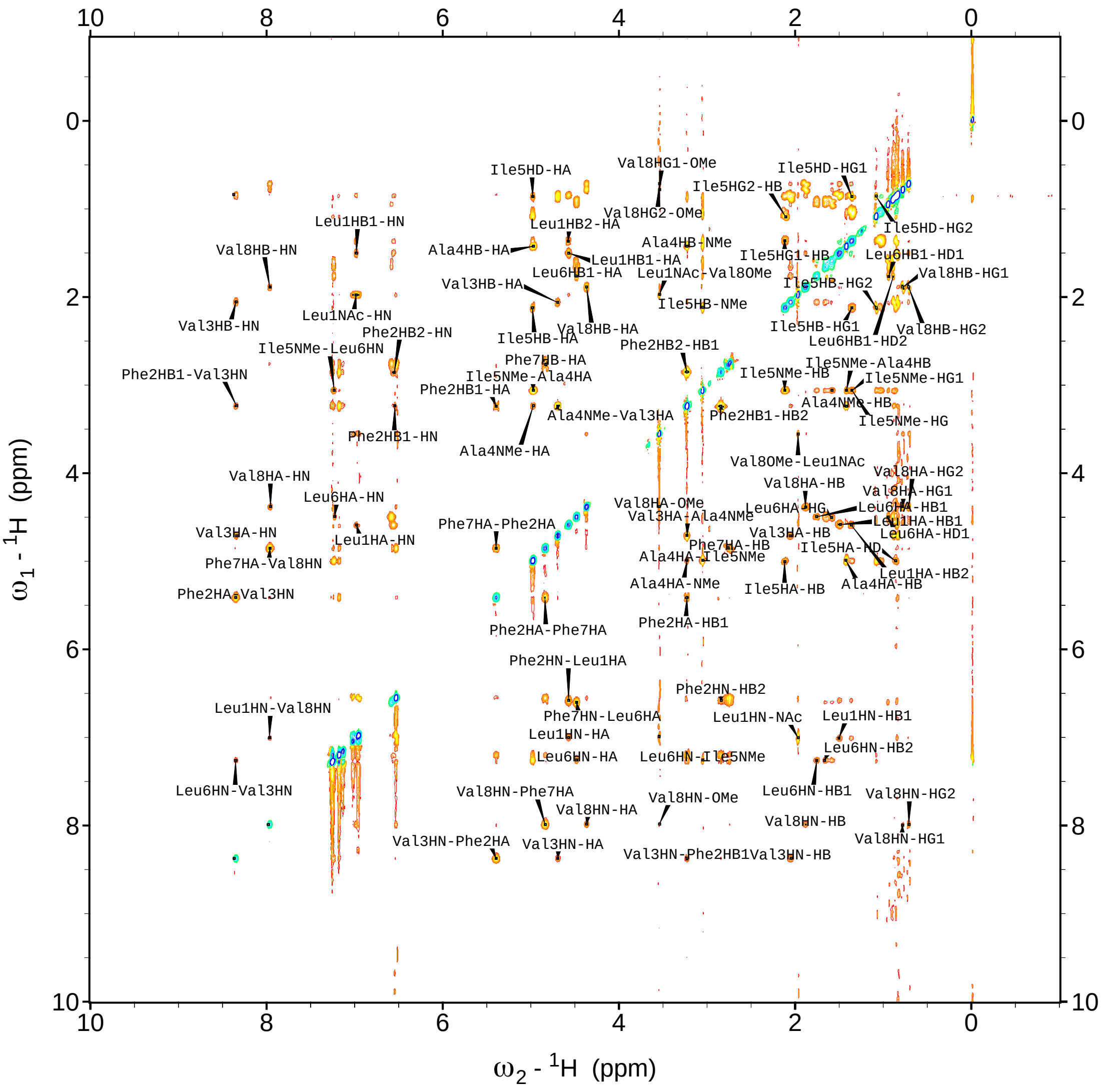

Figure S5.3: ROESY spectra with assigned peaks.

Table5.2: List of ROEs with respective NMR distances and violations.

Interactions	NMR Distance	Upper Limit	Lower Limit	Observed Distance	Violations
**Leu1HA-HN**	3.01	2.71	3.32	2.94	0
**Leu1HB1-HA**	2.3	2.07	2.53	2.81	0.3
**Leu1HB1-HN**	2.6	2.34	2.86	2.76	0
**Leu1HB2-HA**	2.62	2.36	2.88	2.81	0
**Leu1NAc-HN**	2.19	1.97	2.81	2.77	0
**Phe2HA-Val3HN**	2.18	1.96	2.39	2.08	0
**Phe2HB1-HA**	2.5	2.25	2.75	2.56	0
**Phe2HB1-Val3HN**	2.82	2.54	3.11	3.33	0.2
**Phe2HB2-HB1**	1.81	1.63	1.99	1.74	0
***Phe2HN-Leu1HA**	2.36	2.12	2.6	3.1	0.5
**Phe2HN-HB1**	3.08	2.77	3.39	3.82	0.4

**Phe2HB2-HN**	3.12	2.81	3.43	3.51	0.1
**Val3HA-HN**	3.04	2.73	3.34	2.97	0
**Val3HB-HA**	2.55	2.29	2.8	2.89	0.1
**Val3HN-HB**	2.44	2.2	2.69	2.74	0.1
**ala4HB-HA**	2.03	1.83	2.63	2.42	0
**ala4HB-NMe**	2.05	1.84	3.05	3.29	0.2
**ala4NMe-HA**	2.74	2.46	3.41	3.62	0.2
**ala4NMe-Val3HA**	1.93	1.73	2.52	2.61	0.1
**Ile5HA-Leu6HN**	2.31	2.08	2.54	2.69	0.1
**Ile5HB-HA**	2.54	2.28	3.19	3	0
**Ile5HB-NMe**	2.22	2	3.25	3.01	0
***Ile5NMe-ala4HB**	2.52	2.27	3.57	4.09	0.5
**Ile5NMe-Leu6HG**	2.67	2.4	3.34	3.2	0
**Ile5NMe-Leu6HN**	2.57	2.31	3.22	3.38	0.2
**Leu6HA-HN**	2.67	2.4	2.93	2.93	0
**Leu6HB1-HA**	2.41	2.17	2.65	3.01	0.4
**Leu6HB1-HD1**	2.56	2.31	2.82	2.83	0
***Leu6HB1-HD2**	2.76	2.48	3.03	3.81	0.8
**Leu6HN-HB1**	2.82	2.54	3.1	2.69	0
**Leu6HN-Val3HN**	3.01	2.71	3.32	3.39	0.1
**Phe7HN-Leu6HA**	2.16	1.94	2.37	2.45	0.1
**Phe7HA-Val8HN**	2.12	1.91	2.33	2.11	0
**Phe7HA-Phe2HA**	2.32	2.08	2.55	2.38	0
***Val8HA-HG1**	2.48	2.23	2.73	3.44	0.7
**Val8HA-HG2**	2.6	2.34	2.86	2.75	0
**Val8HA-HN**	2.86	2.57	3.14	2.8	0
**Val8HA-OMe**	2.36	2.12	2.59	2.71	0.1
**Val8HB-HA**	2.36	2.12	2.59	3	0.4
**Val8HB-HG1**	2.21	1.99	2.44	2.46	0
**Val8HB-HG2**	2.25	2.02	2.47	2.47	0
**Val8HB-HN**	2.91	2.62	3.2	2.53	-0.1
**Val8HG1-OMe**	2.49	2.24	3.54	3.46	0
***Val8HN-HG1**	3.4	3.06	3.74	4.21	0.5
***Val8HN-OMe**	3.46	3.11	3.81	4.63	0.8
**Leu1HN-Val8HN**	3.01	2.71	3.31	3.5	0.2
**Leu1NAc-Val8OMe**	2.75	2.48	3.83	3.59	0

* violations ≥0.5. The observed high violations can be explained by the local flexibility about the γ and δ methyl groups (Val and Leu respectively) and the terminal ester bond, peak overlap, additional J-mediated transfer and inaccuracies in the force fields.[9]

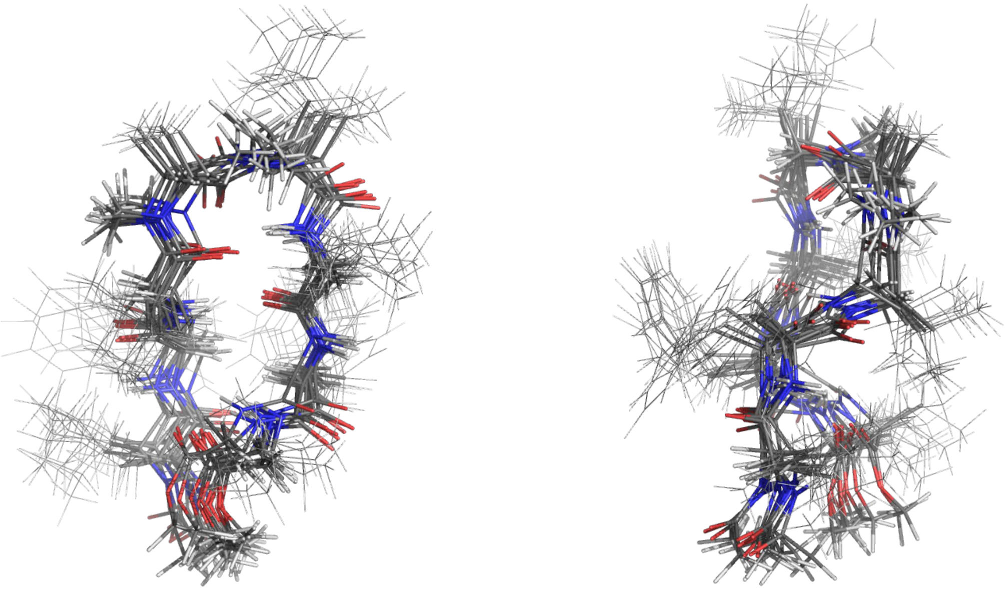

Figure S5.4: Overlay of 10 representative conformations generated using Molecular Dynamics simulation, showing both front view (left panel) and side view (right panel).

(A)

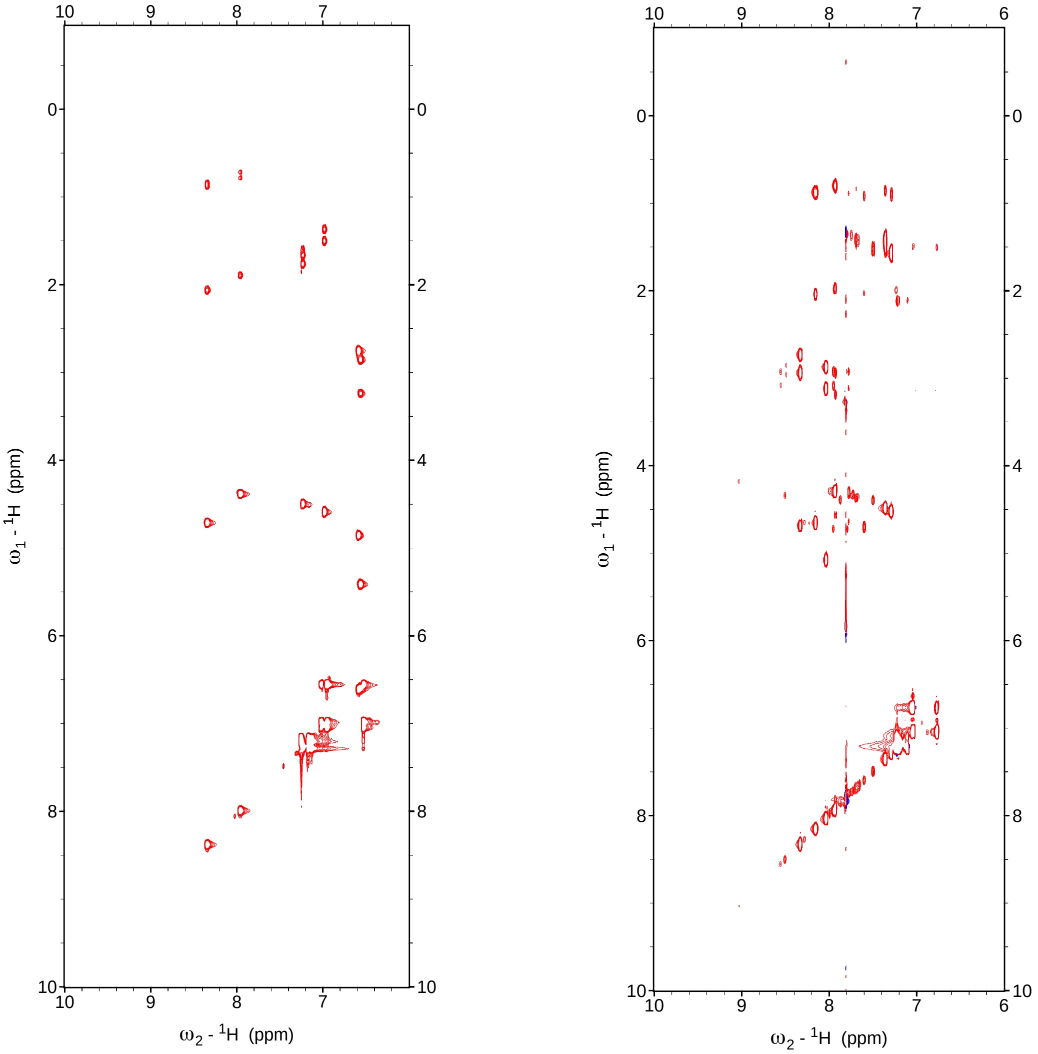

$CDCl_3$

DMSO-d_6 – $CDCl_3$ (1:2)

(B)

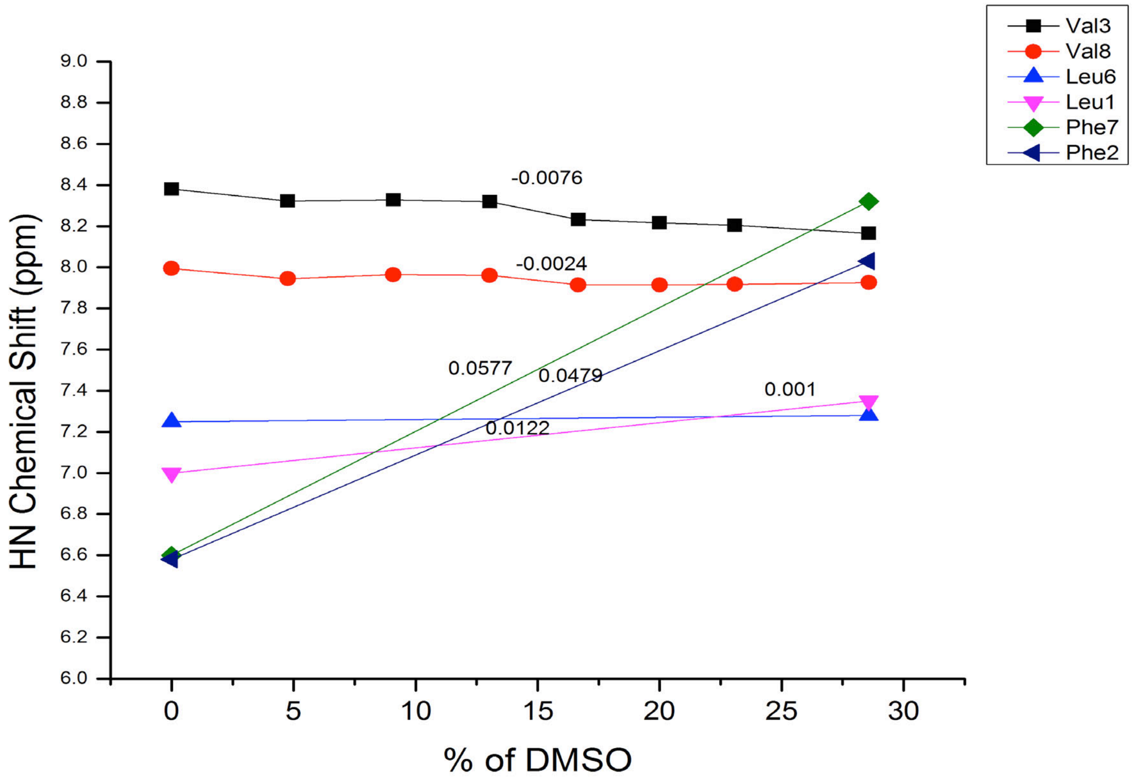

Figure S5.5: (A) TOCSY spectra in $CDCl_3$ and DMSO-d_6 – $CDCl_3$ (1:2) and (B) DMSO-d_6 titration curve indicating the solvent exposed (F2, F7) and solvent shielded (V3, L6, V8) amide protons. The value indicates the slope generated by the linear fit of the data points.

Compound 6:

(A)

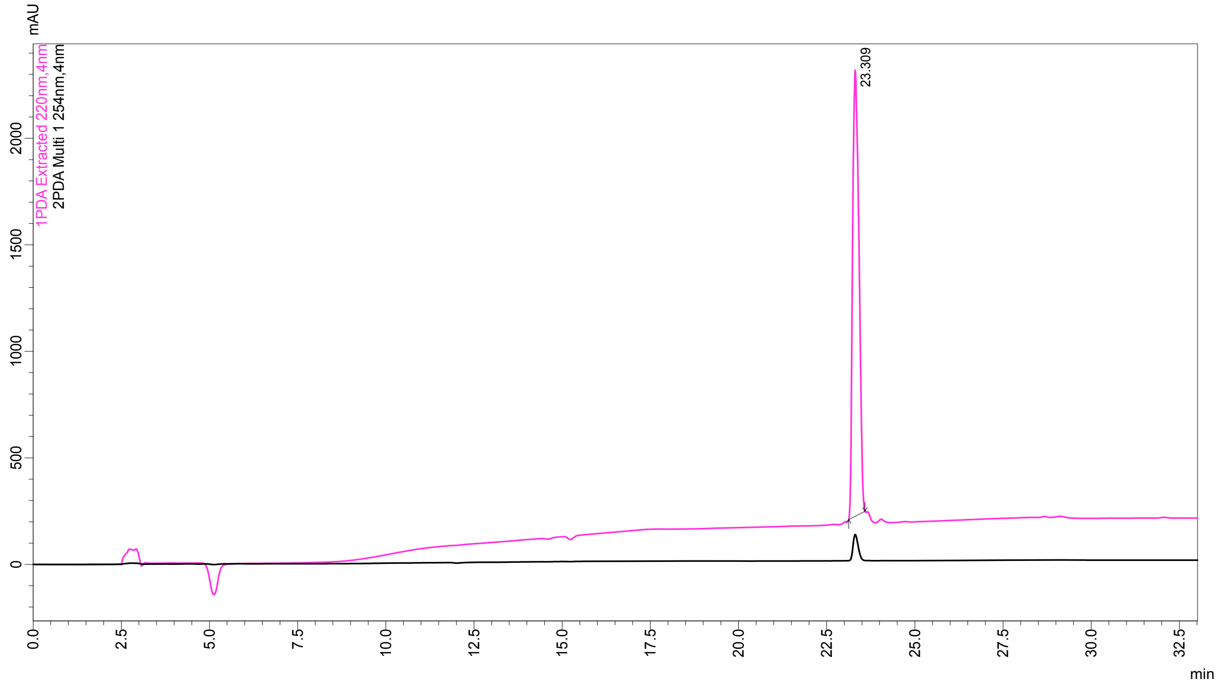

(B)

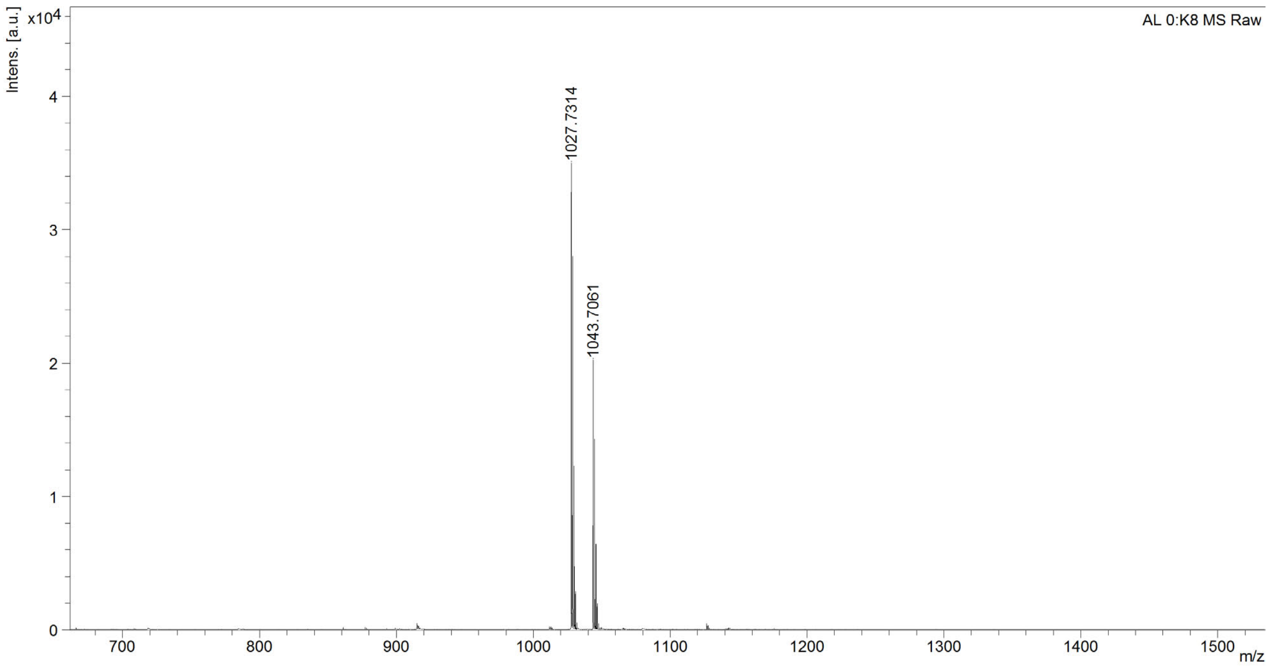

Figure S6.1: A) Analytical HPLC chromatogram of purified compound **6** at 70-100% MeOH/H_2O gradient and (B) the respective MALDI profile of the pure compound. Calculated MW: 1027.6310 [M+Na]; Observed MW: 1027.7314.

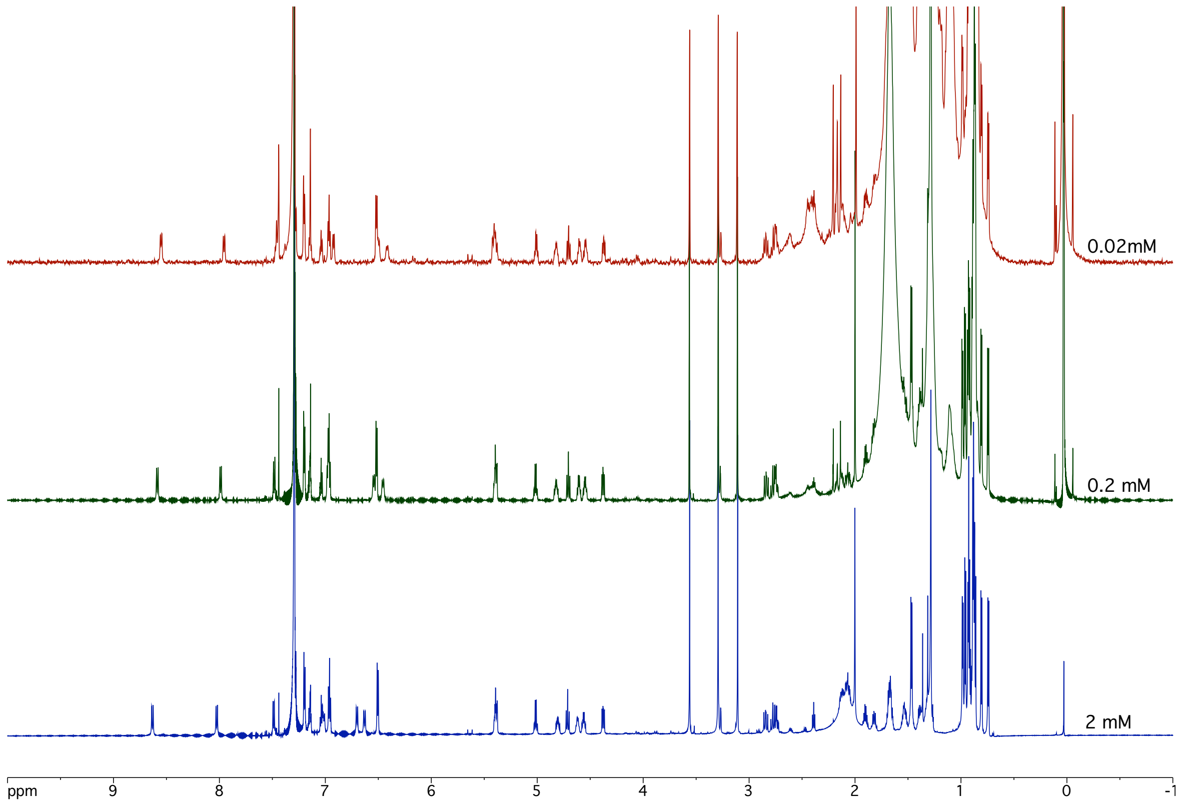

Figure S6.2: ^{1}H NMR spectra of Compound **6** at three different dilutions in $CDCl_3$ at 25°C.

Table 6.1: Chemical shifts table.

Residue	Atoms										
	HN	NMe	HA	HB		HG		HD		OMe	NAc
				1	2	1	2	1	2		
**LEU1**	7.02		4.54	1.47	1.33			0.83			1.97
**PHE2**	6.57		5.33	3.21	2.77						
**VAL3**	8.62		4.65	2.06		0.81	0.86				
**D-ALA4**		3.22	4.95	1.4							
**LEU5**		3.04	5.33	2	1.59	1.41		0.89	0.81		
**LEU6**	7.51		4.49	1.75	1.61			0.86	0.91		
**PHE7**	6.61		4.76	2.69							
**VAL8**	8.03		4.31	1.84		0.74	0.68			3.54	

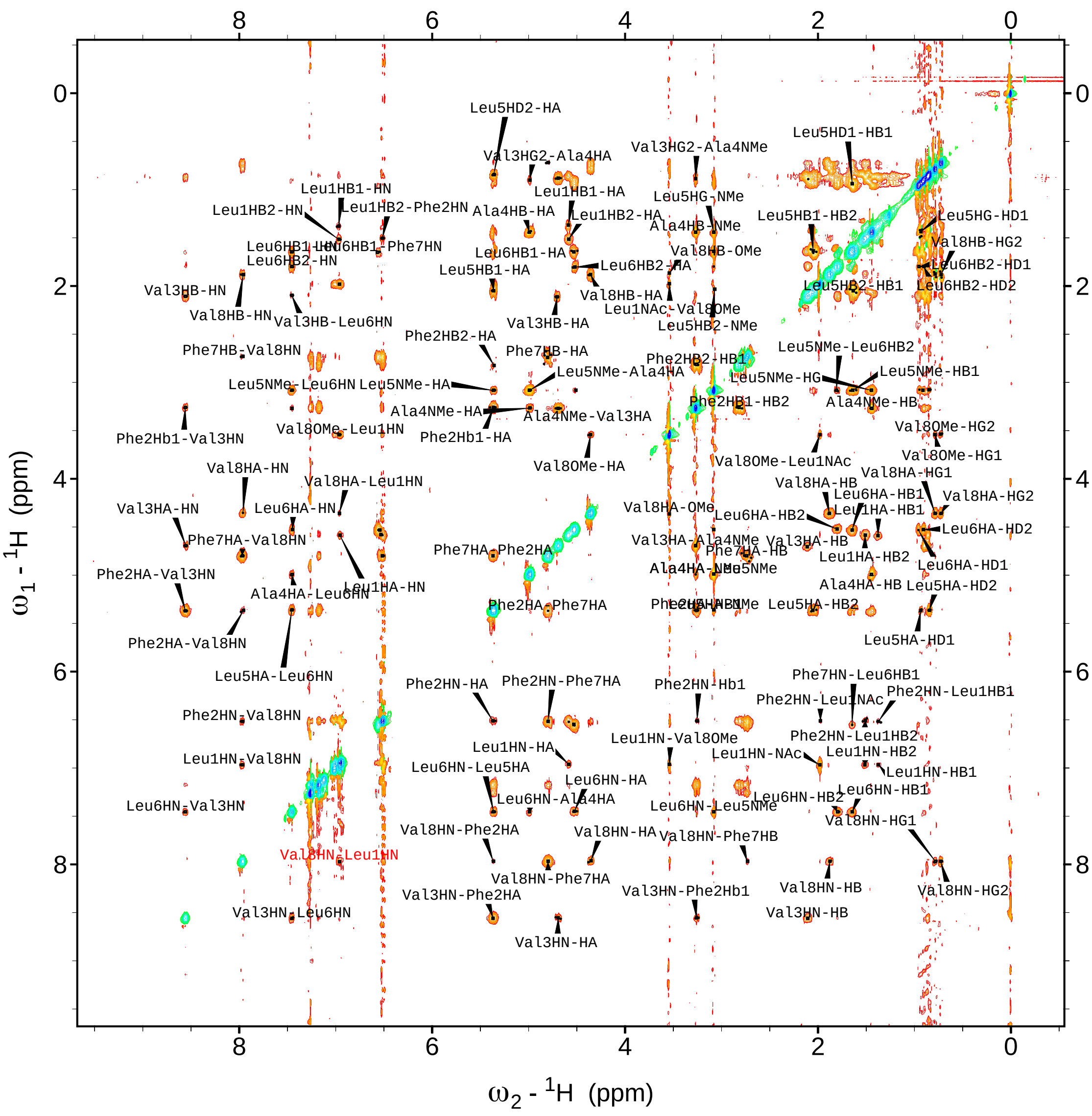

Figure S6.3: ROESY spectra with assigned peaks.

Table6.2: List of ROEs with respective NMR distances and violations.

Interactions	NMR Distance	Lower Limit	Upper Limit	Observed Distance	Violations
**Leu1HN-Val8HN**	3.37	3.03	3.71	3.36	0
**Leu1NAc-HN**	2.48	2.23	3.13	2.75	0
**Phe2HA-HB1**	2.62	2.36	2.88	2.83	0
**Phe2HB2-HA**	3.23	2.90	3.55	2.45	-0.5
**Phe2HB2-HB1**	1.98	1.78	2.18	1.73	-0.1
**Phe2HN-HA**	3.2	2.88	3.52	2.90	0
**Phe2HN-HB1**	3.92	3.52	4.31	3.46	-0.1
**Phe2HB1-Val3HN**	3.3	2.97	3.63	3.65	0
**Phe2HA-Val3HN**	2.37	2.13	2.61	2.09	0
**Val3HA-HN**	3.26	2.94	3.59	2.94	0

**Val3HB-HA**	2.96	2.66	3.25	2.97	0
**Val3HB-HN**	2.91	2.62	3.20	2.91	0
**ala4HB-HA**	2.15	1.94	2.77	2.49	0
**ala4HB-NMe**	2.16	1.94	3.17	3.28	0.1
**ala4NMe-HA**	3	2.70	3.70	3.57	0
**ala4HA-Leu6HN**	3.29	2.96	3.62	3.72	0.1
**ala4NMe-Val3HA**	2.14	1.93	2.76	2.65	0
**Leu5HA-HB2**	2.32	2.09	2.55	2.36	0
**Leu5HA-HD1**	3.32	2.99	4.05	4.48	0.4
**Leu5HB1-HA**	2.34	2.11	2.58	2.97	0.4
**Leu5HB1-HB2**	1.81	1.62	1.99	1.73	0
**Leu5HB1-NMe**	2.14	1.93	2.75	2.9	0.1
**Leu5HA-HD2**	2.36	2.12	2.99	2.82	0
**Leu5HG-NMe**	2.32	2.09	2.96	3.00	0
**Leu5NMe-HA**	3.02	2.72	3.73	3.79	0.1
**Leu5NMe-Leu6HN**	2.58	2.32	3.24	3.24	0
**Leu5HA-Leu6HN**	2.81	2.53	3.09	3.03	0
**Leu5NMe-ala4HA**	1.95	1.75	2.54	2.55	0
**Leu6HA-HD1**	2.89	2.6	3.57	3.98	0.4
**Leu6HA-HD2**	2.56	2.31	3.22	2.95	0
**Leu6HA-HN**	2.91	2.62	3.20	2.95	0
**Leu6HB1-HA**	2.42	2.18	2.66	2.40	0
**Leu6HB1-HN**	2.79	2.51	3.07	3.08	0
***Phe7HN-Leu6HB1**	3.22	2.90	3.55	4.03	0.5
**Leu5NMe-Leu6HB2**	3.06	2.75	3.77	4.08	0.3
**Leu6HB2-HA**	2.76	2.48	3.03	3.05	0
**Leu6HB2-HN**	2.82	2.54	3.10	2.55	0
**Leu6HN-Val3HN**	3.29	2.96	3.62	3.25	0
**Phe7HB-HA**	2.4	2.16	2.64	2.57	0
**Phe7HA-Val8HN**	2.21	1.99	2.43	2.52	0.1
**Phe7HA-Phe2HA**	2.46	2.21	2.70	2.83	0.1
**Val8HA-HN**	2.94	2.64	3.23	2.91	0
**Val8HB-HA**	2.45	2.21	2.70	2.49	0
**Val8HB-HN**	3.01	2.71	3.31	3.25	0
**Val8HN-HG1**	3.31	2.97	4.04	3.68	0
**Val8HN-HG2**	3.13	2.81	3.84	3.07	0
**Val8OMe-HG1**	3.36	3.03	4.50	4.56	0.1
**Val8OMe-HG2**	3.51	3.16	4.66	4.96	0.3
**Leu1NAc-Val8OMe**	2.98	2.68	4.08	3.92	0
**Leu1HN-Val8HN**	3.37	3.03	3.71	3.36	0

* violations ≥0.5. The observed high violations can be explained by the local flexibility about the γ and δ methyl groups (Val and Leu respectively) and the terminal ester bond, peak overlap, additional J-mediated transfer and inaccuracies in the force fields.[9]

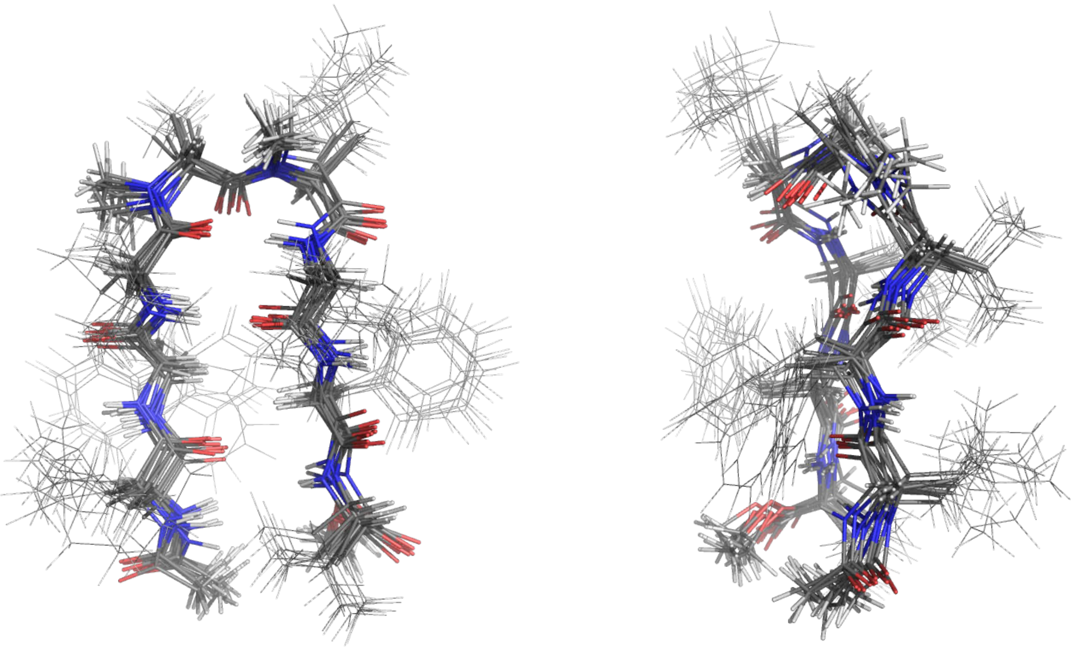

Figure S6.4: Overlay of 10 representative conformations generated using Molecular Dynamics simulation, showing both front view (left panel) and side view (right panel).

(A)

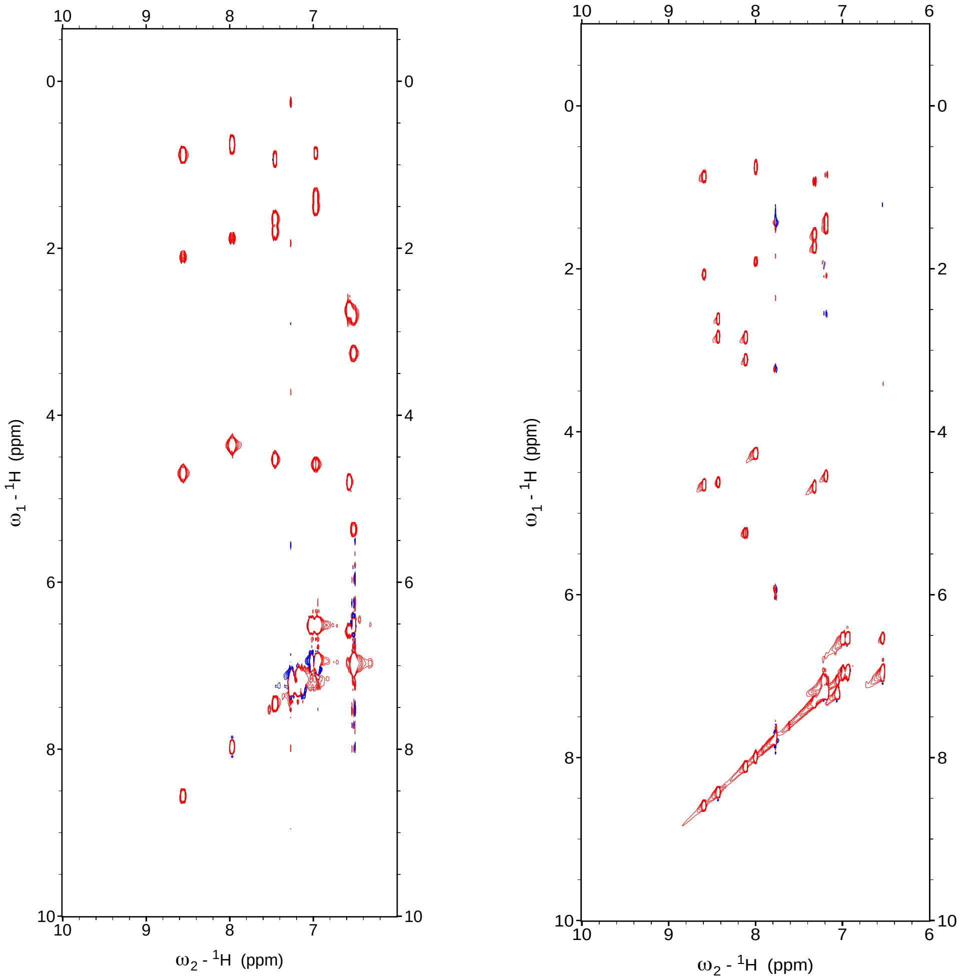

CDCl3

DMSO-d_6 – $CDCl_3$
(1:2)

(B)

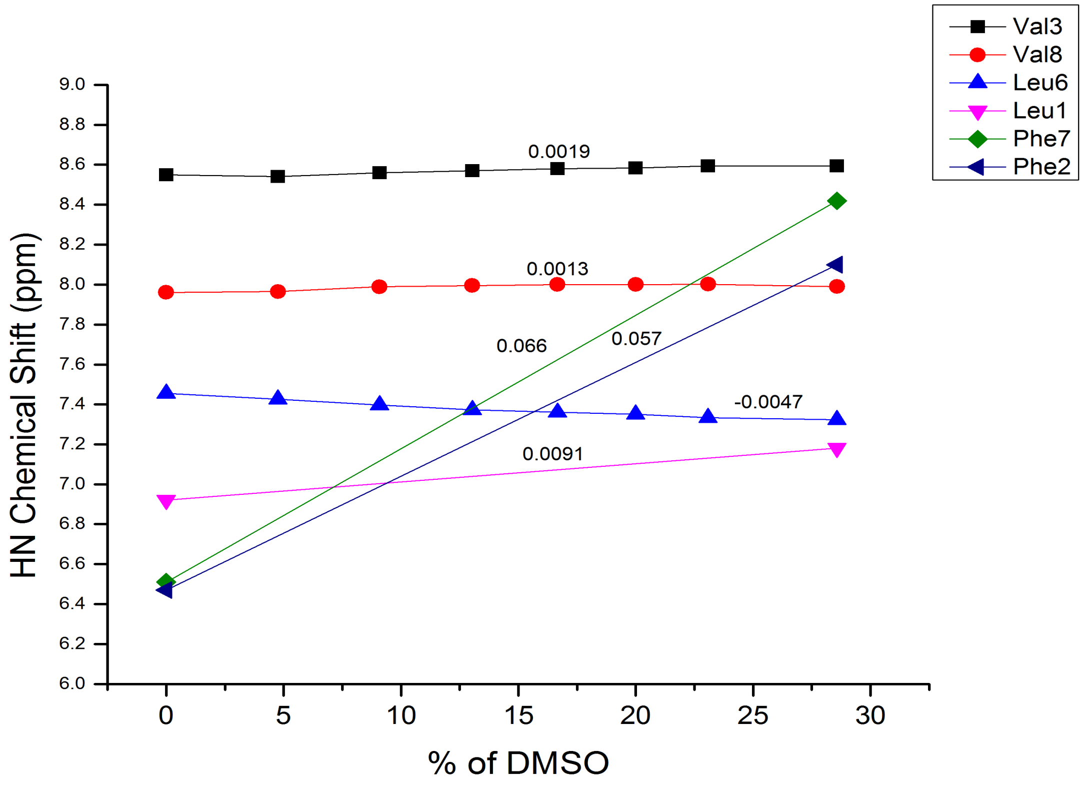

Figure S6.5: (A) TOCSY spectra in $CDCl_3$ and DMSO-d_6 – $CDCl_3$ (1:2) and (B) DMSO-d_6 titration curve indicating the solvent exposed (F2, F7) and solvent shielded (L1, V3, L6, V8) amide protons. The value indicates the slope generated by the linear fit of the data points.

Compound 7:

(A)

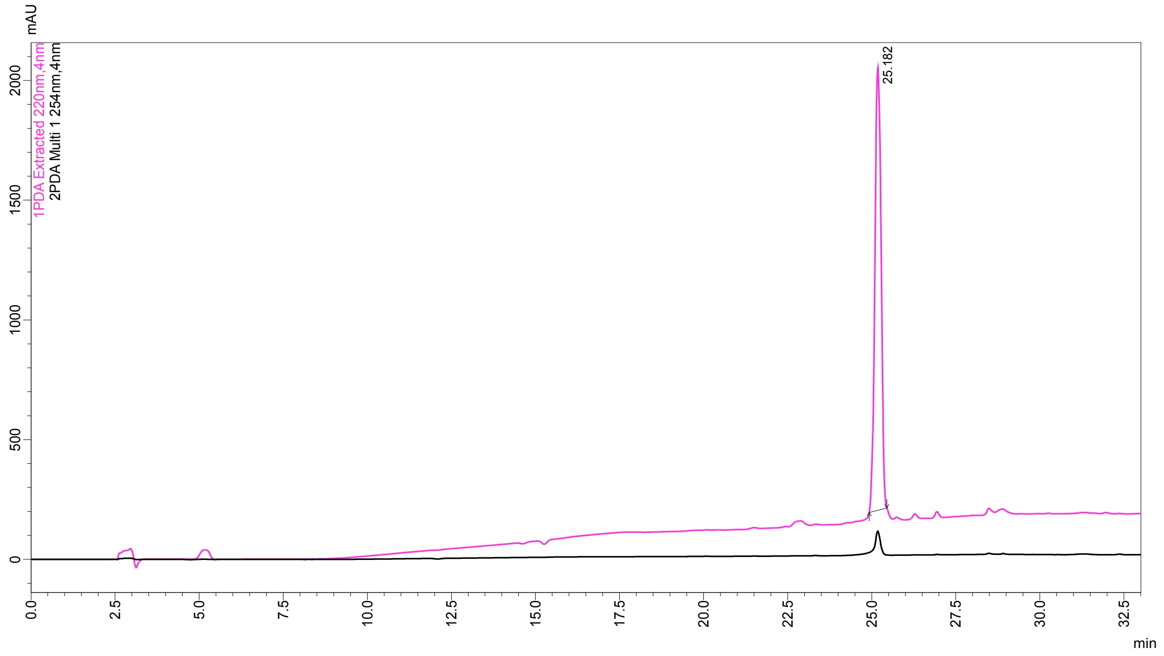

(B)

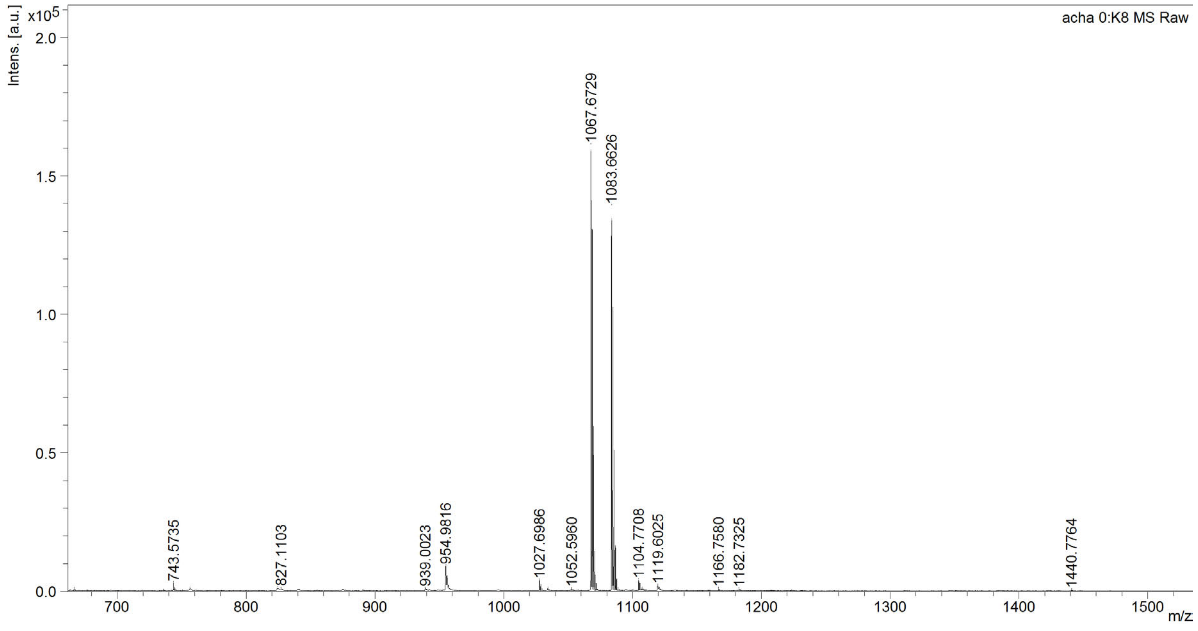

Figure S7.1: A) Analytical HPLC chromatogram of purified compound **7** at 70-100% MeOH/H_2O gradient and (B) the respective MALDI profile of the pure compound. Calculated MW: 1067.6623 [M+Na]; Observed MW: 1067.6729.

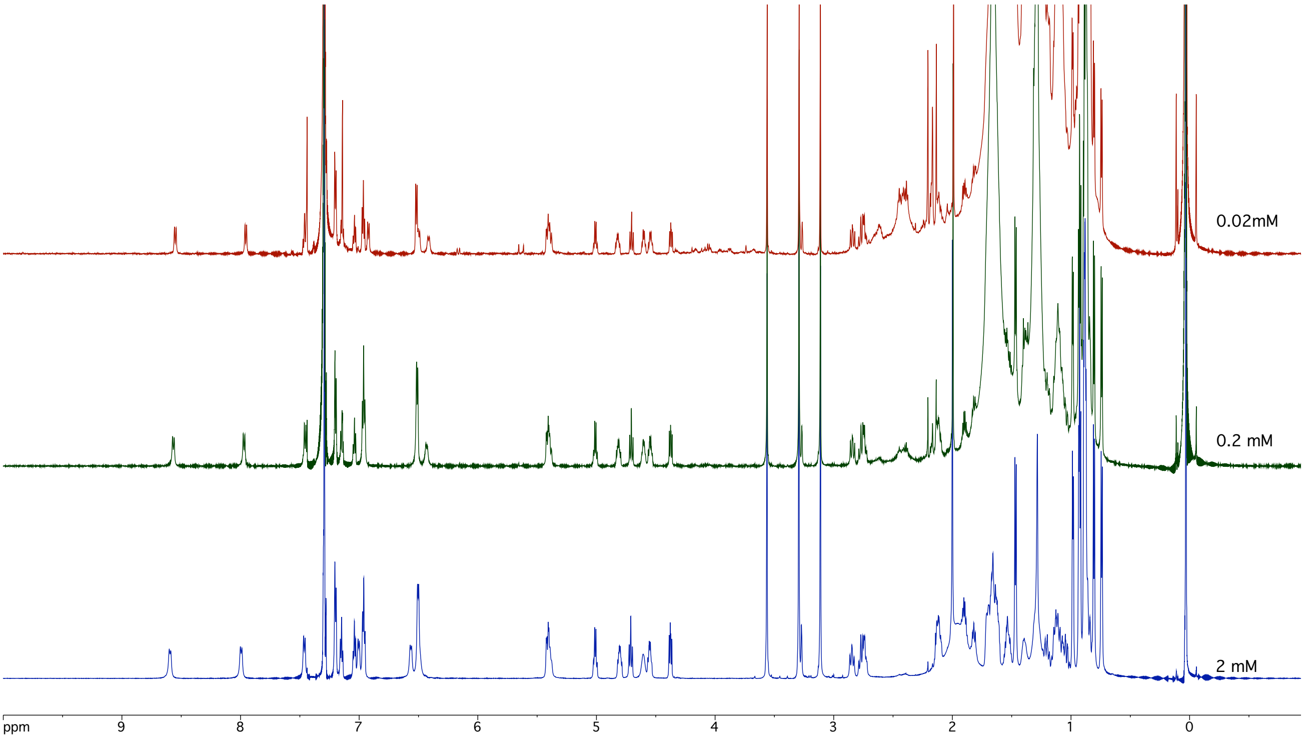

Figure S7.2: ^{1}H NMR spectra of Compound **7** at three different dilutions in $CDCl_3$ at 25°C.

Table 7.1: Chemical shifts table.

Residue	Atoms										
	HN	NMe	HA	HB		HG		HD		OMe	NAc
				1	2	1	2	1	2		
LEU1	6.98		4.59	1.51	1.36			0.88			1.97
PHE2	6.49		5.37	2.82	3.25						
VAL3	8.57		4.71	2.1		0.9	0.86				
D-ALA4		3.27	4.98	1.44							
Cha5		3.08	5.39	2.09	1.6						
LEU6	7.44		4.52	1.79	1.64			0.9	0.95		
PHE7	6.55		4.78	2.72							
VAL8	7.97		4.35	1.87		0.78	0.72			3.53	

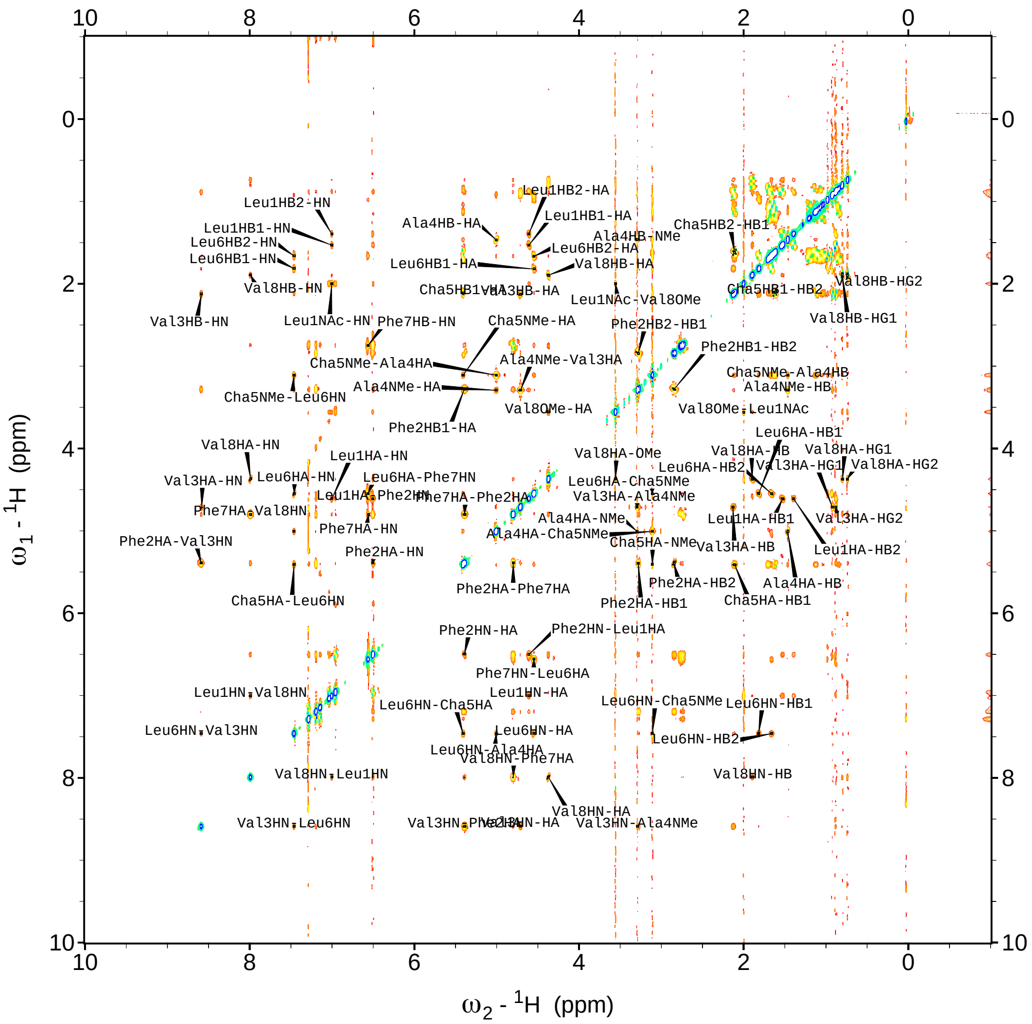

Figure S7.3: ROESY spectra with assigned peaks.

Table7.2: List of ROEs with respective NMR distances and violations.

Interactions	NMR Distance	Lower Limit	Upper Limit	Observed Distance	Violations
**Leu1HA-HN**	3.45	3.1	3.79	2.9	-0.2
**Leu1HA-Phe2HN**	2.89	2.6	3.17	2.39	-0.2
**Leu1HB1-HA**	2.95	2.66	3.25	2.55	-0.1
**Leu1HB1-HN**	3.73	3.36	4.1	3.39	0
**Leu1HB2-HA**	3.20	2.88	3.52	2.98	0
**Leu1HB2-HN**	3.69	3.32	4.06	3.01	-0.3
***Leu1HN-Val8HN**	3.92	3.53	4.32	2.78	-0.8
**Leu1NAc-HN**	2.99	2.69	3.69	2.72	0
**Leu1NAc-Val8OMe**	3.18	2.86	4.29	3.8	0
**Phe2HA-HB2**	2.93	2.63	3.22	2.39	-0.2
**Phe2HA-HN**	3.16	2.84	3.47	2.93	0
**Phe2HA-Val3HN**	2.60	2.34	2.86	2.09	-0.3

**Phe2HB1-HA**	2.47	2.22	2.71	2.6	0
**Phe2HB2-HB1**	1.80	1.62	1.98	1.73	0
**Val3HA-HG1**	2.83	2.55	3.12	2.96	0
**Val3HA-HG2**	2.78	2.5	3.06	3.46	0.4
**Val3HA-HN**	3.33	2.99	3.66	2.95	0
**Val3HB-HA**	2.87	2.58	3.16	2.84	0
**Val3HB-HN**	3.51	3.16	3.86	3.2	0
**Val3HN-ala4NMe**	3.68	3.31	4.05	4.24	0.2
**ala4HA-Leu6HN**	3.54	3.19	3.9	3.64	0
**ala4HB-HA**	2.39	2.15	3.03	2.47	0
**ala4HB-NMe**	2.40	2.16	3.44	3.31	0
**ala4NMe-Val3HA**	2.07	1.86	2.68	2.63	0
**ala4NMe-HA**	3.09	2.78	3.8	3.64	0
**Cha5HA-Leu6HN**	3.15	2.83	3.46	2.78	-0.1
**Cha5HB1-HA**	2.57	2.31	2.82	2.4	0
**Cha5HB2-HB1**	2.21	1.99	2.43	1.72	-0.3
**Cha5NMe-ala4HA**	2.08	1.87	2.68	2.54	0
**Cha5NMe-ala4HB**	3.15	2.83	4.26	4.04	0
**Cha5NMe-HA**	3.28	2.95	4.01	3.8	0
**Cha5NMe-Leu6HN**	2.92	2.63	3.61	3.54	0
**Leu6HA-HN**	3.04	2.74	3.35	2.92	0
**Leu6HA-Phe7HN**	2.35	2.11	2.58	2.26	0
**Leu6HB1-HA**	2.83	2.55	3.11	2.69	0
**Leu6HB1-HN**	3.17	2.85	3.49	3.02	0
**Leu6HB2-HA**	2.64	2.38	2.91	2.88	0
**Leu6HB2-HN**	3.23	2.91	3.55	3.01	0
**Leu6HN-Val3HN**	4.06	3.66	4.47	3.34	-0.3
**Phe7HA-Phe2HA**	2.38	2.14	2.62	2.79	0.2
**Phe7HA-HN**	3.11	2.8	3.42	2.89	0
**Phe7HA-Val8HN**	2.43	2.19	2.68	2.43	0
**Val8HA-HG1**	2.95	2.66	3.25	2.99	0
**Val8HA-HG2**	3.10	2.79	3.41	3.49	0.1
**Val8HA-HN**	3.14	2.83	3.46	2.87	0
**Val8HB-HA**	2.78	2.5	3.05	2.82	0
**Val8HB-HG1**	2.99	2.69	3.29	2.47	-0.2
**Val8HB-HG2**	3.00	2.7	3.29	2.49	-0.2
**Val8HB-HN**	3.80	3.42	4.18	3.26	-0.2
**Val8HN-Phe2HA**	3.84	3.46	4.23	4.46	0.2
***Val8OMe-HA**	3.63	3.01	4.07	4.54	0.5

* violations ≥0.5. The observed high violations can be explained by the local flexibility about the γ and δ methyl groups (Val and Leu respectively) and the terminal ester bond, peak overlap, additional *J*-mediated transfer and inaccuracies in the force fields.[9]

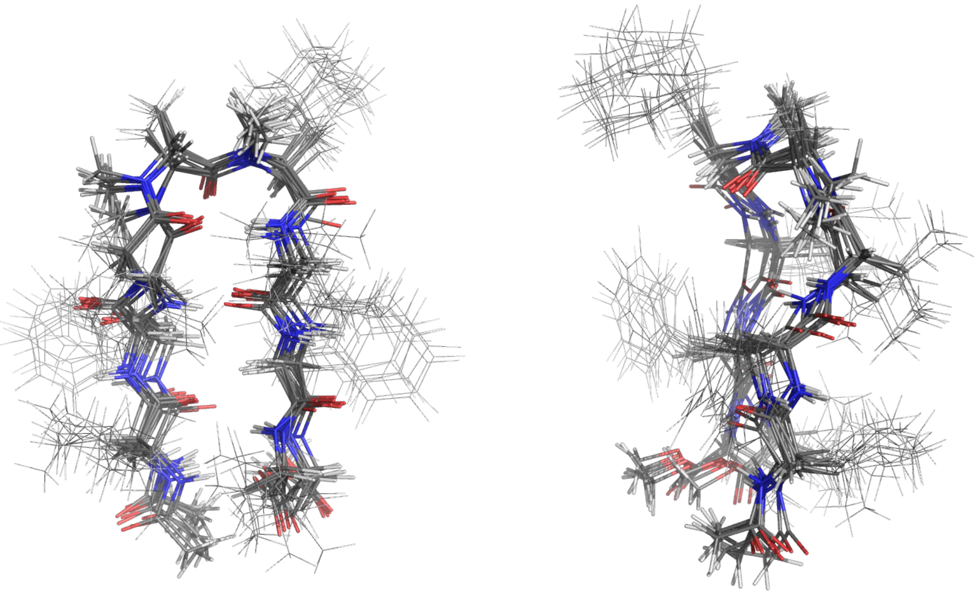

Figure S7.4: Overlay of 10 representative conformations generated using Molecular Dynamics simulation, showing both front view (left panel) and side view (right panel).

(A)

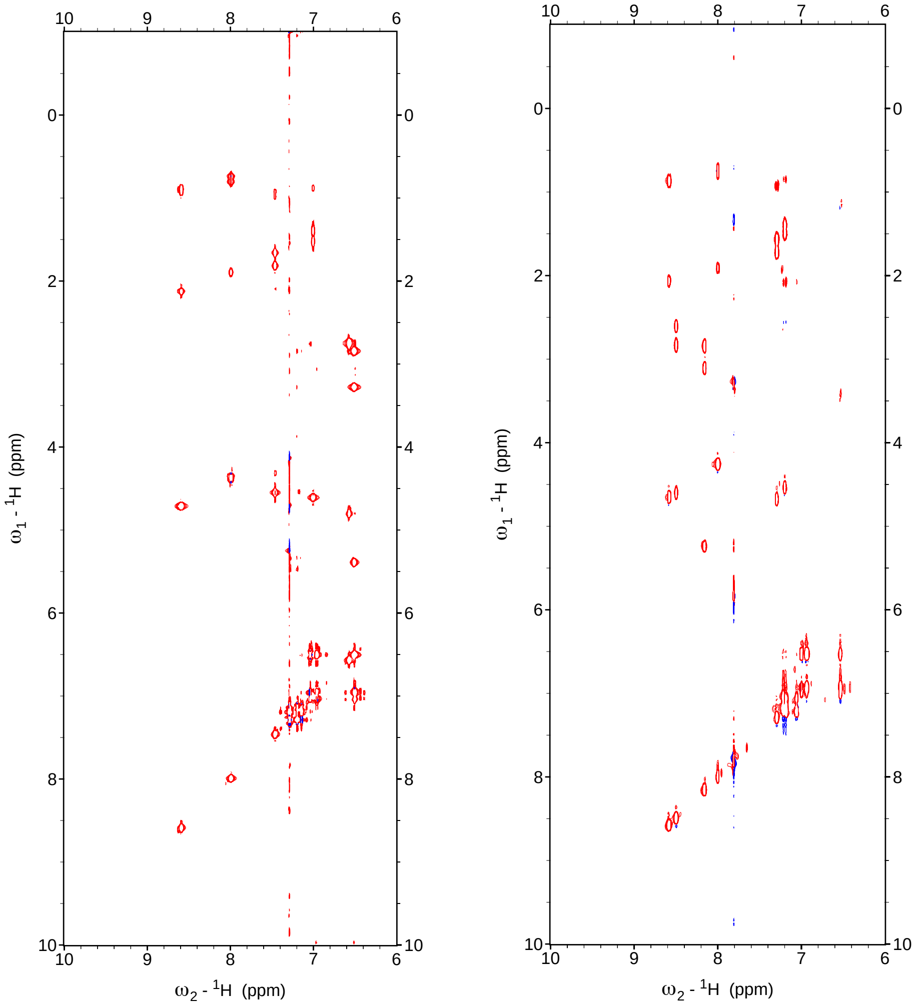

$CDCl_3$ DMSO-d_6 – $CDCl_3$ (1:2)

(B)

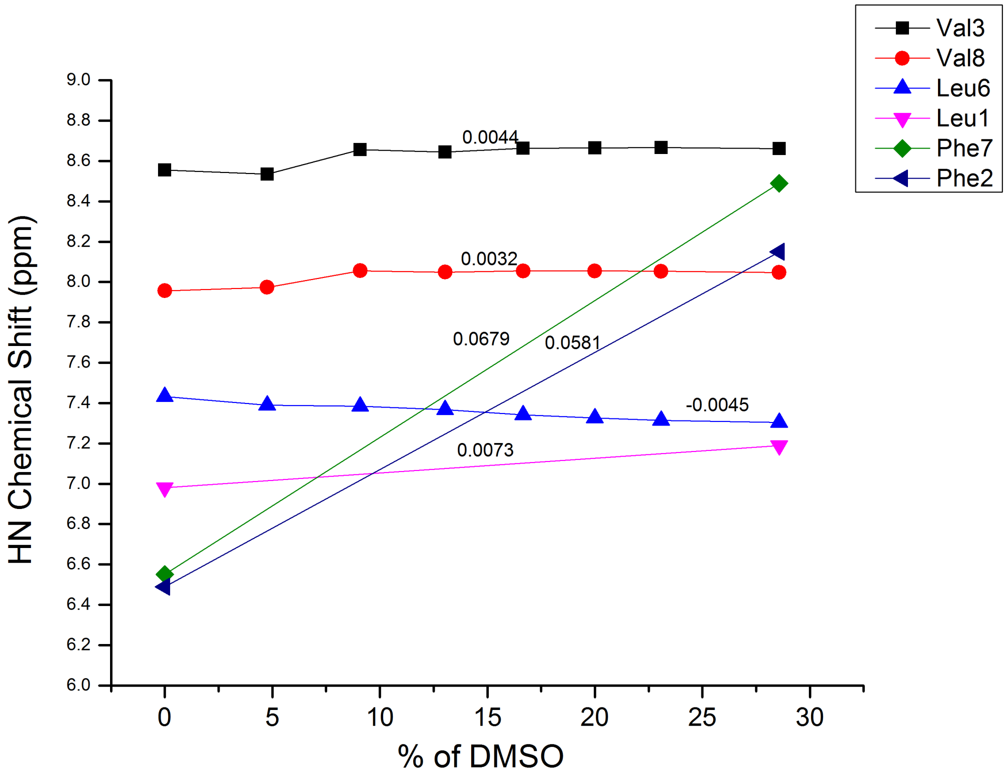

Figure S7.5: (A) TOCSY spectra in $CDCl_3$ and DMSO-d_6 – $CDCl_3$ (1:2) and (B) DMSO-d_6 titration curve indicating the solvent exposed (F2, F7) and solvent shielded (L1, V3, L6, V8) amide protons. The value indicates the slope generated by the linear fit of the data points.

Compound 8:

(A)

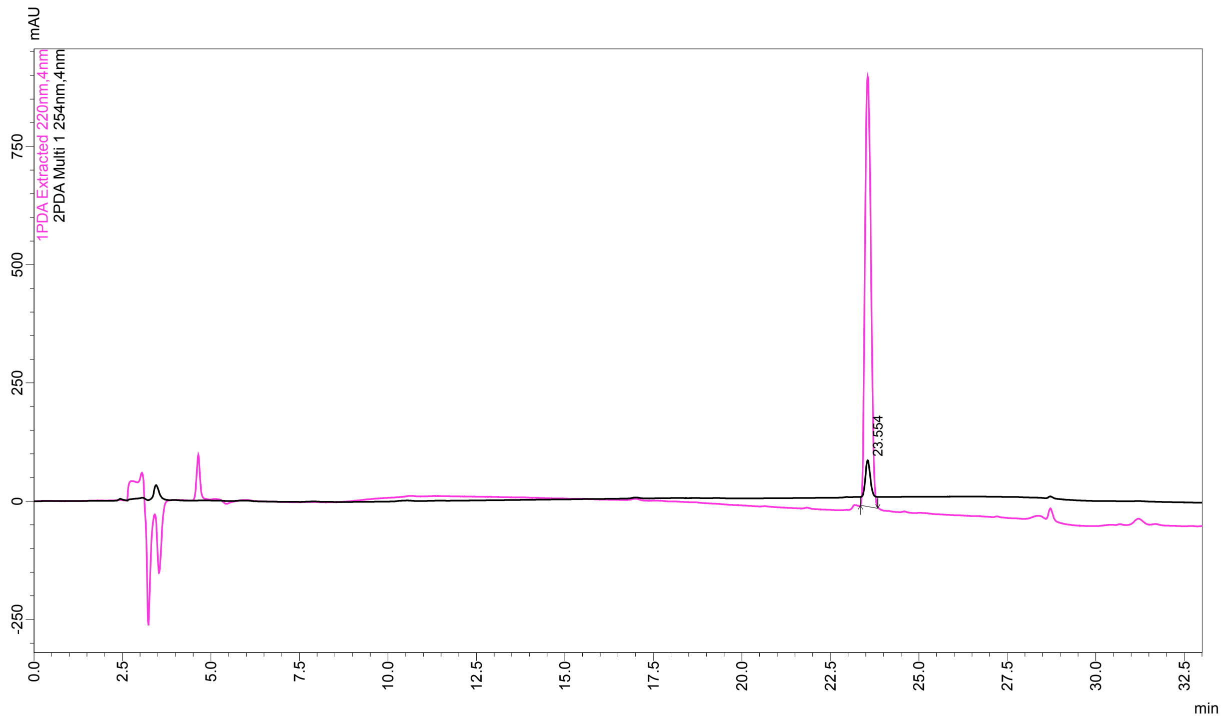

(B)

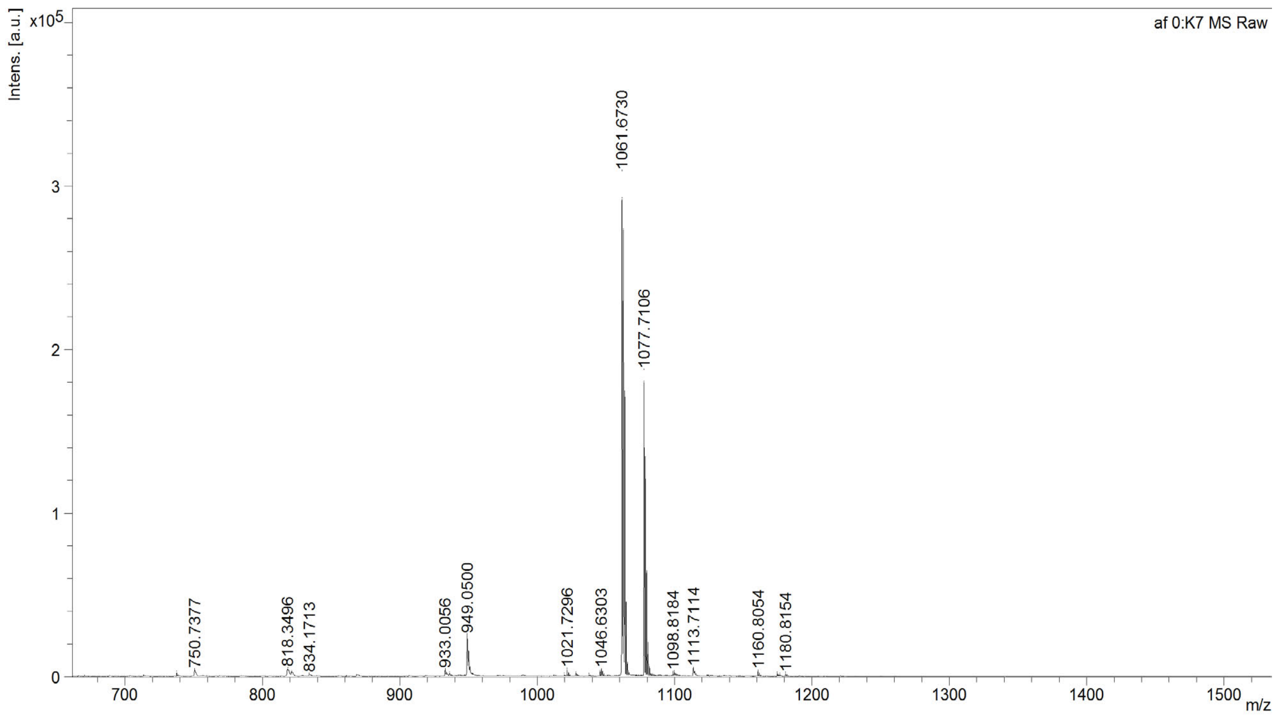

Figure S8.1: A) Analytical HPLC chromatogram of purified compound **8** at 70-100% MeOH/H_2O gradient and (B) the respective MALDI profile of the pure compound. Calculated MW: 1061.6154 [M+Na]; Observed MW: 1061.6730.

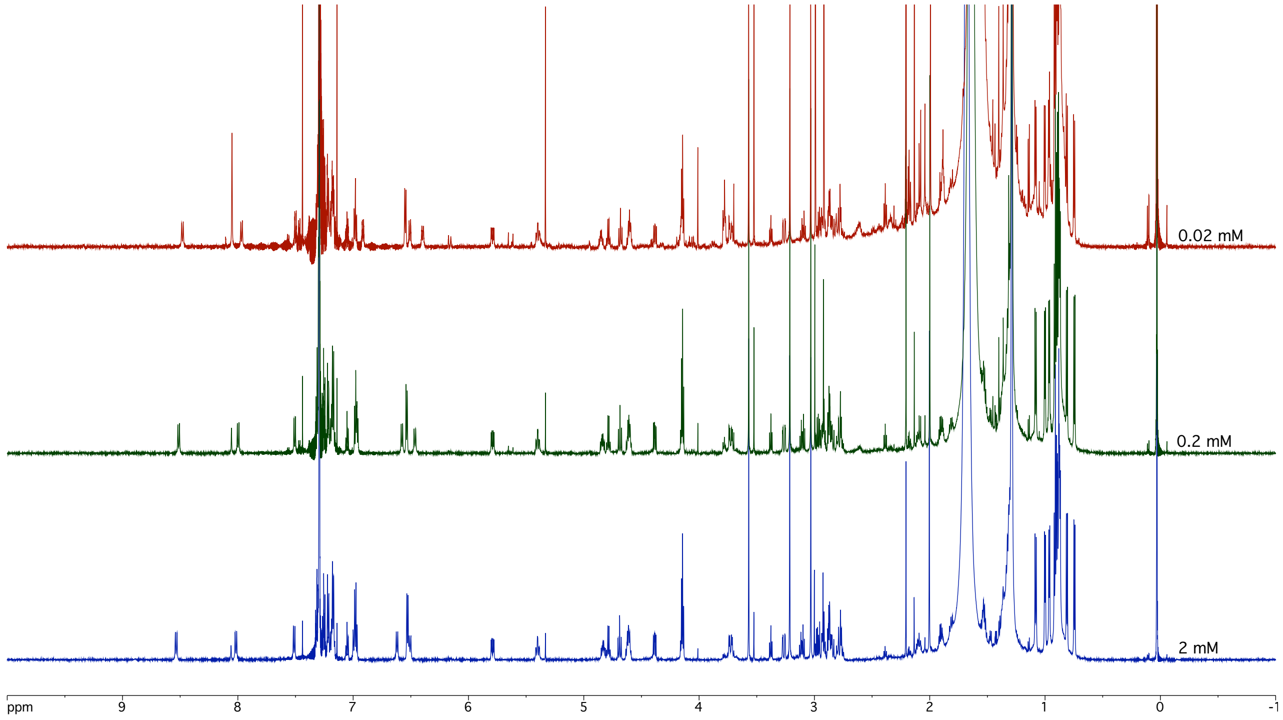

Figure S8.2: ^{1}H NMR spectra of Compound **7** at three different dilutions in $CDCl_3$ at 25°C.

Table 8.1: Chemical shifts table.

Residue	Atoms										
	HN	NMe	HA	HB		HG		HD		OMe	NAc
				1	2	1	2	1	2		
**LEU1**	7.07		4.6	1.51	1.46						1.98
**PHE2**	6.59		5.39	3.26	2.85						
**VAL3**	8.58		4.69	2.09		0.9	0.86				
**D-ALA4**		3.2	4.78	1.07							
**PHE5**		3.02	5.78	3.72	2.93						
**LEU6**	7.51		4.62	1.8	1.67			0.99	0.95		
**PHE7**	6.69		4.81	2.77							
**VAL8**	8.05		4.38	1.9		0.8	0.74			3.54	

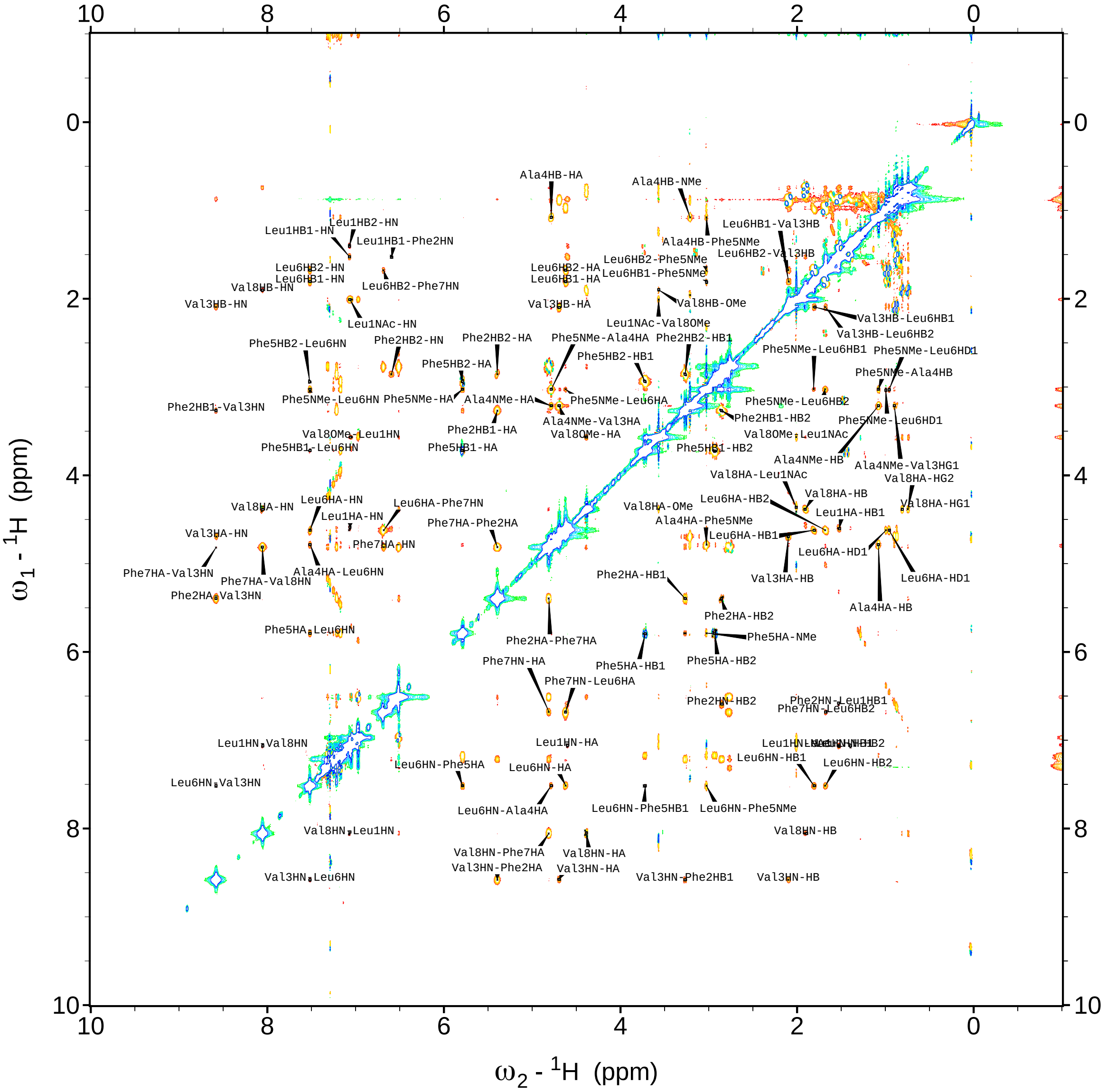

Figure S8.3: ROESY spectra with assigned peaks.

Table8.2: List of ROEs with respective NMR distances and violations.

Interactions	NMR Distance	Lower Limit	Upper Limit	Observed Distance	Violations
***Leu1HA-HB1**	3.28	2.95	3.61	2.49	-0.5
**Leu1HA-HN**	3.23	2.91	3.55	2.87	0
**Leu1HB1-HN**	3.56	3.2	3.92	3.47	0
***Leu1HB1-Phe2HN**	3.18	2.86	3.5	3.98	0.5
**Leu1HB2-HN**	3.45	3.11	3.8	3.06	0
***Leu1HN-Val8HN**	3.5	3.15	3.85	2.5	-0.6
**Leu1NAc-HN**	2.77	2.49	3.45	2.71	0
**Leu1NAc-**	3.18	2.86	4.3	3.88	0

**Val8OMe**					
**Phe2HA-Val3HN**	2.76	2.48	3.04	2.11	-0.4
**Phe2HB1-HA**	2.64	2.38	2.9	2.72	0
**Phe2HB1-Val3HN**	3.62	3.26	3.98	4.01	0
**Phe2HB2-HA**	3.18	2.86	3.5	2.77	-0.1
**Phe2HB2-HB1**	1.87	1.68	2.06	1.74	0
**Phe2HB2-HN**	3.31	2.98	3.64	3.15	0
**Val3HA-HN**	3.54	3.19	3.89	2.98	-0.2
**Val3HB-HA**	2.89	2.6	3.18	3.04	0
**Val3HB-HN**	3.45	3.11	3.8	2.96	-0.1
**ala4HA-Leu6HN**	3.56	3.2	3.92	3.58	0
**ala4HB-HA**	2.44	2.2	3.08	2.45	0
**ala4NMe-Val3HA**	2.08	1.87	2.69	2.72	0
**ala4HB-NMe**	2.36	2.12	3.4	3.34	0
**ala4NMe-HA**	3.06	2.75	3.77	3.69	0
**ala4HB-Phe5NMe**	3.03	2.73	4.13	4.29	0.2
**Phe5HA-Leu6HN**	3.28	2.95	3.61	2.92	0
**Phe5HB1-HA**	2.38	2.14	2.62	2.81	0.2
**Phe5HB2-HA**	2.55	2.3	2.81	2.71	0
**Phe5HB2-HB1**	1.8	1.62	1.98	1.73	0
**Phe5HB2-Leu6HN**	4.64	4.18	5.1	4.35	0
**Phe5NMe-ala4HA**	2.09	1.88	2.7	2.56	0
**Phe5NMe-HA**	3.37	3.03	4.11	3.8	0
***Phe5NMe-Leu6HA**	3.62	3.26	4.38	5	0.6
**Phe5HB1-Leu6HN**	3.83	3.45	4.21	4.35	0.1
**Phe5NMe-Leu6HD1**	3.91	3.52	5.1	4.64	0
**Phe5NMe-Leu6HD2**	4.08	3.67	5.29	5.02	0
**Phe5NMe-Leu6HN**	2.95	2.66	3.65	3.26	0
**Leu6HA-HD2**	2.89	2.6	3.18	2.87	0
**Leu6HA-HN**	3.04	2.74	3.34	2.92	0
**Leu6HB1-Val3HB**	3.04	2.74	3.34	2.65	-0.1
**Leu6HB1-HA**	2.82	2.54	3.1	3.06	0
**Leu6HB1-HN**	3.21	2.89	3.53	2.67	-0.2
**Leu6HB2-Val3HB**	3.35	3.02	3.69	3.66	0
**Leu6HB2-HA**	2.61	2.35	2.87	2.54	0
**Leu6HB2-HN**	3.23	2.91	3.55	3.74	0.2
***Leu6HB2-Phe7HN**	3.71	3.34	4.08	2.88	-0.5
***Leu6HN-Val3HN**	4.18	3.76	4.6	3.31	-0.5
**Phe7HA-Phe2HA**	2.5	2.25	2.75	2.59	0
**Phe7HA-HN**	3.12	2.81	3.43	2.9	0

**Phe7HA-Val8HN**	2.47	2.22	2.72	2.26	0
**Val8HA-HB**	2.7	2.43	2.97	2.91	0
**Val8HA-HG1**	3.01	2.71	3.31	2.91	0
**Val8HA-HG2**	3.12	2.81	3.43	3.47	0
**Val8HA-HN**	3.36	3.02	3.7	2.87	-0.2
**Val8HB-HN**	3.56	3.2	3.92	3.2	0
**Val8OMe-Leu1HN**	3.78	3.4	4.56	3.31	-0.1
**Val8OMe-HA**	3.52	3.17	4.27	4.51	0.2

* violations ≥0.5. The observed high violations can be explained by the local flexibility about the γ and δ methyl groups (Val and Leu respectively) and the terminal ester bond, peak overlap, additional J-mediated transfer and inaccuracies in the force fields. [9]

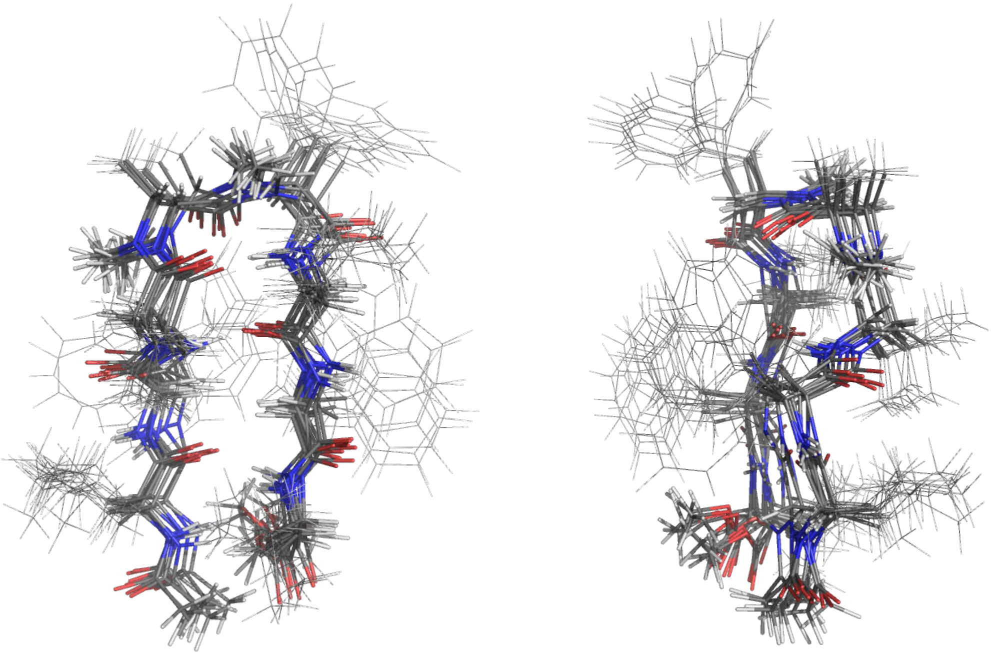

Figure S8.4: Overlay of 10 representative conformations generated using Molecular Dynamics simulation, showing both front view (left panel) and side view (right panel).

(A)

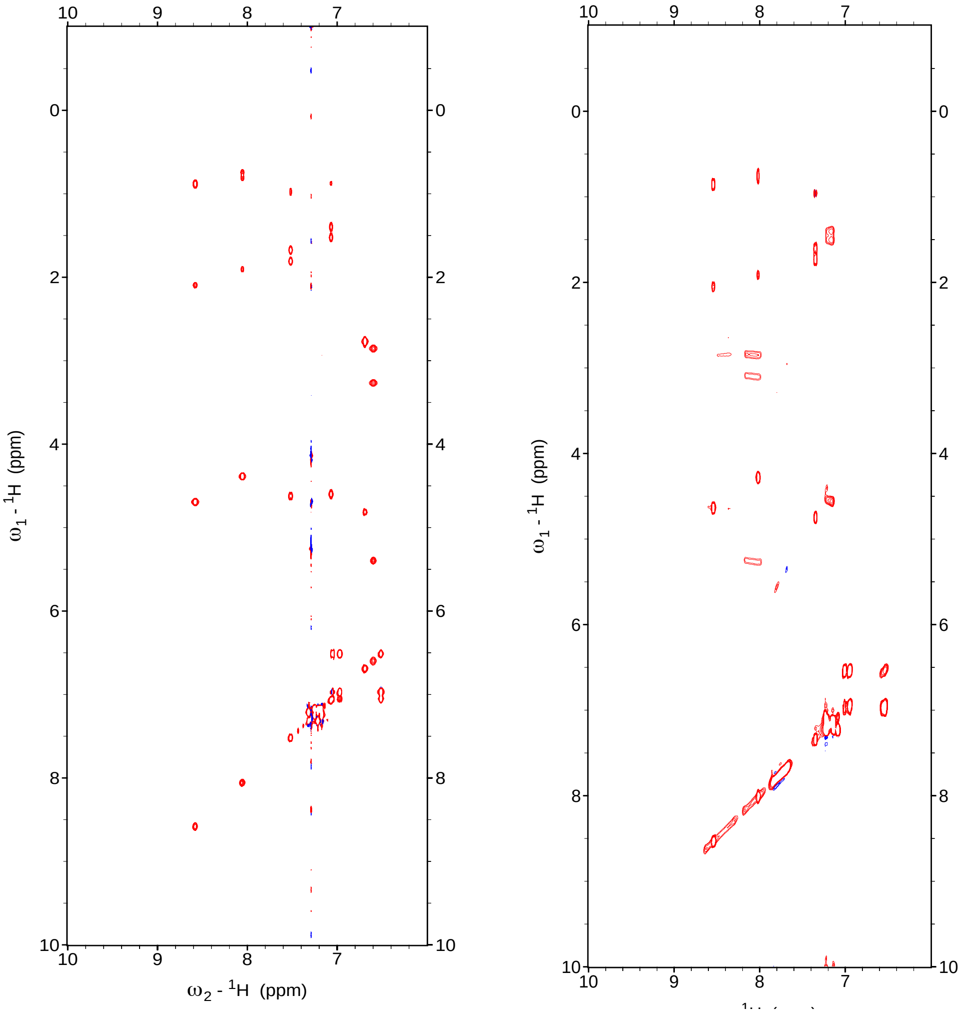

$CDCl_3$

DMSO-d_6 – $CDCl_3$
(1:2)

(B)

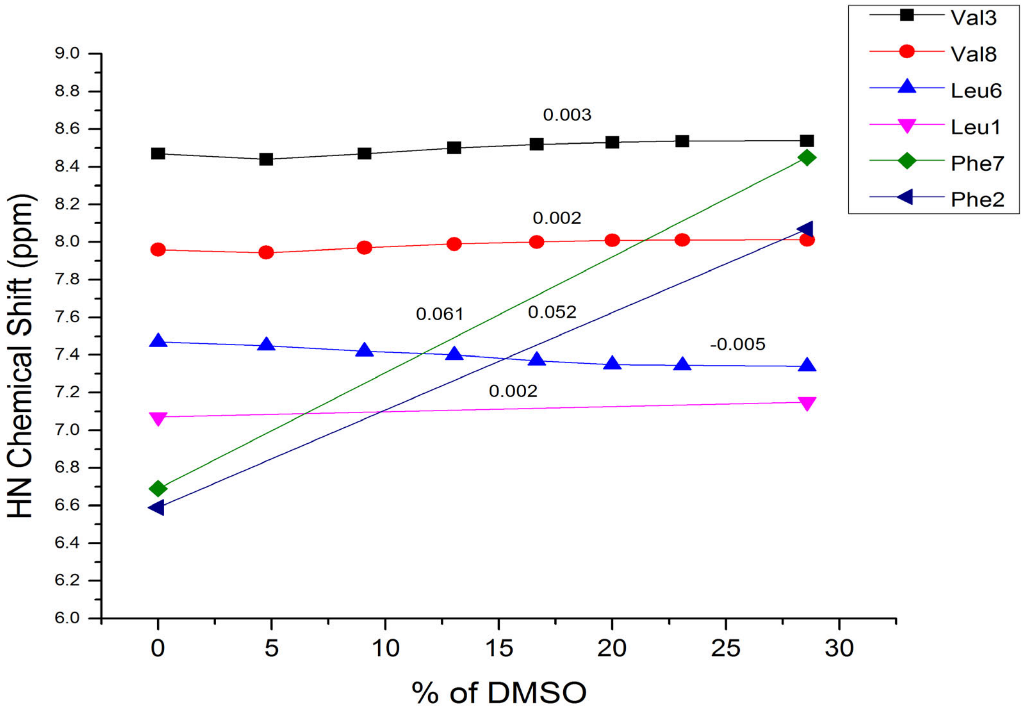

Figure S8.5: (A) TOCSY spectra in $CDCl_3$ and DMSO-d_6 – $CDCl_3$ (1:2) and (B) DMSO-d_6 titration curve indicating the solvent exposed (F2, F7) and solvent shielded (L1, V3, L6, V8) amide protons. The value indicates the slope generated by the linear fit of the data points.

Compound 9:

(A)

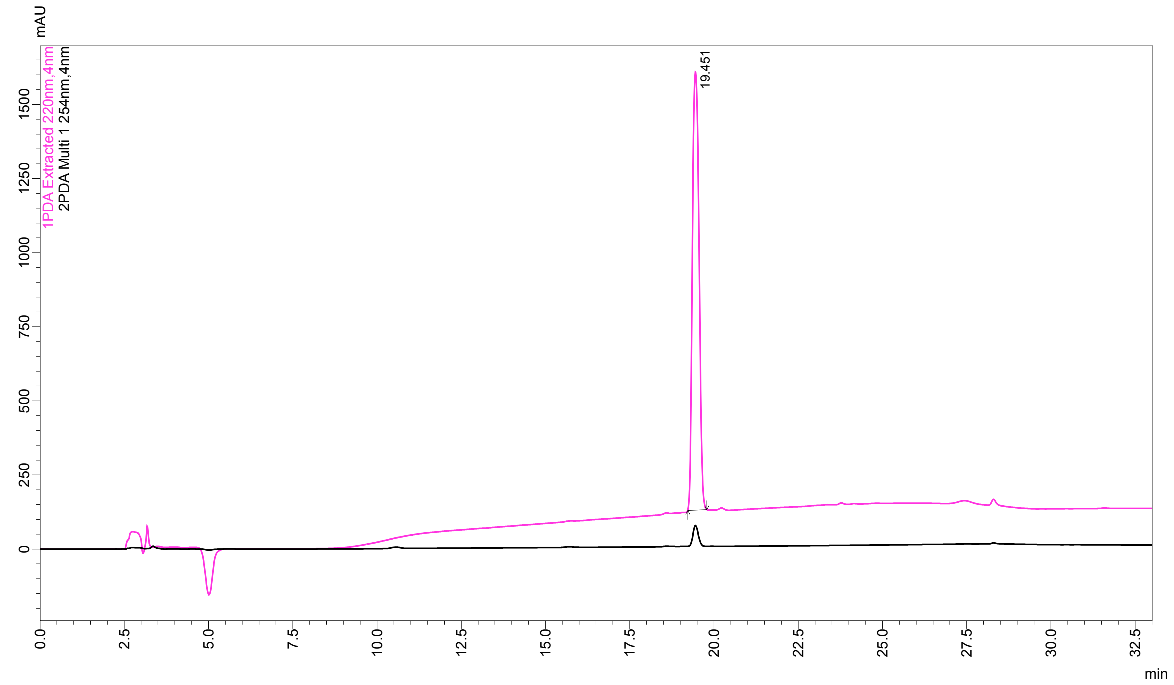

(B)

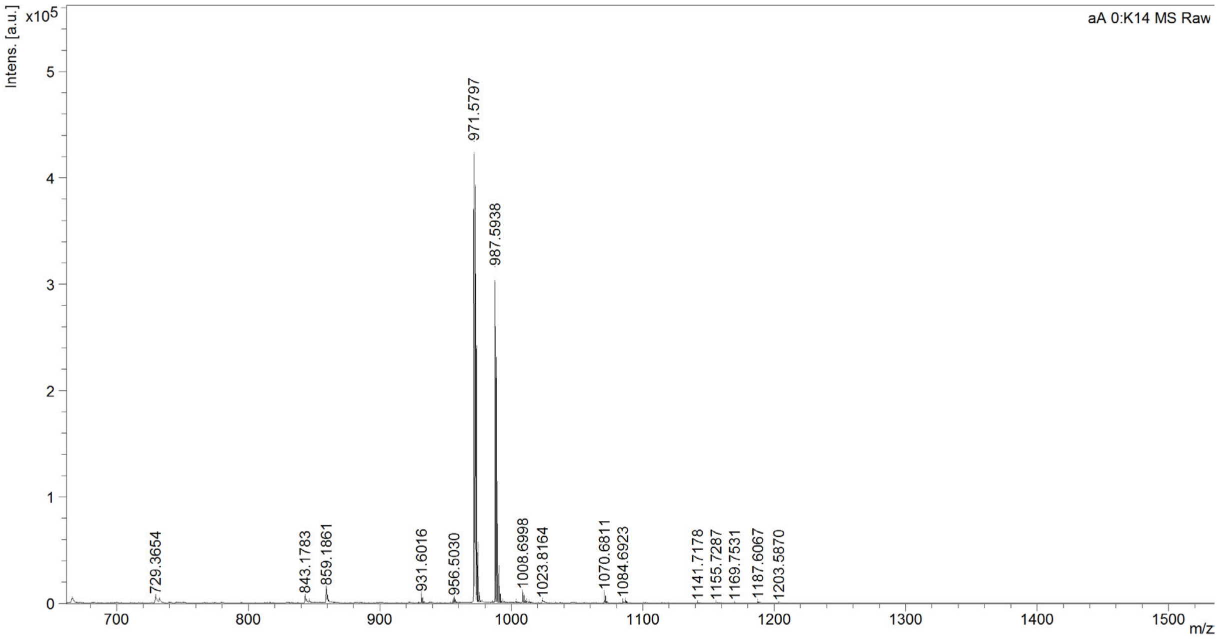

Figure S9.1: A) Analytical HPLC chromatogram of purified compound **9** at 70-100% MeOH/H_2O gradient and (B) the respective MALDI profile of the pure compound. Calculated MW: 971.5684 [M+Na]; Observed MW: 971.5797.

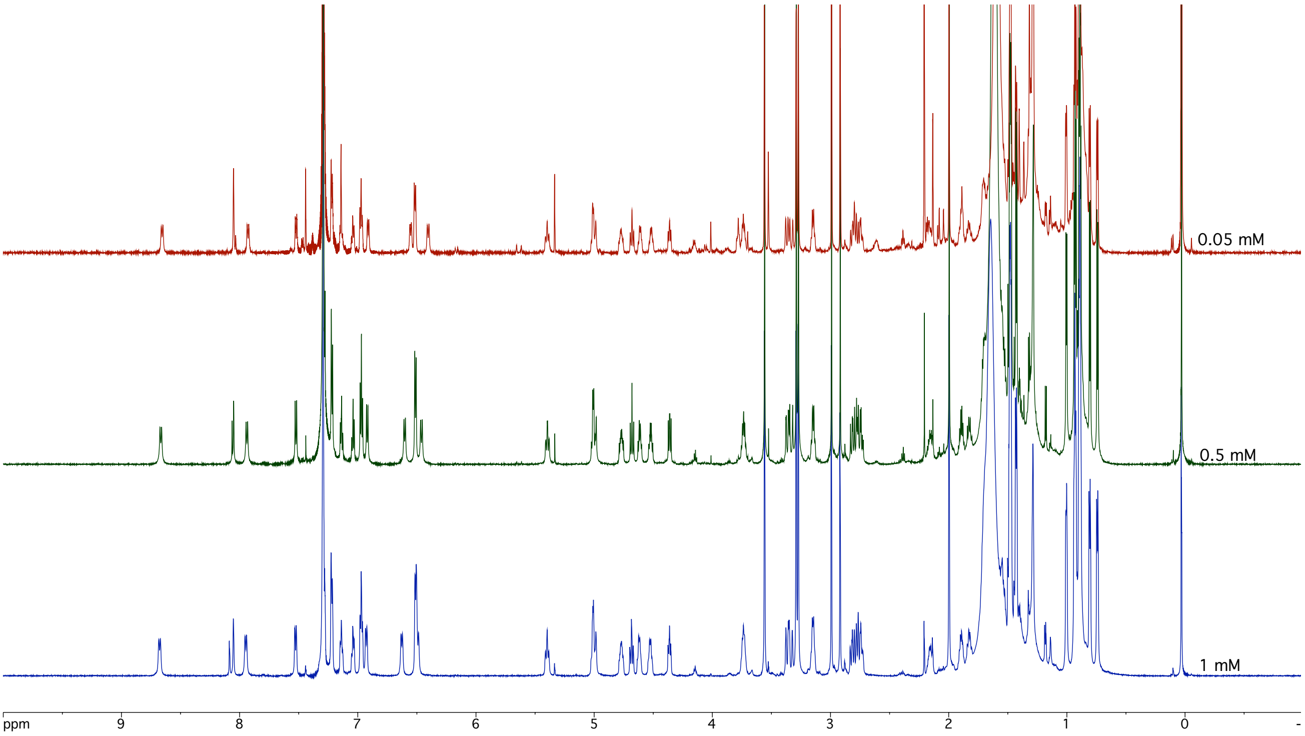

Figure S9.2: ^{1}H NMR spectra of Compound **9** at three different dilutions in $CDCl_3$ at 25°C.

Table 9.1: Chemical shifts table.

Residue	Atoms										
	HN	NMe	HA	HB		HG		HD		OMe	NAc
				1	2	1	2	1	2		
**LEU1**	6.92		4.58	1.51	1.37			0.97	0.91		1.97
**PHE2**	6.42		5.37	2.79	3.34						
**VAL3**	8.64		4.65	2.13		0.88					
**D-ALA4**		3.24	4.98	1.45							
**Gly5**		3.26	3.32								
**LEU6**	7.51		4.49	1.68	1.8			0.86			
**PHE7**	6.57		4.75	2.75							
**VAL8**	7.91		4.34	1.87		0.78	0.71			3.53	

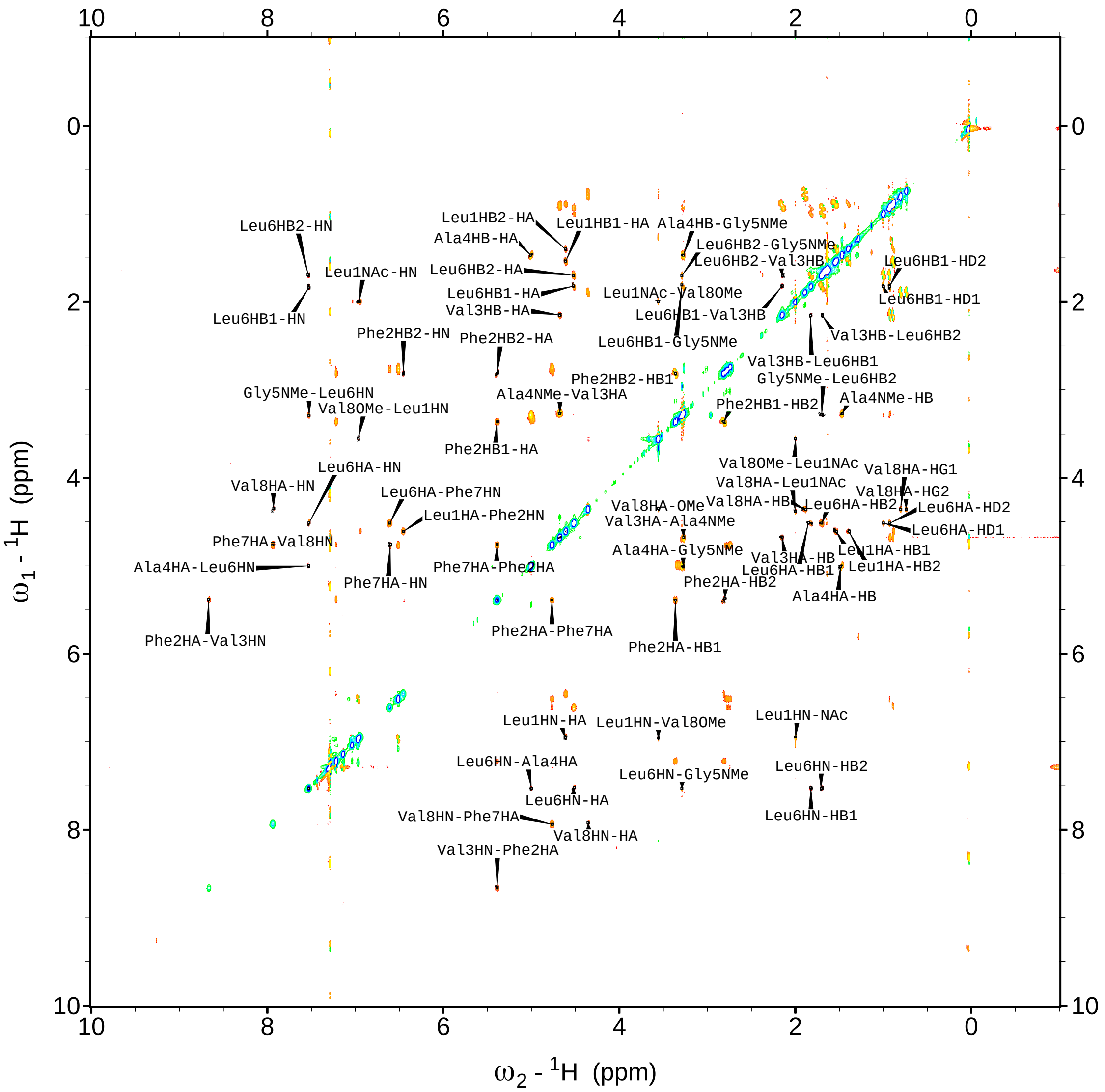

Figure S9.3: ROESY spectra with assigned peaks.

Table9.2: List of ROEs with respective NMR distances and violations.

Interactions	NMR Distance	Lower Limit	Upper Limit	Observed Distance	Violations
**Leu1HB1-HA**	2.54	2.29	2.79	2.57	0
**Leu1HB2-HA**	2.78	2.50	3.06	3.06	0
**Leu1HN-HA**	3.04	2.74	3.34	2.87	0
**Leu1NAc-HN**	2.61	2.35	3.27	2.77	0
**Leu1HA-Phe2HN**	2.53	2.28	2.78	2.24	0
**Phe2HA-Val3HN**	2.68	2.41	2.95	2.10	-0.3
**Phe2HB1-HA**	2.50	2.25	2.75	2.57	0
**Phe2HB2-HA**	2.62	2.36	2.88	2.41	0
**Phe2HB2-HB1**	1.80	1.62	1.98	1.74	0

**Phe2HB2-HN**	2.67	2.40	2.94	3.14	0.2
**Val3HB-HA**	2.62	2.36	2.88	3.03	0.1
**Val3HA-ala4NMe**	2.15	1.94	2.76	2.79	0
**ala4HA-Leu6HN**	3.12	2.81	3.43	3.60	0.2
**ala4HB-HA**	2.14	1.93	2.75	2.47	0
**Gly5NMe-Leu6HN**	2.85	2.57	3.54	3.52	0
**Leu6HA-HD1**	2.99	2.69	3.29	3.25	0
**Leu6HA-HN**	2.93	2.64	3.22	2.93	0
**Leu6HA-Phe7HN**	2.32	2.09	2.55	2.39	0
**Leu6HB1-Val3HB**	2.66	2.42	2.96	3.11	0.1
**Leu6HB1-HA**	2.73	2.46	3.00	2.85	0
**Leu6HB1-HD1**	2.71	2.44	2.98	3.11	0.1
**Leu6HB1-HD2**	3.20	2.88	3.52	3.06	0
**Leu6HB1-HN**	2.87	2.58	3.16	2.71	0
**Leu6HB2-Val3HB**	2.88	2.60	3.18	2.51	-0.1
**Leu6HB2-HA**	2.45	2.21	2.70	2.87	0.2
**Leu6HB2-HN**	2.70	2.43	2.97	3.09	0.1
**Phe7HA-Phe2HA**	2.49	2.24	2.74	2.78	0
**Phe7HA-HN**	2.90	2.61	3.19	2.92	0
**Phe7HA-Val8HN**	2.46	2.21	2.71	2.31	0
**Val8HA-HB**	2.50	2.25	2.75	2.74	0
**Val8HA-HG1**	2.78	2.50	3.06	3.00	0
**Val8HA-HG2**	3.10	2.79	3.41	3.00	0
**Val8HA-HN**	2.85	2.57	3.14	2.87	0
**Leu1NAc-Val8OMe**	3.02	2.72	4.12	3.69	0
**Val8OMe-Leu1HN**	3.16	2.84	3.88	3.25	0

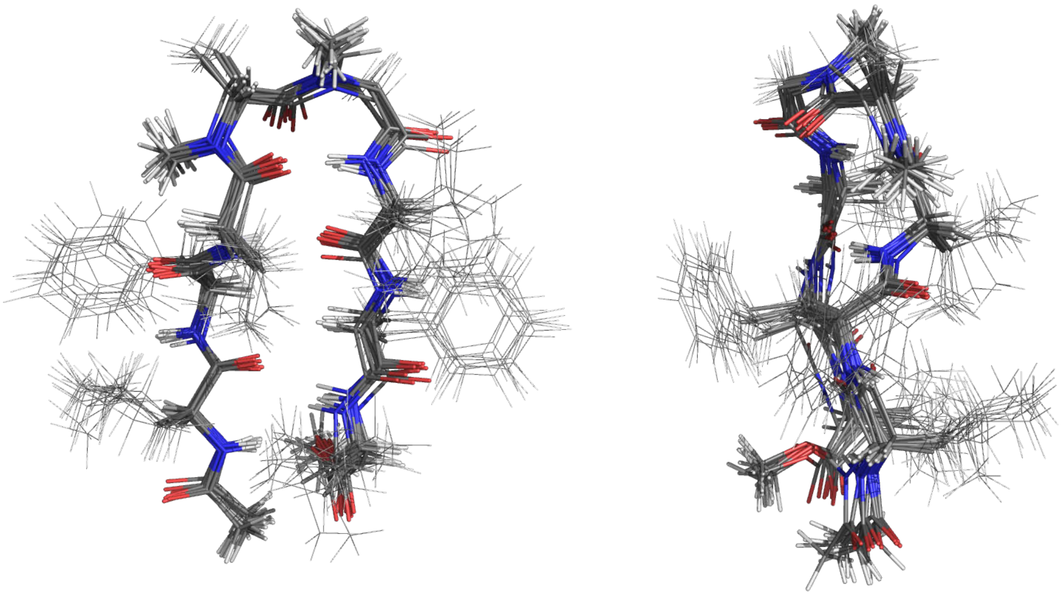

Figure S9.4: Overlay of 10 representative conformations generated using Molecular Dynamics simulation, showing both front view (left panel) and side view (right panel).

(A)

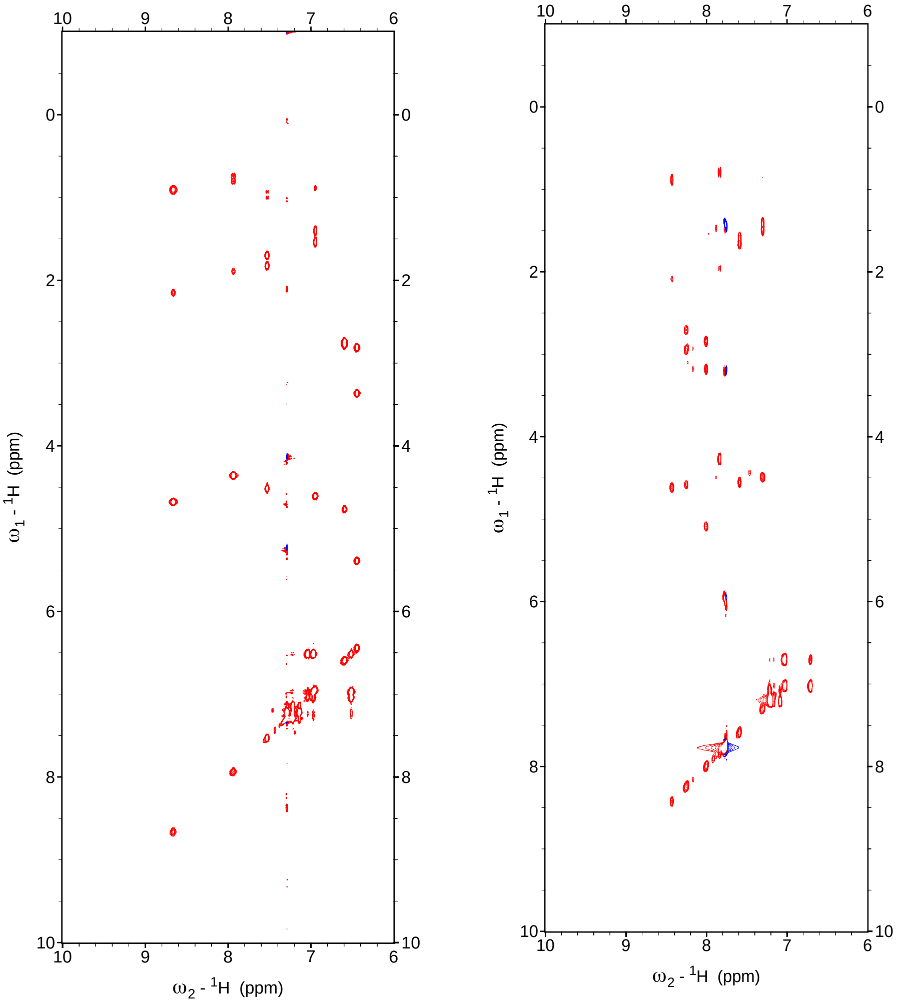

$CDCl_3$

DMSO-d_6 – $CDCl_3$ (1:2)

(B)

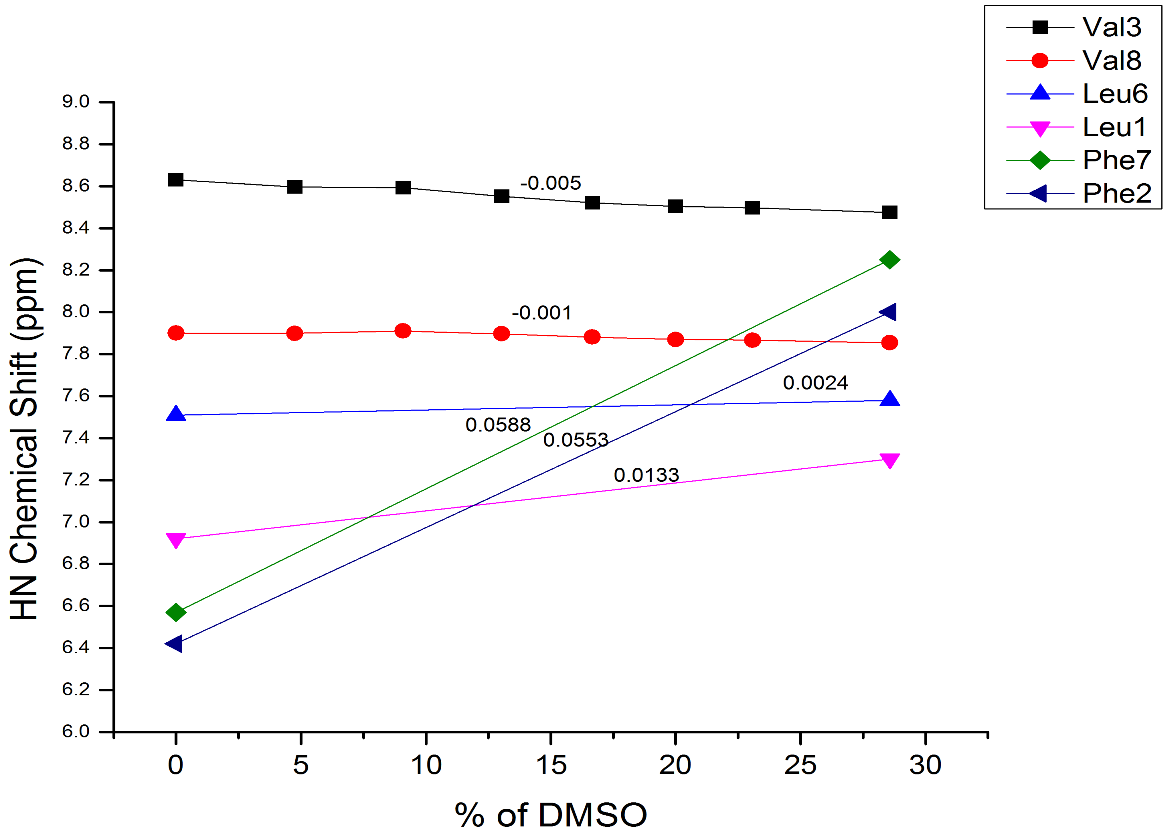

Figure S9.5: (A) TOCSY spectra in $CDCl_3$ and DMSO-d_6 – $CDCl_3$ (1:2) and (B) DMSO-d_6 titration curve indicating the solvent exposed (F2, F7) and solvent shielded (V3, L6, V8) amide protons. The value indicates the slope generated by the linear fit of the data points.

<u>Compound 10:</u>

(A)

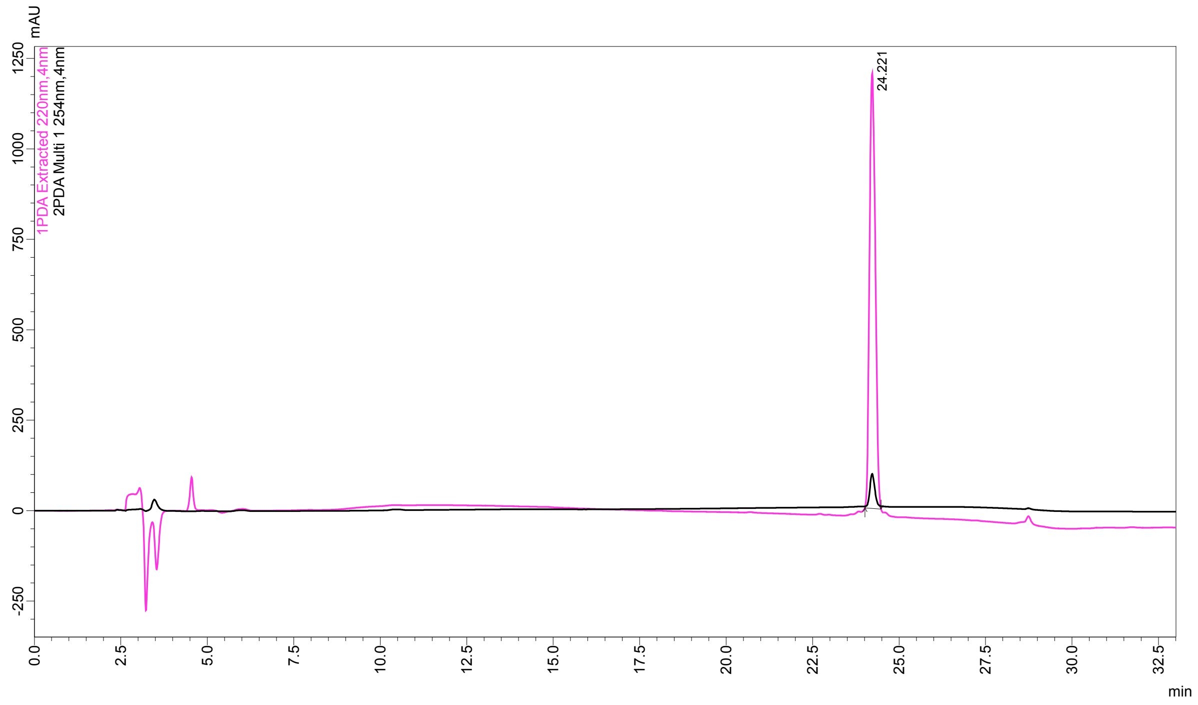

(B)

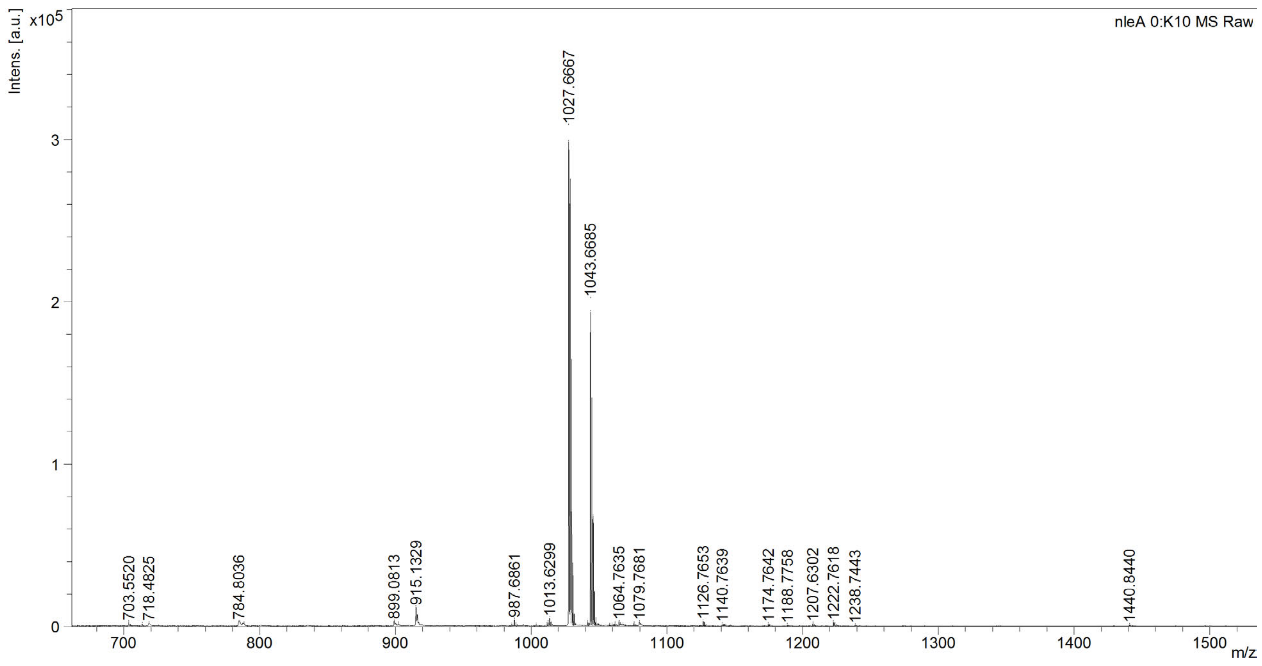

Figure S10.1: A) Analytical HPLC chromatogram of purified compound **10** at 70-100% MeOH/H_2O gradient and (B) the respective MALDI profile of the pure compound. Calculated MW: 1027.6310 [M+Na]; Observed MW: 1027.6667.

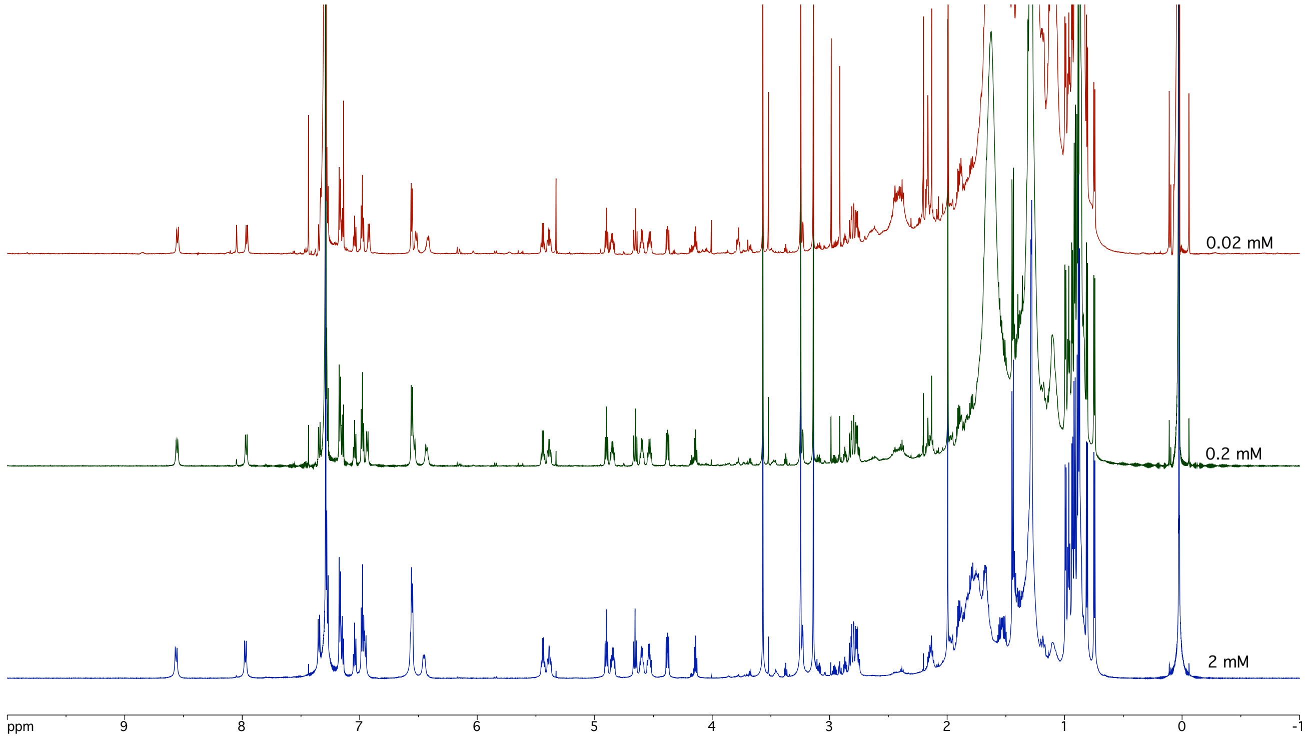

Figure S10.2: ^{1}H NMR spectra of Compound **10** at three different dilutions in $CDCl_3$ at 25°C.

Table 10.1: Chemical shifts table.

Residue	Atoms										
	HN	NMe	HA	HB		HG		HD		OMe	NAc
				1	2	1	2	1	2		
**LEU1**	6.92		4.59	1.52	1.39			0.87			1.97
**PHE2**	6.48		5.37	2.81	3.23						
**VAL3**	8.55		4.65	2.13		0.91	0.88				
**D-NLE4**		3.24	4.9	1.86		1.42	1.34				
**ALA5**		3.13	5.42	1.44							
**LEU6**	7.35		4.53	1.68	1.79			0.98	0.93		
**PHE7**	6.61		4.84	2.78							
**VAL8**	7.96		4.37	1.91		0.81	0.75			3.54	

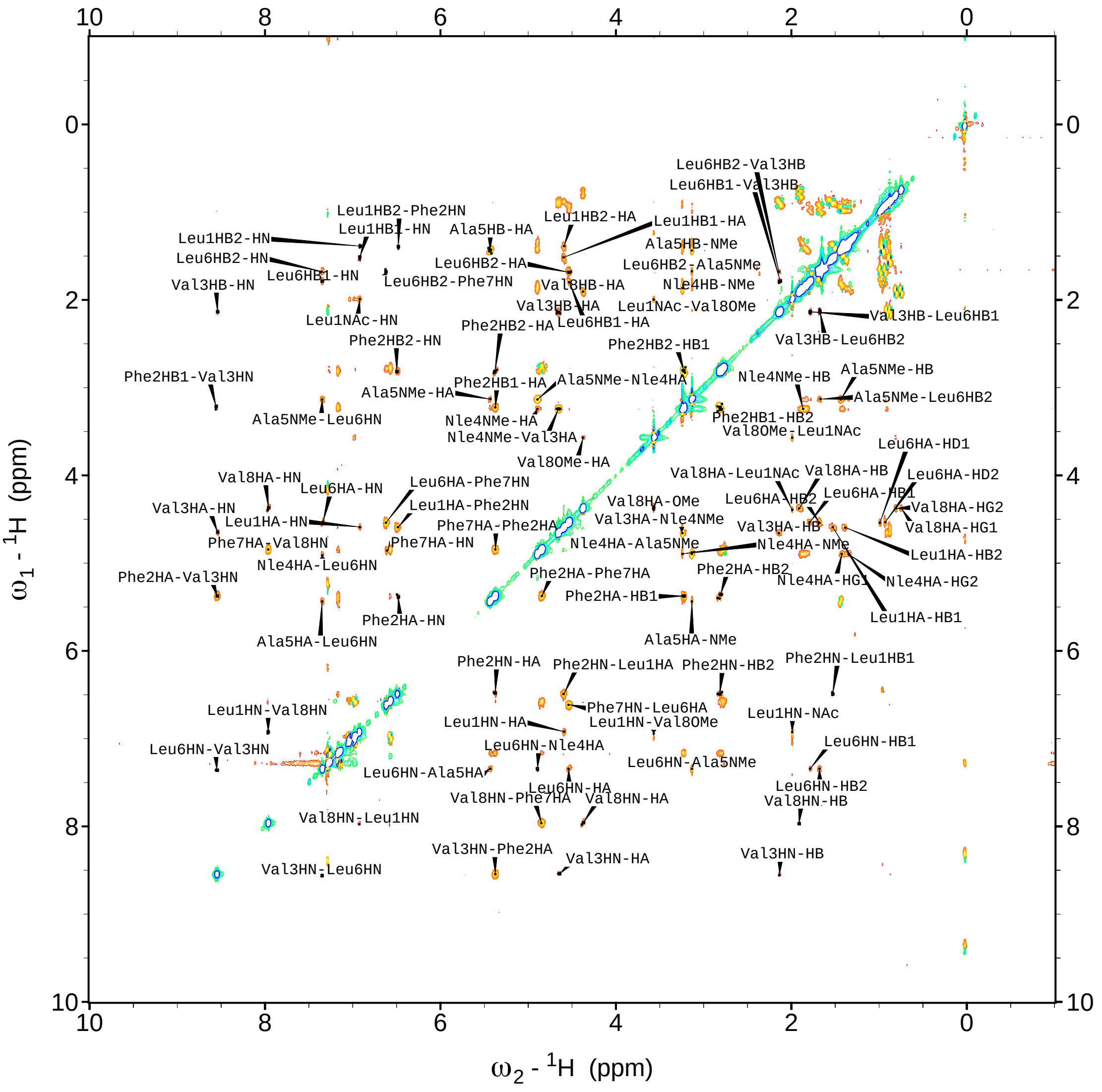

Figure S10.3: ROESY spectra with assigned peaks.

Table10.2: List of ROEs with respective NMR distances and violations.

Interactions	NMR Distance	Lower Limit	Upper Limit	Observed Distance	Violations
Leu1HA-HN	2.94	2.65	3.23	2.91	0
Phe2HN-Leu1HA	2.30	2.07	2.53	2.68	0.2
Leu1HB1-HA	2.51	2.26	2.76	2.64	0
Leu1HB1-HN	3.23	2.91	3.55	3.15	0
Leu1HB2-HA	2.61	2.35	2.87	2.93	0.1
Leu1HN-Val8HN	2.78	2.50	3.06	2.79	0
Leu1HN-Val8OMe	4.04	3.64	4.44	3.77	0
Leu1NAc-HN	2.61	2.35	3.27	2.70	0
Leu1NAc-	2.92	2.63	4.01	3.90	0

**Val8OMe**					
**Phe2HA-HN**	3.13	2.82	3.44	2.96	0
**Phe2HA-Val3HN**	2.28	2.05	2.51	2.06	0
**Phe2HB1-HA**	2.35	2.12	2.59	2.46	0
**Phe2HB1-Val3HN**	2.63	2.37	2.89	3.16	0.3
**Phe2HB2-HA**	2.36	2.12	2.60	2.46	0
**Phe2HB2-HB1**	1.80	1.62	1.98	1.75	0
**Phe2HB2-HN**	2.57	2.31	2.83	3.13	0.3
**Phe2HN-Leu1HB1**	3.70	3.33	4.07	3.52	0
**Val3HA-HN**	3.26	2.93	3.59	2.96	0
**Val3HB-HA**	2.69	2.42	2.96	3.04	0.1
**Val3HB-HN**	3.18	2.86	3.50	2.82	0
**nle4HA-HG1**	2.19	1.97	2.41	2.59	0.2
**nle4HA-HG2**	1.92	1.73	2.11	2.49	0.4
**nle4HA-Leu6HN**	2.84	2.56	3.12	3.48	0.4
***nle4HB-NMe**	2.13	1.92	3.14	3.68	0.5
**nle4NMe-Val3HA**	1.92	1.73	2.51	2.68	0.2
**nle4NMe-HA**	2.81	2.53	3.49	3.68	0.2
**Ala5HA-Leu6HN**	2.86	2.57	3.15	3.01	0
**Ala5HB-HA**	2.33	2.10	2.96	2.46	0
**Ala5HB-NMe**	2.26	2.03	3.29	3.43	0.1
**Ala5NMe-nle4HA**	1.92	1.73	2.51	2.54	0
**Ala5NMe-HA**	2.99	2.69	3.69	3.79	0.1
**Ala5NMe-Leu6HN**	2.63	2.37	3.29	3.15	0
**Leu6HA-HD1**	2.93	2.64	3.22	3.06	0
**Leu6HA-HD2**	2.64	2.38	2.90	3.23	0.3
**Leu6HA-HN**	2.70	2.43	2.97	2.93	0
**Leu6HA-Phe7HN**	2.13	1.92	2.34	2.37	0
**Leu6HB1-Val3HB**	2.81	2.53	3.09	3.28	0.2
**Leu6HB1-HA**	2.60	2.34	2.86	3.03	0.2
**Leu6HB1-HN**	3.00	2.70	3.30	2.46	-0.2
**Leu6HB2-Val3HB**	2.96	2.66	3.26	3.07	0
**Leu6HB2-HA**	2.45	2.21	2.70	2.85	0.2
**Leu6HB2-HN**	2.82	2.54	3.10	3.39	0.3
**Leu6HB2-Phe7HN**	3.27	2.94	3.60	2.66	-0.3
**Leu6HN-Val3HN**	3.28	2.95	3.61	3.45	0
**Phe7HA-Phe2HA**	2.20	1.98	2.42	2.44	0
**Phe7HA-Val8HN**	2.17	1.95	2.39	2.27	0

***Val8HA-Leu1NAc**	3.14	2.83	3.45	4.05	0.6
**Val8HA-HG1**	2.97	2.67	3.27	2.98	0
***Val8HA-HG2**	2.83	2.55	3.11	3.76	0.6
**Val8HA-HN**	2.64	2.38	2.90	2.82	0
**Val8HB-HA**	2.46	2.21	2.71	2.61	0
**Val8HN-HB**	3.44	3.10	3.78	3.69	0
**Val8OMe-HA**	3.28	2.95	4.01	4.30	0.3

* violations ≥0.5. The observed high violations can be explained by the local flexibility about the γ and δ methyl groups (Val and Leu respectively) and the terminal ester bond, peak overlap, additional J-mediated transfer and inaccuracies in the force fields.[9]

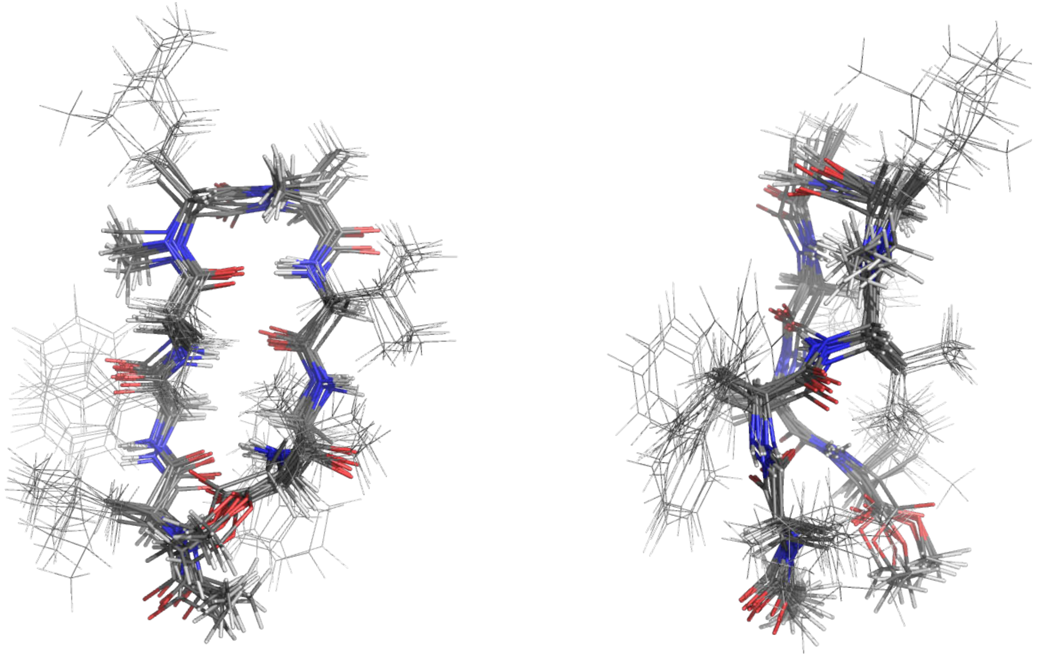

Figure S10.4: Overlay of 10 representative conformations generated using Molecular Dynamics simulation, showing both front view (left panel) and side view (right panel).

(A)

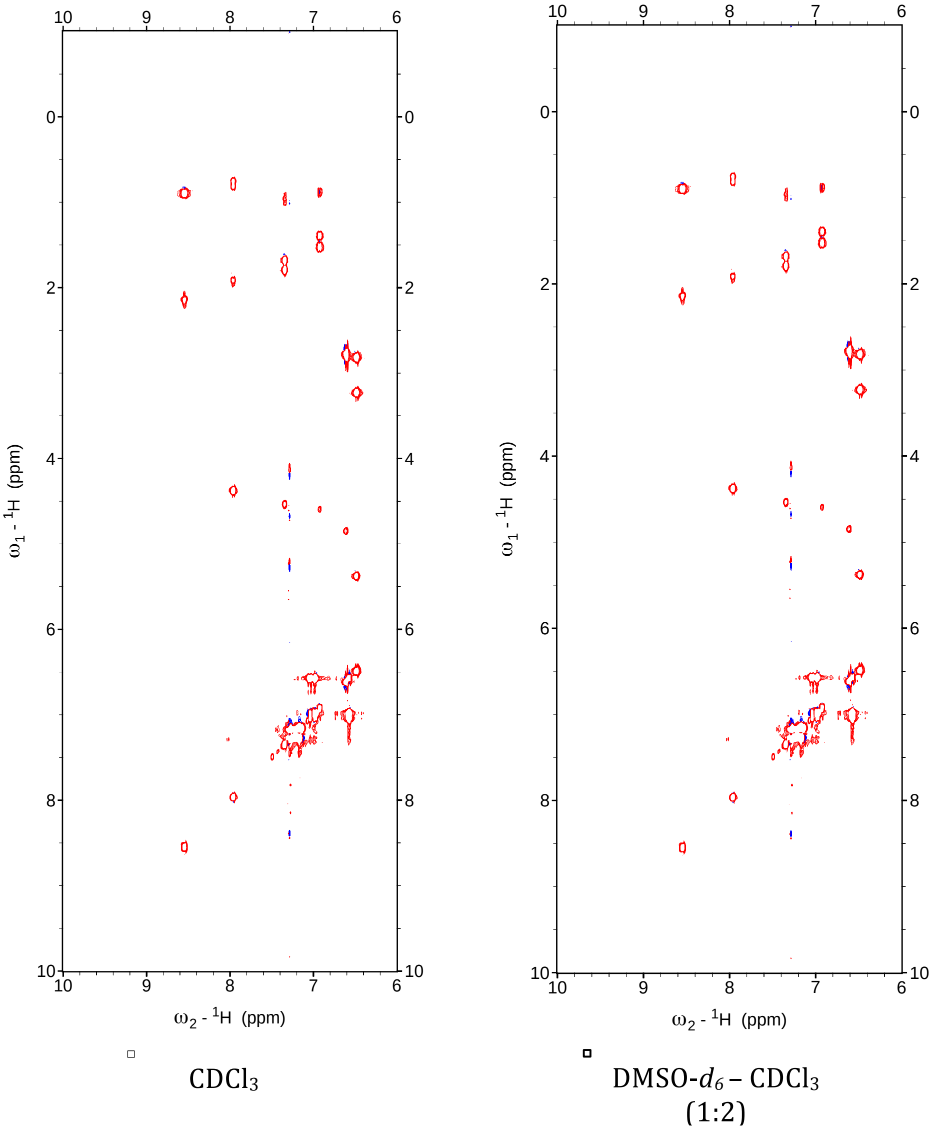

(B)

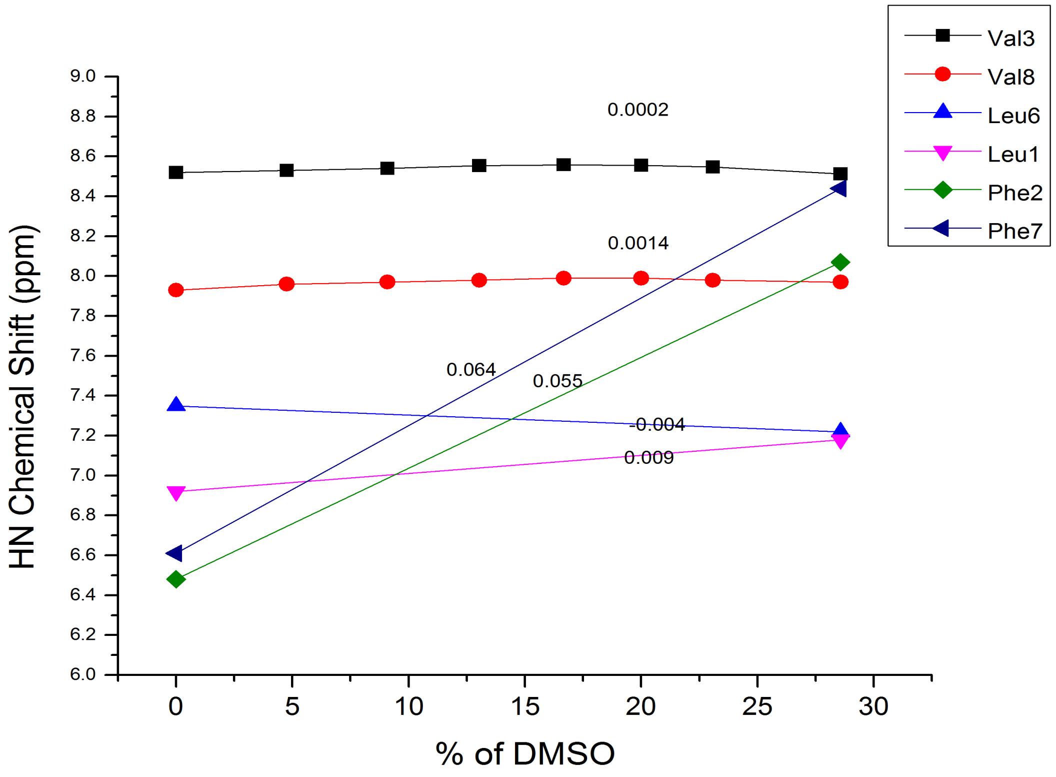

Figure S10.5: (A) TOCSY spectra in $CDCl_3$ and DMSO-d_6 – $CDCl_3$ (1:2) and (B) DMSO-d_6 titration curve indicating the solvent exposed (F2, F7) and solvent shielded (L1, V3, L6, V8) amide protons. The value indicates the slope generated by the linear fit of the data points.

Compound 11:

(A)

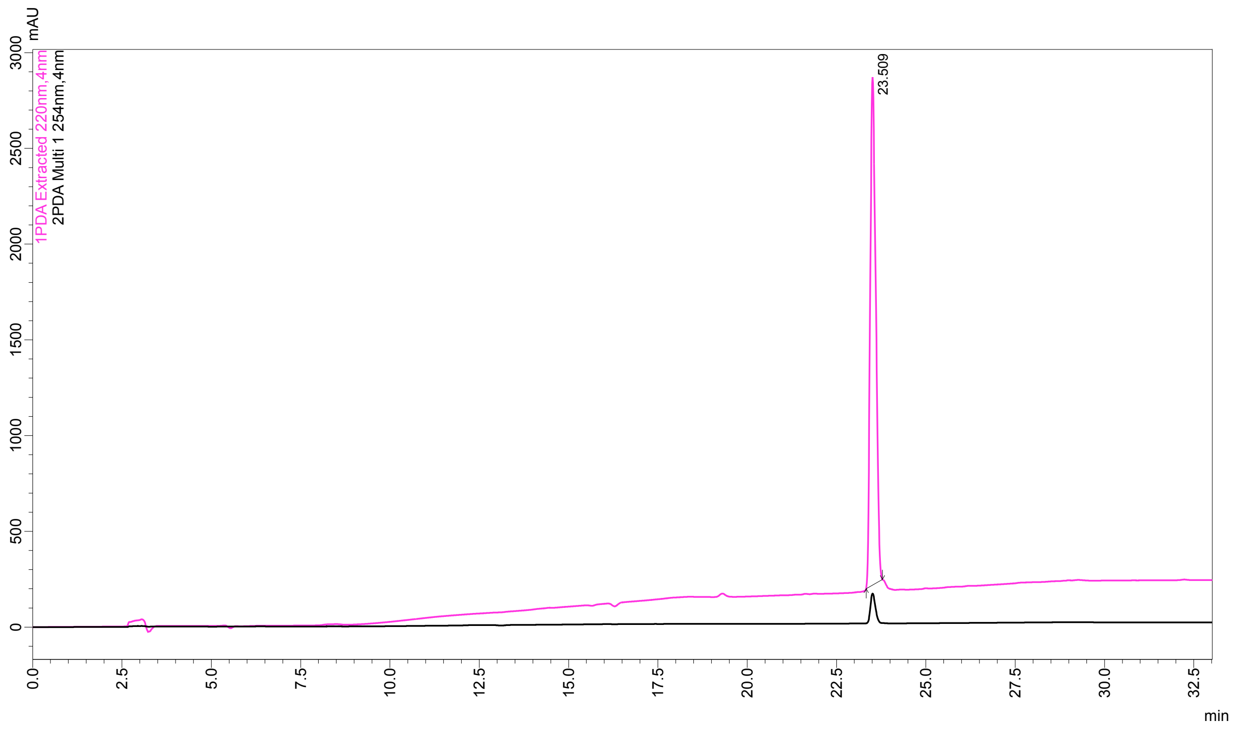

(B)

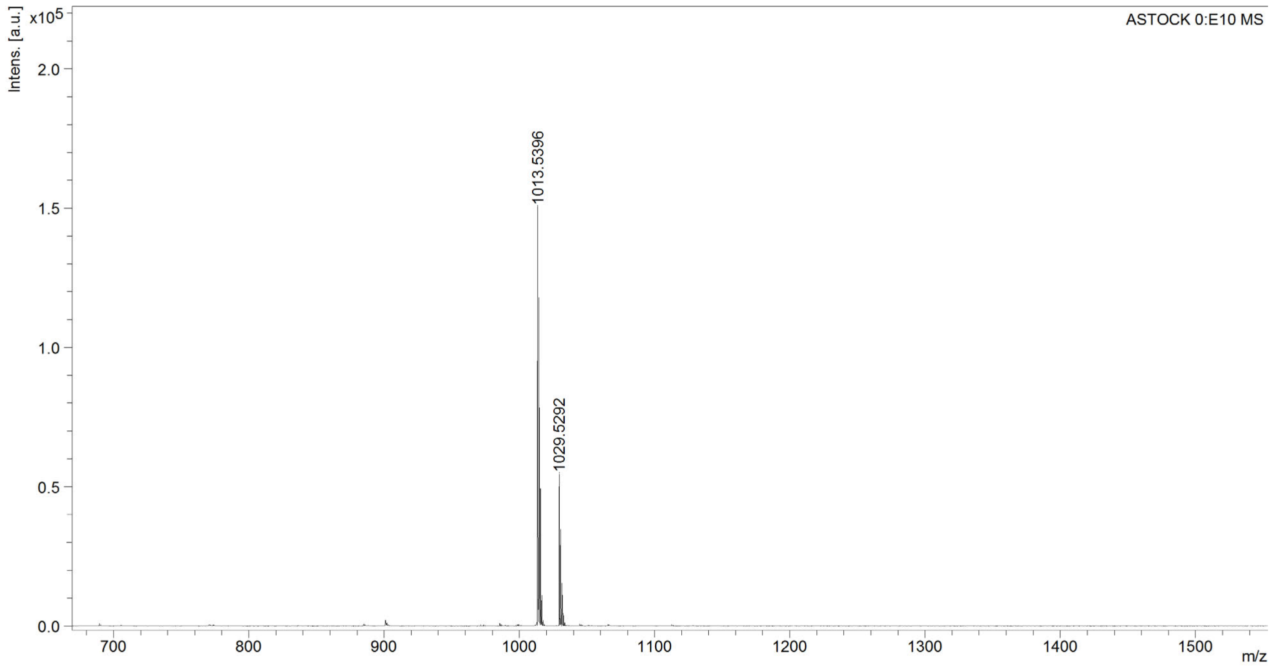

Figure S11.1: A) Analytical HPLC chromatogram of purified compound **11** at 70-100% MeOH/H_2O gradient and (B) the respective MALDI profile of the pure compound. Calculated MW: 1013.6154 [M+Na]; Observed MW: 1013.5396.

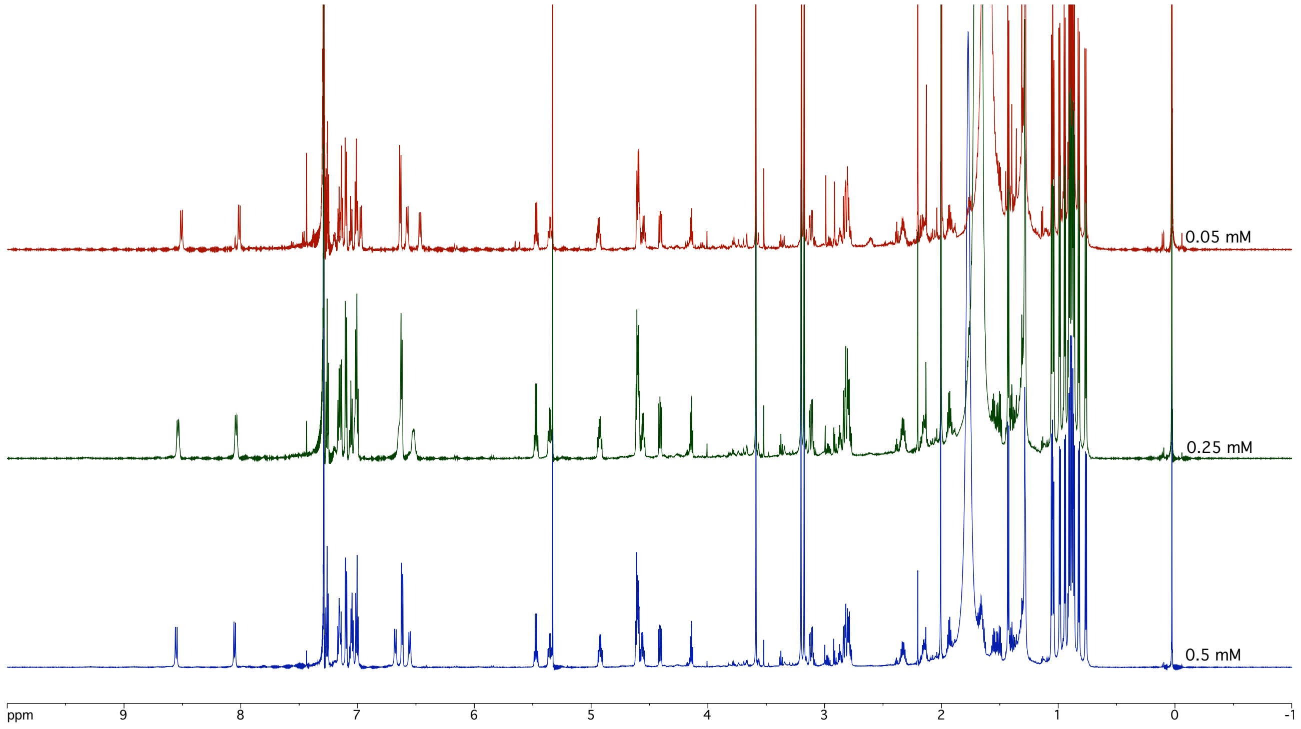

Figure S11.2: ^{1}H NMR spectra of Compound **11** at three different dilutions in $CDCl_3$ at 25°C.

Table 11.1: Chemical shifts table.

Residue	Atoms										
	HN	NMe	HA	HB		HG		HD		OMe	NAc
				1	2	1	2	1	2		
**LEU1**	6.97		4.59	1.5	1.39						1.97
**PHE2**	6.51		5.34	3.11	2.82						
**VAL3**	8.51		4.6	2.16		0.87					
**D-VAL4**		3.21	4.61	2.32		1.04					
**ALA5**		3.17	5.47	1.42							
**LEU6**	7.12		4.55	1.76	1.65						
**PHE7**	6.62		4.93	2.8							
**VAL8**	8		4.41	1.94		0.82	0.76			3.56	

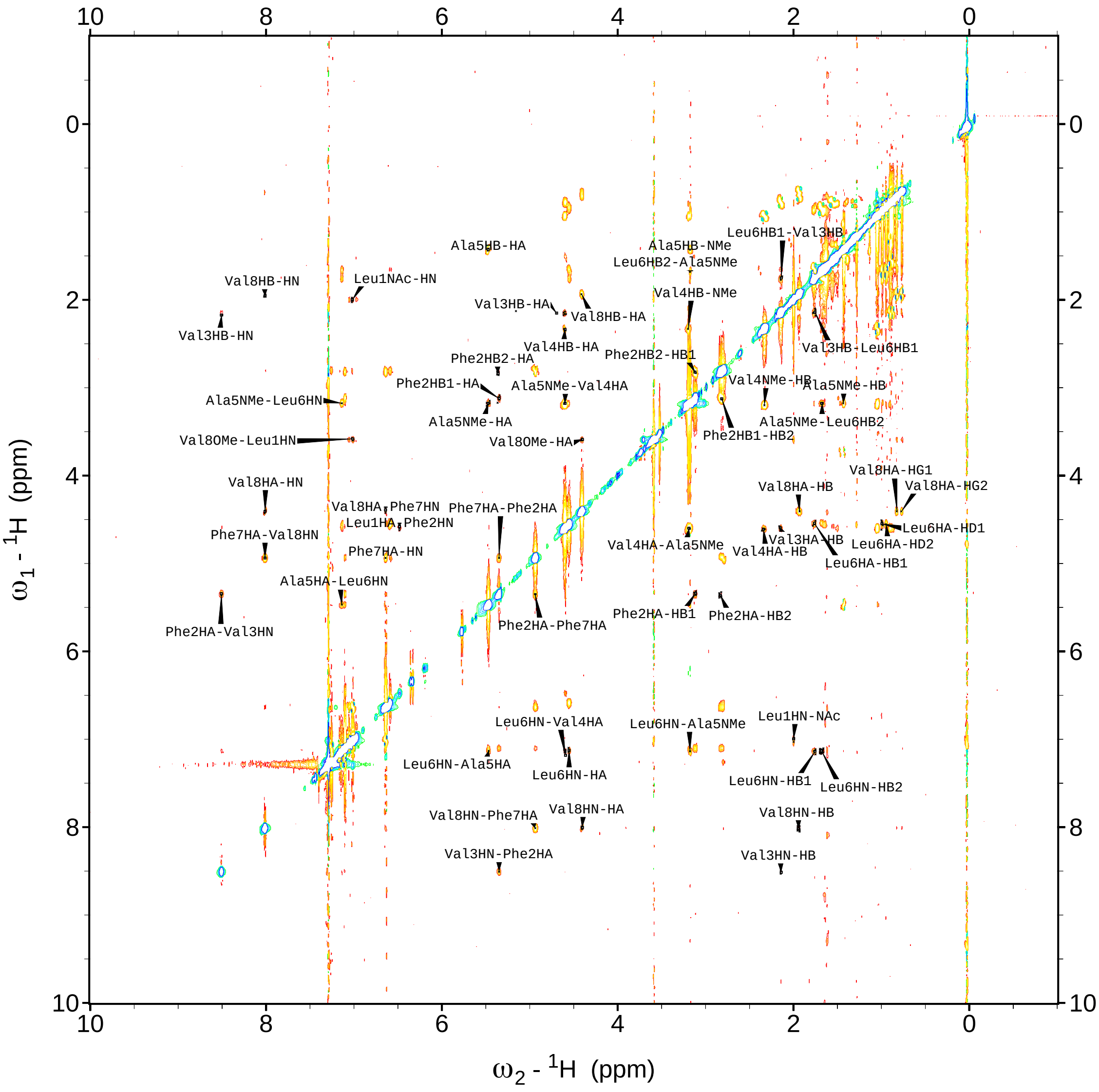

Figure S11.3: ROESY spectra with assigned peaks.

Table11.2: List of ROEs with respective NMR distances and violations.

Interactions	NMR Distance	Lower Limit	Upper Limit	Observed Distance	Violations
*Leu1HA-Phe2HN	2.96	2.66	3.26	2.16	-0.5
Leu1NAc-HN	2.86	2.58	3.55	2.66	0
Phe2HA-Val3HN	2.77	2.49	3.04	2.11	-0.4
Phe2HB1-HA	2.6	2.34	2.86	3.03	0.2
Phe2HB2-HA	3.45	3.1	3.79	2.78	-0.3
Phe2HB2-HB1	1.84	1.66	2.02	1.72	0
Val3HB-HA	2.62	2.36	2.88	3.03	0.2
Val3HB-HN	3.41	3.07	3.75	2.94	-0.1

**val4HB-HA**	2.63	2.37	3.29	3	0
**Ala5HA-Leu6HN**	2.35	2.12	2.59	2.6	0
**Ala5HB-HA**	2.34	2.57	3.55	2.52	-0.1
**Ala5HB-NMe**	2.17	1.95	3.18	3.55	0.4
**Ala5NMe-val4HA**	1.7	1.53	2.27	2.65	0.4
**Ala5NMe-HA**	2.86	2.58	3.54	3.75	0.2
**Ala5NMe-Leu6HN**	2.39	2.15	3.03	3.1	0.1
**Leu6HA-HB1**	2.67	2.4	2.94	2.7	0
***Leu6HA-HD1**	2.71	2.44	2.98	3.57	0.6
**Leu6HA-HD2**	2.53	2.28	2.79	3	0.2
**Leu6HB1-Val3HB**	2.52	2.27	2.77	3.18	0.4
**Leu6HB2-Ala5NMe**	2.59	2.33	3.25	3.57	0.3
**Leu6HN-HA**	2.8	2.52	3.08	2.9	0
**Leu6HN-HB1**	2.36	2.12	2.6	2.99	0.4
**Leu6HN-HB2**	2.68	2.41	2.95	2.78	0
**Phe7HA-Phe2HA**	2.39	2.15	2.62	2.48	0
**Phe7HA-HN**	2.41	2.17	2.65	2.89	0.2
**Phe7HA-Val8HN**	2.2	1.98	2.42	2.14	0
**Val8HA-HG1**	2.49	2.24	2.74	3	0.3
**Val8HA-HG2**	2.7	2.43	2.97	3.26	0.3
**Val8HA-HN**	2.82	2.54	3.11	2.86	0
***Val8HB-HA**	2.26	2.04	2.49	3.03	0.5
**Val8HB-HN**	3.36	3.02	3.69	2.9	-0.1
***Val8OMe-Leu1HN**	2.84	2.55	3.52	3.98	0.5
***Val8OMe-HA**	2.69	2.42	3.35	4.04	0.7
**Val8OMe-Leu1NAc**	2.63	2.37	3.7	3.52	0

* violations ≥0.5. The observed high violations can be explained by the local flexibility about the γ and δ methyl groups (Val and Leu respectively) and the terminal ester bond, peak overlap, additional J-mediated transfer and inaccuracies in the force fields.[9]

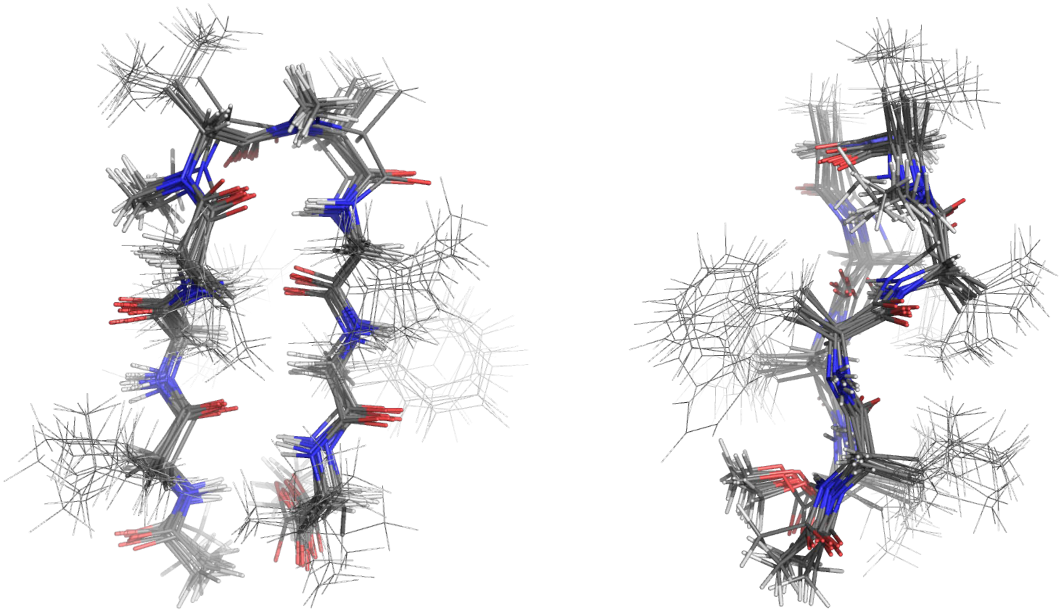

Figure S11.4: Overlay of 10 representative conformations generated using Molecular Dynamics simulation, showing both front view (left panel) and side view (right panel).

(A)

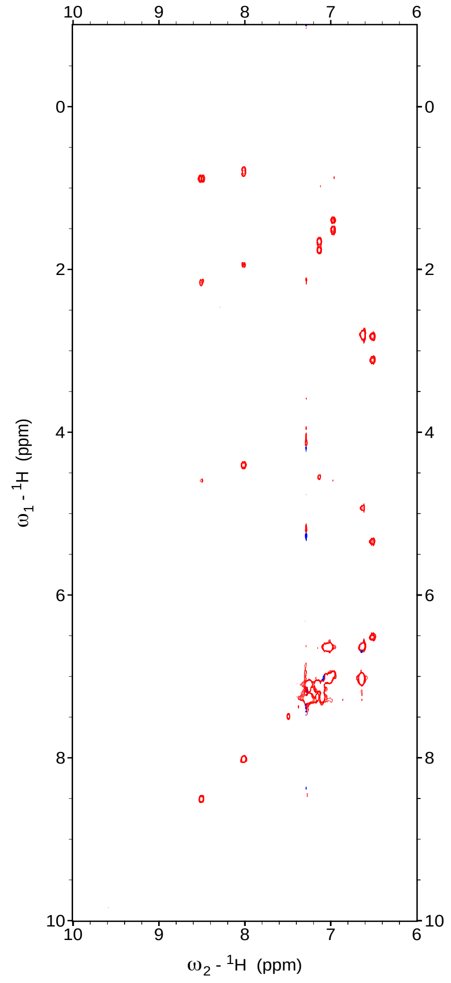

CDCl3

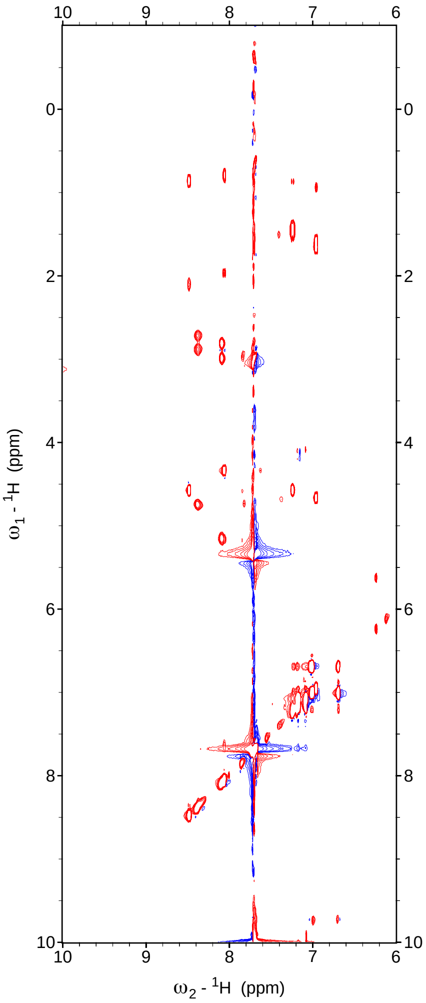

DMSO-d_6 – $CDCl_3$
(1:2)

(B)

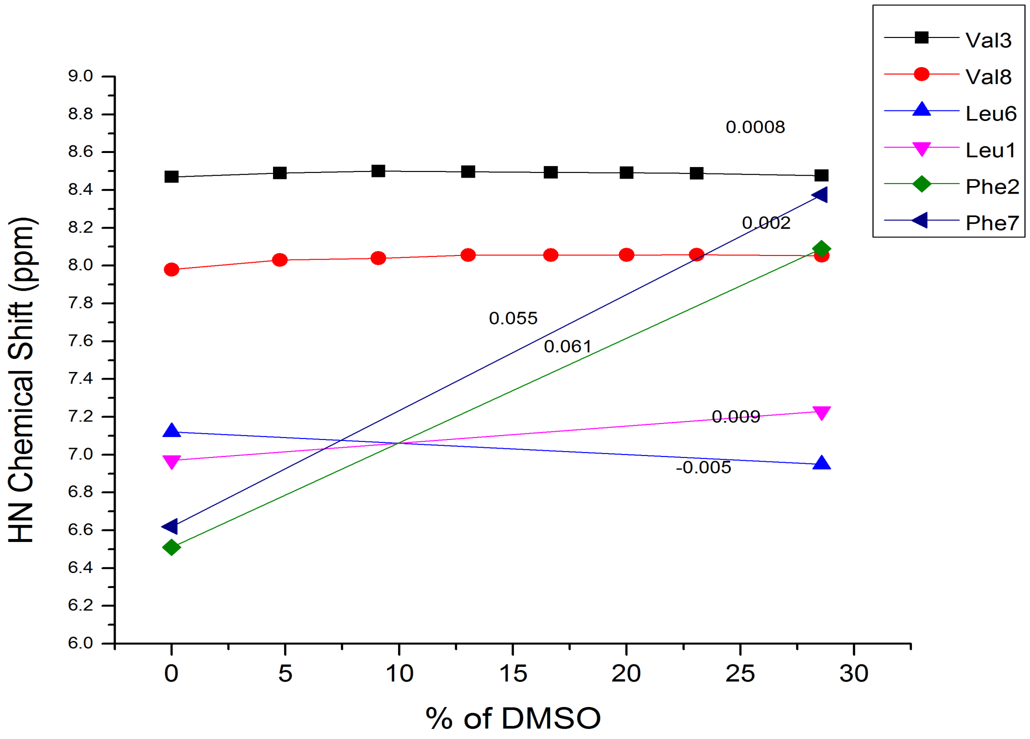

Figure S11.5: (A) TOCSY spectra in $CDCl_3$ and DMSO-d_6 – $CDCl_3$ (1:2) and (B) DMSO-d_6 titration curve indicating the solvent exposed (F2, F7) and solvent shielded (L1, V3, L6, V8) amide protons. The value indicates the slope generated by the linear fit of the data points.

Compound 12:

(A)

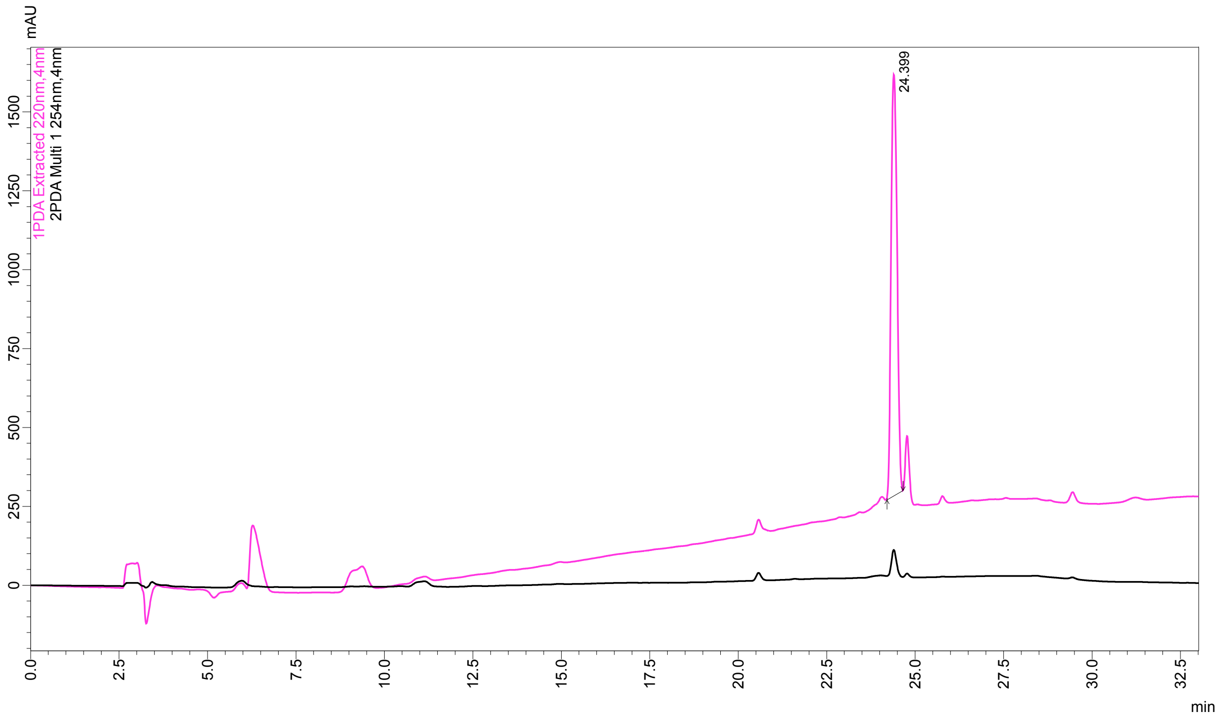

(B)

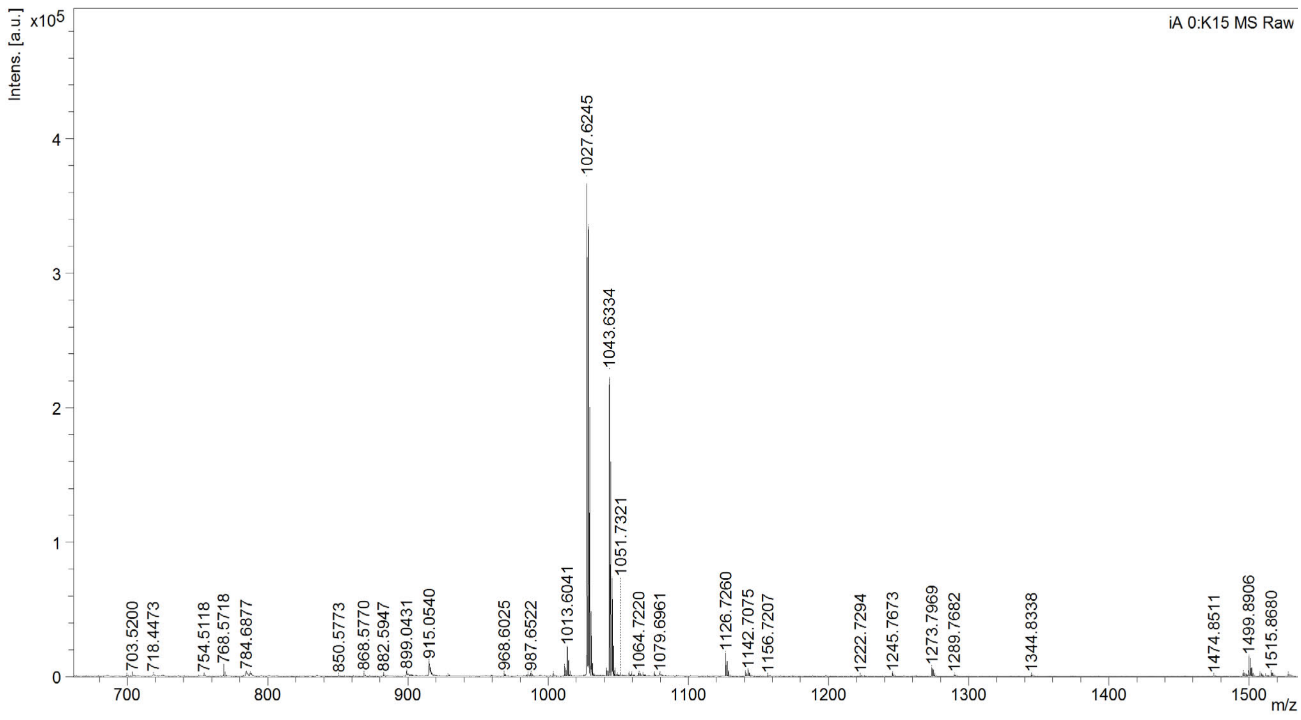

Figure S12.1: A) Analytical HPLC chromatogram of purified compound **12** at 70-100% MeOH/H_2O gradient and (B) the respective MALDI profile of the pure compound. Calculated MW: 1027.6310 [M+Na]; Observed MW: 1027.6245.

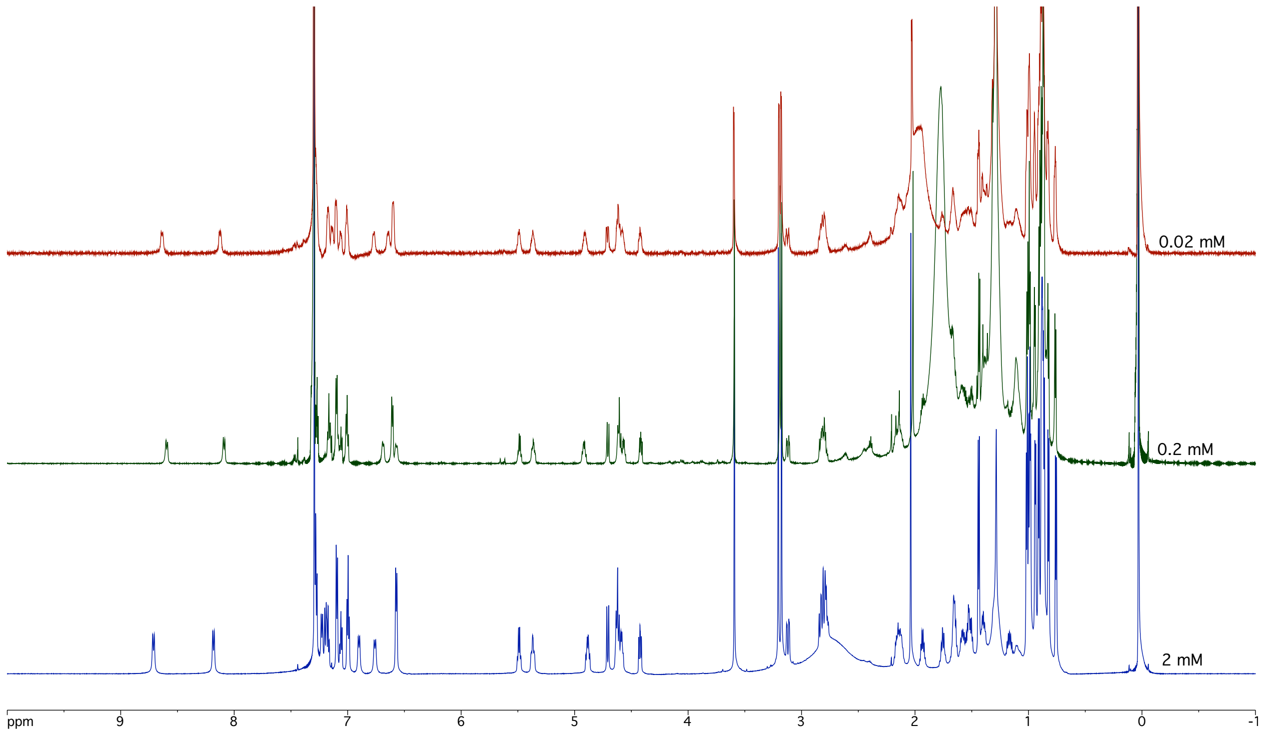

Figure S12.2: ^{1}H NMR spectra of Compound **12** at three different dilutions in $CDCl_3$ at 25°C.

Table 12.1: Chemical shifts table.

Residue	Atoms										
	HN	NMe	HA	HB		HG		HD		OMe	NAc
				1	2	1	2	1	2		
**LEU1**	7.10		4.6	1.51	1.4	1.55		0.89	0.86		1.97
**PHE2**	6.63		5.35	3.11	2.82						
**VAL3**	8.64		4.59	2.14		0.9	0.86				
**D-ILE4**		3.18	4.7	2.13		1.15	1	1.57			
**ALA5**		3.17	5.47	1.43							
**LEU6**	7.27		4.55	1.75	1.66			0.99	0.94		
**PHE7**	6.73		4.94	2.81							
**VAL8**	8.15		4.4	1.93		0.83	0.76			3.56	

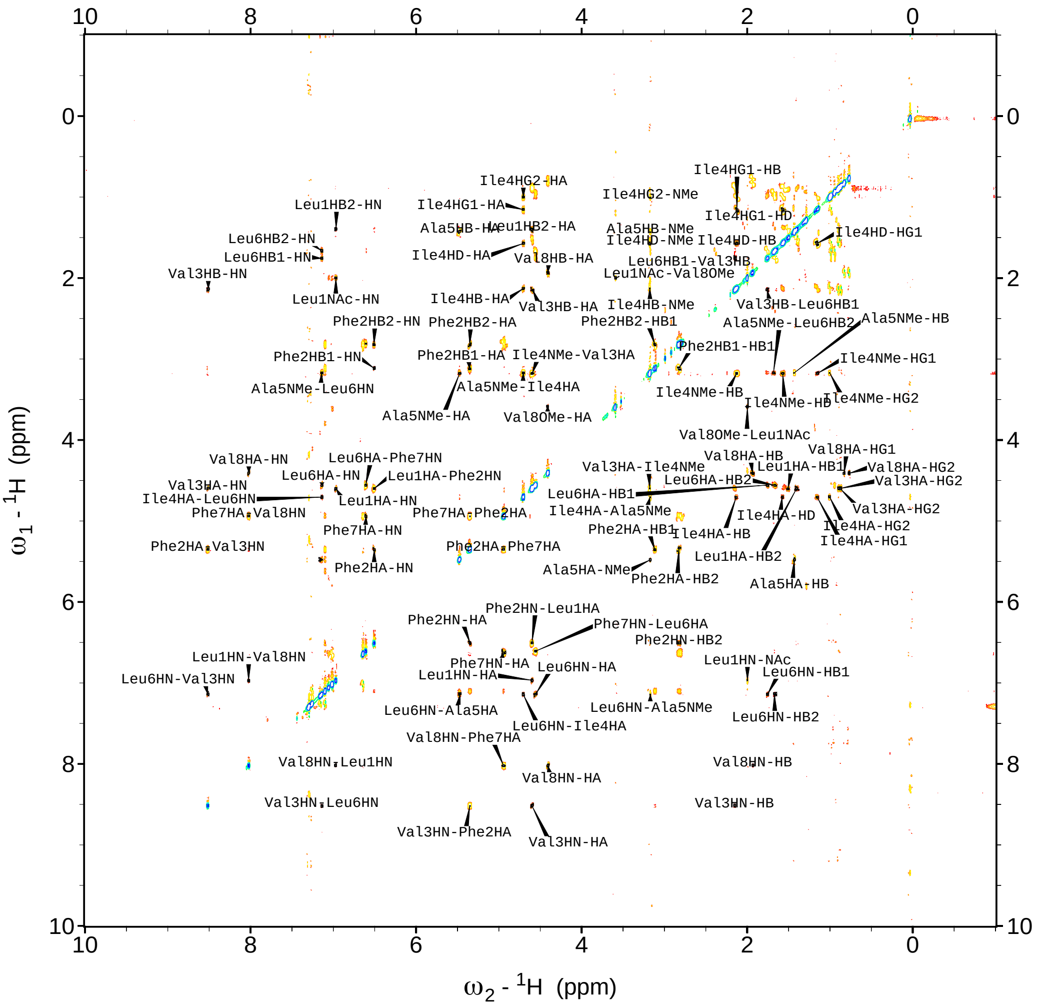

Figure S12.3: ROESY spectra with assigned peaks.

Table12.2: List of ROEs with respective NMR distances and violations.

Interactions	NMR Distance	Lower Limit	Upper Limit	Observed Distance	Violations
Leu1HA-HB1	2.82	2.54	3.10	2.56	0
Leu1HA-HN	3.01	2.71	3.31	2.87	0
Leu1HB2-HA	2.82	2.54	3.10	3.03	0
Leu1HB2-HN	3.16	2.84	3.48	3.09	0
Leu1HN-Val8HN	3.32	2.99	3.65	3.03	0
Leu1NAc-HN	2.82	2.54	3.50	2.68	0
Leu1HB2-Phe2HN	3.83	3.45	4.21	4.38	0.2
Leu1NAc-Val8OMe	3.03	2.73	4.13	4.16	0
Phe2HA-HN	3.08	2.77	3.39	2.97	0
Phe2HA-Val3HN	2.32	2.09	2.55	2.07	0
Phe2HB1-Val3HN	3.07	2.76	3.38	3.28	0
Phe2HB1-HN	3.57	3.21	3.93	3.76	0
Phe2HB1-HA	2.41	2.17	2.65	2.57	0
Phe2HB2-HB1	1.80	1.62	1.98	1.74	0

**Phe2HB2-HA**	2.61	2.35	2.87	2.43	0
**Phe2HB2-HN**	2.79	2.51	3.07	3.18	0.1
**Leu1HA-Phe2HN**	2.33	2.10	2.56	2.33	0
**Val3HA-HN**	3.09	2.78	3.40	2.97	0
**Val3HB-HA**	2.36	2.12	2.60	3.02	0.4
***Val3HA-HG1**	2.29	2.06	2.52	3.00	0.5
**Val3HA-HG2**	2.61	2.35	2.87	3.19	0.3
**Val3HB-HN**	2.62	2.36	2.88	2.84	0
**ile4HA-Leu6HN**	3.35	3.02	3.69	3.71	0
**ile4HB-NMe**	2.19	1.97	3.21	2.94	0
**ile4HG1-HA**	2.71	2.44	2.98	2.98	0
**ile4HG2-HA**	2.49	2.24	3.14	2.98	0
**ile4HB-HA**	2.82	2.54	3.50	3.00	0
**ile4HD1-HA**	2.77	2.49	3.45	3.47	0
**ile4HD1-HB**	2.64	2.38	3.70	3.54	0
**ile4HG1-HB**	2.55	2.30	2.81	2.48	0
**ile4NMe-Val3HA**	1.99	1.79	2.59	2.86	0.3
**Leu6HN-Ala5HA**	2.27	2.04	2.50	2.67	0.2
**Ala5HB-HA**	2.22	2.00	2.84	2.48	0
**Ala5HB-NMe**	2.46	2.21	3.51	3.47	0
**Ala5NMe-ile4HA**	1.97	1.77	2.57	2.55	0
**Ala5NMe-HA**	3.30	2.97	4.03	3.79	0
**Ala5NMe-Leu6HN**	2.59	2.33	3.25	3.35	0.1
**Leu6HA-HB1**	2.82	2.54	3.10	2.85	0
**Leu6HA-HB2**	2.63	2.37	2.89	2.88	0
**Leu6HA-HN**	2.88	2.59	3.17	2.94	0
**Leu6HA-Phe7HN**	2.25	2.03	2.48	2.29	0
**Leu6HB1-Val3HB**	2.65	2.39	2.92	2.96	0
**Leu6HN-HB1**	2.78	2.50	3.06	2.73	0
**Leu6HB2-HN**	2.62	2.36	2.88	3.09	0.2
**Leu6HN-Val3HN**	3.67	3.30	4.04	3.16	-0.1
**Phe7HA-Phe2HA**	2.21	1.99	2.43	2.41	0
**Phe7HA-HN**	2.99	2.69	3.29	2.94	0
**Phe7HA-Val8HN**	2.29	2.06	2.52	2.20	0
**Val8HA-HG1**	2.95	2.66	3.25	2.95	0
**Val8HA-HG2**	3.24	2.92	3.56	3.60	0
**Val8HA-HN**	2.95	2.66	3.25	2.84	0
**Val8HB-HA**	2.56	2.30	2.82	2.81	0
**Val8HN-HB**	3.81	3.43	4.19	3.41	0
**Val8OMe-HA**	2.21	1.99	2.83	3.28	0.4

* violations ≥0.5. The observed high violations can be explained by the local flexibility about the γ and δ methyl groups (Val and Leu respectively) and the terminal ester bond, peak overlap, additional J-mediated transfer and inaccuracies in the force fields.[9]

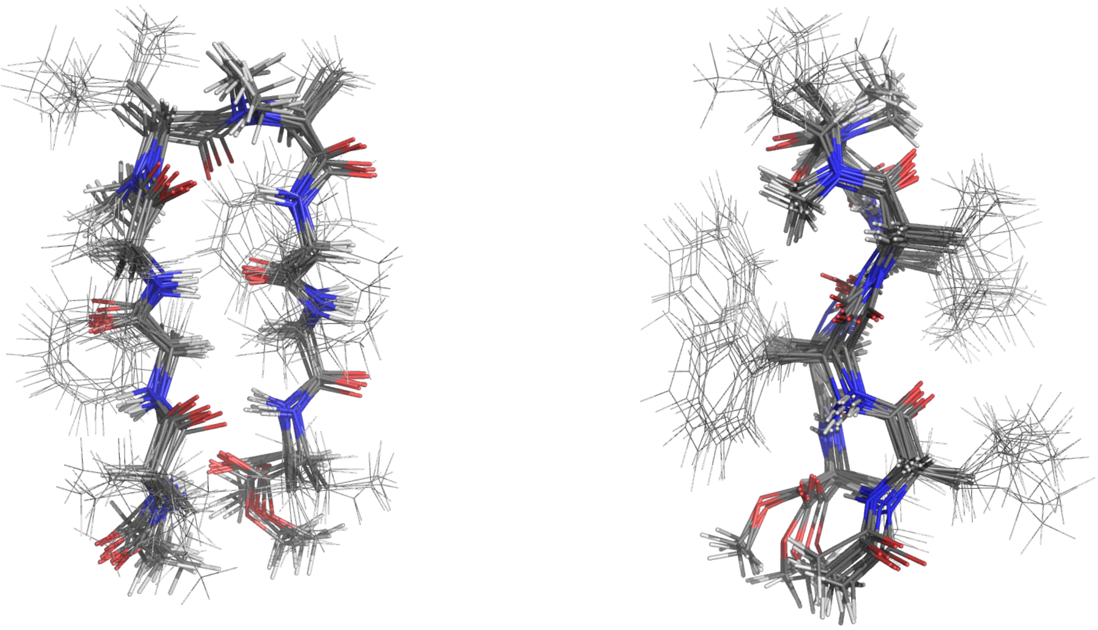

Figure S12.4: Overlay of 10 representative conformations generated using Molecular Dynamics simulation, showing both front view (left panel) and side view (right panel).

(A)

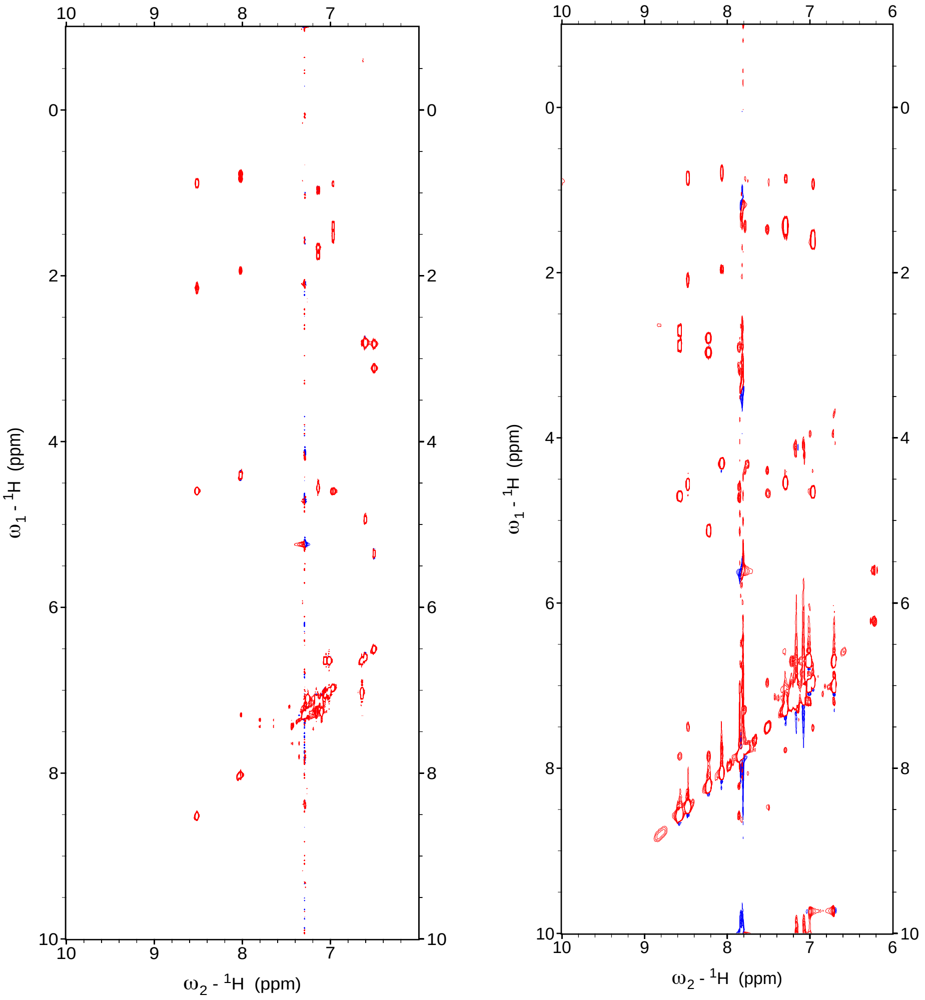

$CDCl_3$

DMSO-d_6 – $CDCl_3$ (1:2)

(B)

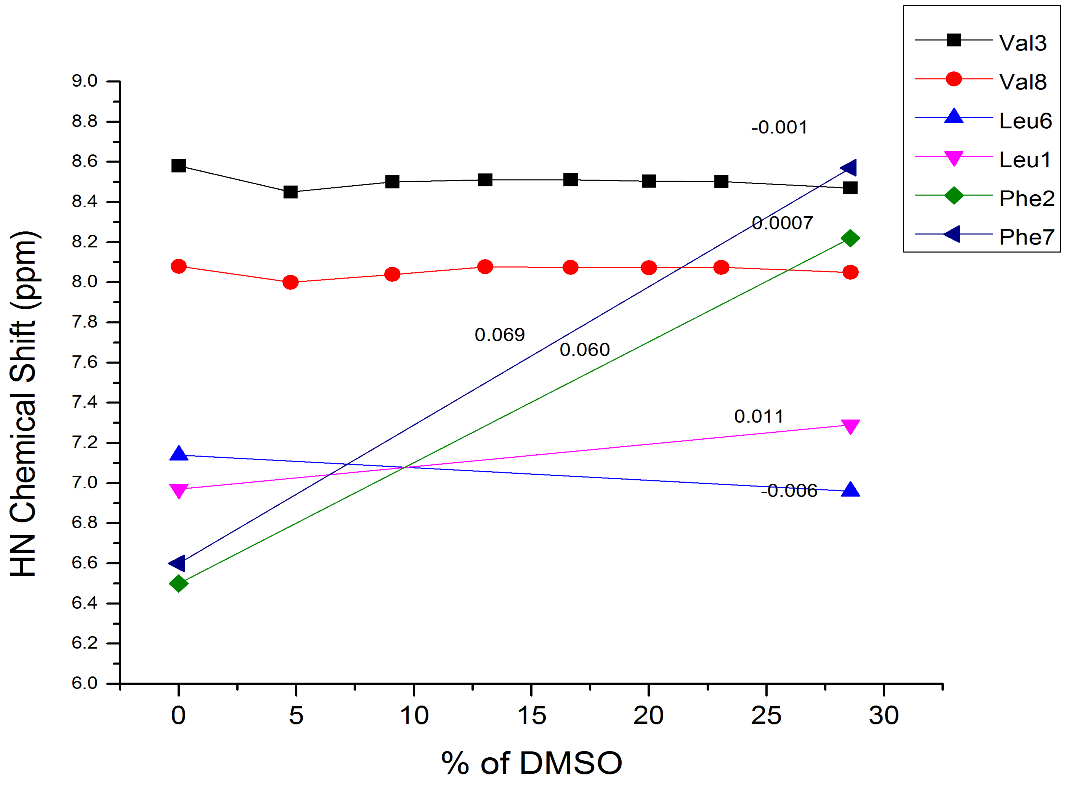

Figure S12.5: (A) TOCSY spectrum in $CDCl_3$ and DMSO-d_6 – $CDCl_3$ (1:2) and (B) DMSO-d_6 titration curve indicating the solvent exposed (F2, F7) and solvent shielded (V3, L6, V8) amide protons. The value indicates the slope generated by the linear fit of the data points.

Compound 13:

(A)

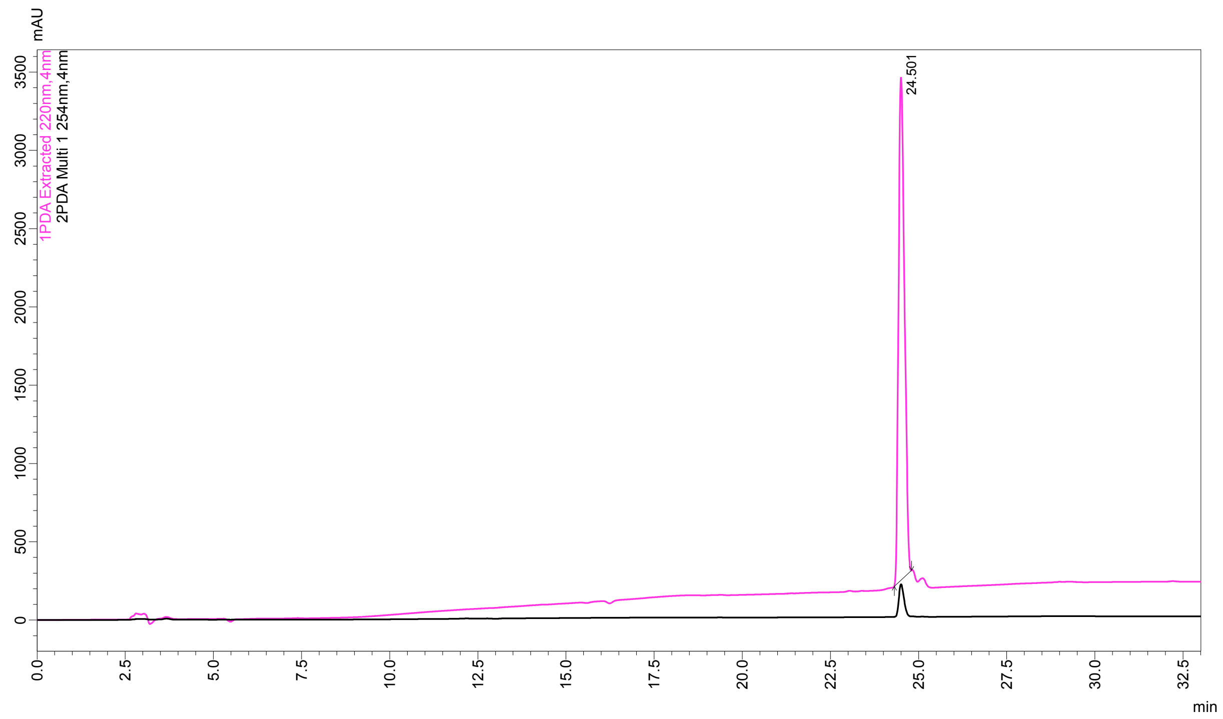

(B)

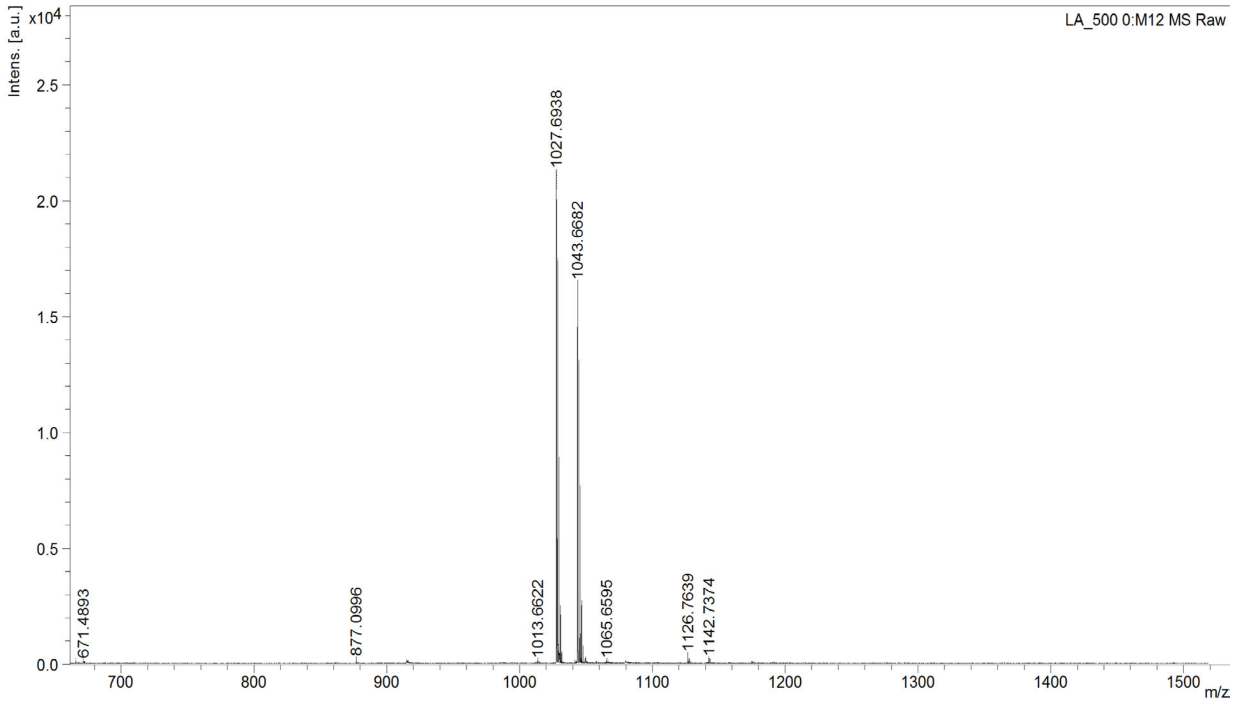

Figure S13.1: : A) Analytical HPLC chromatogram of purified compound **12** at 70-100% MeOH/H_2O gradient and (B) the respective MALDI profile of the pure compound. Calculated MW: 1027.6310 [M+Na]; Observed MW: 1027.6938.

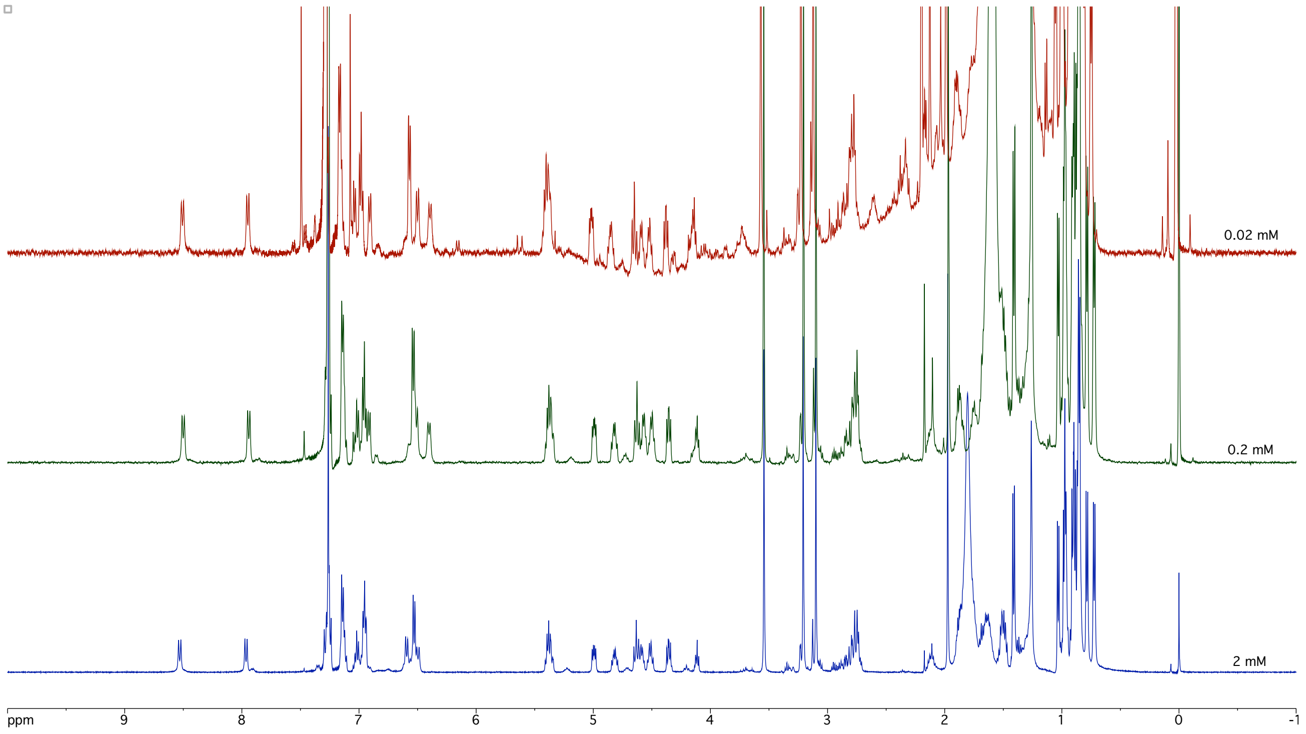

Figure S13.2: ^{1}H NMR spectra of Compound **13** at three different dilutions in $CDCl_3$ at 25°C.

Table 13.1: Chemical shifts table.

**Residue**	**Atoms**										
	**HN**	**NMe**	**HA**	**HB**		**HG**		**HD**		**OMe**	**NAc**
				1	2	1	2	1	2		
**LEU1**	7.07		4.6	1.53	1.4			0.87			1.98
**PHE2**	6.62		5.38	3.25	2.82						
**VAL3**	8.61		4.66	2.14		0.91	0.87				
**D-LEU4**		3.23	5.02	1.89	1.52	1.63		1.05	1		
**ALA5**		3.12	5.41	1.44							
**LEU6**	7.33		4.54	1.77	1.7	1.65		0.99	0.93		
**PHE7**	6.73		4.82	2.77							
**VAL8**	8.03		4.38	1.91		0.81	0.75			3.54	

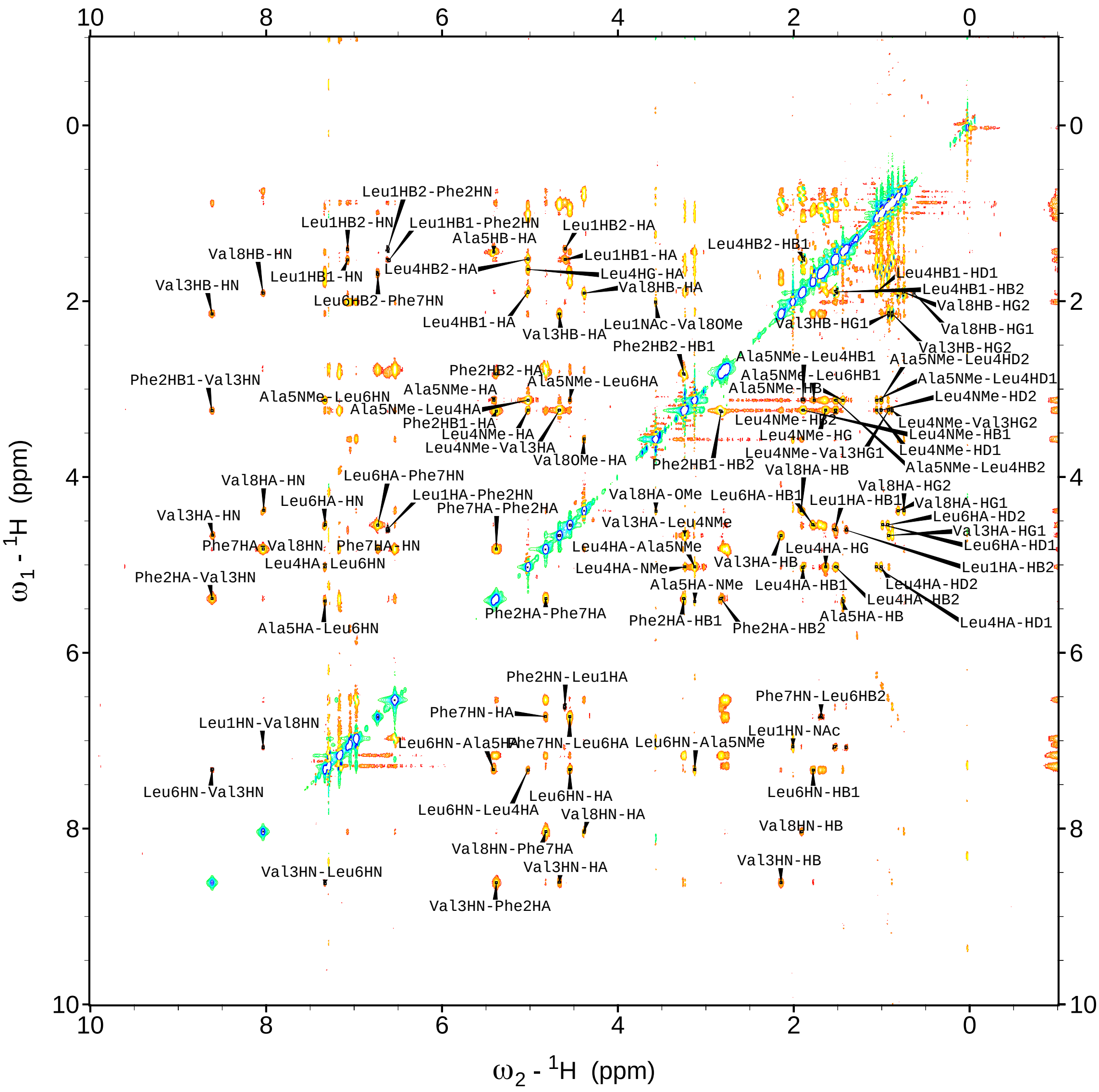

Figure S13.3: ROESY spectra with assigned peaks.

Table13.2: List of ROEs with respective NMR distances and violations.

Interactions	NMR Distance	Lower Limit	Upper Limit	Observed Distance	Violations
**Leu1HB1-HA**	2.92	2.63	3.21	2.59	0
**Leu1HB1-HN**	3.33	3.00	3.66	3.37	0
**Leu1HB2-HA**	3.30	2.97	3.63	3.05	0
**Leu1HB2-HN**	3.66	3.29	4.03	3.14	-0.2
**Leu1HN-Val8HN**	3.98	2.97	3.63	2.63	-0.3
**Leu1HA-Phe2HN**	2.80	2.52	3.08	2.31	-0.2
**Leu1HA-HN**	3.39	3.05	3.73	2.89	-0.2
**Leu1HN-NAc**	2.56	2.30	2.82	2.71	0
**Leu1NAc-Val8OMe**	2.77	2.49	3.85	3.63	0
**Phe2HA-Val3HN**	2.66	2.39	2.93	2.20	-0.2

**Phe2HB1-HA**	2.41	2.17	2.65	2.58	0
**Phe2HB2-HA**	2.94	2.65	3.23	3.08	0
**Phe2HB2-HB1**	1.71	1.54	1.88	1.73	0
**Val3HA-HN**	3.28	2.95	3.61	2.97	0
**Val3HB-HA**	2.61	2.35	2.87	3.03	0.2
**Val3HB-HN**	3.19	2.87	3.51	2.79	-0.1
**Val3HA-HG2**	2.51	2.26	2.76	3.15	0.4
**Val3HA-HG1**	2.49	2.24	2.74	2.99	0.2
**Val3HB-Leu6HB1**	2.75	2.48	3.03	2.87	0
**Val3HB-Leu6HB2**	2.95	2.66	3.25	2.93	0
**Val3HB-HG1**	2.30	2.07	2.53	2.49	0
**Val3HB-HG2**	2.35	2.12	2.59	2.49	0
**leu4NMe-Val3HN**	3.44	3.10	4.18	4.00	0
**leu4HA-Leu6HN**	3.16	2.84	3.48	3.51	0
**leu4HB1-HA**	2.59	2.33	2.85	2.59	0
**leu4HB2-HB1**	1.80	1.62	1.98	1.72	0
**leu4HG-HA**	2.64	2.38	2.90	2.86	0
**leu4NMe-Val3HA**	1.86	1.67	2.45	2.70	0.3
**leu4NMe-HA**	2.77	2.49	3.45	3.67	0.2
**Ala5HA-Leu6HN**	3.01	2.71	3.31	3.03	0
**Ala5HB-HA**	2.37	2.13	3.01	2.45	0
**Ala5NMe-HB**	2.22	2.00	3.24	3.43	0.2
**Ala5NMe-Leu4HD1**	3.16	2.84	4.28	4.58	0.3
**Ala5NMe-Leu4HA**	1.84	1.66	2.42	2.55	0.1
**Ala5NMe-HA**	3.06	2.75	3.77	3.78	0
**Ala5NMe-Leu6HN**	2.59	2.33	3.25	3.39	0.1
**Ala5NMe-Leu6HB1**	3.42	3.08	4.16	4.27	0.1
**Leu6HA-HB1**	2.55	2.30	2.81	3.02	0.2
**Leu6HA-HD1**	2.77	2.49	3.05	3.00	0
**Leu6HA-HD2**	2.64	2.38	2.90	3.28	0.4
**Leu6HA-HN**	2.81	2.53	3.09	2.95	0
**Leu6HA-Phe7HN**	2.29	2.06	2.52	2.52	0
**Leu6HN-Val3HN**	2.96	2.66	3.26	3.19	0
**Leu6HN-HB1**	2.86	2.57	3.15	2.59	0
**Leu6HB2-Phe7HN**	3.15	2.84	3.47	2.44	-0.4
**Phe7HA-Phe2HA**	2.36	2.12	2.60	2.54	0
**Phe7HA-HN**	3.10	2.79	3.41	2.86	0
**Phe7HA-Val8HN**	2.32	2.09	2.55	2.31	0
**Val8HA-HG1**	2.66	2.39	2.93	2.98	0.1
***Val8HA-HG2**	2.79	2.51	3.07	3.74	0.7
**Val8HA-HN**	3.01	2.71	3.31	2.86	0
**Val8HB-HA**	2.38	2.14	2.62	2.60	0
**Val8HB-HN**	3.46	3.11	3.81	3.64	0

***Val8OMe-HA**	3.11	2.80	3.82	4.35	0.5
**Val8HB-HG1**	2.41	2.17	2.65	2.47	0
**Val8HB-HG2**	2.36	2.12	2.60	2.47	0

* violations ≥0.5. The observed high violations can be explained by the local flexibility about the γ and δ methyl groups (Val and Leu respectively) and the terminal ester bond, peak overlap, additional J-mediated transfer and inaccuracies in the force fields.[9]

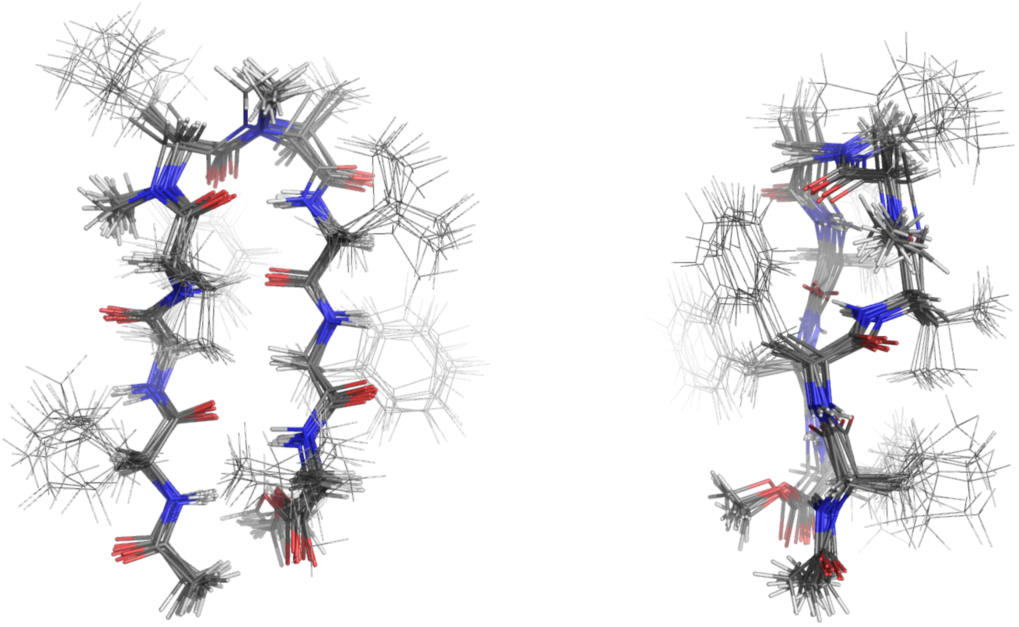

Figure S13.4: Overlay of 10 representative conformations generated using Molecular Dynamics simulation, showing both front view (left panel) and side view (right panel).

(A)

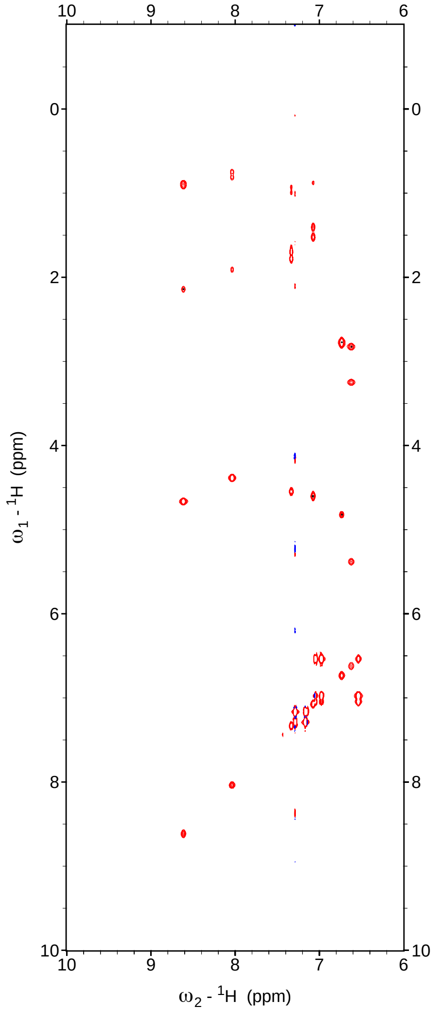

CDCl3

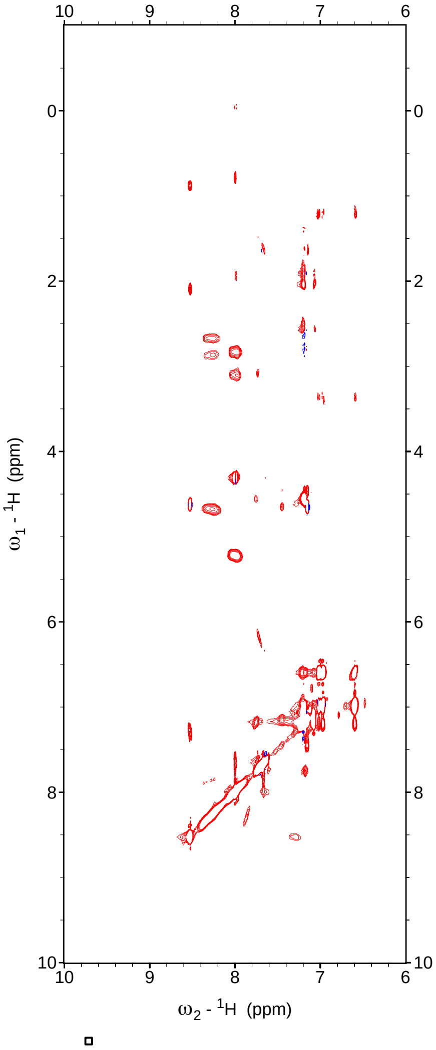

DMSO-d_6 – $CDCl_3$
(1:2)

(B)

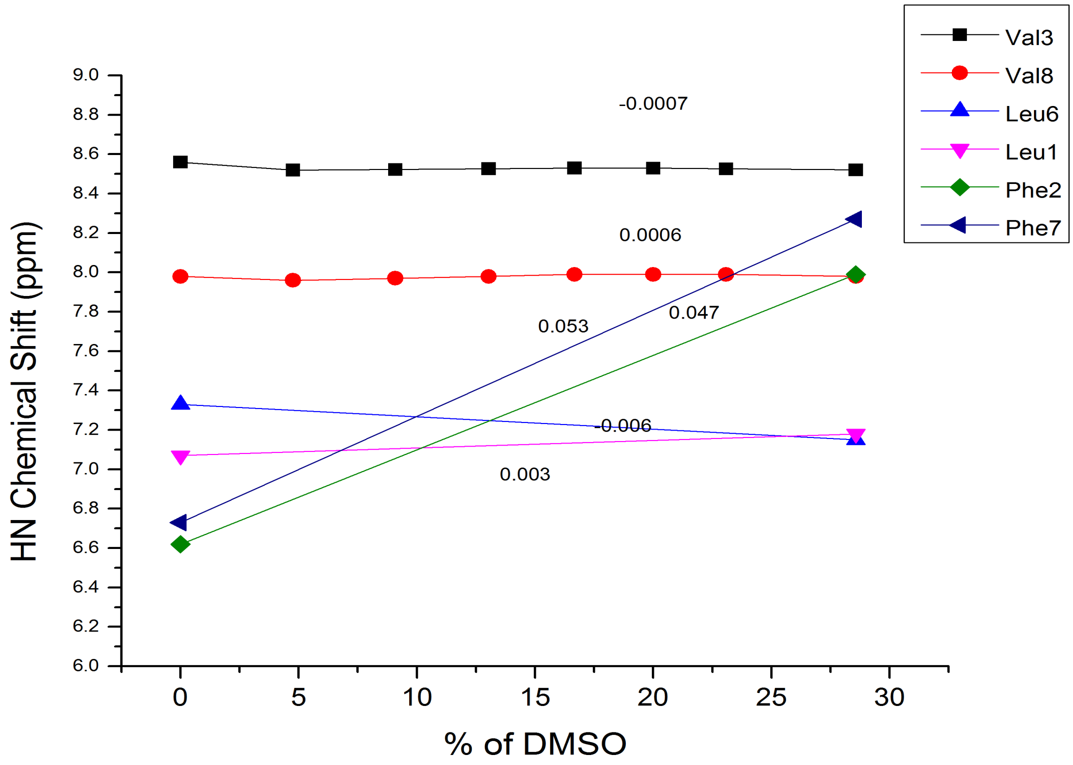

Figure S13.5: (A) TOCSY spectrum in $CDCl_3$ and DMSO-d_6 – $CDCl_3$ (1:2) and (B) DMSO-d_6 titration curve indicating the solvent exposed (F2, F7) and solvent shielded (L1, V3, L6, V8) amide protons. The value indicates the slope generated by the linear fit of the data points.

Compound 14:

(A)

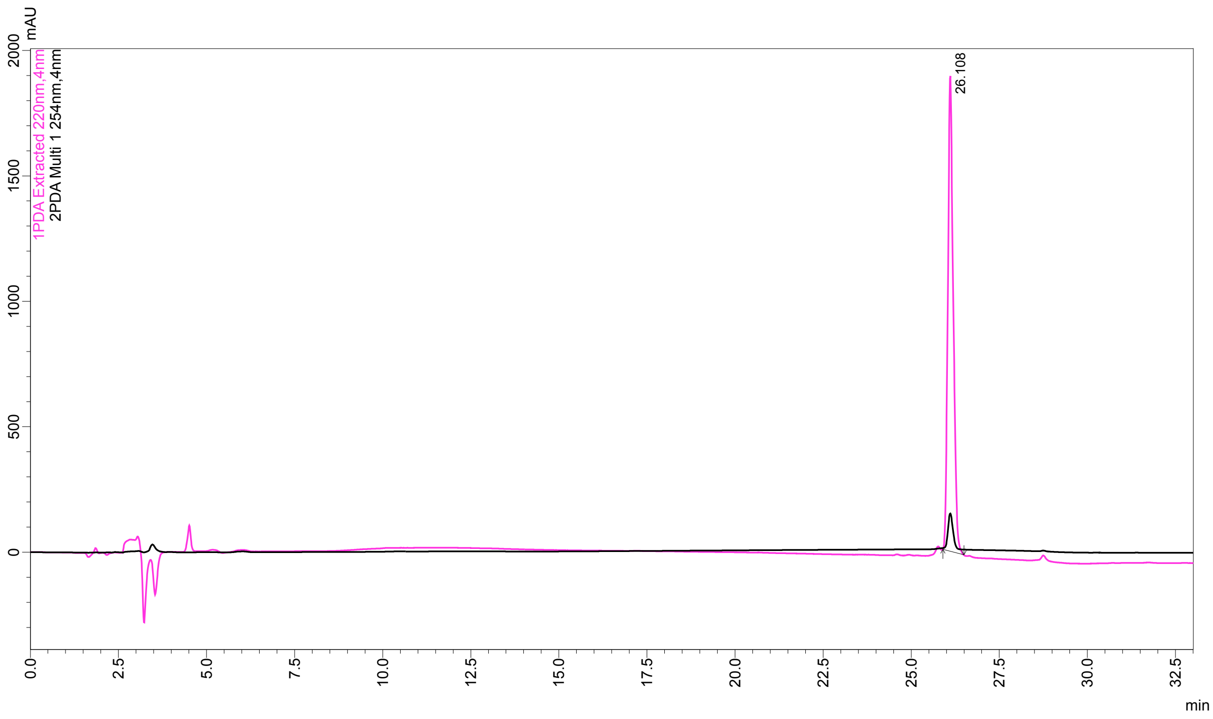

(B)

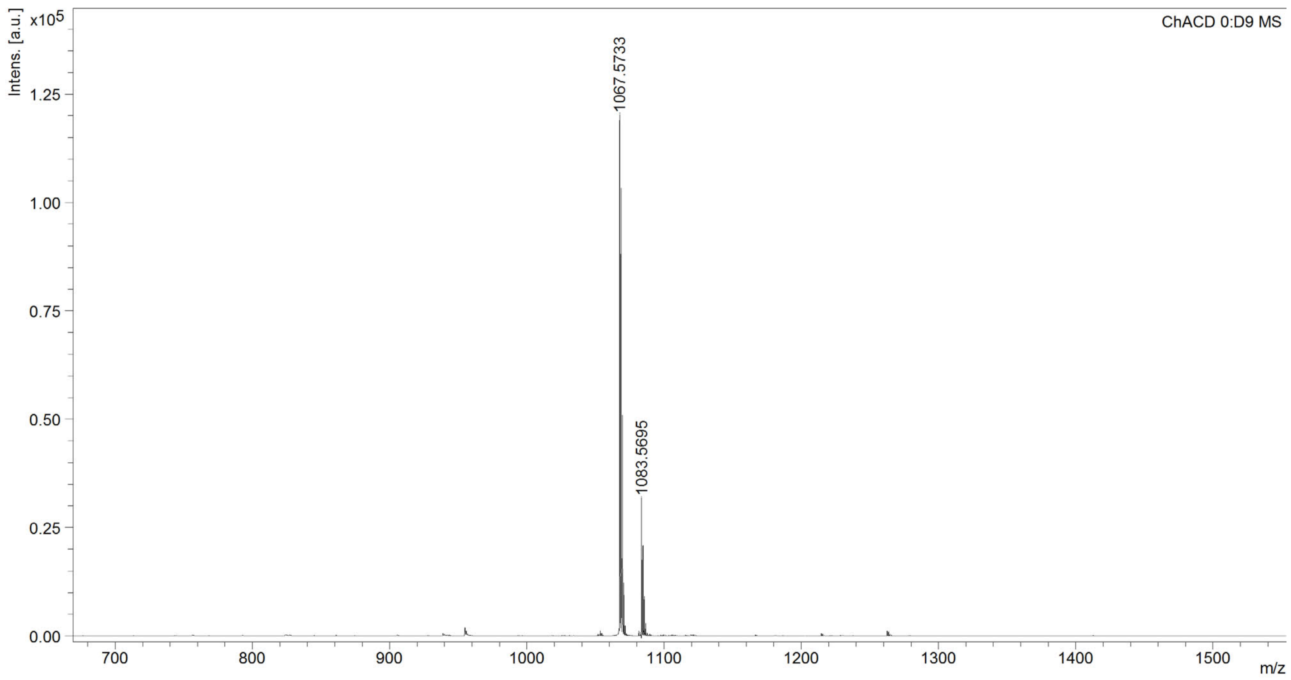

Figure S14.1: A) Analytical HPLC chromatogram of purified compound **7** at 70-100% MeOH/H_2O gradient and (B) the respective MALDI profile of the pure compound. Calculated MW: 1067.6623 [M+Na]; Observed MW: 1067.5733.

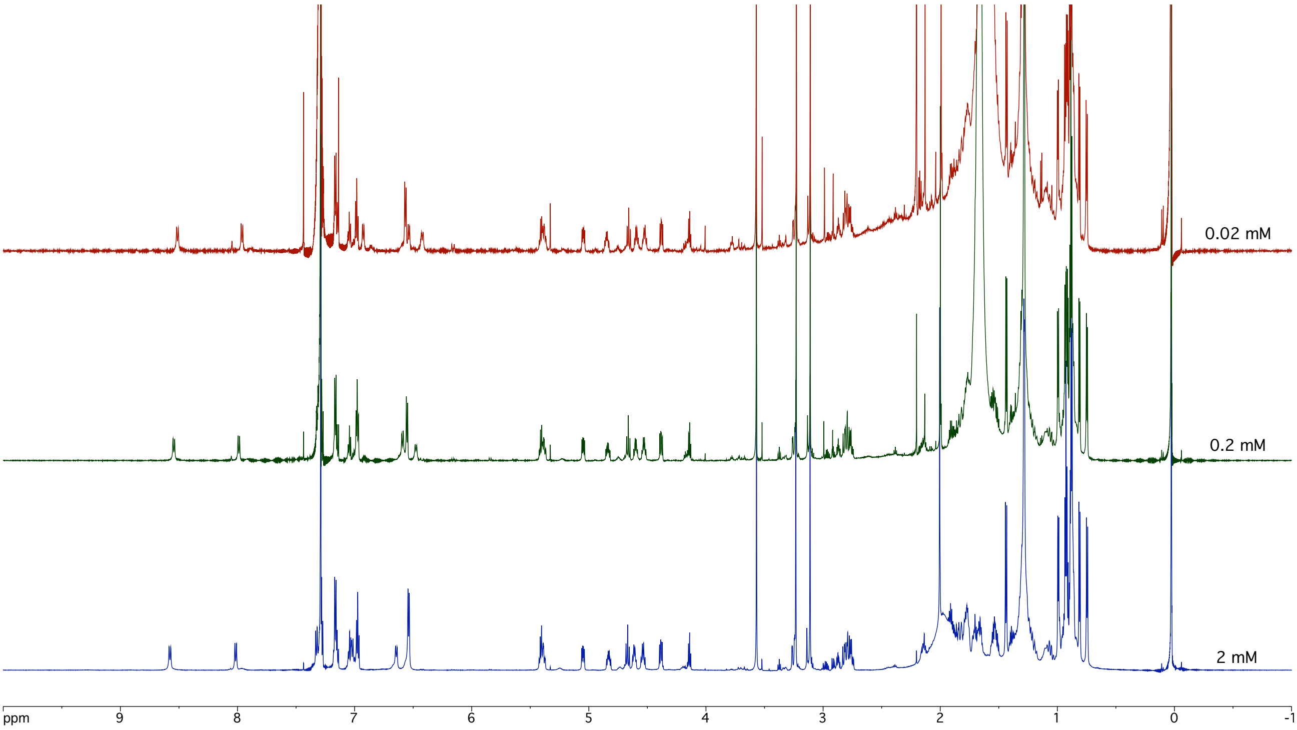

Figure S14.2: ^{1}H NMR spectra of Compound **14** at three different dilutions in $CDCl_3$ at 25°C.

Table 14.1: Chemical shifts table.

Residue	Atoms										
	HN	NMe	HA	HB		HG		HD		OMe	NAc
				1	2	1	2	1	2		
**LEU1**	7.07		4.6	1.53	1.39			0.88			1.98
**PHE2**	6.61		5.38	3.25	2.82						
**VAL3**	8.61		4.67	2.14		0.92	0.88				
**D-CHA4**		3.24	5.05	1.86	1.55						
**ALA5**		3.11	5.41	1.44							
**LEU6**	7.34		4.54	1.78	1.69	1.65		0.99	0.93		
**PHE7**	6.74		4.82	2.77							
**VAL8**	8.04		4.39	1.91		0.74	0.81			3.54	

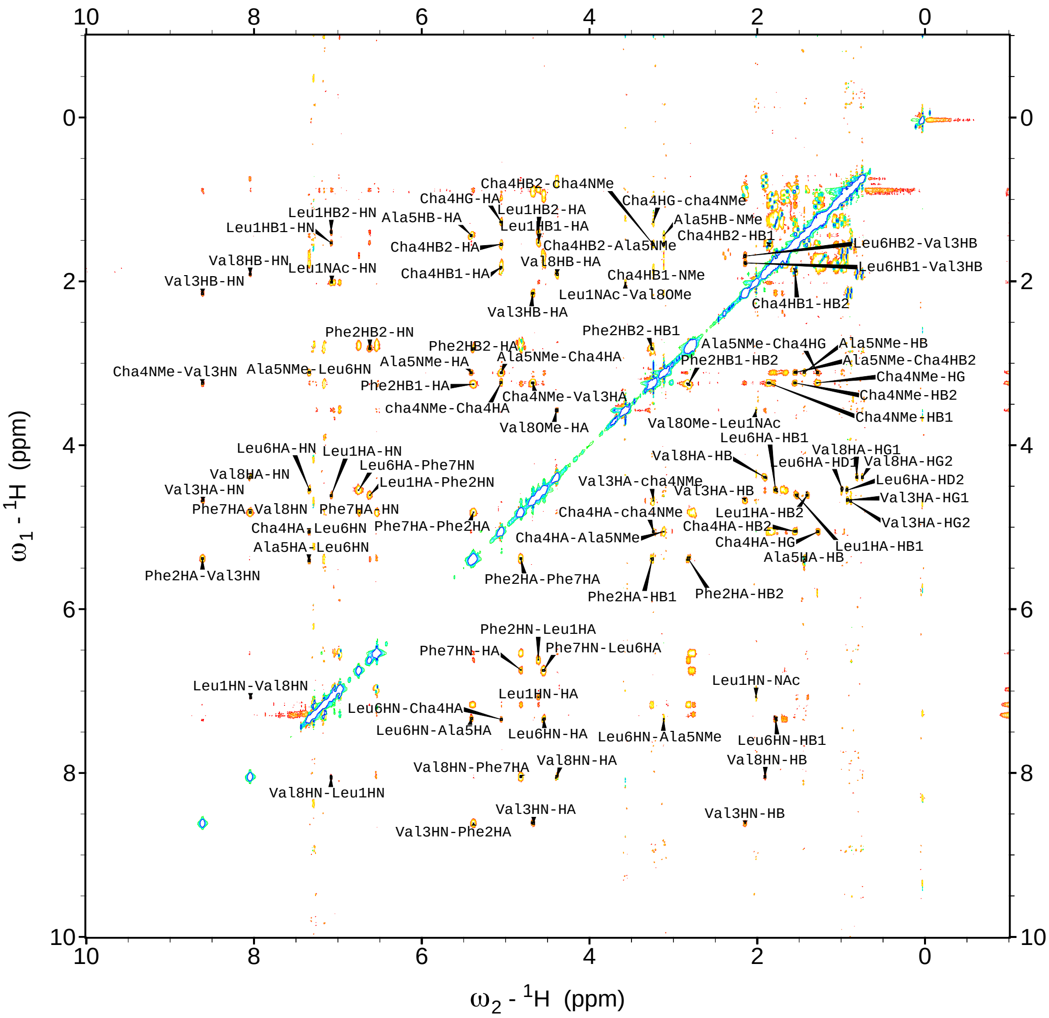

Figure S14.3: ROESY spectra with assigned peaks.

Table14.2: List of ROEs with respective NMR distances and violations.

Interactions	NMR Distance	Lower Limit	Upper Limit	Observed Distance	Violations
**Leu1HN-HA**	3.35	3.02	3.69	2.88	-0.1
**Leu1HA-Phe2HN**	2.78	2.50	3.06	2.81	0
**Leu1HB1-HA**	2.80	2.52	3.08	2.49	0
**Leu1HB1-HN**	3.62	3.26	3.98	3.81	0
**Leu1HB2-HA**	2.92	2.63	3.21	2.65	0
**Leu1HB2-HN**	3.28	2.95	3.61	3.37	0
**Leu1NAc-HN**	2.62	2.36	3.28	2.75	0
**Leu1NAc-Val8OMe**	2.95	2.66	4.05	3.71	0
**Phe2HA-Val3HN**	2.63	2.37	2.89	2.19	-0.2
**Phe2HB1-HA**	2.49	2.24	2.74	2.61	0
**Phe2HB2-HA**	2.73	2.46	3.00	2.97	0
**Phe2HB2-HB1**	1.80	1.62	1.98	1.74	0
**Phe2HB2-HN**	2.92	2.63	3.21	2.99	0
**Val3HA-HG1**	2.63	2.37	2.89	3.07	0.2

***Val3HA-HG2**	2.28	2.05	2.51	3.10	0.6
**Val3HA-HN**	3.31	2.98	3.64	2.95	0
***Val3HA-cha4NMe**	2.01	1.81	2.21	2.72	0.5
**Val3HB-HA**	2.78	2.50	3.06	3.06	0
**Val3HB-HN**	3.17	2.85	3.49	2.85	0
**cha4HA-Leu6HN**	3.20	2.88	3.52	3.39	0
**cha4HB1-HA**	2.13	1.92	2.34	2.56	0.2
**cha4HB2-HA**	2.56	2.30	2.82	3.00	0.2
**cha4HB2-HB1**	1.89	1.70	2.08	1.67	0
**cha4HB2-NMe**	3.46	3.11	3.81	2.79	-0.3
**cha4HG-HA**	2.94	2.65	3.23	2.38	-0.3
**cha4HG-NMe**	2.62	2.36	2.88	3.16	0.3
**cha4NMe-Val3HA**	1.98	1.78	2.58	2.72	0.1
**cha4NMe-Val3HN**	3.08	2.77	3.79	3.89	0.1
**cha4NMe-HB2**	2.90	2.61	3.59	2.79	0
**cha4NMe-HG**	2.58	2.32	3.24	3.16	0
**cha4NMe-HA**	2.85	2.57	3.54	3.62	0.1
**Ala5HA-Leu6HN**	2.95	2.66	3.25	2.99	0
**Ala5HB-HA**	2.42	2.18	3.06	2.46	0
**Ala5HB-NMe**	2.39	2.15	3.43	3.38	0
**Ala5NMe-cha4HA**	1.96	1.76	2.56	2.49	0
**Ala5NMe-HA**	3.12	2.81	3.83	3.76	0
**Ala5NMe-Leu6HN**	2.71	2.44	3.38	3.70	0.3
**Leu6HA-HB1**	2.73	2.46	3.00	2.98	0
**Leu6HA-HD1**	2.74	2.47	3.01	3.10	0.1
**Leu6HA-HD2**	2.59	2.33	2.85	3.21	0.4
**Leu6HA-HN**	2.92	2.63	3.21	2.94	0
**Leu6HA-Phe7HN**	2.31	2.08	2.54	2.42	0
**Leu6HB1-Val3HB**	2.90	2.61	3.19	3.11	0
**Leu6HN-HB1**	3.01	2.71	3.31	2.62	-0.1
**Leu6HB2-Val3HB**	2.82	2.54	3.10	2.82	0
**Phe7HA-Phe2HA**	2.38	2.14	2.62	2.67	0
**Phe7HA-HN**	3.03	2.73	3.33	2.88	0
**Phe7HA-Val8HN**	2.36	2.12	2.60	2.43	0
**Val8HA-HG1**	2.93	2.64	3.22	3.00	0
***Val8HA-HG2**	2.98	2.68	3.28	3.82	0.5
**Val8HA-HN**	3.10	2.79	3.41	2.84	0
**Val8HB-HA**	2.62	2.36	2.88	2.56	0
**Val8HB-HN**	4.00	3.60	4.40	3.76	0
***Val8OMe-HA**	3.04	2.74	3.74	4.35	0.6
**Leu1HN-Val8HN**	3.53	3.18	3.88	2.73	-0.4

*violations ≥0.5. The observed high violations can be explained by the local flexibility about the γ and δ methyl groups (Val and Leu respectively) and the terminal ester bond, peak overlap, additional J-mediated transfer and inaccuracies in the force fields.[9]

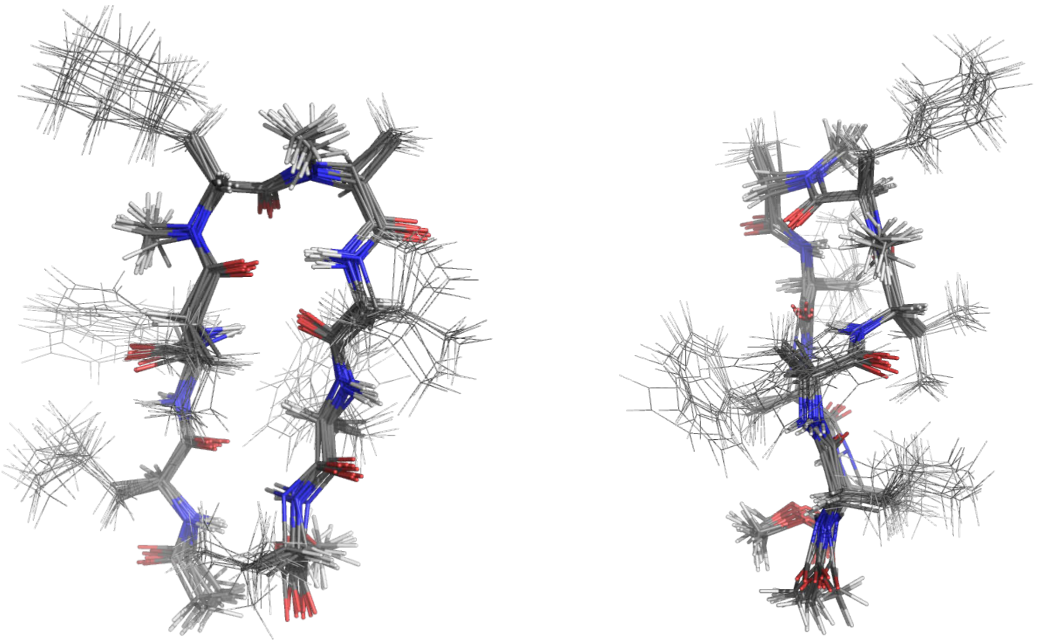

Figure S14.4: Overlay of 10 representative conformations generated using Molecular Dynamics simulation, showing both front view (left panel) and side view (right panel).

(A)

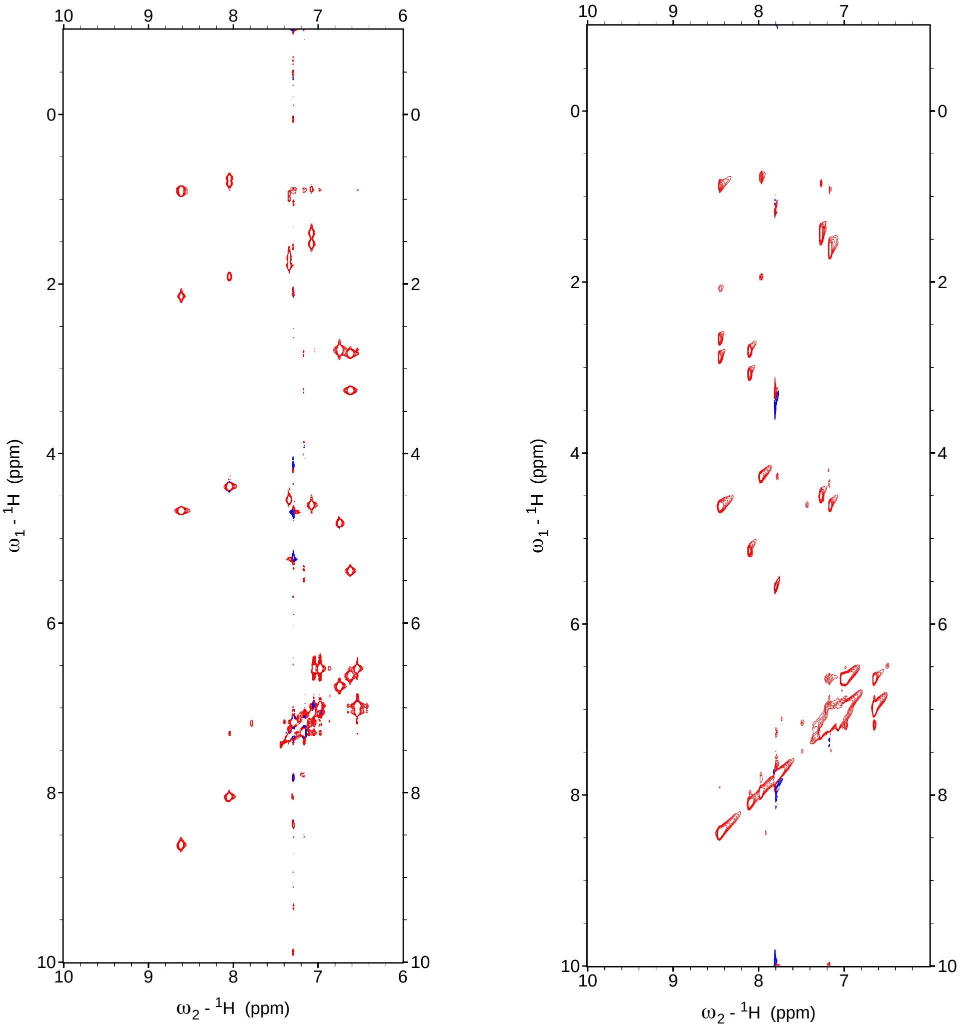

$CDCl_3$

DMSO-d_6 – $CDCl_3$ (1:2)

(B)

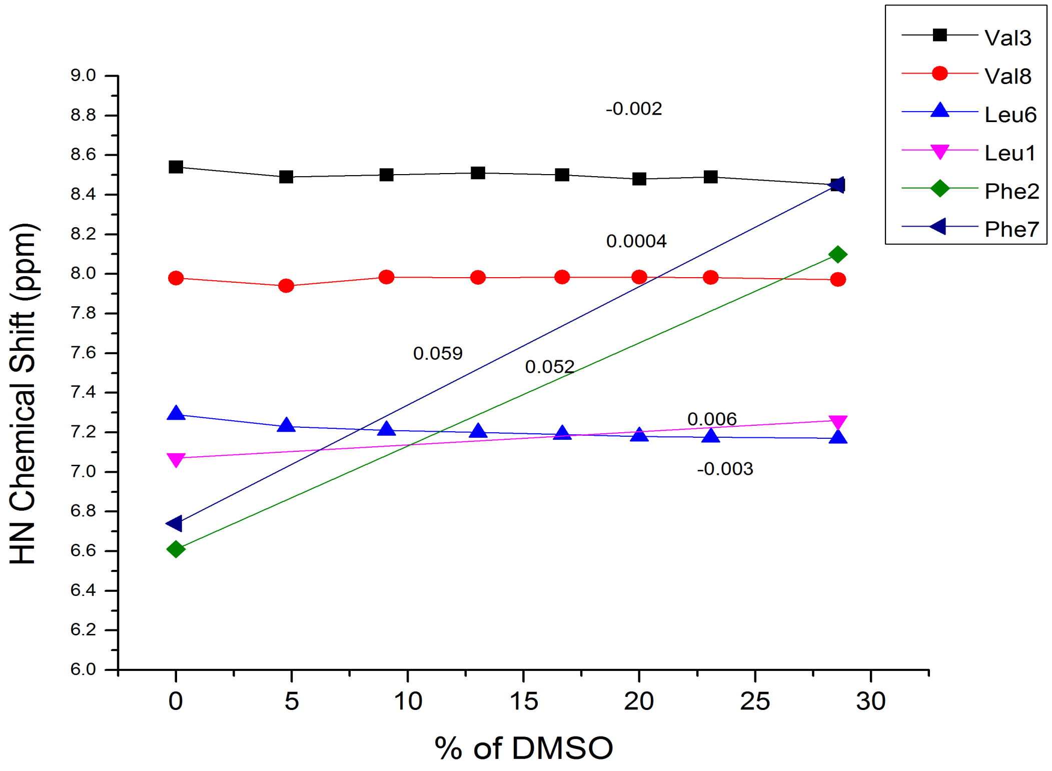

Figure S14.5: (A) TOCSY spectrum in $CDCl_3$ and DMSO-d_6 – $CDCl_3$ (1:2) and (B) DMSO-d_6 titration curve indicating the solvent exposed (F2, F7) and solvent shielded (L1, V3, L6, V8) amide protons. The value indicates the slope generated by the linear fit of the data points.

Compound 15:

(A)

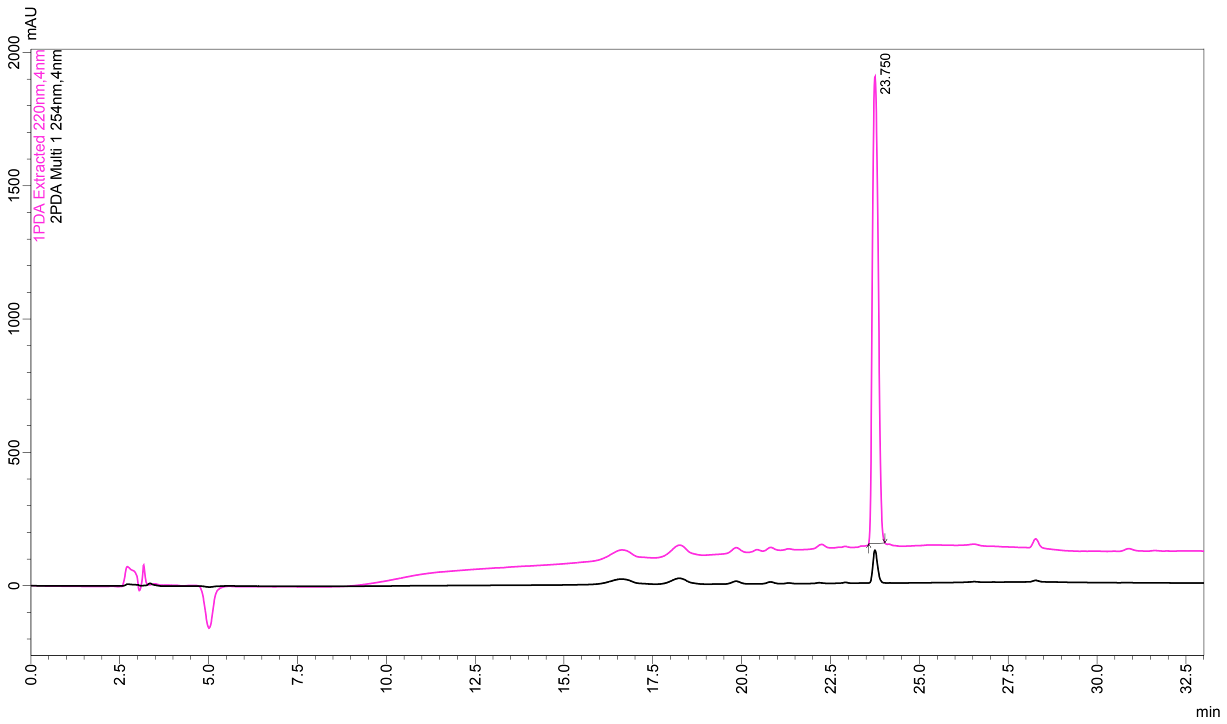

(B)

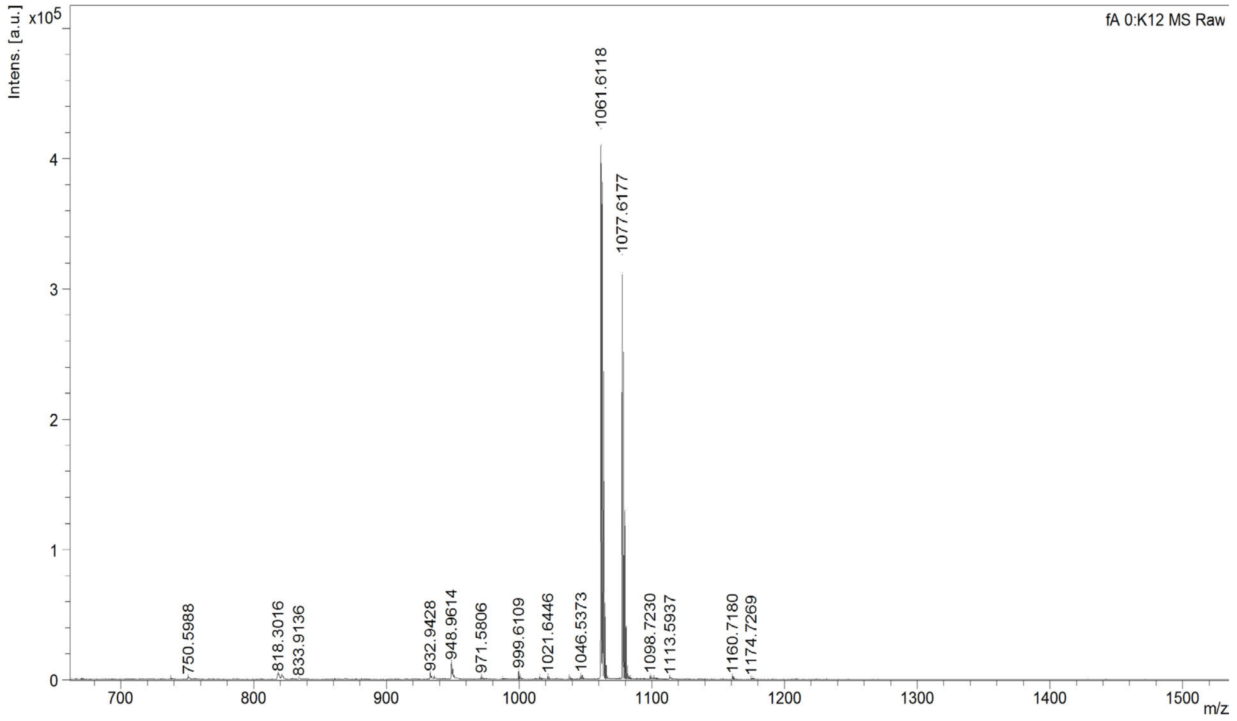

Figure S15.1: A) Analytical HPLC chromatogram of purified compound **15** at 70-100% MeOH/H_2O gradient and (B) the respective MALDI profile of the pure compound. Calculated MW: 1061.6154 [M+Na]; Observed MW: 1061.6118.

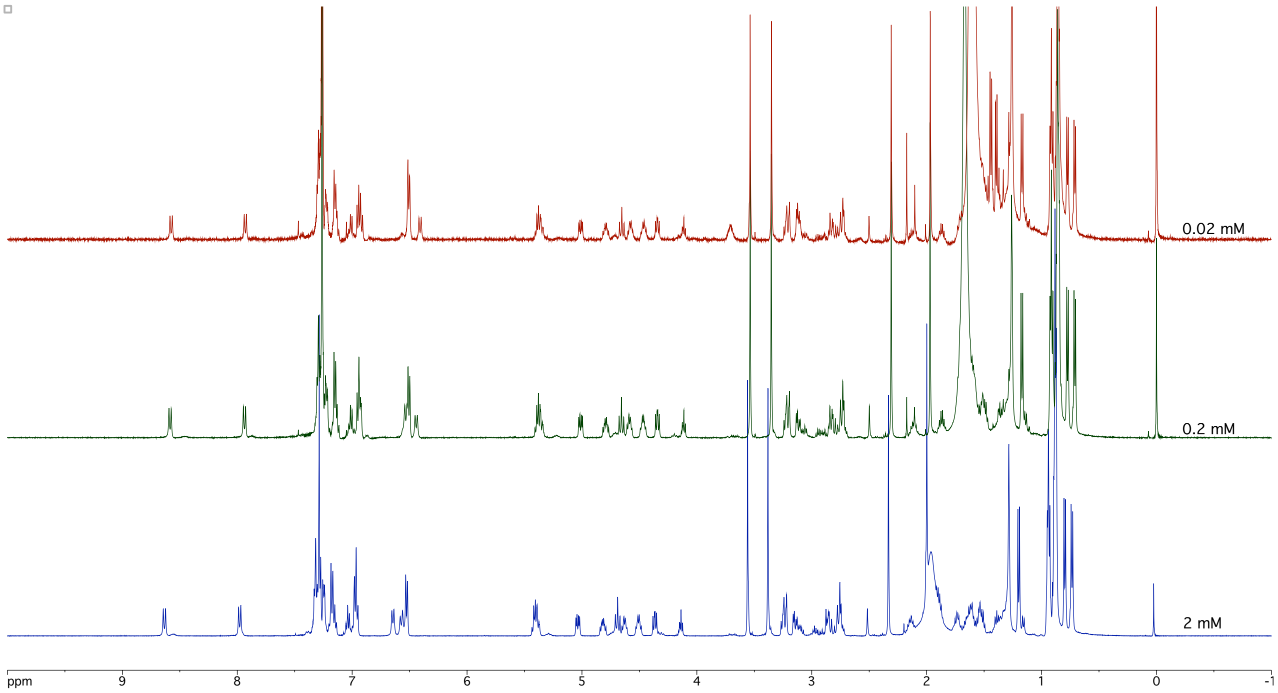

Figure S15.2: ^{1}H NMR spectra of Compound **15** at three different dilutions in $CDCl_3$ at 25°C.

Table 15.1: Chemical shifts table.

Residue	Atoms										
	HN	NMe	HA	HB		HG		HD		OMe	NAc
				1	2	1	2	1	2		
**LEU1**	6.96		4.58	1.5	1.37			0.86			1.97
**PHE2**	6.44		5.36	3.21	2.82						
**VAL3**	8.6		4.67	2.11		0.91	0.85				
**D-PHE4**		3.35	5.01	3.22	3.12						
**Ala5**		2.31	5.38	1.17							
**LEU6**	7.25		4.47	1.71	1.59			0.92	0.86		
**PHE7**	6.56		4.8								
**VAL8**	7.94		4.34	1.87		0.77	0.71			3.53	

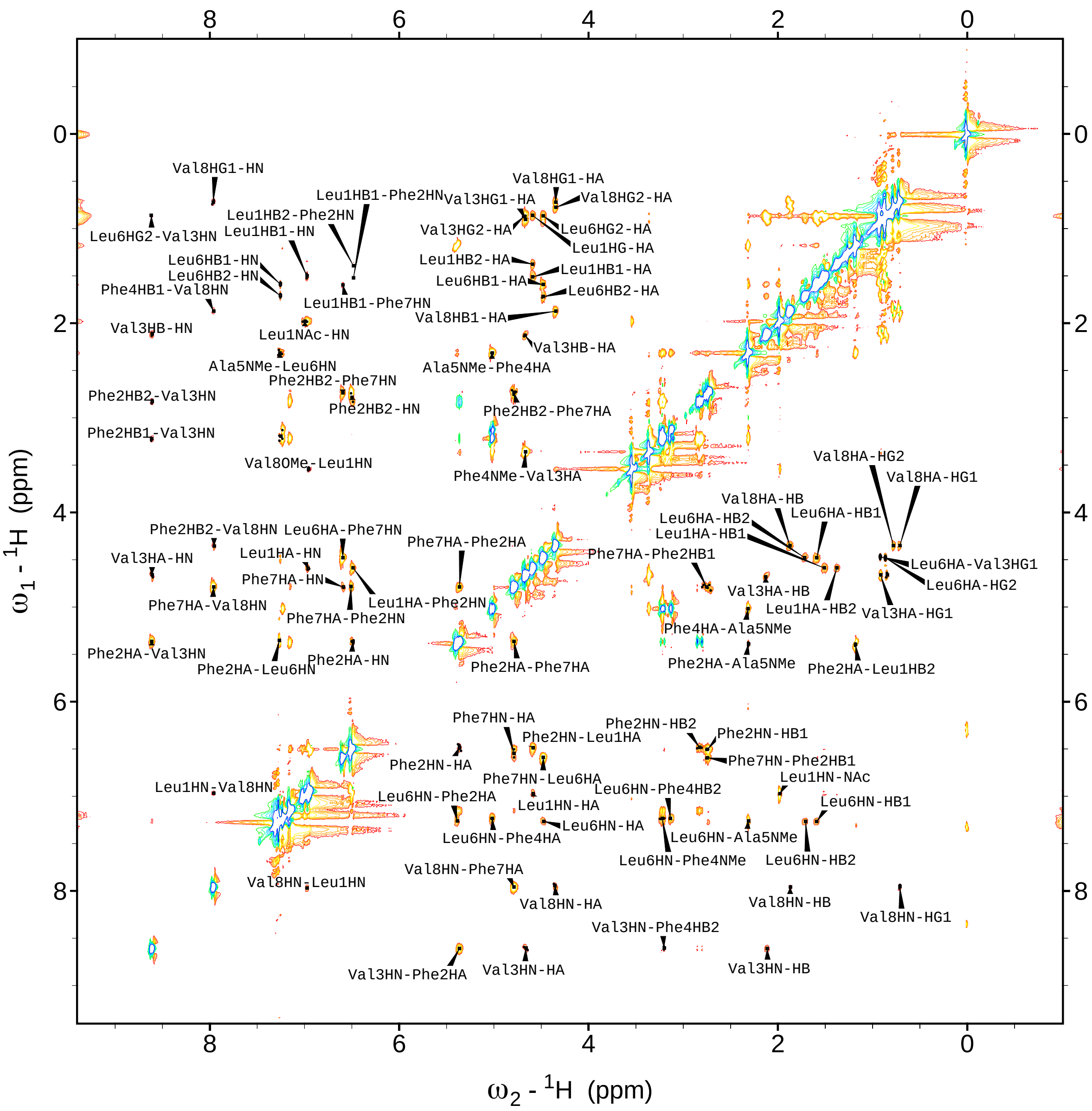

Figure S15.3: ROESY spectra with assigned peaks.

Table15.2: List of ROEs with respective NMR distances and violations.

Interactions	NMR Distance	Lower Limit	Upper Limit	Observed Distance	Violations
Leu1HA-HN	2.97	2.67	3.27	2.81	0
Leu1HA-Phe2HN	2.46	2.21	2.71	2.41	0
Leu1HB1-HA	2.37	2.13	2.61	2.47	0
Leu1HB2-HA	2.48	2.23	2.73	2.77	0
Leu1HN-Val8HN	3.22	2.9	3.54	2.47	-0.4
Leu1NAc-HN	2.45	2.21	3.1	2.8	0
Leu1HB1-Phe2HN	2.95	2.66	3.25	3.05	0
Leu1HB2-Phe2HN	3.03	2.73	3.33	3.62	0.3
Leu1NAc-Val8OMe	2.9	2.61	3.99	3.71	0

**Phe2HA-Val3HN**	2.45	2.21	2.7	2.12	-0.1
**Phe2HB1-Val3HN**	2.97	2.67	3.27	3.21	0
**Phe2HB1-HB2**	1.95	1.76	2.15	1.73	0
**Val3HA-HN**	3.14	2.83	3.45	2.97	0
**Val3HB-HA**	2.79	2.51	3.07	3.05	0
**Val3HB-HN**	3.02	2.72	3.32	2.8	0
***phe4HA-NMe**	2.81	2.53	3.09	3.62	0.5
**phe4NMe-Val3HA**	2.05	1.85	2.66	2.79	0.1
***phe4HB1-NMe**	2.13	1.92	2.34	2.89	0.5
**phe4HA-HB1**	2.41	2.17	2.65	2.61	0
**Ala5HB-HA**	2.23	2.01	2.85	2.45	0
**Ala5HB-NMe**	2.44	2.2	3.48	3.39	0
**Ala5NMe-phe4HA**	2.27	2.04	2.9	2.6	0
**Ala5NMe-Leu6HN**	2.58	2.32	3.24	3.28	0
**Ala5NMe-HA**	3.1	3.1	2.79	3.81	0
**Ala5HA-Leu6HN**	2.64	2.38	2.9	3.02	0.1
**Leu6HA-HN**	3.25	2.93	3.58	2.96	0
**Leu6HA-Phe7HN**	2.27	2.04	2.5	2.46	0
***Leu6HB1-HA**	2.3	2.07	2.53	3.03	0.5
***Leu6HB1-HN**	3.41	3.07	3.75	2.61	-0.5
**Leu6HB2-HA**	2.31	2.08	2.54	2.54	0
**Leu6HB2-HN**	3.29	2.96	3.62	3.68	0.1
**Leu6HB1-Val3HB**	2.72	2.45	2.99	2.34	-0.1
**Leu6HB2-Val3HB**	2.81	2.53	3.09	3.23	0.1
**Leu6HN-Val3HN**	3.25	2.93	3.58	3.34	0
**Phe7HA-Phe2HA**	2.49	2.24	2.74	2.74	0
**Phe7HA-HN**	2.87	2.58	3.16	2.9	0
**Phe7HA-Val8HN**	2.3	2.07	2.53	2.3	0
**Val8HA-HG1**	2.79	2.51	3.07	2.97	0
**Val8HA-HG2**	2.92	2.63	3.21	3.44	0.2
**Val8HA-HN**	3.1	2.79	3.41	2.87	0
**Val8HB-HA**	2.6	2.34	2.86	2.88	0
**Val8HB-HN**	3.32	2.99	3.65	3.15	0
**Val8OMe-Leu1HN**	3.01	2.71	3.71	3.12	0

* violations ≥0.5. The observed high violations can be explained by the local flexibility about the γ and δ methyl groups (Val and Leu respectively) and the terminal ester bond, peak overlap, additional J-mediated transfer and inaccuracies in the force fields.[9]

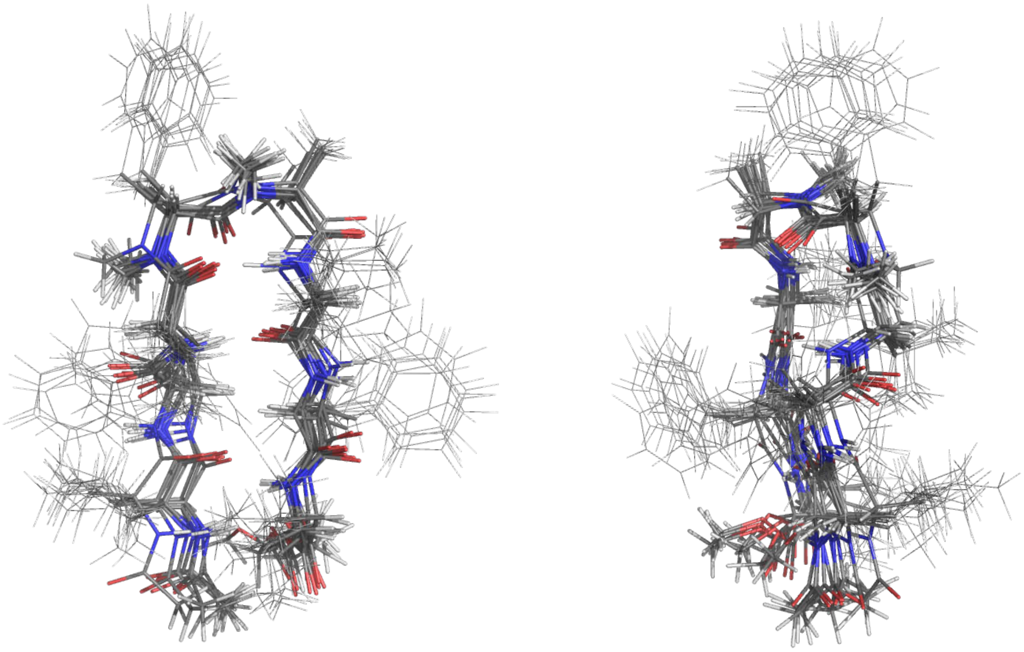

Figure S15.4: Overlay of 10 representative conformations generated using Molecular Dynamics simulation, showing both front view (left panel) and side view (right panel).

(A)

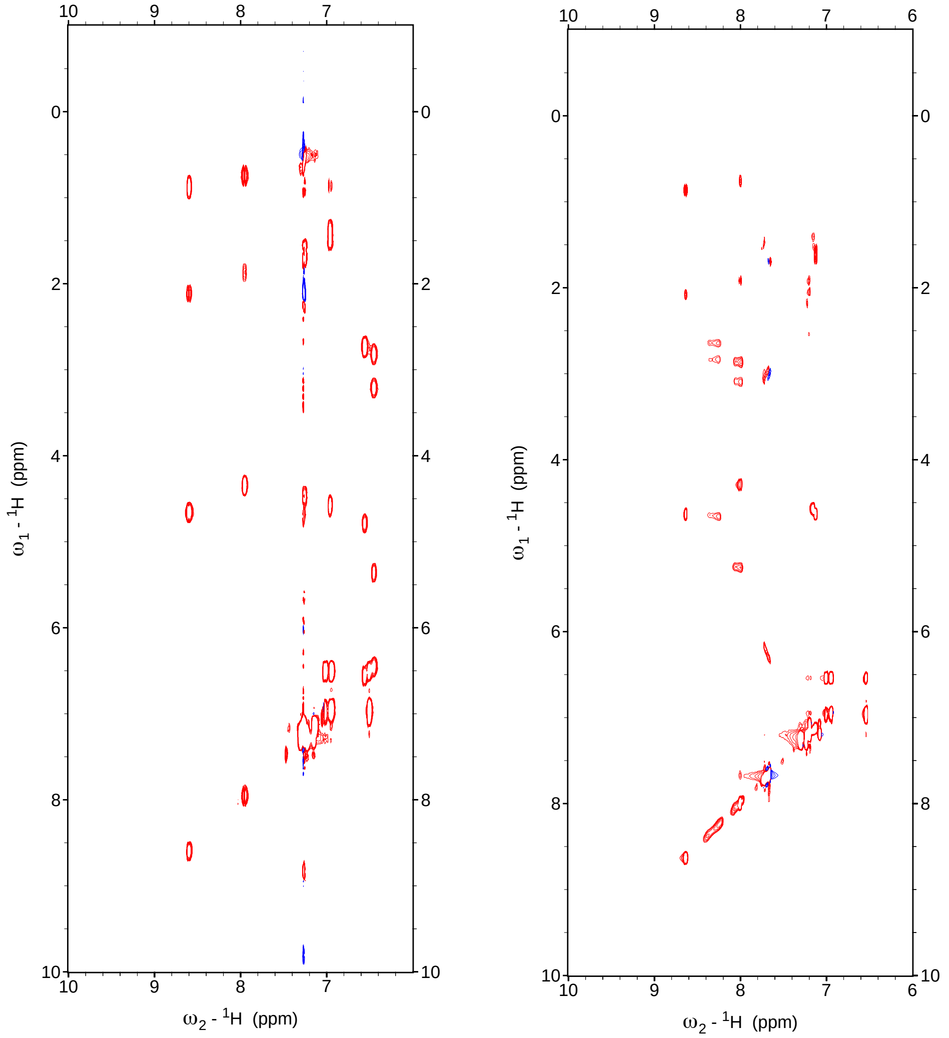

$CDCl_3$

DMSO-d_6 – $CDCl_3$ (1:2)

(B)

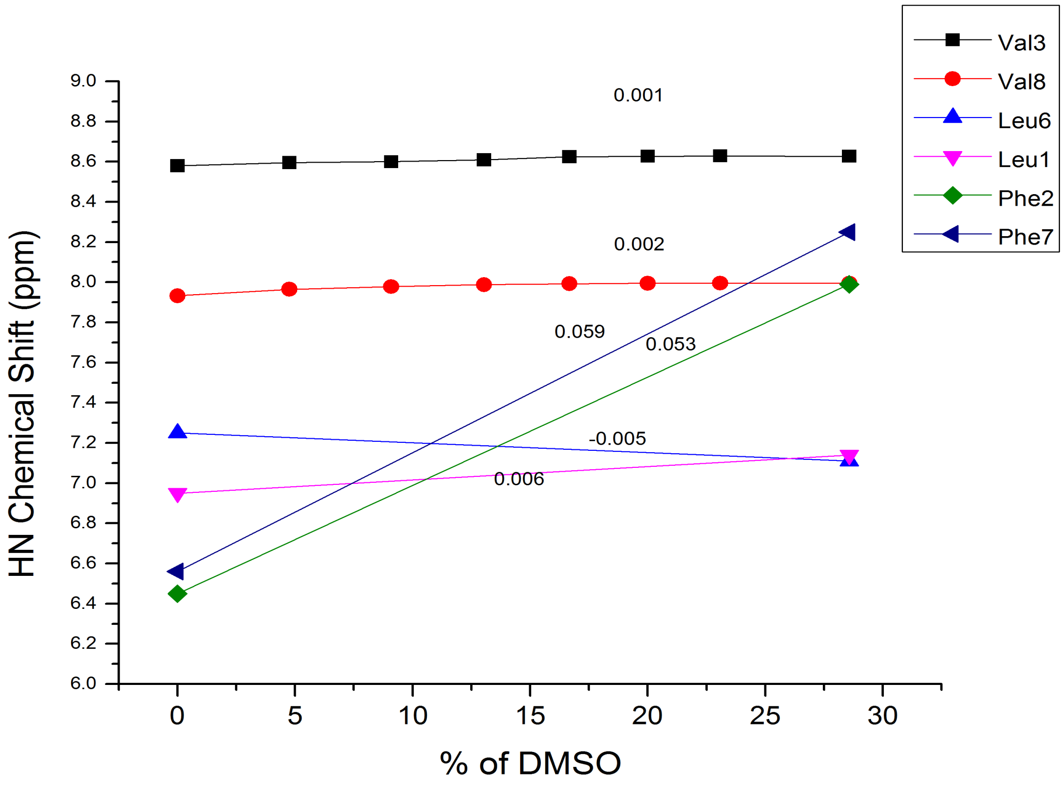

Figure S15.5: (A) TOCSY spectrum in $CDCl_3$ and DMSO-d_6 – $CDCl_3$ (1:2) and (B) DMSO-d_6 titration curve indicating the solvent exposed (F2, F7) and solvent shielded (L1, V3, L6, V8) amide protons. The value indicates the slope generated by the linear fit of the data points.

Compound 16:

(A)

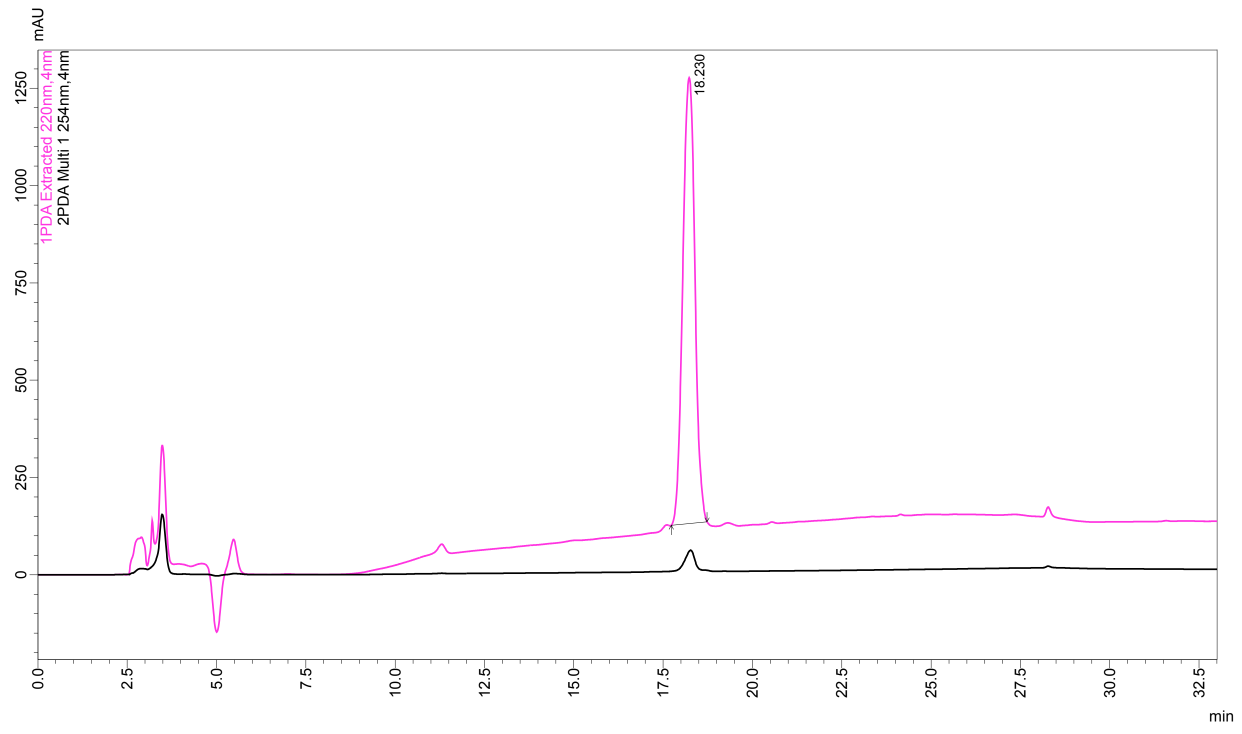

(B)

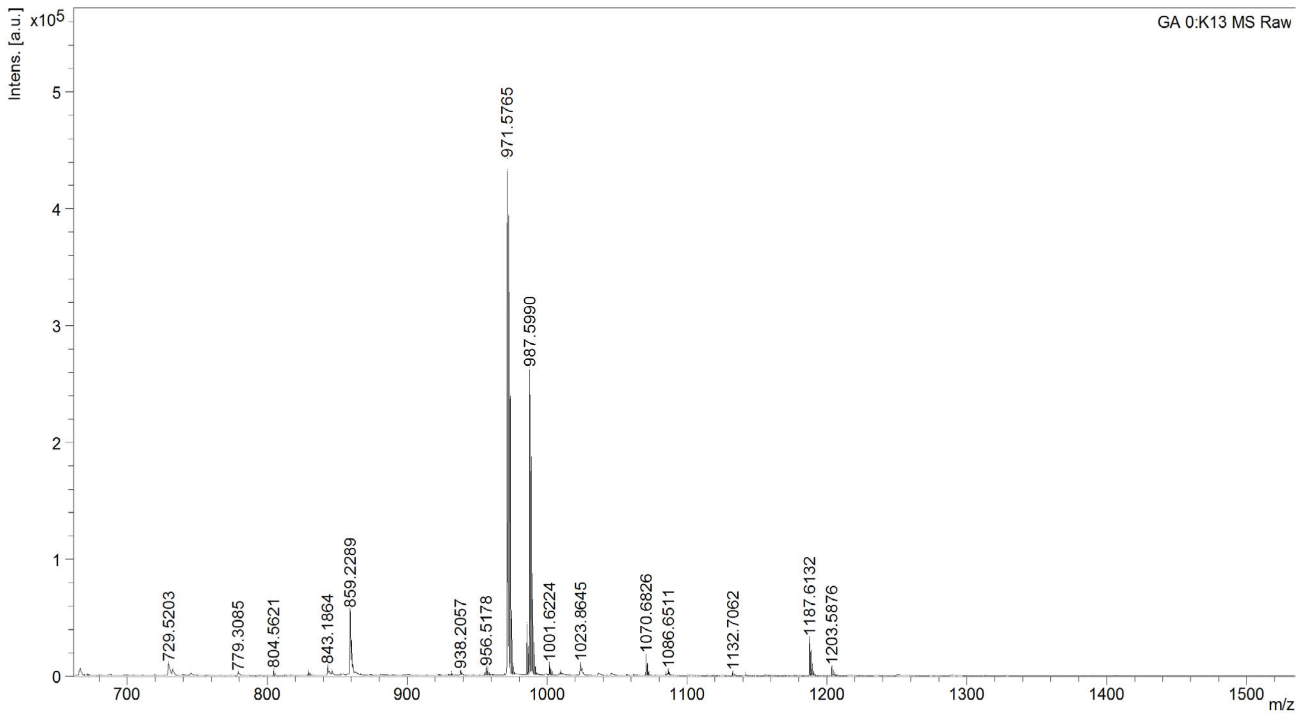

Figure S16.1: A) Analytical HPLC chromatogram of purified compound **16** at 70-100% MeOH/H_2O gradient and (B) the respective MALDI profile of the pure compound. Calculated MW: 971.5684 [M+Na]; Observed MW: 971.5765.

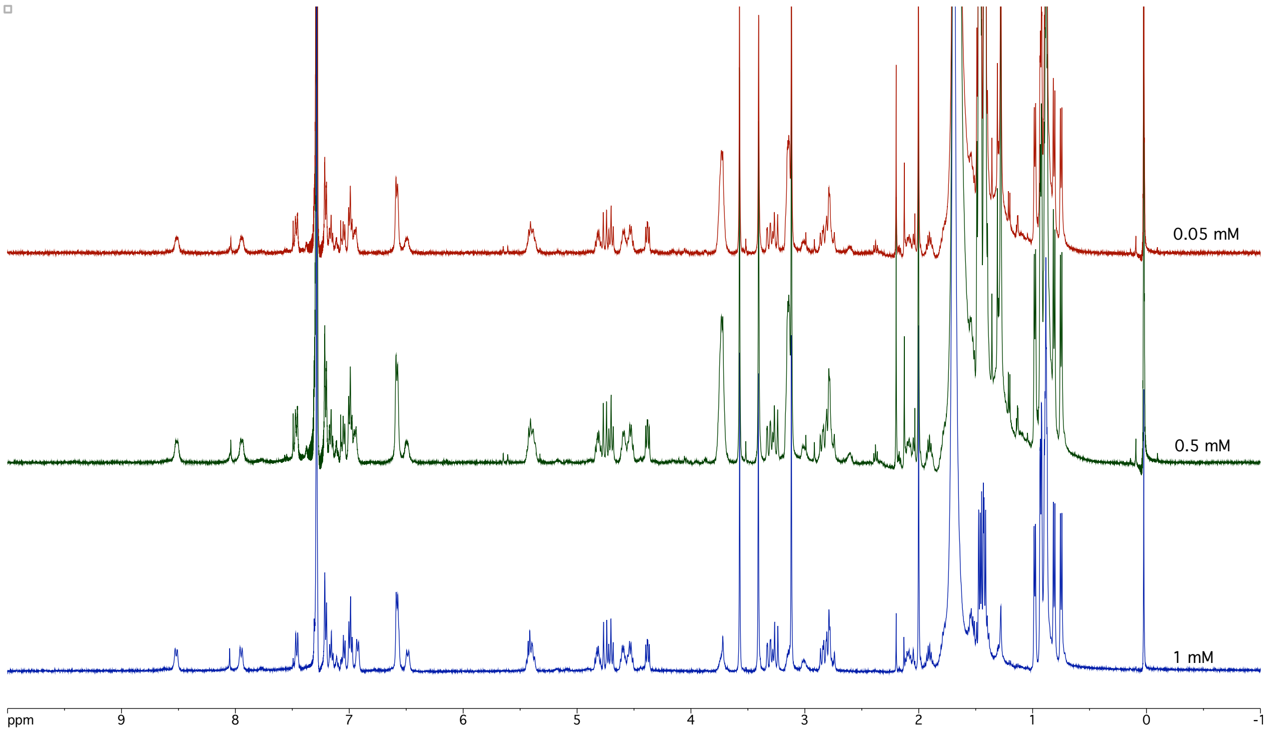

Figure S16.2: ^{1}H NMR spectra of Compound **16** at three different dilutions in $CDCl_3$ at 25°C.

Table 16.1: Chemical shifts table.

**Residue**	**Atoms**											
	**HN**	**NMe**	**HA**		**HB**		**HG**		**HD**		**OMe**	**NAc**
			1	2	1	2	1	2	1	2		
**LEU1**	6.98		4.57		1.5	1.38			0.84			1.98
**PHE2**	6.53		5.36		2.82	3.3						
**VAL3**	8.51		4.68		2.07		0.91	0.87				
**GLY4**		3.38	3.23	4.73								
**ALA5**		3.09	5.39		1.42							
**LEU6**	7.46		4.52		1.76	1.65			0.95	0.9		
**PHE7**	6.6		4.79		2.77							
**VAL8**	7.93		4.35		0.89		0.78	0.72			3.55	

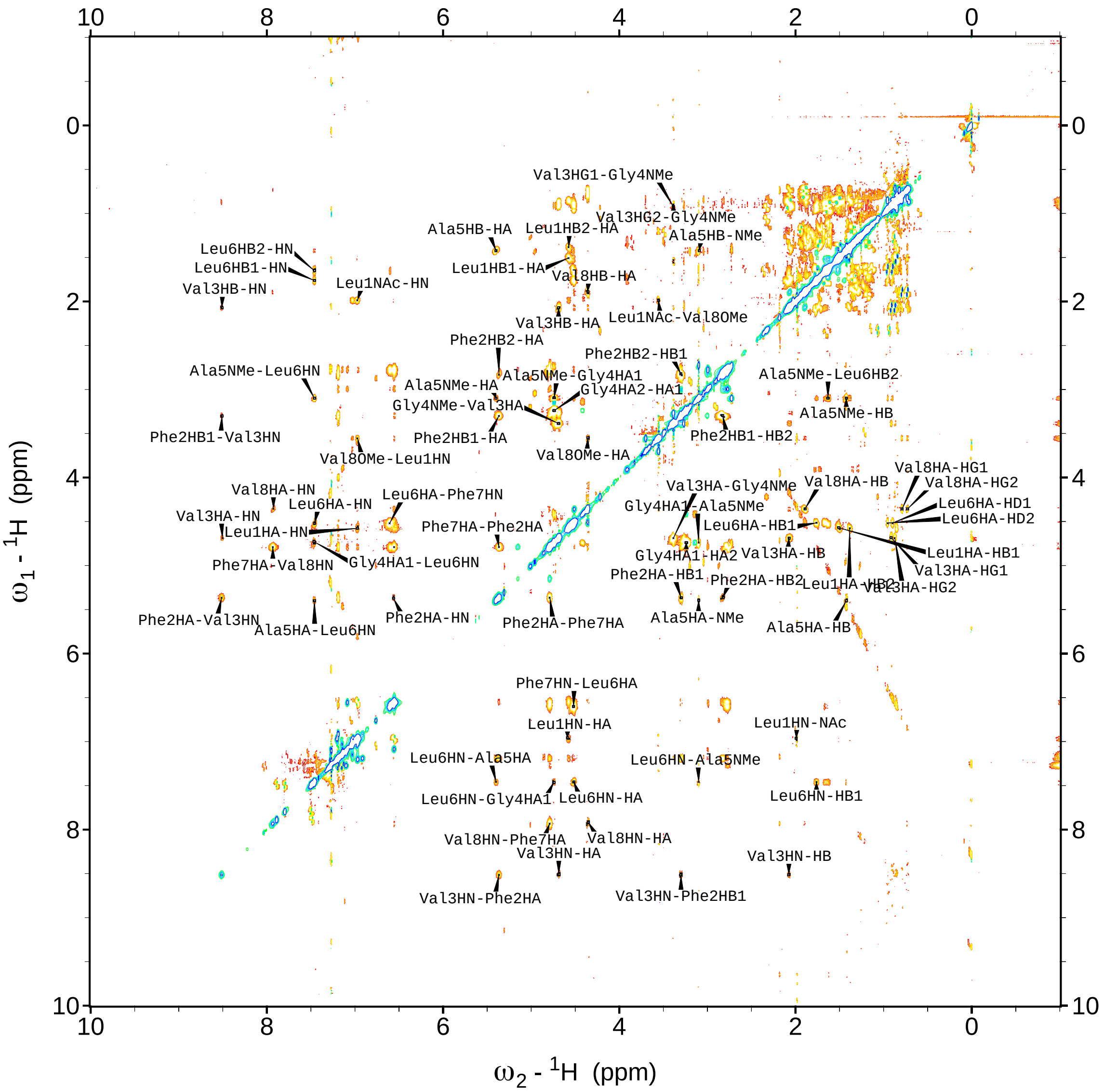

Figure S16.3: ROESY spectra with assigned peaks.

Table16.2: List of ROEs with respective NMR distances and violations.

Interactions	NMR Distance	Lower Limit	Upper Limit	Observed Distance	Violations
**Leu1HB1-HA**	2.62	2.36	2.88	2.80	0
**Leu1HB2-HA**	2.81	2.53	3.09	2.82	0
**Leu1HN-HA**	2.82	2.54	3.10	2.93	0
**Leu1NAc-HN**	2.35	2.12	2.99	2.70	0
**Leu1HB1-HN**	3.44	3.10	3.78	3.04	-0.1
**Leu1HB2-HN**	3.83	3.45	4.21	3.40	-0.1
**Leu1NAc-Val8OMe**	2.85	2.57	3.94	3.65	0
**Phe2HB1-HA**	2.63	2.37	2.89	2.59	0
**Phe2HB2-HA**	3.15	2.84	3.47	2.71	-0.1
***Phe2HA-HN**	3.98	3.28	4.18	2.72	-0.6

**Phe7HA-Phe2HA**	2.56	2.30	2.82	2.50	0
**Phe2HB1-HN**	3.30	2.97	3.63	3.64	0
**Phe2HB1-HB2**	1.80	1.62	1.98	1.73	0
***Val3HN-Phe2HA**	3.05	2.75	3.36	2.20	-0.5
**Val3HN-Phe2HB1**	3.81	3.43	4.19	3.88	0
**Val3HN-HB**	3.16	2.84	3.48	2.82	0
**Val3HB-HA**	2.65	2.39	2.92	3.04	0.1
**Val3HA-HG1**	2.66	2.39	2.93	3.02	0.1
**Val3HA-HG2**	2.69	2.42	2.96	3.14	0.2
**Val3HB-Leu6HB1**	2.73	2.46	3.00	3.17	0.2
**Val3HB-Leu6HB2**	2.57	2.31	2.83	2.69	0
**Val3HN-HA**	3.78	3.40	4.16	2.97	-0.4
**Gly4NMe-Val3HA**	2.01	1.81	2.61	2.73	0.1
**Gly4HA2-HA1**	1.80	1.62	1.98	1.73	0
**Ala5NMe-Gly4HA1**	2.11	1.90	2.72	2.65	0
**Leu6HN-Gly4HA1**	3.48	3.13	3.83	3.90	0.1
**Ala5HB-HA**	2.63	2.37	3.29	2.45	0
**Ala5NMe-HA**	3.47	3.12	4.22	3.78	0
**Leu6HN-Ala5HA**	3.47	3.12	3.82	3.00	-0.1
**Ala5NMe-HB**	2.37	2.13	3.41	3.38	0
**Leu6HN-Ala5NMe**	2.91	2.62	3.20	3.43	0.2
**Leu6HA-HB1**	2.73	2.46	3.00	2.87	0
**Leu6HN-HB1**	3.13	2.82	3.44	2.80	0
**Leu6HN-HA**	2.89	2.60	3.18	2.91	0
**Phe7HN-Leu6HA**	2.24	2.02	2.46	2.40	0
**Phe7HN-HA**	2.54	2.29	2.79	2.92	0.1
**Val8HN-Phe7HA**	2.58	2.32	2.84	2.27	0
**Val8HB-HA**	2.57	2.31	2.83	2.51	0
**Val8HA-HG1**	2.90	2.61	3.19	2.99	0
**Val8HA-HG2**	3.16	2.84	3.48	3.86	0.4
**Val8HN-HA**	3.18	2.86	3.50	2.85	0
**Val8OMe-HA**	3.33	3.00	4.06	4.30	0.2
***Val8HB-HN**	4.81	4.33	5.29	3.83	-0.5
**Val8OMe-Leu1HN**	3.16	2.84	3.88	3.67	0

* violations ≥0.5. The observed high violations can be explained by the local flexibility about the γ and δ methyl groups (Val and Leu respectively) and the terminal ester bond, peak overlap, additional J-mediated transfer and inaccuracies in the force fields.[9]

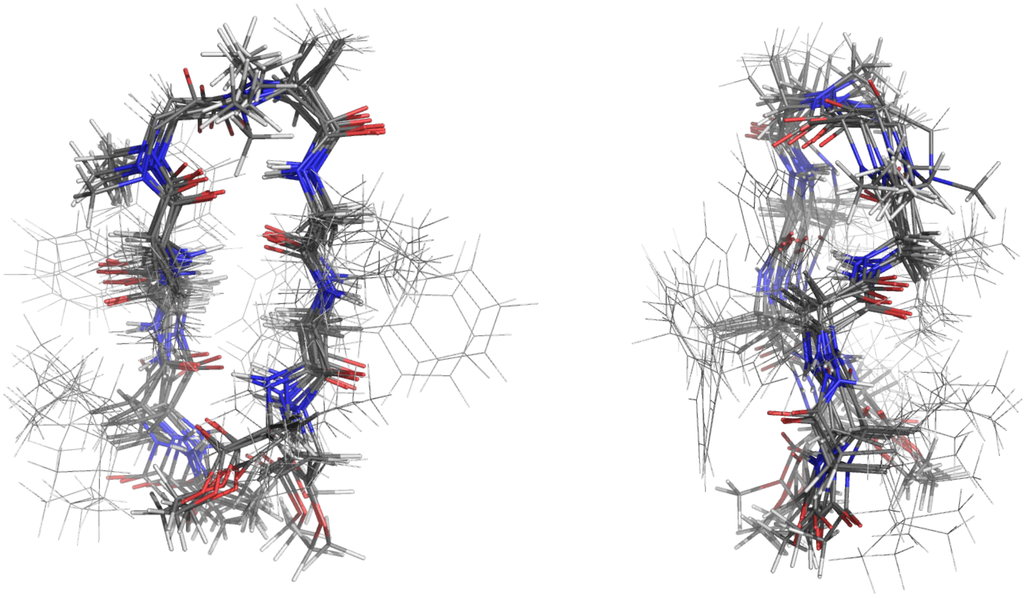

Figure S16.4: Overlay of 10 representative conformations generated using Molecular Dynamics simulation, showing both front view (left panel) and side view (right panel).

(A)

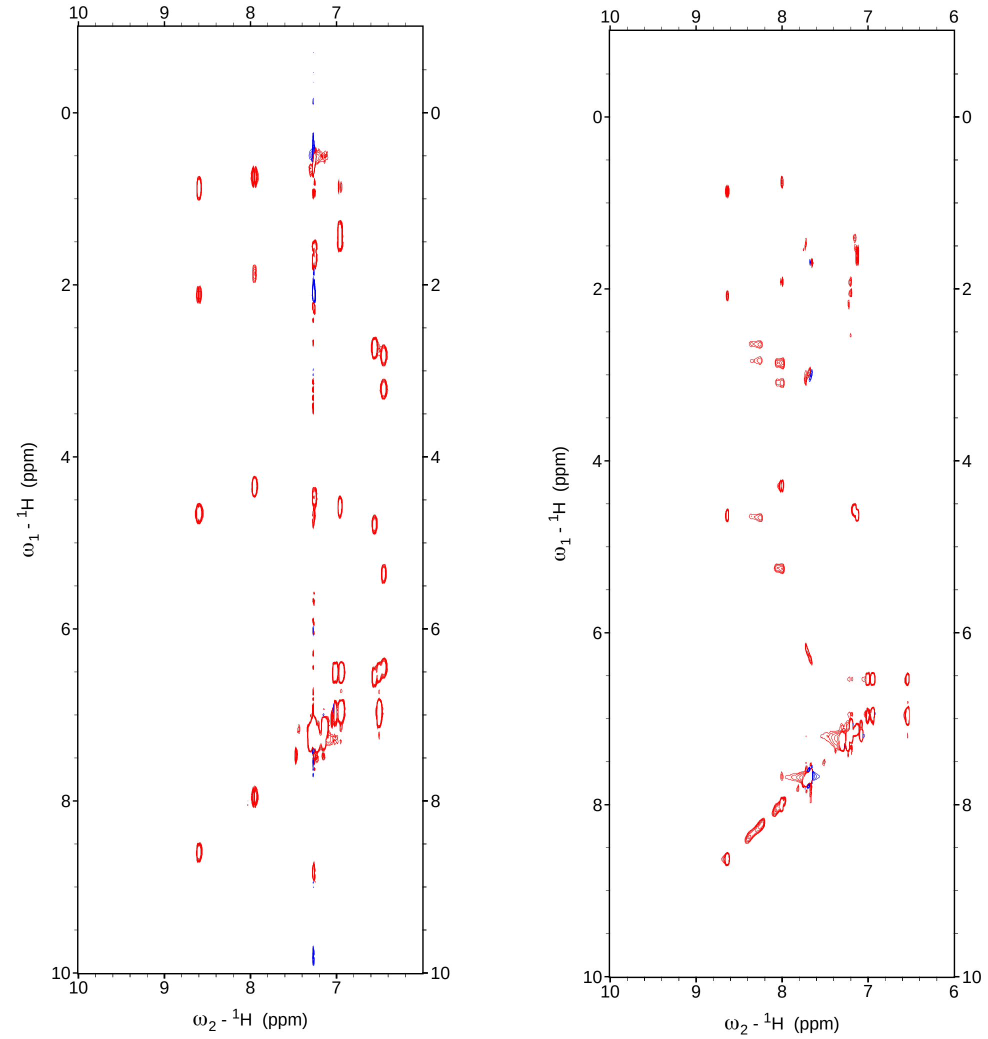

$CDCl_3$

DMSO-d_6 – $CDCl_3$
(1:2)

(B)

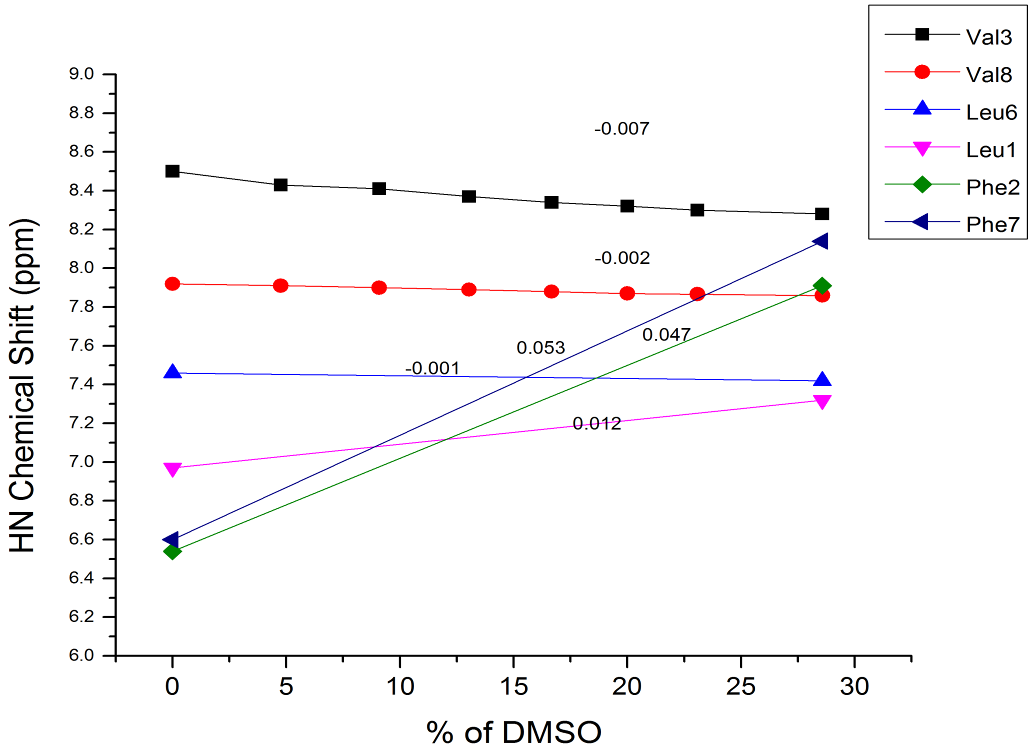

Figure S16.5: (A) TOCSY spectrum in $CDCl_3$ and DMSO-d_6 – $CDCl_3$ (1:2) and (B) DMSO-d_6 titration curve indicating the solvent exposed (F2, F7) and solvent shielded (V3, L6, V8) amide protons. The value indicates the slope generated by the linear fit of the data points.

Table S1: Coupling constants of compounds **1** to **16**

	$^3J_{H^N\text{-}H^\alpha}$ Coupling Constants (Hz)					
**Comp.**	**Leu1**	**Phe2**	**Val3**	**Leu6**	**Phe7**	**Val8**
**1**	7.8	8.1	9.1	8.3	8.3	9.2
**2**		9.2	9.8	8.9	8.7	9.6
**3**		9.2	9.7	9	8.7	9.4
**4**		8.4	9.4	8.2		9.5
**5**			8.7		8.2	8.9
**6**	7.5	7.3	9.8	9	8.8	9.3
**7**	8.3	7.6	9.5	9	8.8	9.5
**8**	8.3		8.3	8.8	8.1	9
**9**			9.8	9		9.7
**10**	8.4	9	9.5	8.8		9.4
**11**	7.9	9.4	10.3	7.3	9	9.3
**12**	8.3	9.3	9.8		9.1	9.5
**13**			9.7	8.4	8.1	9
**14**	8.3		8.4	9.1	7.9	9.4
**15**		9.6	9		8.7	9.2
**16**			9.2	8.8	9	9.3

Table S2: DMSO titration slope of each residue for compounds **1** to **16**

Slope Obtained From DMSO Titration						
**Comp.**	**Leu1**	**Phe2**	**Val3**	**Leu6**	**Phe7**	**Val8**
**1**	0.005	0.053	0	-0.002	0.048	0.001
**2**	0.003	0.048	0	-0.004	0.058	0
**3**	0.005	0.053	0.002	-0.004	0.062	0.001
**4**	0.012	0.053	-0.003	-0.001	0.063	0
**5**	0.012	0.048	-0.008	0.001	0.056	-0.002
**6**	0.009	0.057	0.002	-0.005	0.066	0.001
**7**	0.007	0.058	0.004	-0.004	0.068	0.003
**8**	0.002	0.052	0.003	-0.005	0.061	0.003
**9**	0.013	0.055	-0.005	0.002	0.059	-0.001
**10**	0.009	0.055	0	-0.004	0.064	0.001
**11**	0.009	0.061	0.001	-0.005	0.055	0.002
**12**	0.011	0.06	-0.001	-0.006	0.069	0.001
**13**	0.003	0.047	-0.001	-0.006	0.053	0.001
**14**	0.006	0.052	-0.002	-0.003	0.059	0
**15**	0.006	0.053	0.001	-0.005	0.059	0.002
**16**	0.012	0.047	-0.007	-0.001	0.053	0.002

Compound 17:

(A)

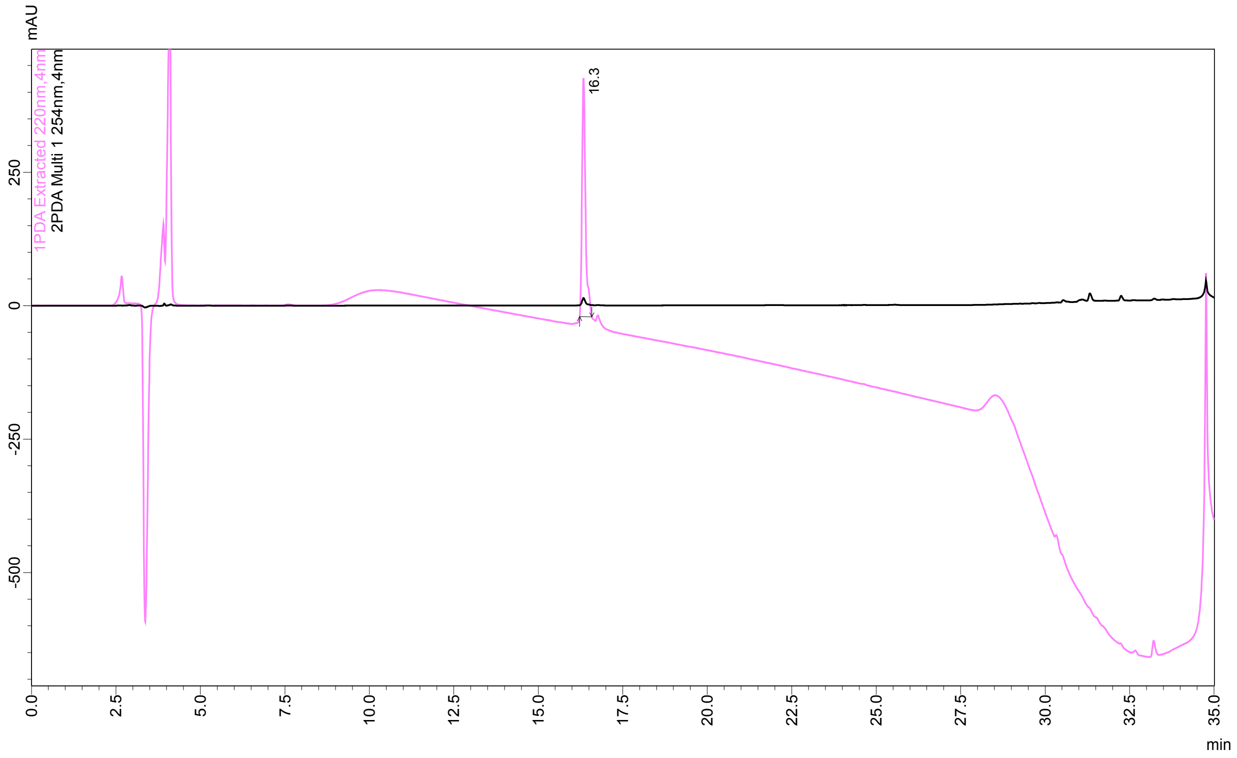

(B)

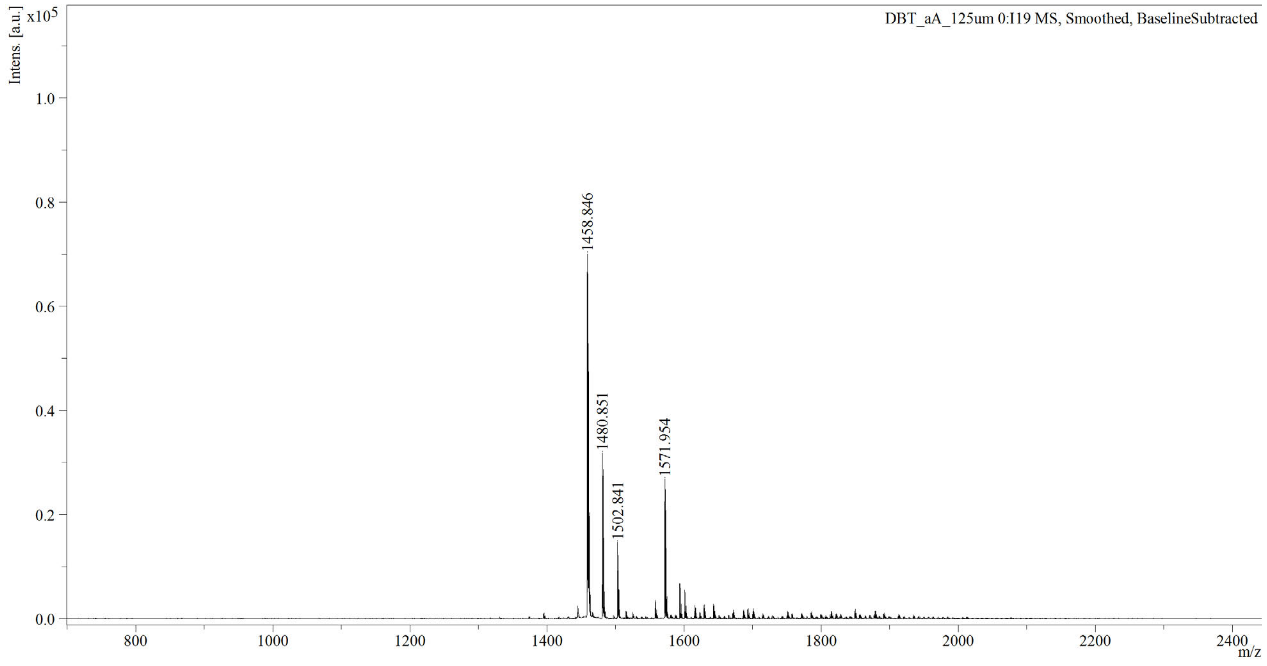

Figure S17.1: A) Analytical HPLC chromatogram of purified compound **17** at 10-50% ACN/H_2O gradient and (B) the respective MALDI profile of the pure compound. Calculated MW: 1458.8718 [M+H]; Observed MW: 1458.846.

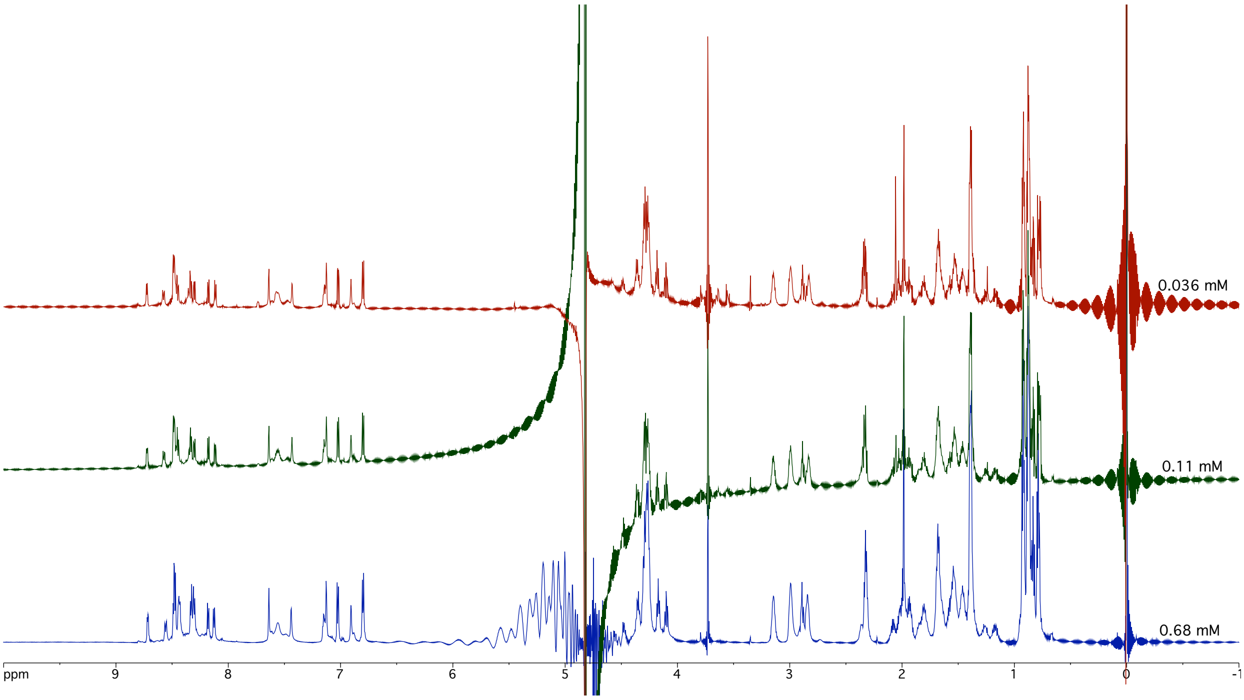

Figure S17.2: ^{1}H NMR spectra of Compound **17** at three different dilutions in 50 mM sodium acetate buffer (pH=3.8) in 9:1 H_2O/D_2O at 25°C.

Compound 18:

(A)

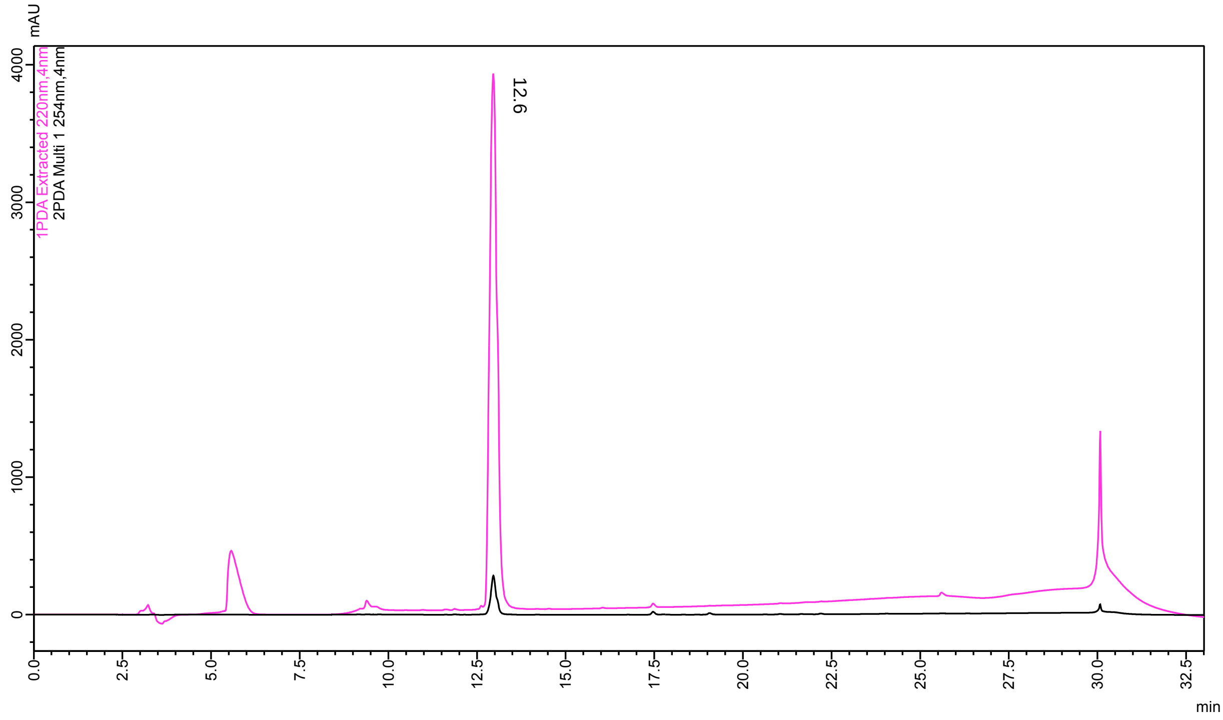

(B)

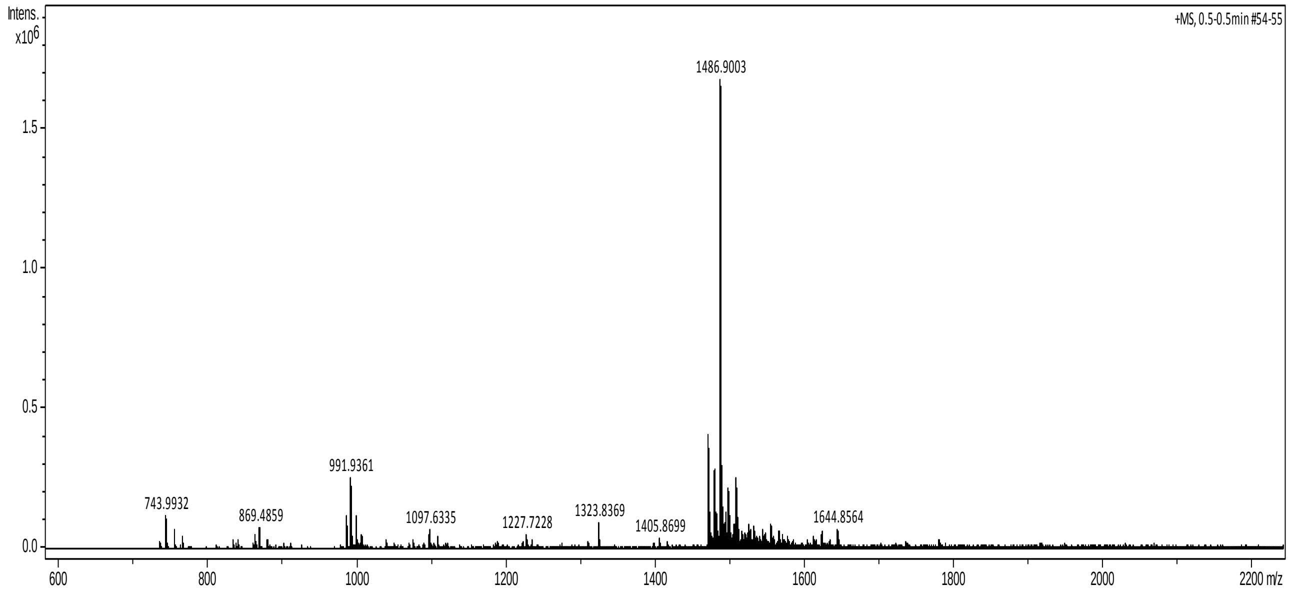

Figure S18.1: A) Analytical HPLC chromatogram of purified compound **18** at 10-50% ACN/H_2O gradient and (B) the respective HRMS profile of the pure compound. Calculated MW: 1486.9031 [M+H]; Observed MW: 1486.9003.

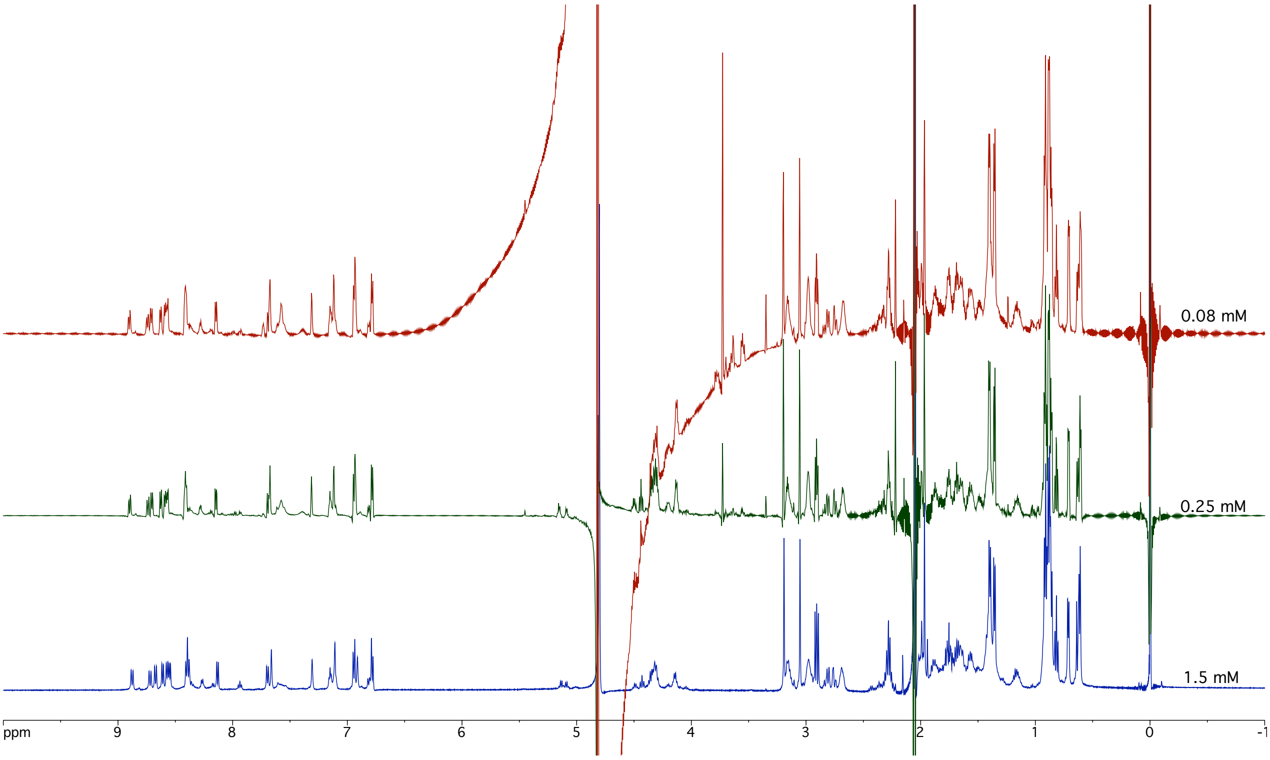

Figure S18.2: ^{1}H NMR spectra of Compound **18** at three different dilutions in 50 mM sodium acetate buffer (pH=3.8) in 9:1 H_2O/D_2O at 25°C.

Compound 18a:

Sequence: Ac-R-Y-V-E-V-A’-A’-K-K-I-L-Q-CONH$_2$

(A)

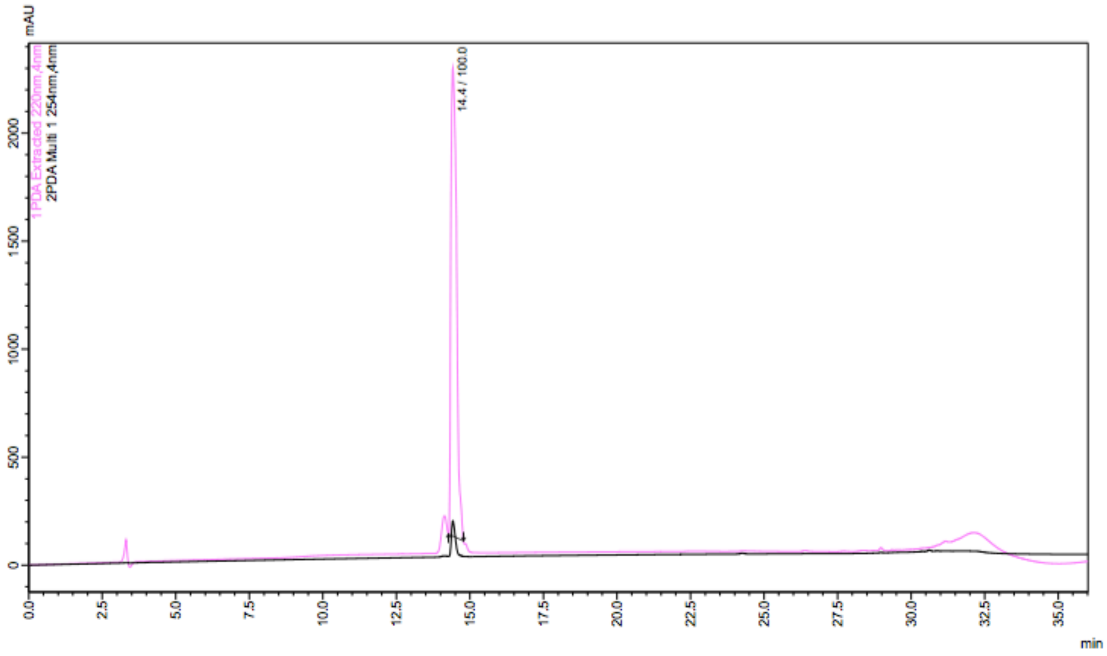

(B)

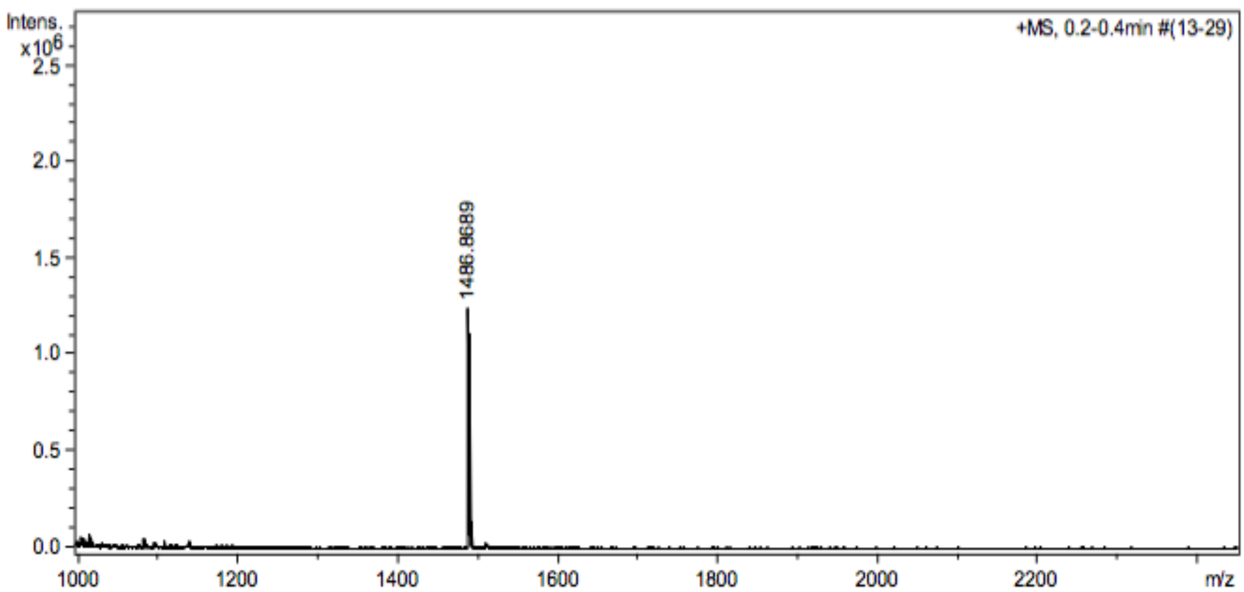

Figure S18a.1: A) Analytical HPLC chromatogram of purified compound **18a** at 10-50% ACN/H_2O gradient and (B) the respective ESI-MS profile of the pure compound. Calculated MW: 1486.9031 [M+H]; Observed MW: 1486.8689.

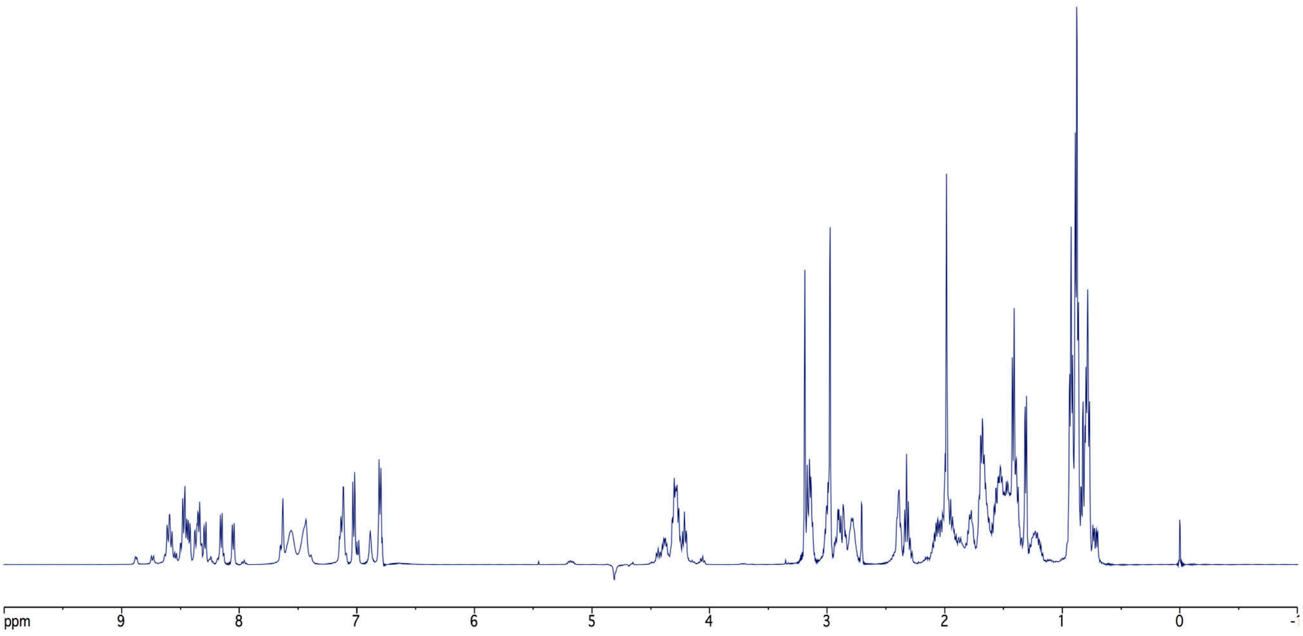

Figure S18a.2: ^{1}H NMR spectra of Compound **18a** in 50 mM sodium acetate buffer (pH=3.8) in 9:1 H_2O/D_2O at 25°C

Compound 19:

(A)

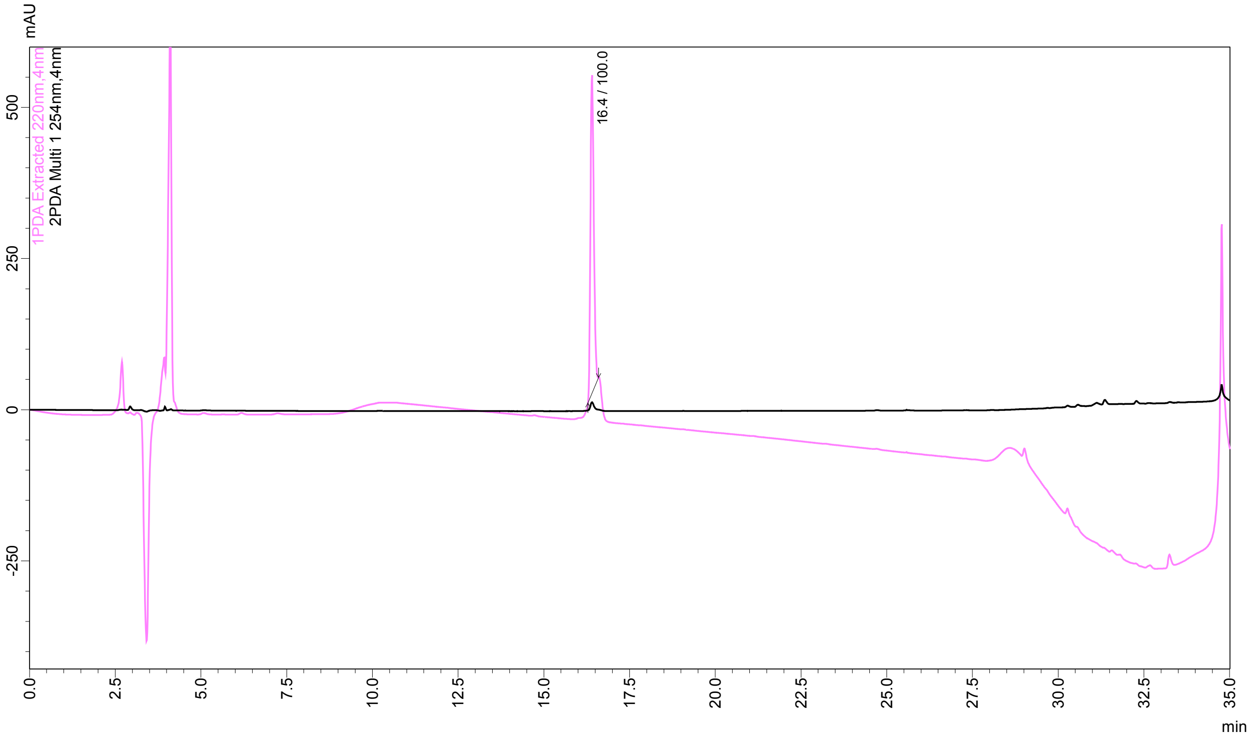

(B)

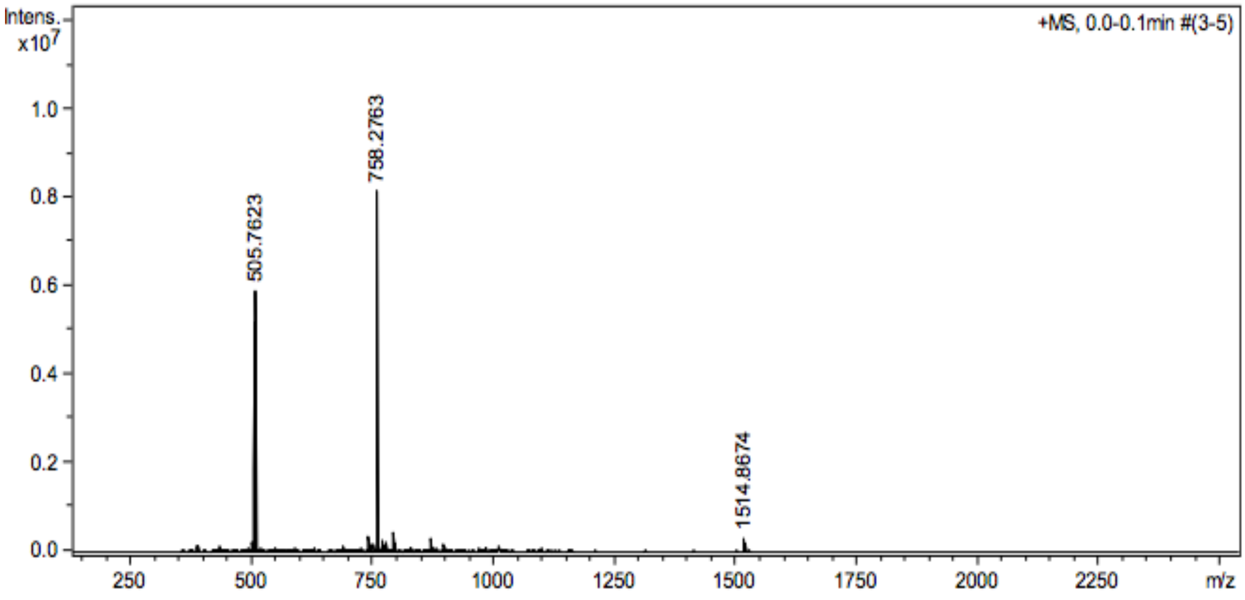

Figure S19.1: A) Analytical HPLC chromatogram of purified compound **19** at 10-50% ACN/H_2O gradient and (B) the respective ESI-MS profile of the pure compound. Calculated MW: 1514.8810 [M+H]; Observed MW: 1514.8674.

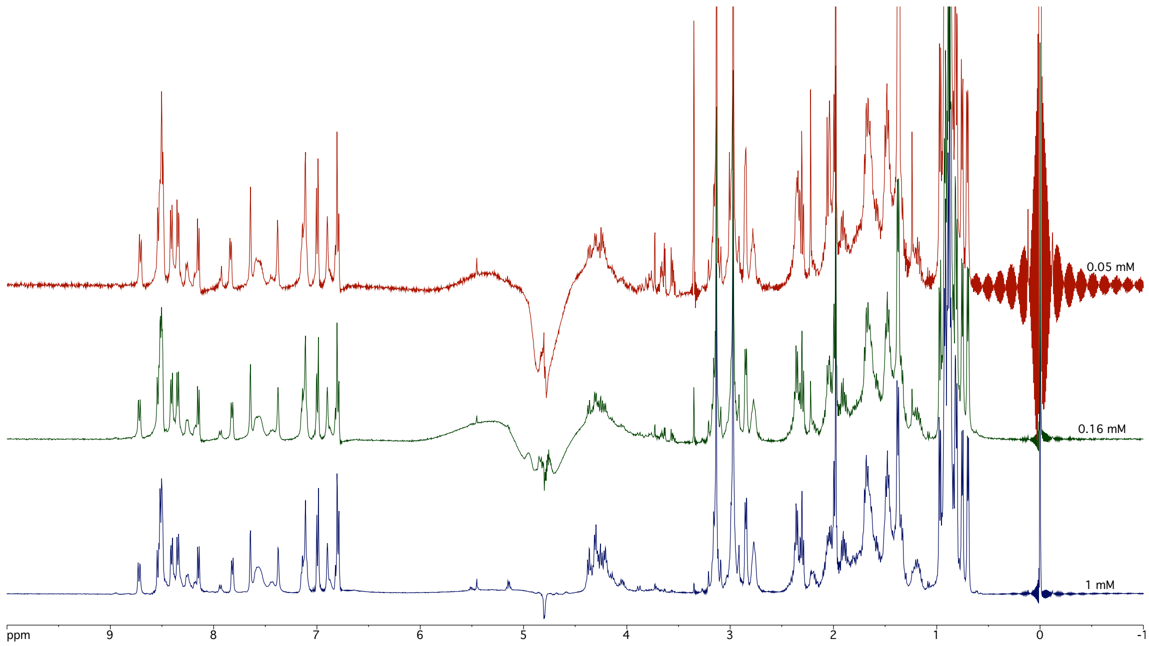

Figure S19.2: ^{1}H NMR spectra of Compound **19** at three different dilutions in 50 mM sodium acetate buffer (pH=3.8) in 9:1 H_2O/D_2O at 25°C.

Compound 19a:

Sequence: Ac-R-Y-V-E-V-A'-V'-K-K-I-L-Q-CONH$_2$

(A)

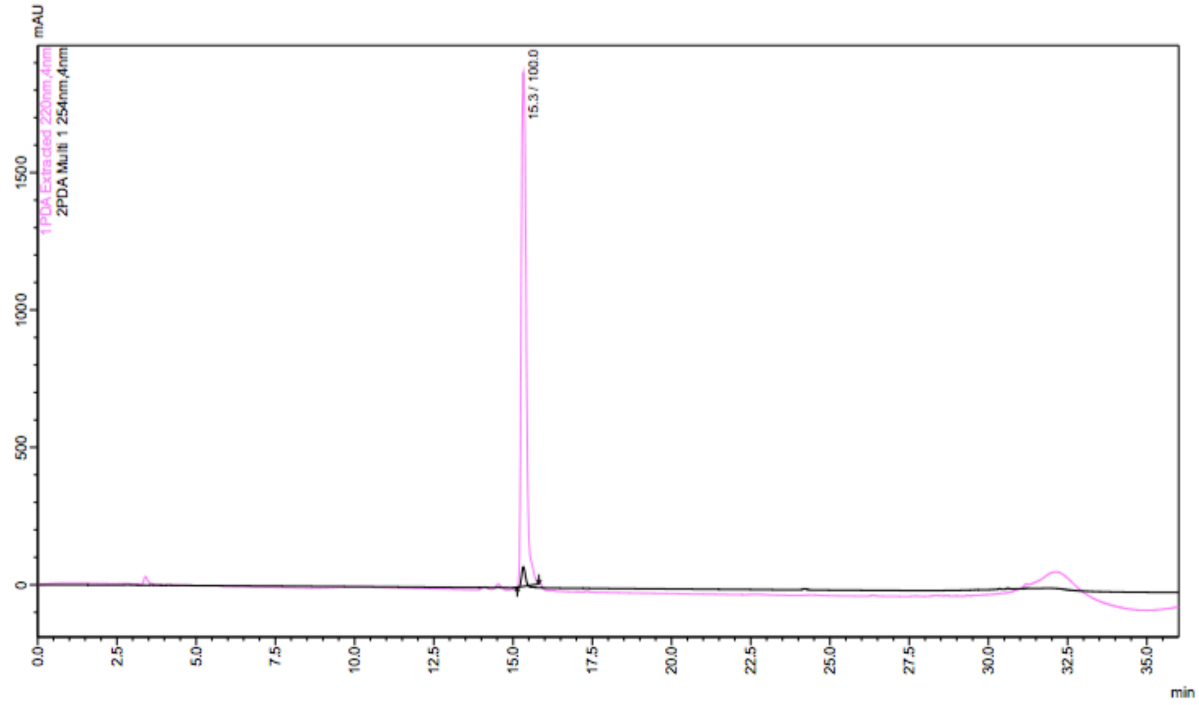

(B)

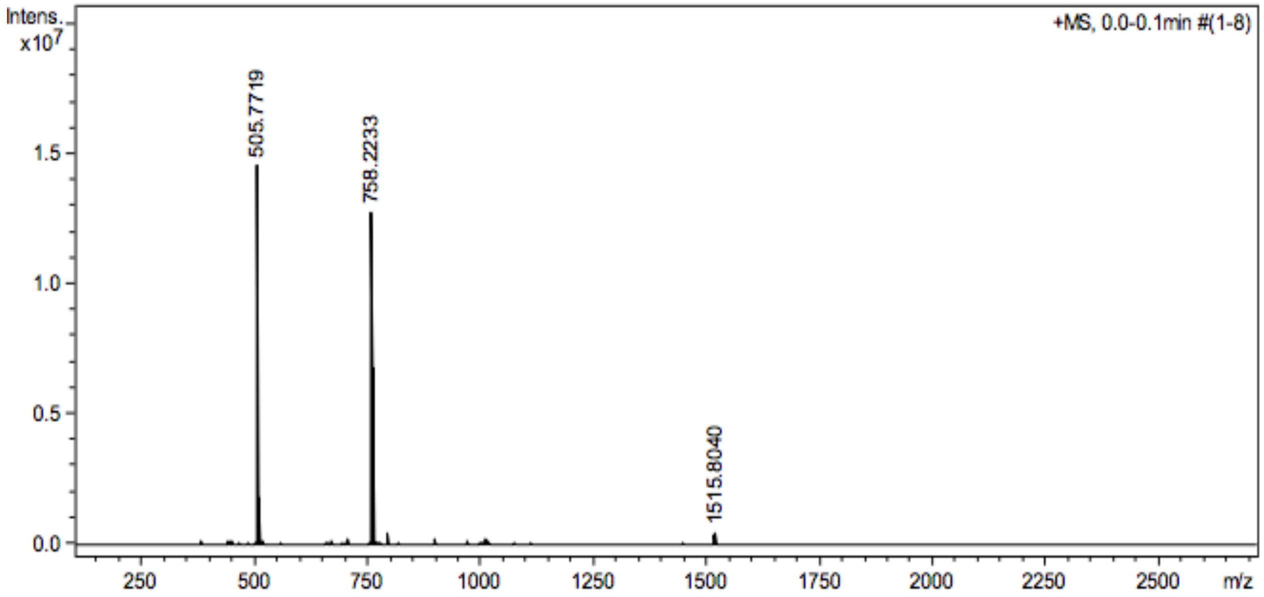

Figure S19a.1: A) Analytical HPLC chromatogram of purified compound **19a** at 10-50% ACN/H_2O gradient and (B) the respective ESI-MS profile of the pure compound. Calculated MW: 1515.8810 [M+H]; Observed MW: 1515.8040.

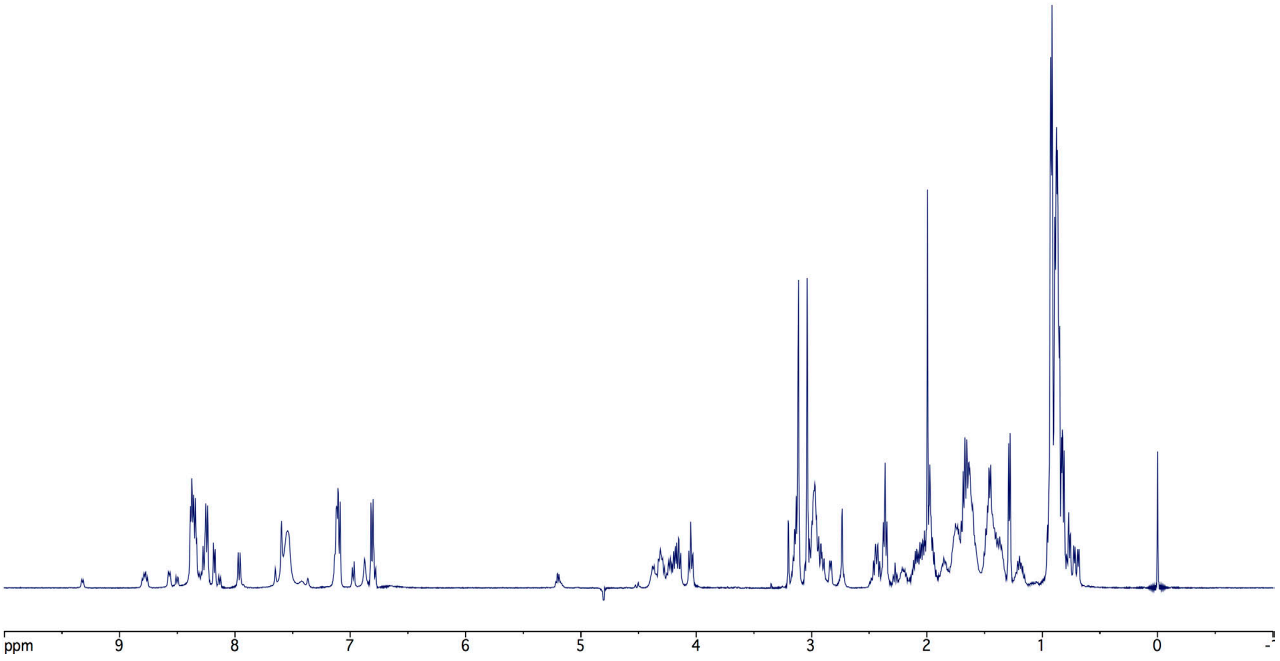

Figure S19a.2: ^{1}H NMR spectra of Compound **19a** in 50 mM sodium acetate buffer (pH=3.8) in 9:1 H_2O/D_2O at 25°C.

Compound 20:

(A)

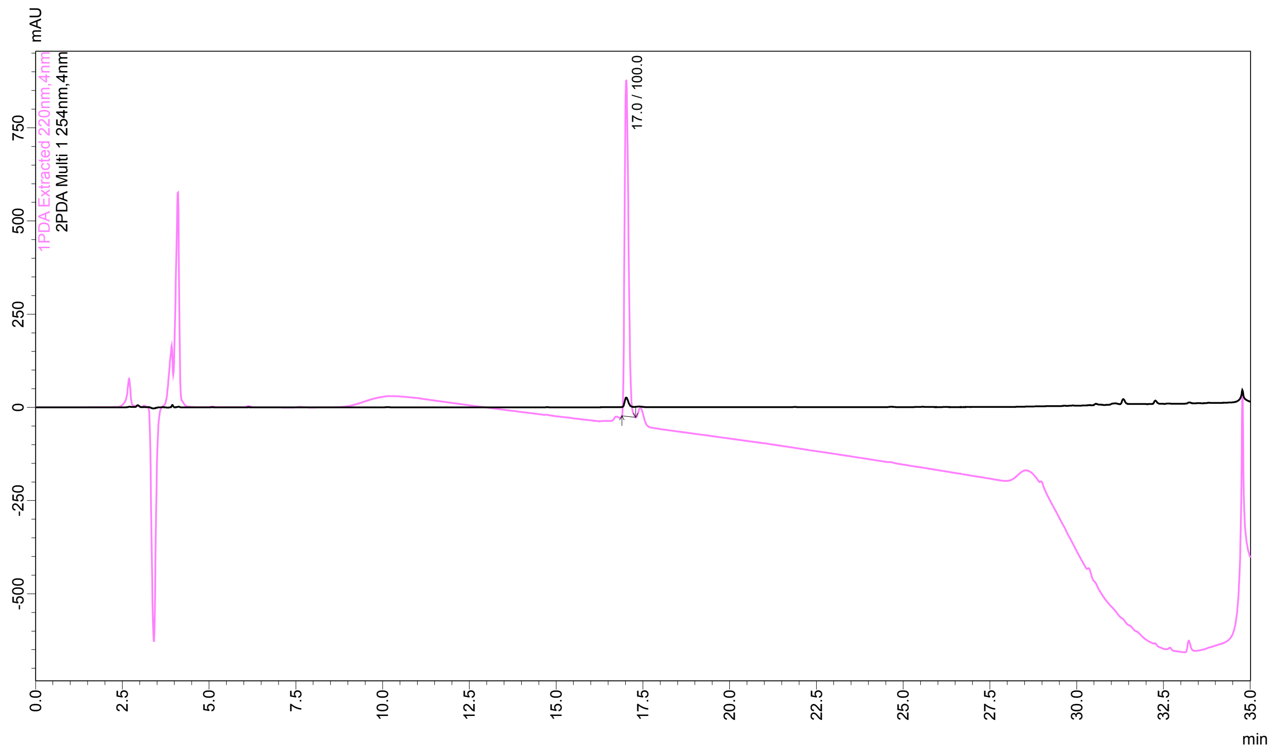

(B)

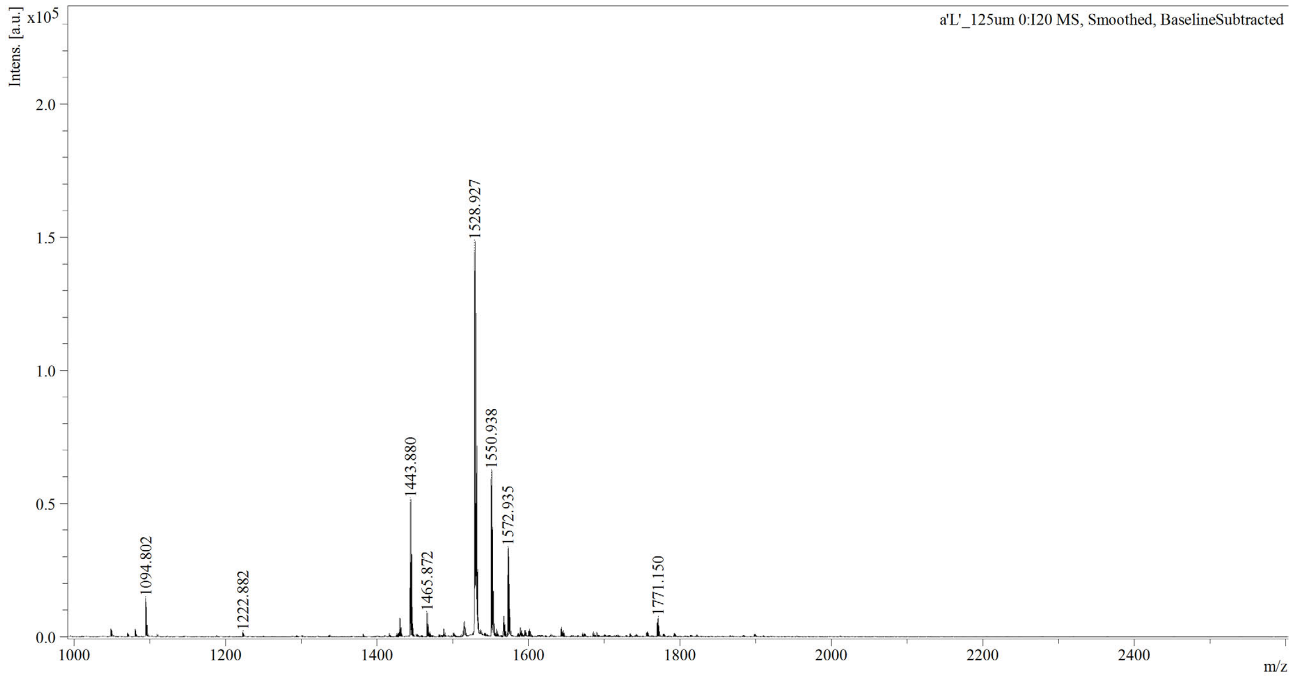

Figure S20.1: A) Analytical HPLC chromatogram of purified compound **20** at 10-50% ACN/H_2O gradient and (B) the respective MALDI profile of the pure compound. Calculated MW: 1528.9501 [M+H]; Observed MW: 1528.927.

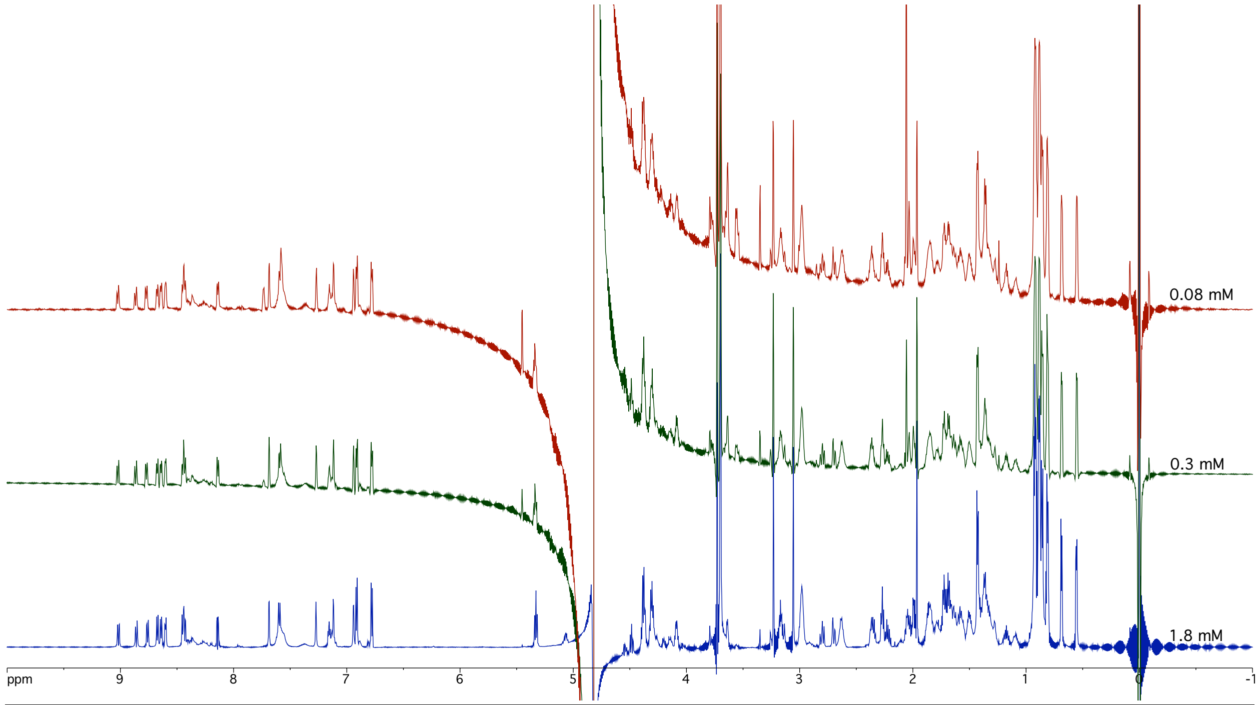

Figure S20.2: ^{1}H NMR spectra of Compound **20** at three different dilutions in 50 mM sodium acetate buffer (pH=3.8) in 9:1 H_2O/D_2O at 25°C.

Compound 20a:

Sequence: Ac-R-Y-V-E-V-A'-L'-K-K-I-L-Q-CONH$_2$

(A)

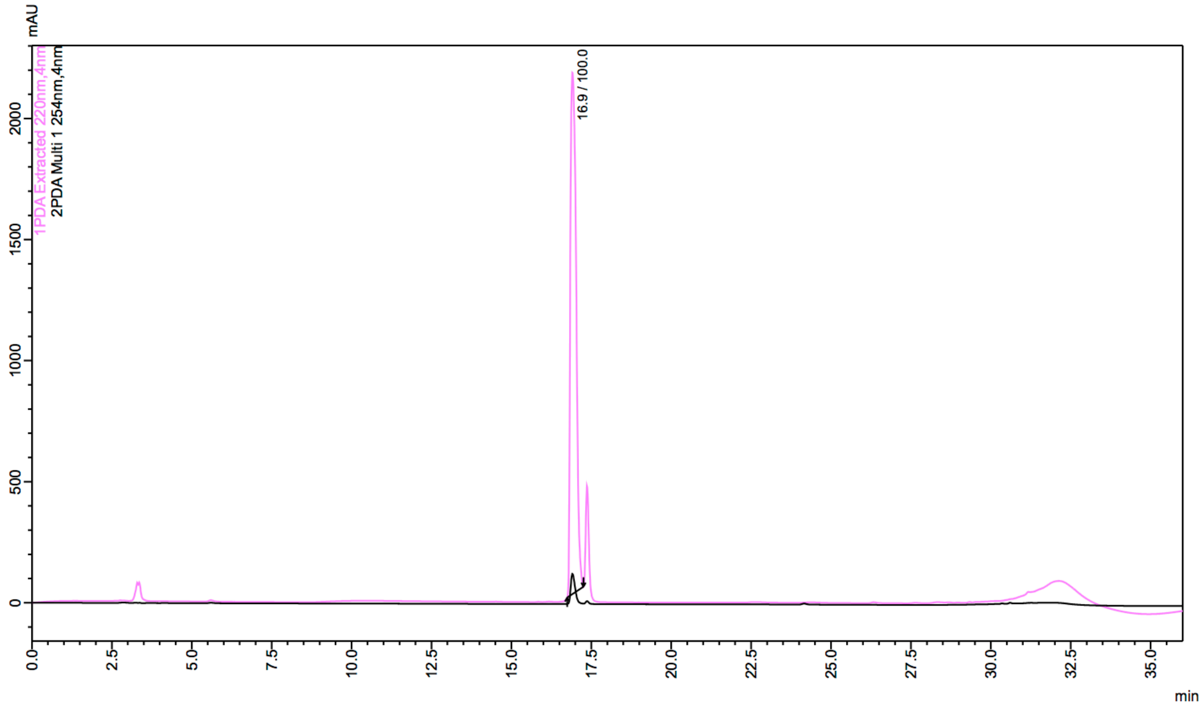

(B)

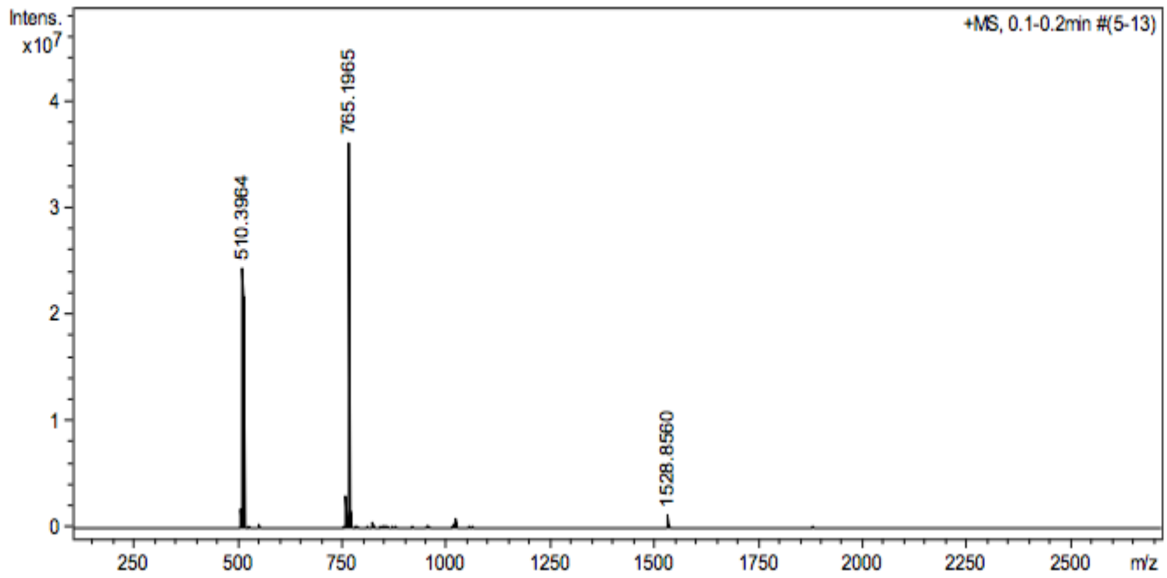

Figure S20a.1: A) Analytical HPLC chromatogram of purified compound **20a** at 10-50% ACN/H_2O gradient and (B) the respective ESI-MS profile of the pure compound. Calculated MW: 1528.9501 [M+H]; Observed MW: 1528.8560.

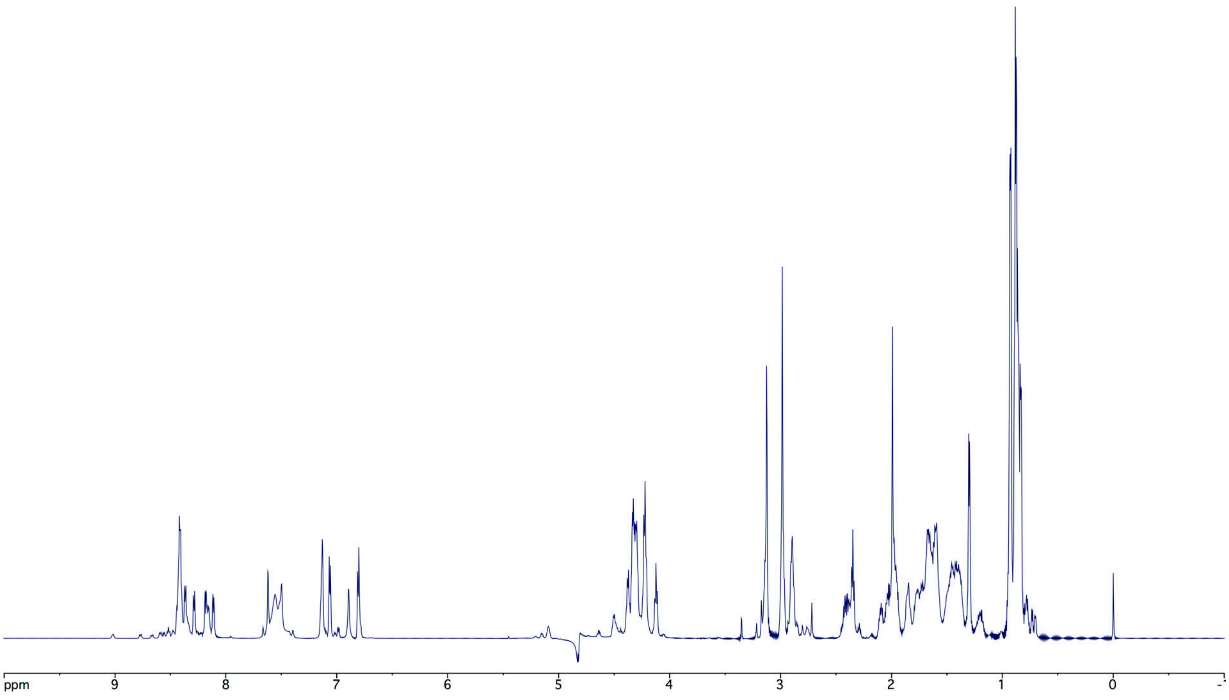

Figure S20a.2: ^{1}H NMR spectra of Compound **20a** in 50 mM sodium acetate buffer (pH=3.8) in 9:1 H_2O/D_2O at 25°C.

Compound 21:

(A)

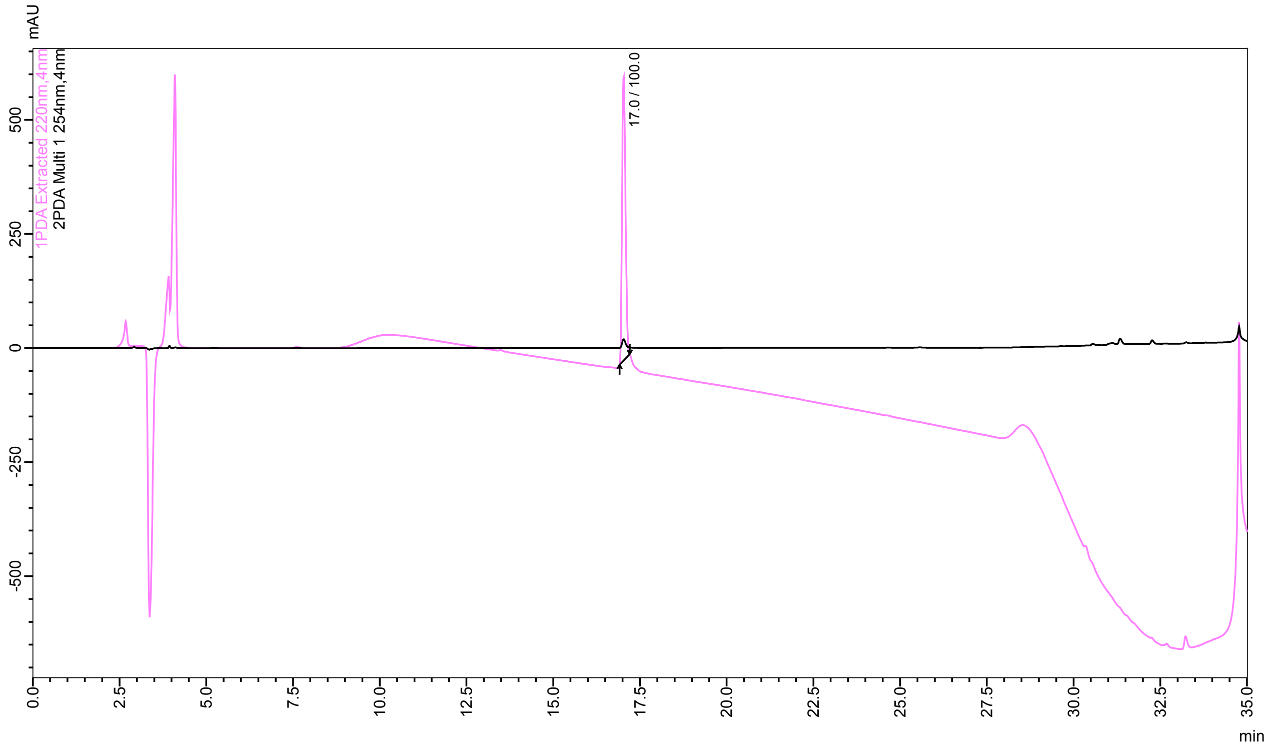

(B)

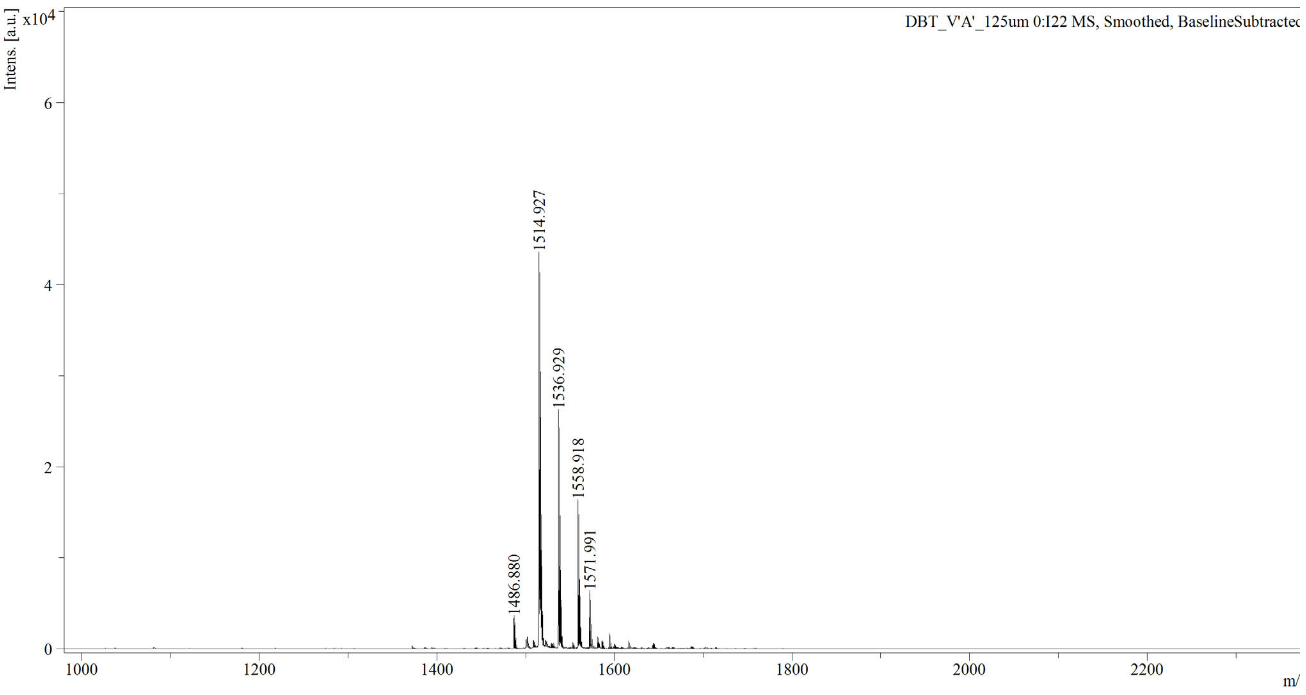

Figure S21.1: A) Analytical HPLC chromatogram of purified compound **21** at 10-50% ACN/H_2O gradient and (B) the respective MALDI profile of the pure compound. Calculated MW: 1514.9344 [M+H]; Observed MW: 1514.927.

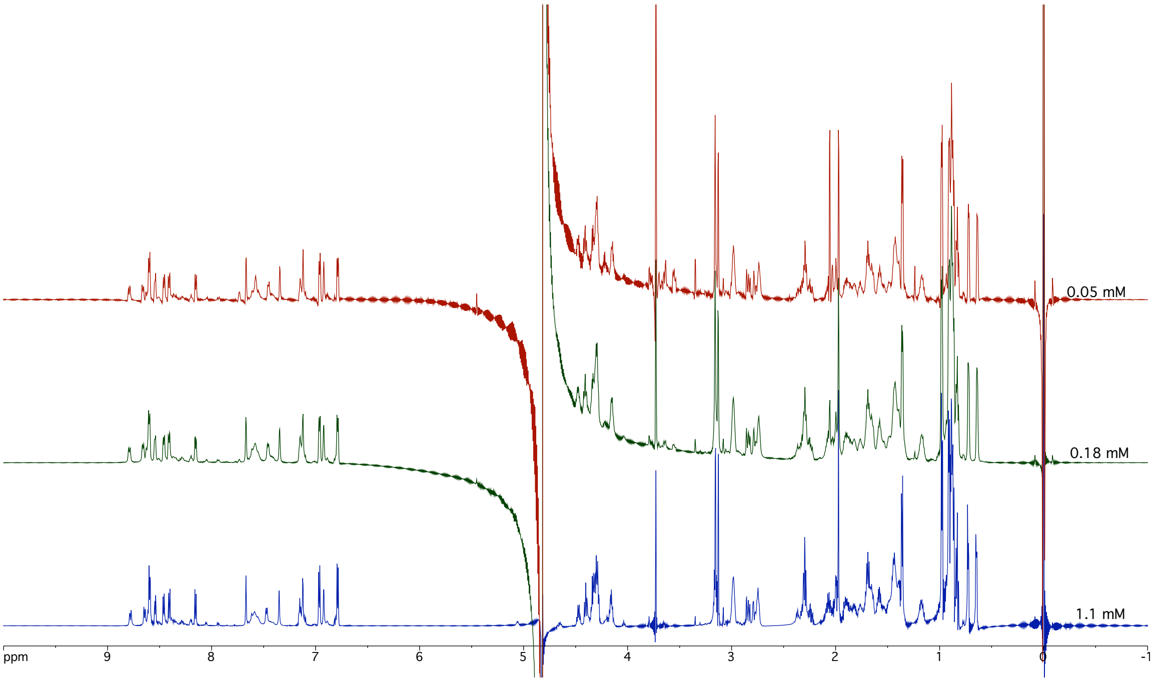

Figure S21.2: ^{1}H NMR spectra of Compound **21** at three different dilutions in 50 mM sodium acetate buffer (pH=3.8) in 9:1 H_2O/D_2O at 25°C.

Compound 21a:

Sequence: Ac-R-Y-V-E-V-V'-A'-K-K-I-L-Q-CONH$_2$

(A)

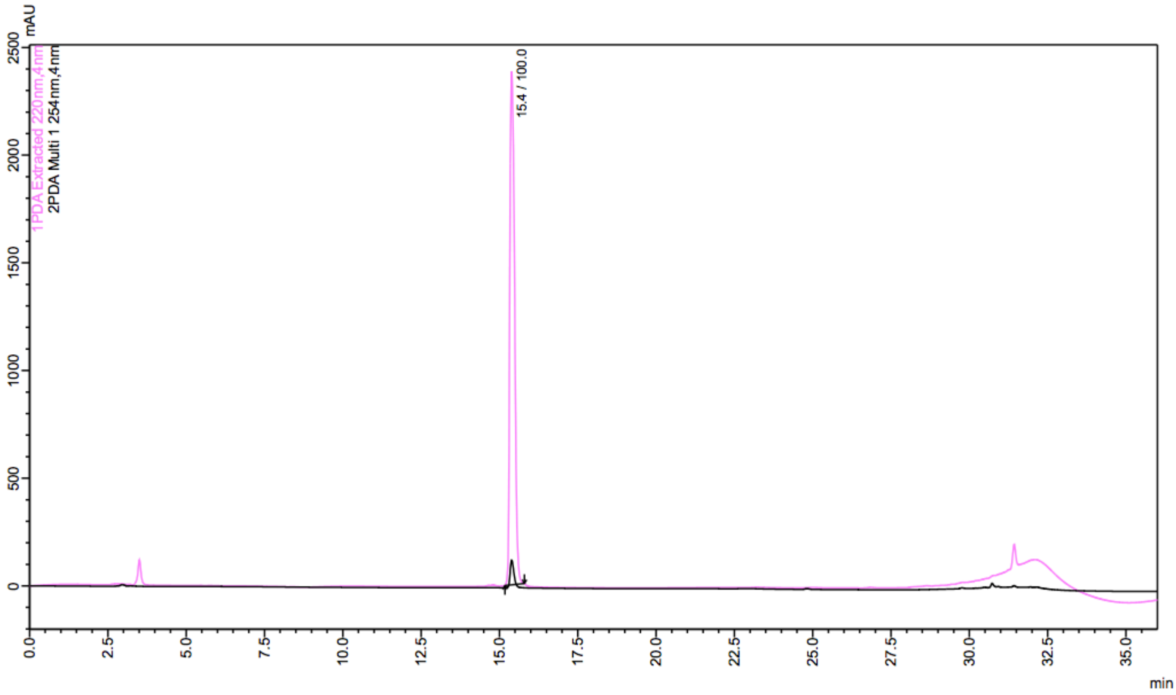

(B)

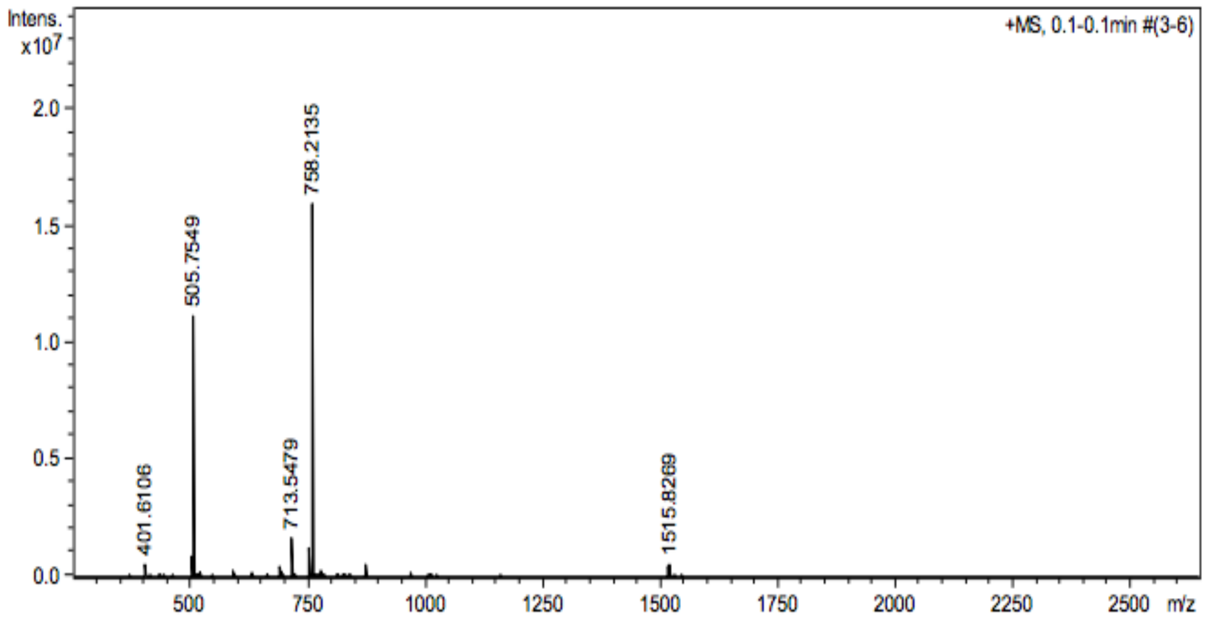

Figure S21a.1: A) Analytical HPLC chromatogram of purified compound **21a** at 10-50% ACN/H_2O gradient and (B) the respective MALDI profile of the pure compound. Calculated MW: 1515.9344 [M+H]; Observed MW: 1515.8269.

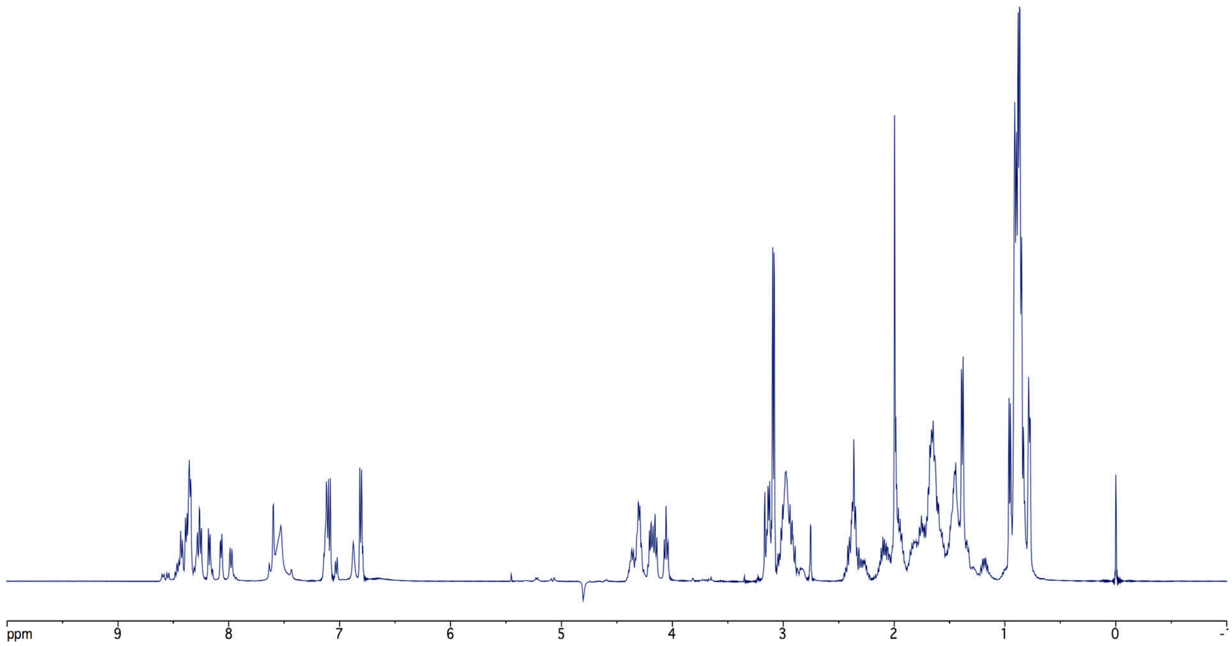

Figure S21a.2: ^{1}H NMR spectra of Compound **21a** in 50 mM sodium acetate buffer (pH=3.8) in 9:1 H_2O/D_2O at 25°C.

Table S3: Chemical shifts table of compounds **17** to **21.**

Compounds							
**Residues**			**17**	**18**	**19**	**20**	**21**
**Arg1**	**HN**		8.18	8.15	8.17	8.14	8.15
	**HA**		4.27	4.35	4.29	4.38	4.34
	**HB**	1	1.66			1.73	1.72
		2				1.66	1.65
	**HG**	1	1.5			1.57	1.55
		2				1.52	1.5
	**HD**		3.15	3.16	3.16	3.18	3.17
	**NAc**		1.99	1.98	1.98	1.96	1.97
**Tyr2**	**HN**		8.33	8.39	8.36	8.44	8.41
	**HA**		5.06	5.01	4.88	5.08	4.95
	**HB**	1	2.89	2.82	2.86	2.8	2.84
		2		2.75		2.71	2.8
**Val3**	**HN**		8.33	8.72	8.44	8.86	8.61
	**HA**		4.17	4.32	4.23	4.37	4.3
	**HB**		1.99	1.99	1.98	2	1.98
	**HG**	1	0.88	0.87	0.89	0.88	0.89
		2					
**Glu4**	**HN**		8.48	8.58	8.53	8.64	8.6
	**HA**		4.56	4.78	4.64	4.9	4.66
	**HB**	1	2.04		2.05	2.06	2.01
		2	1.95		1.92	1.88	1.92
	**HG**	1	2.33		2.32	2.36	2.31
		2				2.22	2.25
**Val5**	**HN**		8.45	8.67	8.49	8.76	8.65
	**HA**		4.11	4.71	4.68	4.76	4.68
	**HB**		2.01	2.01	2.03	2.04	2.08
	**HG**	1	0.93	0.92	0.93	0.93	0.92
		2					
**Xaa6**	**NMe/HN**		8.72	3.19	3.12	3.23	3.16
	**HA**		4.26	5.09	5.17	5.07	4.81
	**HB**		1.4	1.4	1.38	1.43	2.28
	**HG**	1					
		2					
	**HD**	1					
		2					
**Xaa7**	**NMe/HN**		8.44	3.05	2.97	3.06	3.13
	**HA**		4.09	5.14		5.33	5.06
	**HB**	1	1.39	1.37	2.2	1.72	1.37
		2				1.43	
	**HG**	1			0.96		
		2			0.81		

	**HD**	1				0.94	
		2				0.82	
**Lys8**	**HN**		8.13	7.7	7.9	7.6	7.47
	**HA**		4.35	4.49	4.34	4.55	4.48
	**HB**						
	**HG**						
	**HD**						
	**HE**			2.97		2.99	3
**Lys9**	**HN**		8.31	8.4	8.44	8.45	8.47
	**HA**		4.49	4.7	4.56	4.75	4.66
	**HB**						
	**HG**						
	**HD**						
	**HE**				2.78		2.74
**Ile10**	**HN**		8.56	8.88	8.69	9.01	8.79
	**HA**		4.28	4.43	4.34	4.5	4.41
	**HB**		1.87	1.87	1.87	1.9	1.87
	**HG**	1	1.45	1.18	1.49	1.42	1.43
		2	1.18		1.19	1.19	1.19
	**HD**		0.87	0.89	0.88	0.89	0.9
**Leu11**	**HN**		8.48	8.55	8.5	8.6	8.54
	**HA**		4.29	4.15	4.23	4.08	4.16
	**HB**	1	1.57	1.59	1.58	1.58	1.58
		2		1.41	1.49	1.39	1.44
	**HG**						
	**HD**	1	0.79	0.71	0.77	0.69	0.74
		2		0.61	0.73	0.56	0.65
**Gln12**	**HN**			8.62		8.68	8.6
	**HA**			4.3		4.31	4.3
	**HB**	1				1.87	2.07
		2				2.04	1.9
	**HG**	1				2.28	2.37
		2					2.3

*Note : The chemical shifts of certain residues in compounds **17** to **21** couldn't be assigned due to resonance overlap.

<u>Table S3a:</u> HN and $C^{\alpha}H$ shifts table of control compounds **18a** to **21a.**

Compounds					
Residues		18a	19a	20a	21a
Arg1	HN	8.16	8.21	8.21	8.17
	HA	4.27	4.2	4.19	4.22
	NAc	1.98	1.99	1.99	1.97
Tyr2	HN	8.25	8.26	8.28	8.26
	HA	4.65	4.65	4.74	4.65
Val3	HN	7.97	7.97	8.15	7.97
	HA	4.08	4.07	4.12	4.07
Glu4	HN	8.48	8.37	8.4	8.34
	HA	4.3	4.3	4.33	4.32
Val5	HN	8.43	8.25	8.44	8.42
	HA	4.68	4.62	4.65	4.61
Xaa6	NMe/HN	3.19	3.1	3.13	3.09
	HA	4.68	5.19	5.1	5.2
Xaa7	NMe/HN	2.98	3.04	2.99	3.08
	HA	4.46	4.51	5.11	4.69
Lys8	HN	8.05	8.37	8.12	8.05
	HA	4.38	4.32	4.37	4.33
Lys9	HN	8.34	8.33	8.41	8.36
	HA	4.4	4.24	4.33	4.33
Ile10	HN	8.58	8.41	8.4	8.38
	HA	4.31	4.15	4.22	4.15
Leu11	HN	8.46	8.36	8.35	8.36
	HA	4.33	4.37	4.32	4.36
Gln12	HN	8.42	8.36		
	HA	4.22	4.22		

*Note : The chemical shifts of certain residues in compounds **18a** to **21a** couldn't be assigned due to resonance overlap.

<u>Table S4:</u> $^{3}J_{H^{N}\text{-}H^{\alpha}}$ table of compounds **17** to **21.**

Comp.	Arg1	Tyr2	Val3	Glu4	Val5	Lys8	Lys9	Ile10	Leu11	Gln12
18	8.1	8.9	10.1	8	9.6	8.6	8.8	9.6	6.8	8.2
20	8.2	9.7	10.6	8.3	9.7	9.6	9.6	11	8.1	8.2

*Note: Coupling Constant of compounds **17**, **19** and **21** couldn't be correctly determined from the ^{1}H NMR due to overlap of the amide chemical shifts.

Structure calculation of compound 18 :

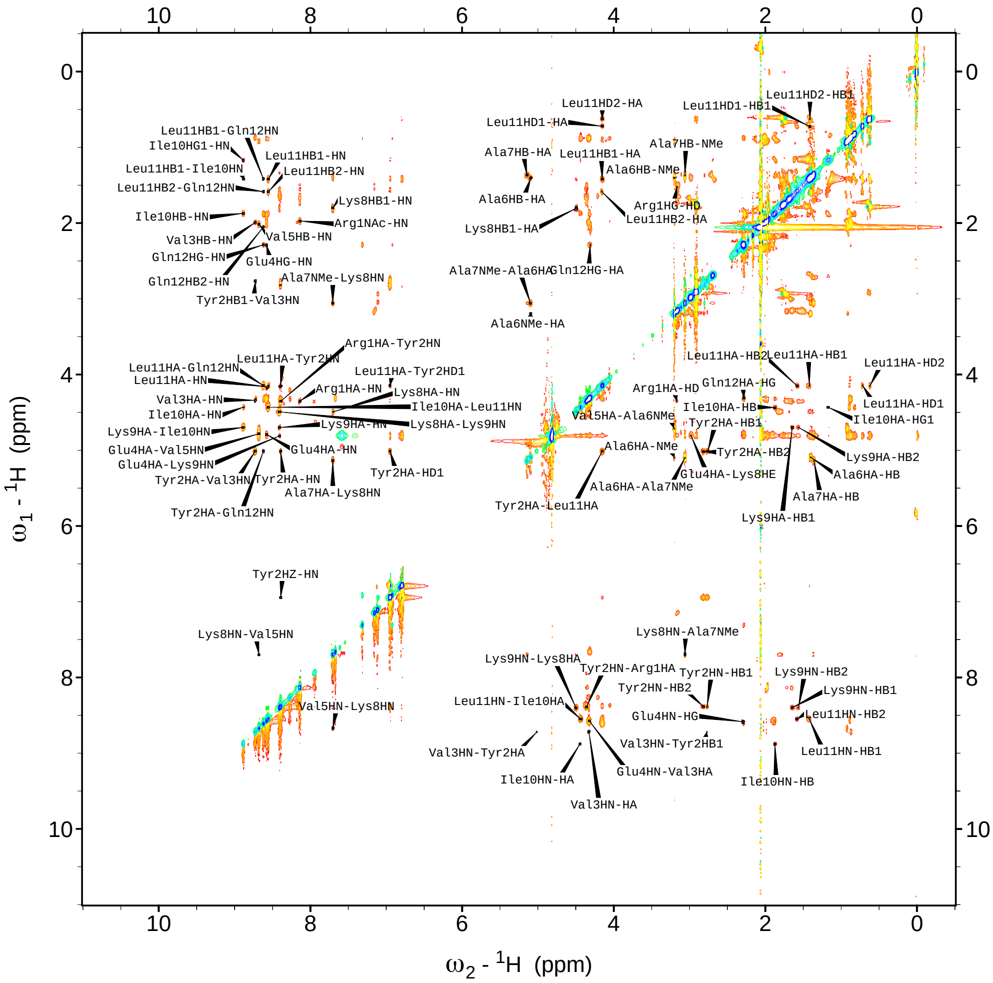

Figure S22.1: ROESY spectra with assigned peaks

Table22.2: List of ROEs with respective NMR distances and violations.

Interactions	NMR Distance	Lower Limit	Upper Limit	Observed Distance	Violations
Arg1HA-HN	2.92	2.63	3.21	2.9	0
*Arg1HA-Tyr2HN	2.22	2	2.44	2.96	0.5
Arg1NAc-HN	2.58	2.32	3.24	2.75	0
Tyr2HA-HN	2.97	2.67	3.27	2.97	0
Tyr2HA-Val3HN	2.39	2.15	2.63	2.22	0
Tyr2HA-Gln12HN	3.31	2.98	3.64	3.53	0
Tyr2HB1-Val3HN	3.76	3.38	4.14	3.88	0
Val3HA-HN	2.93	2.64	3.22	2.9	0
Val3HB-HN	3.01	2.71	3.31	3.29	0
Glu4HA-HN	2.65	2.39	2.92	2.96	0
Glu4HA-Val5HN	2.19	1.97	2.41	2.25	0
Glu4HG-HN	3.29	2.96	3.62	2.96	0
Val5HB-HN	2.74	2.47	3.01	2.85	0

**ala6HB-NMe**	2.28	2.05	3.31	3.31	0
**ala6NMe-HA**	3.89	3.5	4.68	3.69	0
**Ala7HA-Lys8HN**	2.63	2.37	2.89	3.3	0.4
**Ala7HB-NMe**	2.25	2.03	3.28	3.31	0
**Ala7NMe-ala6HA**	2.44	2.2	3.08	2.75	0
**Ala7NMe-Lys8HN**	2.71	2.44	3.38	3.08	0
**Lys8HA-HN**	2.75	2.48	3.03	2.94	0
**Lys8HA-Lys9HN**	2.22	2	2.44	2.24	0
**Lys8HB1-HA**	2.54	2.29	2.79	2.5	0
**Lys8HB1-HN**	2.48	2.23	2.73	2.75	0
**Val5HN-Lys8HN**	3.11	2.8	3.42	3.71	0.3
**Lys9HA-HN**	2.75	2.48	3.03	2.96	0
**Lys9HA-Ile10HN**	2.34	2.11	2.57	2.25	0
**Ile10HA-HN**	2.97	2.67	3.27	2.86	0
**Ile10HA-Leu11HN**	2.2	1.98	2.42	2.63	0.2
**Ile10HB-HN**	2.82	2.54	3.1	3.32	0.2
**Ile10HG1-HN**	3.13	2.82	3.44	3.32	0
**Leu11HA-HN**	2.56	2.3	2.82	2.94	0.1
**Leu11HA-Gln12HN**	2.29	2.06	2.52	2.36	0
**Leu11HB1-HA**	2.53	2.28	2.78	2.79	0
**Leu11HB1-HN**	2.54	2.29	2.79	2.72	0
***Leu11HB1-Gln12HN**	3.55	3.2	3.91	4.38	0.5
**Leu11HB2-HA**	2.74	2.47	3.01	2.98	0
**Leu11HB2-HN**	2.76	2.48	3.04	2.92	0
**Leu11HB2-Gln12HN**	3.74	3.37	4.11	3.79	0
**Leu11HD1-HA**	2.81	2.53	3.49	2.98	0
**Leu11HD2-HA**	2.73	2.46	3.4	2.98	0
**Gln12HB2-HN**	3.32	2.99	3.65	3.51	0
**Tyr2HA-Leu11HA**	2.46	2.21	2.71	2.58	0
**Tyr2HA-HD1**	2.79	2.51	3.07	2.44	-0.1
**Tyr2HD1-Leu11HA**	3.33	3	3.66	3.2	0

* violations ≥0.5. The observed high violations can be explained by the local flexibility about the γ and δ methyl groups, peak overlap, additional J-mediated transfer and inaccuracies in the force fields.[9]

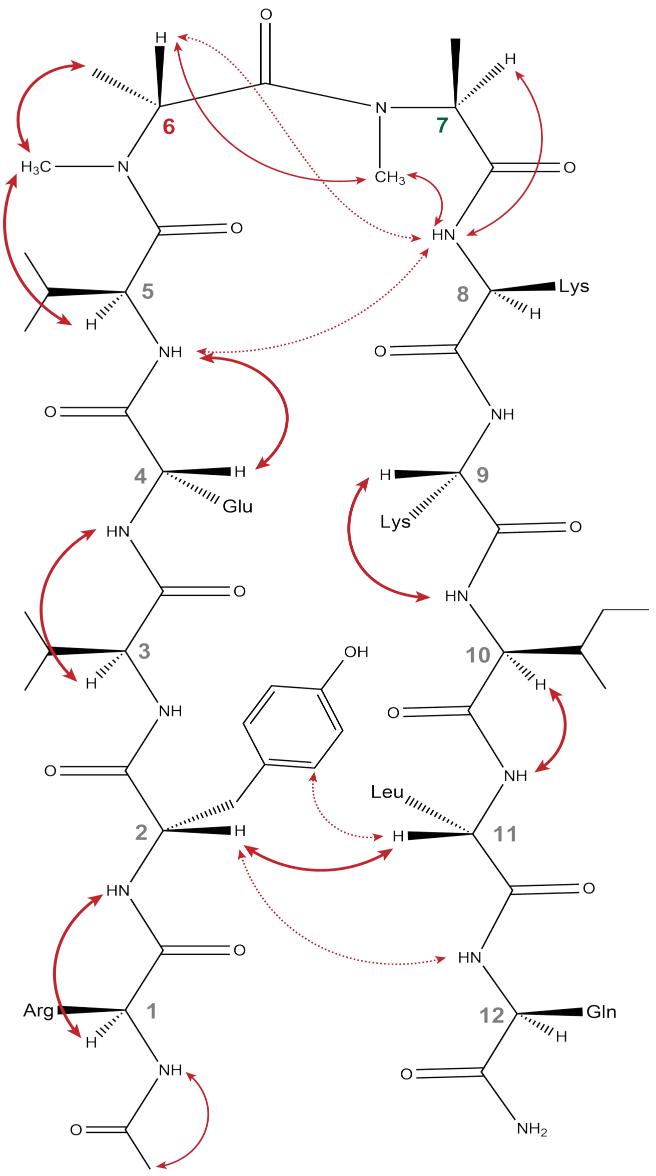

Figure S22.2: Characteristic inter-residue (short and long-range) NOEs of compound **18** are shown with thick arrows denoting distances between 1.8-2.5 Å, thin arrows denoting 2.6-3.0 Å and dotted arrows denoting 3.0 Å and above.

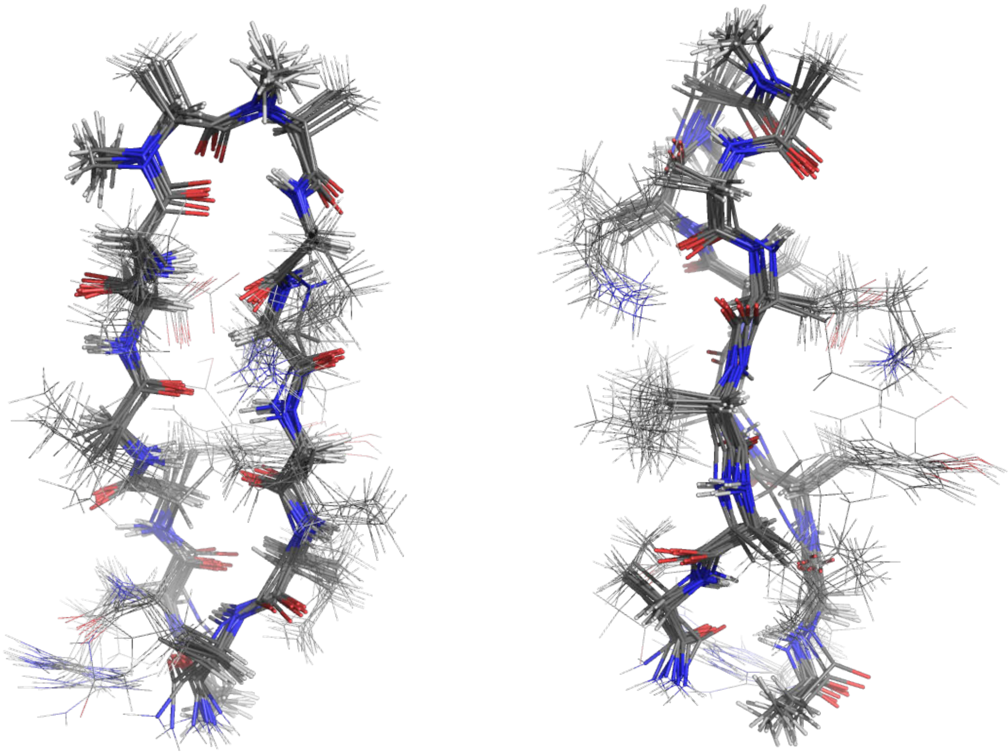

Figure S22.3: Overlay of 10 representative conformations generated using Molecular Dynamics simulation, showing both front view (left panel) and side view (right panel).

Table 22.3: Average dihedral angles for compound 18 obtained from the conformations generated by the restrained molecular dynamics simulation.

**Residues**	φ	ψ
**Arg1**		-150 ± 14
**Tyr2**	-126 ± 15	167 ± 7
**Val3**	-149 ± 9	134 ± 10
**Glu4**	-99 ± 8	143 ± 8
**Val5**	-138 ± 9	97 ± 9
**D-ala6 (i+1)**	**66 ± 8**	**-142 ± 7**
**Ala7 (i+2)**	**-76 ± 8**	**-12 ± 7**
**Lys8**	-83 ± 7	72 ± 9
**Lys9**	-68 ± 9	166 ± 9
**Ile10**	-160 ± 11	165 ± 9
**Leu11**	-108 ± 9	141 ± 7
**Gln12**	-150 ± 8	

BACKBONE OVERLAYED STRUCTURES

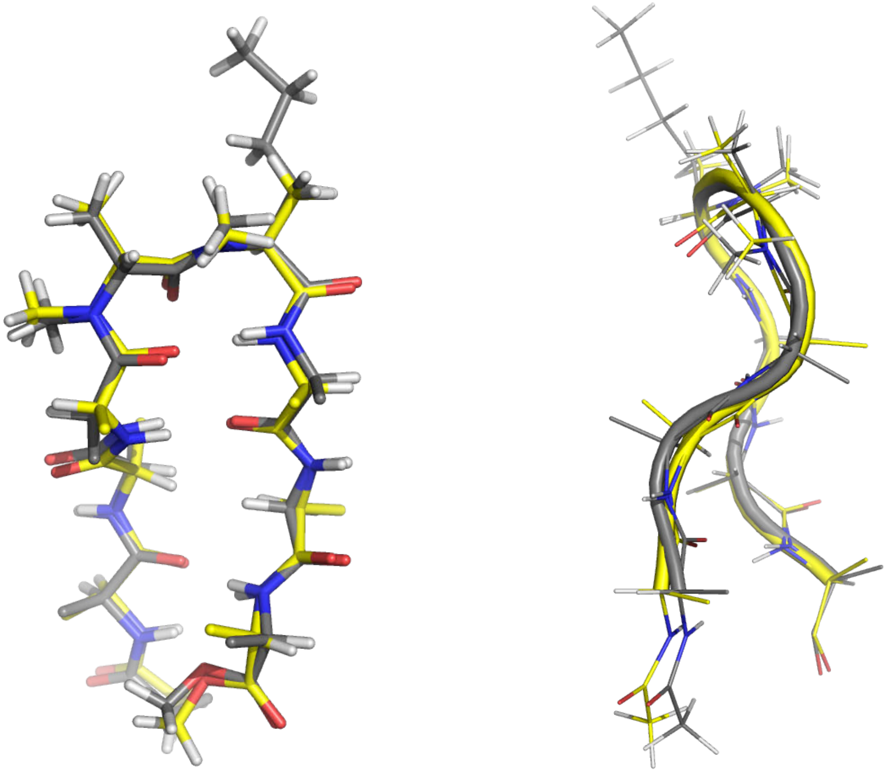

Figure S23.1: Backbone overlay of compounds **2** (yellow) and **3** (grey), showing front (left panel) and side (right panel) view and having a RMSD = 0.20 Å.

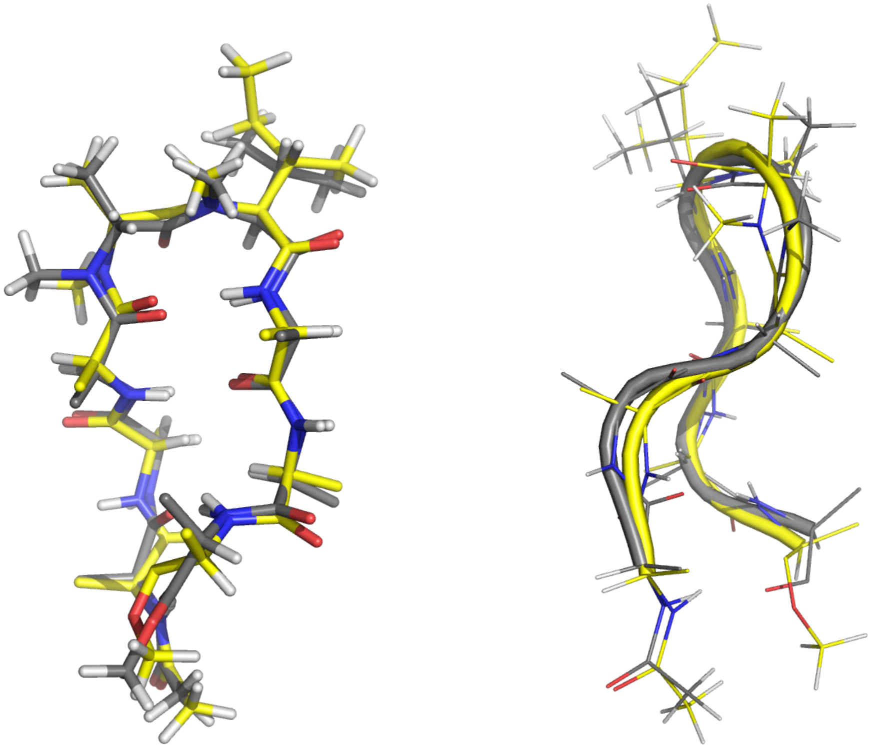

Figure S23.2: Backbone overlay of compounds **5** (yellow) and **4** (grey), showing front (left panel) and side (right panel) view and having a RMSD = 0.45 Å.

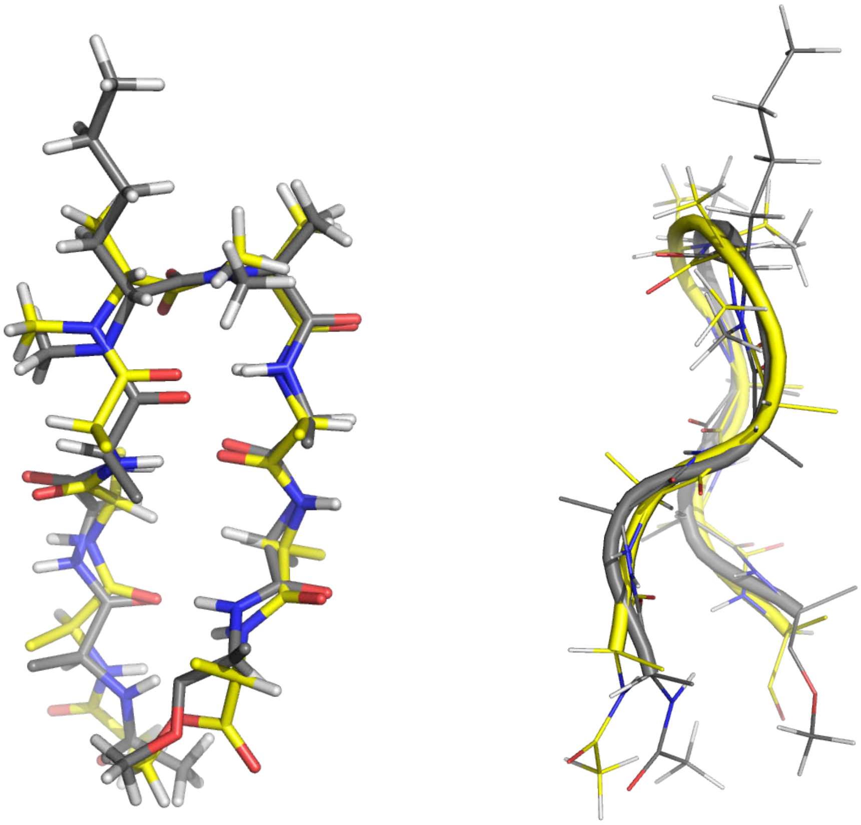

Figure S23.3: Backbone overlay of compounds **2** (yellow) and **10** (grey), showing front (left panel) and side (right panel) view and having a RMSD = 0.56 Å.

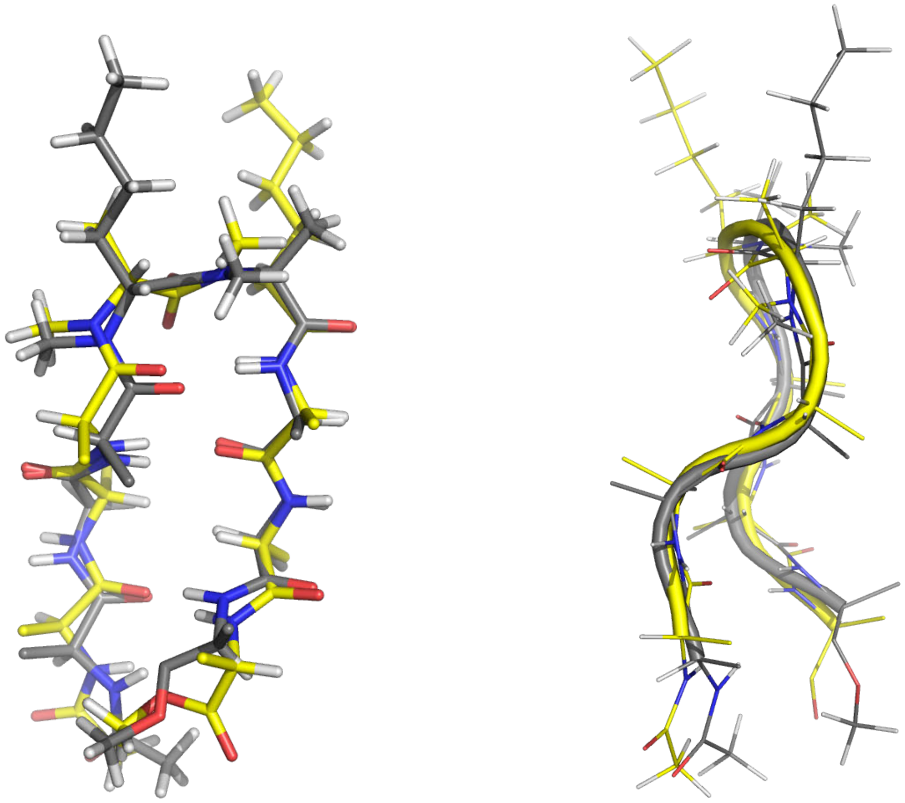

Figure S23.4: Backbone overlay of compounds **10** (yellow) and **3** (grey), showing front (left panel) and side (right panel) view and having a RMSD = 0.50 Å.

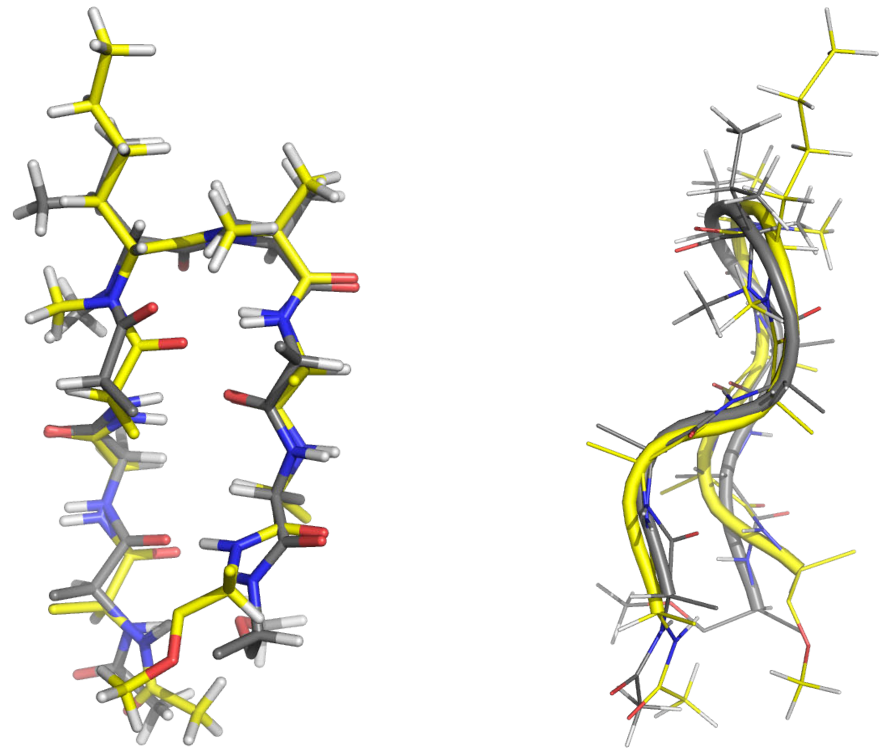

Figure S23.5: Backbone overlay of compounds **10** (yellow) and **11** (grey), showing front (left panel) and side (right panel) view and having a RMSD = 0.39 Å.

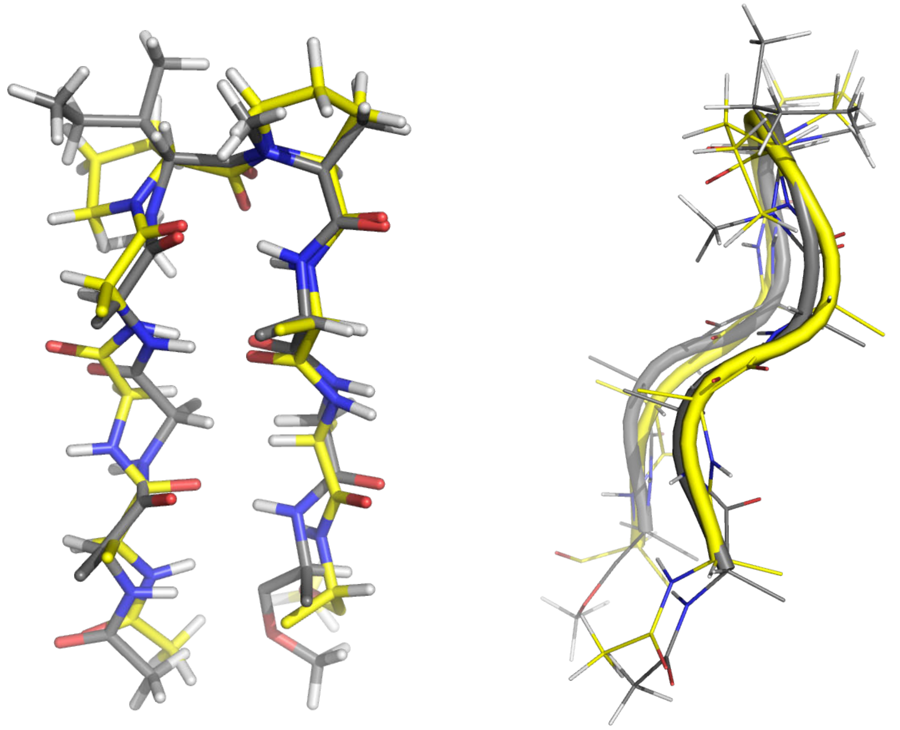

Figure S23.6: Backbone overlay of compounds **1** (yellow) and **12** (grey), showing front (left panel) and side (right panel) view and having a RMSD = 0.64 Å.

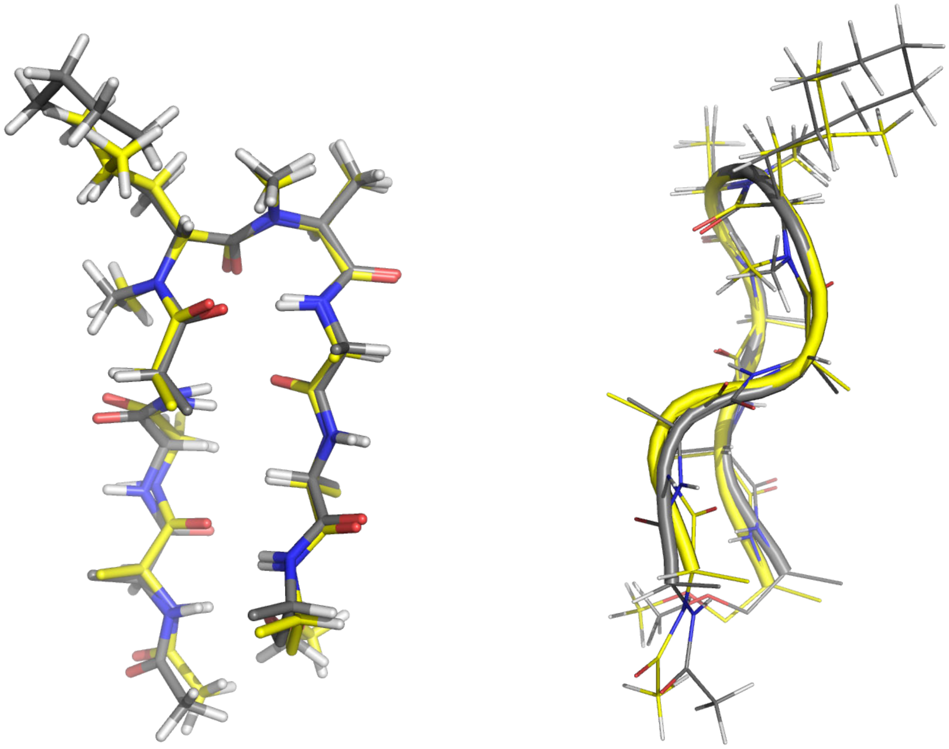

Figure S23.7: Backbone overlay of compounds **13** (yellow) and **14** (grey), showing front (left panel) and side (right panel) view and having a RMSD = 0.24 Å.

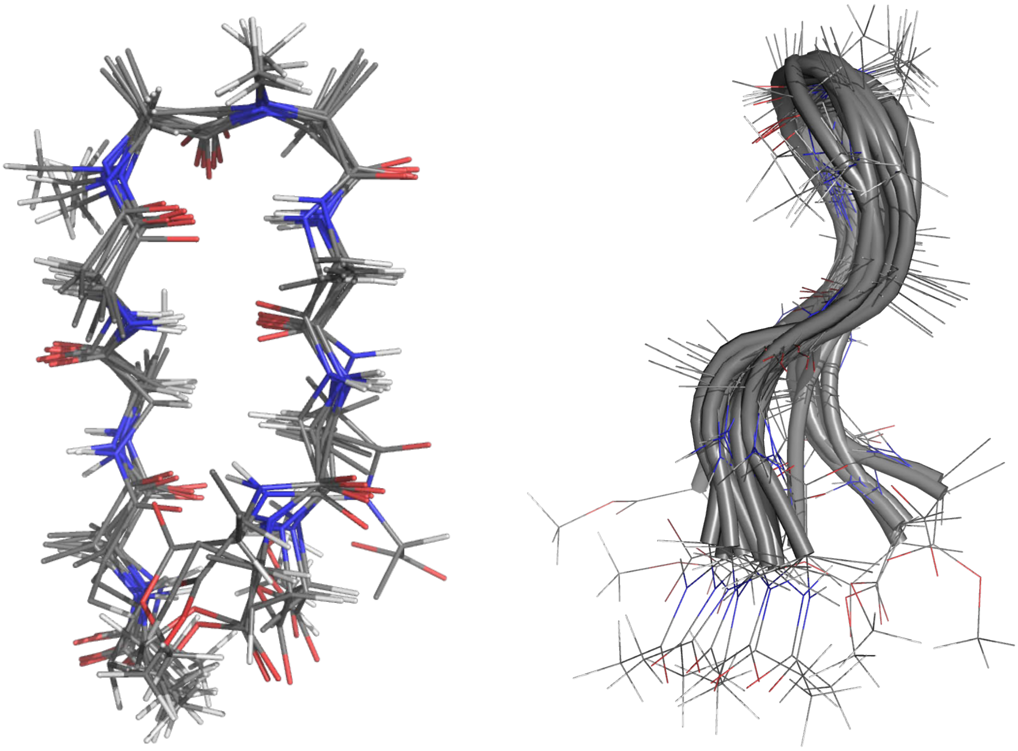

Figure S23.8: Backbone overlay of all ***i*+2** analogs (**2-9**), showing both front (left panel) and side (right panel) view.

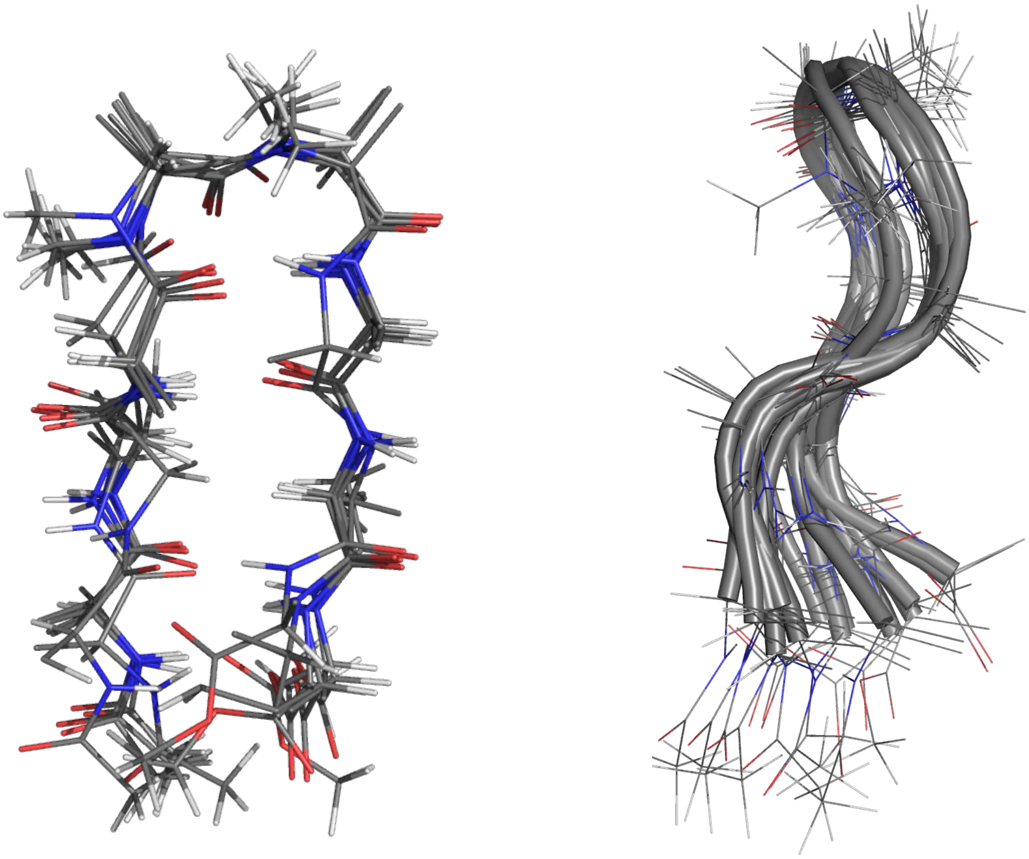

Figure S23.9: Backbone overlay of all ***i*+1** analogs (**2,10-16)**, showing both front (left panel) and side (right panel) view.

References:

1. Carpino, L. A.; Han, G. Y., *J. Org. Chem.* **1972,** *37* , 3404-3409.
2. Chatterjee, J.; Laufer, B.; Kessler, H., *Nat. Prot.* **2012,** *7*, 432-444.
3. Carpino, L. A., *J. Am. Chem. Soc.* **1993,** *115* , 4397-4398.
4. Campbell, C. D.; Concellón, C.; Smith, A. D., *Tet. Asymm.* **2011,** *22*, 797-811.
5. T. D. Goddard and D. G. Kneller, SPARKY 3, University of California, San Francisco.
6. Brooks, B. R.; Bruccoleri, R. E.; Olafson, B. D.; States, D. J.; Swaminathan, S.; Karplus, M., *J. Comput. Chem* **1983,** *4*, 187-217.
7. Beck, J. G.; Chatterjee, J.; Laufer, B.; Kiran, M. U.; Frank, A. O.; Neubauer, S.; Ovadia, O.; Greenberg, S.; Gilon, C.; Hoffman, A.; Kessler, H., *J. Am. Chem. Soc.* **2012,** *134*, 12125-12133.
8. Bystrov, V. F.; Ivanov, V. T.; Portnova, S. L.; Balashova, T. A.; Ovchinnikov, Y. A., *Tetrahedron* **1973,** *29*, 873-877.
9. Mas-Moruno, C.; Beck, J. G.; Doedens, L.; Frank, A. O.; Marinelli, L.; Cosconati, S.; Novellino, E.; Kessler, H., *Angew. Chem., Int. Ed.* **2011,** *50*, 9496-9500.
